# Large scale statistical inference of signaling pathways from RNAi and microarray data

**DOI:** 10.1186/1471-2105-8-386

**Published:** 2007-10-15

**Authors:** Holger Froehlich, Mark Fellmann, Holger Sueltmann, Annemarie Poustka, Tim Beissbarth

**Affiliations:** 1 German Cancer Research Center (DKFZ), Im Neuenheimer Feld 580, 69120 Heidelberg, Germany

## Abstract

**Background:**

The advent of RNA interference techniques enables the selective silencing of biologically interesting genes in an efficient way. In combination with DNA microarray technology this enables researchers to gain insights into signaling pathways by observing downstream effects of individual knock-downs on gene expression. These secondary effects can be used to computationally reverse engineer features of the upstream signaling pathway.

**Results:**

In this paper we address this challenging problem by extending previous work by Markowetz *et al*., who proposed a statistical framework to score networks hypotheses in a Bayesian manner. Our extensions go in three directions: First, we introduce a way to omit the data discretization step needed in the original framework via a calculation based on *p*-values instead. Second, we show how prior assumptions on the network structure can be incorporated into the scoring scheme using regularization techniques. Third and most important, we propose methods to scale up the original approach, which is limited to around 5 genes, to large scale networks.

**Conclusion:**

Comparisons of these methods on artificial data are conducted. Our proposed module network is employed to infer the signaling network between 13 genes in the ER-*α *pathway in human MCF-7 breast cancer cells. Using a bootstrapping approach this reconstruction can be found with good statistical stability.

The code for the module network inference method is available in the latest version of the *R*-package *nem*, which can be obtained from the Bioconductor homepage.

## Background

In the modern field of systems biology scientists aim to get insights into the architecture and behavior of complex cellular and genomic processes. An important task in this context is the detection of novel interdependencies between gene products. This insight into the genomic networks is an important step towards a better understanding of the functional aspects of a biological system and of great value for drug target identification at a later stage. Within this context modern DNA microarray technology plays an important role. In addition, the advent of RNA silencing techniques has further increased its power by allowing the selective knock-down of certain genes of interest. This may enable us to detect interdependencies between gene products on a non-transcriptional level. The genes of interest are knocked down individually, and the respective downstream effects on gene expression are measured by using genome-wide microarrays. By observing the nested structure of significant up or down regulations of affected genes, this may allow one to reverse engineer features of the upstream signaling pathway [1]. In a recent work Markowetz *et al*. [2] introduced a method to reverse engineer the signaling pathway between perturbed genes using the nested structure of secondary downstream effects. They developed a Bayesian statistical framework, in which for a given network hypothesis one can calculate a score and thus can reduce the set of all possible networks to the most likely ones. A severe limitation of this method lies, however, in the restriction to small networks of up to 5 genes, because the method completely enumerates all possible network hypotheses. Furthermore, a difficulty in the practical use is the required binary discretization of the data ("secondary effect present/not present").

In our work we therefore aim to extend the framework by Markowetz *et al*. in order to make it practically applicable for a broader range of real life problems. We are thereby motivated by biological experiments conducted in our department: 13 genes in the ER-*α *pathway in human MCF-7 breast cancer cells were silenced via small interfering RNAs and the effects on gene expression were subsequently measured on cDNA microarrays. Our extensions of the original approach go in three directions: First, we introduce a way to omit the data discretization step needed via a calculation based on *p*-values instead, which is more suitable for our data and makes the whole framework more flexible (*generalized inference framework*). Second, we show how prior assumptions on the network structure can be incorporated into the network scoring scheme via techniques from regularization theory [3]. Third and most important, we develop and investigate methods to scale up the network inference to large scale networks. For this purpose two approaches are considered: simulated annealing on a restricted set of possible networks and our so-called *module networks*, which build the complete network recursively from smaller pieces that are connected subsequently. In order to validate these approaches we conduct studies on artificially created networks and show that module networks offer the highest sensitivity and specificity in the reconstruction of edges in the networks. Finally, we demonstrate the applicability of our approach to real data by inferring the complete 13 genes ER-*α *signaling pathway network. Using a bootstrapping approach this reconstruction can be found with good statistical stability and hence seems to be reliable.

## Results and discussion

### Statistical Inference Framework for Signaling Pathways from RNAi Data

**Figure 1:**
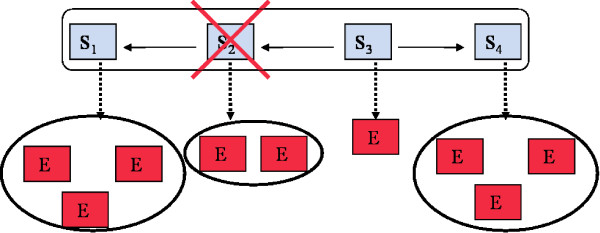
Main idea of the inference framework by Markowetz *et al*.: A network hypothesis is a directed graph between S-genes. Attached to each S-gene are several E-genes. Knocking down S-gene *S*_2 _interrupts signal flow in the downstream pathway, and hence an effect of E-genes attached to *S*_2 _and to *S*_1 _is expected.

We start with a brief review of the statistical inference framework for signaling pathways by Markowetz *et al*.: In general within this framework one distinguishes between silenced genes (S-genes) and genes showing a downstream effect (E-genes). It is assumed that each E-gene is attached to a single S-gene only (Figure 1). Knocking down a specific S-gene *S*_*k *_interrupts signal flow in the downstream pathway, and hence an effect on the E-genes attached to *S*_*k *_and all S-genes depending on *S*_*k *_is expected. Let us assume *n *knock-downs are performed and there exist *m *E-genes in total. The outcomes of these experiments are summarized in an *m *× *n *data matrix *D*. According to Bayes' formula a specific network hypothesis Φ ∈ {0,1}^*n *^× {0,1}^*n *^can be scored as:

*P*(Φ|*D*) ∝ *P*(*D|Φ*)*P*(Φ)

The position of the E-genes is introduced as a model parameter Θ = {*θ*_*i*_|*θ*_*i *_∈ {1,..., *n*}, *i *= 1,..., *m*}, i.e *θ*_*i *_= *j*, if E-gene *i *is attached to S-gene *j*. Assuming independence of the observations (rows) *D*_*i *_in the data matrix *D *(given a fixed network hypothesis Φ and model parameters Θ) one can write down the conditional likelihood *P*(*D*)|Φ, Θ) as:

P(D|Φ,Θ)=∏i=1mP(Di|Φ,θi)
 MathType@MTEF@5@5@+=feaafiart1ev1aaatCvAUfKttLearuWrP9MDH5MBPbIqV92AaeXatLxBI9gBaebbnrfifHhDYfgasaacH8akY=wiFfYdH8Gipec8Eeeu0xXdbba9frFj0=OqFfea0dXdd9vqai=hGuQ8kuc9pgc9s8qqaq=dirpe0xb9q8qiLsFr0=vr0=vr0dc8meaabaqaciaacaGaaeqabaqabeGadaaakeaacqWGqbaucqGGOaakcqWGebarcqGG8baFcqqHMoGrcqGGSaalcqqHyoqucqGGPaqkcqGH9aqpdaqeWbqaaiabdcfaqjabcIcaOiabdseaenaaBaaaleaacqWGPbqAaeqaaOGaeiiFaWNaeuOPdyKaeiilaWccciGae8hUde3aaSbaaSqaaiabdMgaPbqabaGccqGGPaqkaSqaaiabdMgaPjabg2da9iabigdaXaqaaiabd2gaTbqdcqGHpis1aaaa@4A79@

It is furthermore assumed that all parameters *θ*_*i *_are statistically independent, i.e.

P(Θ|Φ)=∏i=1mP(θi|Φ)
 MathType@MTEF@5@5@+=feaafiart1ev1aaatCvAUfKttLearuWrP9MDH5MBPbIqV92AaeXatLxBI9gBaebbnrfifHhDYfgasaacH8akY=wiFfYdH8Gipec8Eeeu0xXdbba9frFj0=OqFfea0dXdd9vqai=hGuQ8kuc9pgc9s8qqaq=dirpe0xb9q8qiLsFr0=vr0=vr0dc8meaabaqaciaacaGaaeqabaqabeGadaaakeaacqWGqbaucqGGOaakcqqHyoqucqGG8baFcqqHMoGrcqGGPaqkcqGH9aqpdaqeWbqaaiabdcfaqjabcIcaOGGaciab=H7aXnaaBaaaleaacqWGPbqAaeqaaOGaeiiFaWNaeuOPdyKaeiykaKcaleaacqWGPbqAcqGH9aqpcqaIXaqmaeaacqWGTbqBa0Gaey4dIunaaaa@4506@

The likelihood *P*(*D*|Φ) can now be written as:

*P*(*D*|Φ) = ∫*P*(*D*|Φ, Θ) *P*(Θ|Φ)*d*Θ

=∏i=1m∑j=1nP(Di|Φ,θi=j)P(θi=j|Φ)
 MathType@MTEF@5@5@+=feaafiart1ev1aaatCvAUfKttLearuWrP9MDH5MBPbIqV92AaeXatLxBI9gBaebbnrfifHhDYfgasaacH8akY=wiFfYdH8Gipec8Eeeu0xXdbba9frFj0=OqFfea0dXdd9vqai=hGuQ8kuc9pgc9s8qqaq=dirpe0xb9q8qiLsFr0=vr0=vr0dc8meaabaqaciaacaGaaeqabaqabeGadaaakeaacqGH9aqpdaqeWbqaamaaqahabaGaemiuaaLaeiikaGIaemiraq0aaSbaaSqaaiabdMgaPbqabaGccqGG8baFcqqHMoGrcqGGSaaliiGacqWF4oqCdaWgaaWcbaGaemyAaKgabeaakiabg2da9iabdQgaQjabcMcaPiabdcfaqjabcIcaOiab=H7aXnaaBaaaleaacqWGPbqAaeqaaOGaeyypa0JaemOAaOMaeiiFaWNaeuOPdyKaeiykaKcaleaacqWGQbGAcqGH9aqpcqaIXaqmaeaacqWGUbGBa0GaeyyeIuoaaSqaaiabdMgaPjabg2da9iabigdaXaqaaiabd2gaTbqdcqGHpis1aaaa@5613@

Please note that the edges in network Φ can either represent transcriptional regulation events or phosphorilation or post-translational effects, as we reconstruct the signal flow in the network based on the nested structure of the measured effects. The effects on the E-genes that are measured are transcriptional effects, which are ultimately regulated by transcription factors. Some E-genes may be regulated by kinases, as due to the inherent nature of microarray measurements, it is impossible to distinguish between direct and indirect effects.

### Our Approach

#### Generalized Inference Framework

In their original work Markowetz et al. suppose the data matrix D to consist of counts, how often a specific E-gene shows an effect in ℓ experiment repetitions. This requires a data discretization step, for which user specified type-I and type-II error rates are assumed. The choice of these parameters is certainly critical for the inference procedure, because it directly influences (5) and appears to be difficult to estimate. Markowetz *et al*. suppose to have both, positive and negative controls (pathway stimulated/not stimulated) for this procedure, which for our data is not available (see Section "Methods"). In contrast, in our approach we make the assumption that *D *is an *m *× *n *matrix of (raw) *p*-values, which specify the likelihood of E-gene *i *being differentially expressed after knock-down of S-gene *k*. The *p*-values are calculated using a method for detecting differential gene expression, e.g. *limma *[4]. This way various experimental designs, including dye swaps, on arbitrary chip platforms can be used in a simple manner.

We now suppose a decomposition of *P*(*D*_*i*_|Φ,*θ*_*i*_) as follows:

P(Di|Φ,θi)=∏k=1nP(Dik|Φ,θi)
 MathType@MTEF@5@5@+=feaafiart1ev1aaatCvAUfKttLearuWrP9MDH5MBPbIqV92AaeXatLxBI9gBaebbnrfifHhDYfgasaacH8akY=wiFfYdH8Gipec8Eeeu0xXdbba9frFj0=OqFfea0dXdd9vqai=hGuQ8kuc9pgc9s8qqaq=dirpe0xb9q8qiLsFr0=vr0=vr0dc8meaabaqaciaacaGaaeqabaqabeGadaaakeaacqWGqbaucqGGOaakcqWGebardaWgaaWcbaGaemyAaKgabeaakiabcYha8jabfA6agjabcYcaSGGaciab=H7aXnaaBaaaleaacqWGPbqAaeqaaOGaeiykaKIaeyypa0ZaaebCaeaacqWGqbaucqGGOaakcqWGebardaWgaaWcbaGaemyAaKMaem4AaSgabeaakiabcYha8jabfA6agjabcYcaSiab=H7aXnaaBaaaleaacqWGPbqAaeqaaOGaeiykaKcaleaacqWGRbWAcqGH9aqpcqaIXaqmaeaacqWGUbGBa0Gaey4dIunaaaa@4F3A@

In accordance to [2] this makes the assumption that knock-down experiments are statistically independent from each other. Hence, Eq. (5) can be written down as

P(D|Φ)=∏i=1m∑j=1n∏k=1nP(Dik|Φ,θi=j)P(θi=j|Φ)
 MathType@MTEF@5@5@+=feaafiart1ev1aaatCvAUfKttLearuWrP9MDH5MBPbIqV92AaeXatLxBI9gBaebbnrfifHhDYfgasaacH8akY=wiFfYdH8Gipec8Eeeu0xXdbba9frFj0=OqFfea0dXdd9vqai=hGuQ8kuc9pgc9s8qqaq=dirpe0xb9q8qiLsFr0=vr0=vr0dc8meaabaqaciaacaGaaeqabaqabeGadaaakeaacqWGqbaucqGGOaakcqWGebarcqGG8baFcqqHMoGrcqGGPaqkcqGH9aqpdaqeWbqaamaaqahabaWaaebCaeaacqWGqbaucqGGOaakcqWGebardaWgaaWcbaGaemyAaKMaem4AaSgabeaakiabcYha8jabfA6agjabcYcaSGGaciab=H7aXnaaBaaaleaacqWGPbqAaeqaaOGaeyypa0JaemOAaOMaeiykaKIaemiuaaLaeiikaGIae8hUde3aaSbaaSqaaiabdMgaPbqabaGccqGH9aqpcqWGQbGAcqGG8baFcqqHMoGrcqGGPaqkaSqaaiabdUgaRjabg2da9iabigdaXaqaaiabd6gaUbqdcqGHpis1aaWcbaGaemOAaOMaeyypa0JaeGymaedabaGaemOBa4ganiabggHiLdaaleaacqWGPbqAcqGH9aqpcqaIXaqmaeaacqWGTbqBa0Gaey4dIunaaaa@6543@

The only thing missing is the definition of *P*(*D*_*ik*_|Φ,*θ*_*i*_). For this purpose we suppose the *D*_*ik *_to be drawn from a mixture of a uniform [0, 1] distribution reflecting the null hypothesis and another distribution *f*_1 _reflecting the alternative hypothesis [5-7]:

*P*(*D*_*ik*_) = *γ*_*k *_+ (1 - *γ*_*k*_)·*f*_1_(*D*_*ik*_), *γ*_*k *_∈ (0,1)

Under the alternative hypothesis there is a high density for small *p*-values and a strong decrease for increasing *p*-values. Both distributions overlap with mixing coefficient *γ*_*k*_. *P*(*D*_*ik*_|Φ,*θ*_*i*_) can therefore be decomposed as:

P(Dik|Φ,θi)={f1(Dik)if Φ predicts an effect1otherwise
 MathType@MTEF@5@5@+=feaafiart1ev1aaatCvAUfKttLearuWrP9MDH5MBPbIqV92AaeXatLxBI9gBaebbnrfifHhDYfgasaacH8akY=wiFfYdH8Gipec8Eeeu0xXdbba9frFj0=OqFfea0dXdd9vqai=hGuQ8kuc9pgc9s8qqaq=dirpe0xb9q8qiLsFr0=vr0=vr0dc8meaabaqaciaacaGaaeqabaqabeGadaaakeaacqWGqbaucqGGOaakcqWGebardaWgaaWcbaGaemyAaKMaem4AaSgabeaakiabcYha8jabfA6agjabcYcaSGGaciab=H7aXnaaBaaaleaacqWGPbqAaeqaaOGaeiykaKIaeyypa0ZaaiqabeaafaqaaeGacaaabaGaemOzay2aaSbaaSqaaiabigdaXaqabaGccqGGOaakcqWGebardaWgaaWcbaGaemyAaKMaem4AaSgabeaakiabcMcaPaqaaiabbMgaPjabbAgaMjabbccaGiabfA6agjabbccaGiabbchaWjabbkhaYjabbwgaLjabbsgaKjabbMgaPjabbogaJjabbsha0jabbohaZjabbccaGiabbggaHjabb6gaUjabbccaGiabbwgaLjabbAgaMjabbAgaMjabbwgaLjabbogaJjabbsha0bqaaiabigdaXaqaaiabb+gaVjabbsha0jabbIgaOjabbwgaLjabbkhaYjabbEha3jabbMgaPjabbohaZjabbwgaLbaaaiaawUhaaaaa@6F57@

The density function *f*_1 _reflects the strength of the knock-down effect on E-gene *i *under the alternative hypothesis. If it is greater than 1 the alternative hypothesis would be accepted, and if it is smaller than 1 rejected. In this work we assume *f*_1 _to be a mixture of a Beta(1, *β*_*k*_) distribution (*β*_*k *_≫ 2) and a small uniform component:

*f*_1_(*D*_*ik*_) = *π*_*k *_+ (1 - *π*_*k*_)Beta(*D*_*ik*_, 1, *β*_*k*_)

In practice we set *π*_*k *_= 0.01 and tuned the parameter *β*_*k *_on the full distribution of raw *p*-values for knock-down experiment *k *(26709 genes) such that *f*_1_(*D*_*ik*_) > 1, if the Benjamini-Hochberg false discovery rate [8] for *D*_*ik *_was ≤ 10% and *f*_1_(*D*_*ik*_) ≤ 1 otherwise. An alternative treatment using a fitting procedure with Expectation Maximization [9] is described in our recent publication [10].

### Regularization

Eq. (1) allows one to specify a prior *P*(Φ) on the network structure itself. This can be thought of as biasing the score of possible network hypotheses towards prior knowledge. It is crucial to understand that in principle in any inference scheme there exist two competing goals: Belief in prior assumptions/prior knowledge versus belief in data. Only trusting the data itself may lead to overfitting, whereas only trusting in prior assumptions does not give any new information and prevents learning. Therefore, we need a trade-off between both goals. This technique is known as *regularization *in the machine learning literature [3,11]. We have to take into account at this point that our assumptions may only be true up to a certain degree. Hence, for each edge we should suppose a prior probability reflecting the degree of belief in its existence. In principle, this degree of belief can be very different for each edge. We summarize all prior edge probabilities in an *n *× *n *matrix Φ^
 MathType@MTEF@5@5@+=feaafiart1ev1aaatCvAUfKttLearuWrP9MDH5MBPbIqV92AaeXatLxBI9gBaebbnrfifHhDYfgasaacH8akY=wiFfYdH8Gipec8Eeeu0xXdbba9frFj0=OqFfea0dXdd9vqai=hGuQ8kuc9pgc9s8qqaq=dirpe0xb9q8qiLsFr0=vr0=vr0dc8meaabaqaciaacaGaaeqabaqabeGadaaakeaacuqHMoGrgaqcaaaa@2E36@. Making the assumption that all edge priors *P*(Φ_*ij*_) are independent, i.e.

P(Φ)=∏i,jP(Φij)
 MathType@MTEF@5@5@+=feaafiart1ev1aaatCvAUfKttLearuWrP9MDH5MBPbIqV92AaeXatLxBI9gBaebbnrfifHhDYfgasaacH8akY=wiFfYdH8Gipec8Eeeu0xXdbba9frFj0=OqFfea0dXdd9vqai=hGuQ8kuc9pgc9s8qqaq=dirpe0xb9q8qiLsFr0=vr0=vr0dc8meaabaqaciaacaGaaeqabaqabeGadaaakeaacqWGqbaucqGGOaakcqqHMoGrcqGGPaqkcqGH9aqpdaqeqbqaaiabdcfaqjabcIcaOiabfA6agnaaBaaaleaacqWGPbqAcqWGQbGAaeqaaOGaeiykaKcaleaacqWGPbqAcqGGSaalcqWGQbGAaeqaniabg+Givdaaaa@3EF4@

allows us to define the connection between Φ_*ij *_and Φ^ij
 MathType@MTEF@5@5@+=feaafiart1ev1aaatCvAUfKttLearuWrP9MDH5MBPbIqV92AaeXatLxBI9gBaebbnrfifHhDYfgasaacH8akY=wiFfYdH8Gipec8Eeeu0xXdbba9frFj0=OqFfea0dXdd9vqai=hGuQ8kuc9pgc9s8qqaq=dirpe0xb9q8qiLsFr0=vr0=vr0dc8meaabaqaciaacaGaaeqabaqabeGadaaakeaacuqHMoGrgaqcamaaBaaaleaacqWGPbqAcqWGQbGAaeqaaaaa@311A@ for each edge separately. Note that Φ_*ij *_∈ {0,1} depending on whether we set the edge *i *→ *j *or not. Hence, for each edge we have a certain difference |Φij−Φ^ij|
 MathType@MTEF@5@5@+=feaafiart1ev1aaatCvAUfKttLearuWrP9MDH5MBPbIqV92AaeXatLxBI9gBaebbnrfifHhDYfgasaacH8akY=wiFfYdH8Gipec8Eeeu0xXdbba9frFj0=OqFfea0dXdd9vqai=hGuQ8kuc9pgc9s8qqaq=dirpe0xb9q8qiLsFr0=vr0=vr0dc8meaabaqaciaacaGaaeqabaqabeGadaaakeaadaabdaqaaiabfA6agnaaBaaaleaacqWGPbqAcqWGQbGAaeqaaOGaeyOeI0IafuOPdyKbaKaadaWgaaWcbaGaemyAaKMaemOAaOgabeaaaOGaay5bSlaawIa7aaaa@399B@ to our prior assumptions. The smaller this difference, the higher *P*(Φ_*ij*_) should be. We can therefore model *P*(Φ_*ij*_) as a Laplacian distribution with parameter *λ *:

P(Φij|λ)=λ2exp⁡(−λ|Φij−Φ^ij|)
 MathType@MTEF@5@5@+=feaafiart1ev1aaatCvAUfKttLearuWrP9MDH5MBPbIqV92AaeXatLxBI9gBaebbnrfifHhDYfgasaacH8akY=wiFfYdH8Gipec8Eeeu0xXdbba9frFj0=OqFfea0dXdd9vqai=hGuQ8kuc9pgc9s8qqaq=dirpe0xb9q8qiLsFr0=vr0=vr0dc8meaabaqaciaacaGaaeqabaqabeGadaaakeaacqWGqbaucqGGOaakcqqHMoGrdaWgaaWcbaGaemyAaKMaemOAaOgabeaakiabcYha8HGaciab=T7aSjabcMcaPiabg2da9maalaaabaGae83UdWgabaGaeGOmaidaaiGbcwgaLjabcIha4jabcchaWnaabmaabaGaeyOeI0Iae83UdW2aaqWaaeaacqqHMoGrdaWgaaWcbaGaemyAaKMaemOAaOgabeaakiabgkHiTiqbfA6agzaajaWaaSbaaSqaaiabdMgaPjabdQgaQbqabaaakiaawEa7caGLiWoaaiaawIcacaGLPaaaaaa@5029@

If we now write down the log-posterior of Eq. (1)

log *P*(Φ|*D*) ∝ log *P*(*D*|Φ) + log *P*(Φ)

∝log⁡P(D|Φ)−λ∑i,j|Φij−Φ^ij|
 MathType@MTEF@5@5@+=feaafiart1ev1aaatCvAUfKttLearuWrP9MDH5MBPbIqV92AaeXatLxBI9gBaebbnrfifHhDYfgasaacH8akY=wiFfYdH8Gipec8Eeeu0xXdbba9frFj0=OqFfea0dXdd9vqai=hGuQ8kuc9pgc9s8qqaq=dirpe0xb9q8qiLsFr0=vr0=vr0dc8meaabaqaciaacaGaaeqabaqabeGadaaakeaacqGHDisTcyGGSbaBcqGGVbWBcqGGNbWzcqWGqbaucqGGOaakcqWGebarcqGG8baFcqqHMoGrcqGGPaqkcqGHsisliiGacqWF7oaBdaaeqbqaamaaemaabaGaeuOPdy0aaSbaaSqaaiabdMgaPjabdQgaQbqabaGccqGHsislcuqHMoGrgaqcamaaBaaaleaacqWGPbqAcqWGQbGAaeqaaaGccaGLhWUaayjcSdaaleaacqWGPbqAcqGGSaalcqWGQbGAaeqaniabggHiLdaaaa@4E82@

we see that *λ *specifies the trade-off between the model's fit to our data and our prior assumptions. An important special case of the latter would be Φ^
 MathType@MTEF@5@5@+=feaafiart1ev1aaatCvAUfKttLearuWrP9MDH5MBPbIqV92AaeXatLxBI9gBaebbnrfifHhDYfgasaacH8akY=wiFfYdH8Gipec8Eeeu0xXdbba9frFj0=OqFfea0dXdd9vqai=hGuQ8kuc9pgc9s8qqaq=dirpe0xb9q8qiLsFr0=vr0=vr0dc8meaabaqaciaacaGaaeqabaqabeGadaaakeaacuqHMoGrgaqcaaaa@2E36@ = 0, i.e. the matrix consisting only of zeros. The meaning of this prior would be to generally prefer sparse networks structures over dense ones. Setting *λ *→ ∞ corresponds to completely trusting the prior, whereas *λ *= 0 leads to a maximum likelihood estimate, i.e. complete trust in data. In practice we would like to choose a *λ *balancing both goals. This leads to an instance of the classical *model selection *problem (e.g. [12]) in statistical learning. One way of dealing with it is to tune *λ *such that the *Akaike information criterion *(AIC)

*AIC*(*λ*, Φ_*opt*_) = -2 log *P*(*D*|Φ_*opt*_) + 2*d*(*λ*, Φ_*opt*_)

becomes minimal [12]. Here *d*(*λ*, Φ_*opt*_) denotes the number of free parameters (i.e. the number of unknown edges) in the network structure Φ_*opt *_optimizing (14).

### Large Scale Network Inference

The inference framework does not answer the question how to come up with a candidate network topology, which we would like to score. Markowetz *et al*. [2] completely enumerate all possible topologies. This is, however, only suitable for small networks of up to 5 S-genes. For 5 S-genes there already exist more than 1,000,000 and for 10 genes more than 10^27 ^possible network topologies. In this context it should be noted that the scoring scheme cannot distinguish between two network hypotheses, if they only differ in transitive edges. This issue is known as *prediction equivalence*. Hence, it only makes sense to consider the set of all transitively closed network hypotheses. However, restricting ourselves to this limited class of network structures does not generally solve the problem, since even then the number of networks to consider scales in a similar way with the number of S-genes (for 5 genes there are already more than 6,000 transitively closed networks to test). Hence, we have to resort to heuristics.

#### Stochastic Sampling

A quite obvious idea to prevent the computational effort to enumerate all possible network hypothesis is to sample from the set of all transitively closed network graphs randomly. We decided to use simulated annealing (SA) here [13]. SA is rather similar to Markov chain Monte Carlo (MCMC) sampling [14], but additionally makes use of a so-called cooling scheme, which gradually decreases the neighborhood size of a given state in search space. SA has been successfully applied to many difficult optimization problems from various disciplines, including bioinformatics [15,16]. In order to use SA, we have to define a state transition function *t *: *S *→ *S*, which defines how to come from one graph to a modified one in search space. A special challenge in this context is that we need to guarantee that in principle all possible transitively closed network topologies can be reached by our function *t*.

Supposed we have functions *add *and *del*, which add and remove edges from a given transitively closed graph and produce a new one from this. We will restrict ourselves to the set of all transitively closed directed graphs (DAGs) here for reasons that will become clear soon. We now define *add *and *del *in a formal way as follows:

#### Definition 1

Let Tn
 MathType@MTEF@5@5@+=feaafiart1ev1aaatCvAUfKttLearuWrP9MDH5MBPbIqV92AaeXatLxBI9gBaebbnrfifHhDYfgasaacH8akY=wiFfYdH8Gipec8Eeeu0xXdbba9frFj0=OqFfea0dXdd9vqai=hGuQ8kuc9pgc9s8qqaq=dirpe0xb9q8qiLsFr0=vr0=vr0dc8meaabaqaciaacaGaaeqabaqabeGadaaakeaat0uy0HwzTfgDPnwy1egaryqtHrhAL1wy0L2yHvdaiqaacqWFtepvdaWgaaWcbaGaemOBa4gabeaaaaa@39D7@ be the set of all transitively closed graphs DAGs with *n *nodes. Suppose the nodes for *G *∈ Tn
 MathType@MTEF@5@5@+=feaafiart1ev1aaatCvAUfKttLearuWrP9MDH5MBPbIqV92AaeXatLxBI9gBaebbnrfifHhDYfgasaacH8akY=wiFfYdH8Gipec8Eeeu0xXdbba9frFj0=OqFfea0dXdd9vqai=hGuQ8kuc9pgc9s8qqaq=dirpe0xb9q8qiLsFr0=vr0=vr0dc8meaabaqaciaacaGaaeqabaqabeGadaaakeaat0uy0HwzTfgDPnwy1egaryqtHrhAL1wy0L2yHvdaiqaacqWFtepvdaWgaaWcbaGaemOBa4gabeaaaaa@39D7@ are indexed in some way by natural numbers. We define *add *: Tn×ℰi→Tn
 MathType@MTEF@5@5@+=feaafiart1ev1aaatCvAUfKttLearuWrP9MDH5MBPbIqV92AaeXatLxBI9gBaebbnrfifHhDYfgasaacH8akY=wiFfYdH8Gipec8Eeeu0xXdbba9frFj0=OqFfea0dXdd9vqai=hGuQ8kuc9pgc9s8qqaq=dirpe0xb9q8qiLsFr0=vr0=vr0dc8meaabaqaciaacaGaaeqabaqabeGadaaakeaat0uy0HwzTfgDPnwy1egaryqtHrhAL1wy0L2yHvdaiqaacqWFtepvdaWgaaWcbaGaemOBa4gabeaakiabgEna0kab=btifnaaBaaaleaacqWGPbqAaeqaaOGaeyOKH4Qae83eXt1aaSbaaSqaaiabd6gaUbqabaaaaa@43FF@ as a function, which takes a graph *G *and a pair of node indices (*i*, *j*) ∈ ℰ1
 MathType@MTEF@5@5@+=feaafiart1ev1aaatCvAUfKttLearuWrP9MDH5MBPbIqV92AaeXatLxBI9gBaebbnrfifHhDYfgasaacH8akY=wiFfYdH8Gipec8Eeeu0xXdbba9frFj0=OqFfea0dXdd9vqai=hGuQ8kuc9pgc9s8qqaq=dirpe0xb9q8qiLsFr0=vr0=vr0dc8meaabaqaciaacaGaaeqabaqabeGadaaakeaat0uy0HwzTfgDPnwy1egaryqtHrhAL1wy0L2yHvdaiqaacqWFWesrdaWgaaWcbaGaeGymaedabeaaaaa@38A0@ ⊂ ℕ × ℕ, inserts the edge between *i*, *j *and transitively closes *G *then. ℰ1
 MathType@MTEF@5@5@+=feaafiart1ev1aaatCvAUfKttLearuWrP9MDH5MBPbIqV92AaeXatLxBI9gBaebbnrfifHhDYfgasaacH8akY=wiFfYdH8Gipec8Eeeu0xXdbba9frFj0=OqFfea0dXdd9vqai=hGuQ8kuc9pgc9s8qqaq=dirpe0xb9q8qiLsFr0=vr0=vr0dc8meaabaqaciaacaGaaeqabaqabeGadaaakeaat0uy0HwzTfgDPnwy1egaryqtHrhAL1wy0L2yHvdaiqaacqWFWesrdaWgaaWcbaGaeGymaedabeaaaaa@38A0@ is defined as the set of all pairs of node indices, for which there exists no edge in *G *and which after edge insertion do not violate the DAG property. Likewise, *del *: Tn×ℰ2→Tn
 MathType@MTEF@5@5@+=feaafiart1ev1aaatCvAUfKttLearuWrP9MDH5MBPbIqV92AaeXatLxBI9gBaebbnrfifHhDYfgasaacH8akY=wiFfYdH8Gipec8Eeeu0xXdbba9frFj0=OqFfea0dXdd9vqai=hGuQ8kuc9pgc9s8qqaq=dirpe0xb9q8qiLsFr0=vr0=vr0dc8meaabaqaciaacaGaaeqabaqabeGadaaakeaat0uy0HwzTfgDPnwy1egaryqtHrhAL1wy0L2yHvdaiqaacqWFtepvdaWgaaWcbaGaemOBa4gabeaakiabgEna0kab=btifnaaBaaaleaacqaIYaGmaeqaaOGaeyOKH4Qae83eXt1aaSbaaSqaaiabd6gaUbqabaaaaa@4396@ is a function, which takes a graph *G *and a pair of node indices (*i*, *j*) ∈ ℰ2
 MathType@MTEF@5@5@+=feaafiart1ev1aaatCvAUfKttLearuWrP9MDH5MBPbIqV92AaeXatLxBI9gBaebbnrfifHhDYfgasaacH8akY=wiFfYdH8Gipec8Eeeu0xXdbba9frFj0=OqFfea0dXdd9vqai=hGuQ8kuc9pgc9s8qqaq=dirpe0xb9q8qiLsFr0=vr0=vr0dc8meaabaqaciaacaGaaeqabaqabeGadaaakeaat0uy0HwzTfgDPnwy1egaryqtHrhAL1wy0L2yHvdaiqaacqWFWesrdaWgaaWcbaGaeGOmaidabeaaaaa@38A2@ ⊂ ℕ × ℕ. *G *is transitively reduced, the edge between *i*, *j *deleted and then transitively closed again. With a transitive reduction G* of a graph *G *∈ Tn
 MathType@MTEF@5@5@+=feaafiart1ev1aaatCvAUfKttLearuWrP9MDH5MBPbIqV92AaeXatLxBI9gBaebbnrfifHhDYfgasaacH8akY=wiFfYdH8Gipec8Eeeu0xXdbba9frFj0=OqFfea0dXdd9vqai=hGuQ8kuc9pgc9s8qqaq=dirpe0xb9q8qiLsFr0=vr0=vr0dc8meaabaqaciaacaGaaeqabaqabeGadaaakeaat0uy0HwzTfgDPnwy1egaryqtHrhAL1wy0L2yHvdaiqaacqWFtepvdaWgaaWcbaGaemOBa4gabeaaaaa@39D7@ we mean a graph with a minimal number of edges such that the transitive closure of G* is *G*. ℰ2
 MathType@MTEF@5@5@+=feaafiart1ev1aaatCvAUfKttLearuWrP9MDH5MBPbIqV92AaeXatLxBI9gBaebbnrfifHhDYfgasaacH8akY=wiFfYdH8Gipec8Eeeu0xXdbba9frFj0=OqFfea0dXdd9vqai=hGuQ8kuc9pgc9s8qqaq=dirpe0xb9q8qiLsFr0=vr0=vr0dc8meaabaqaciaacaGaaeqabaqabeGadaaakeaat0uy0HwzTfgDPnwy1egaryqtHrhAL1wy0L2yHvdaiqaacqWFWesrdaWgaaWcbaGaeGOmaidabeaaaaa@38A2@ is defined as the set of all pairs of node indices, for which there exists an edge in *G*.

In contrast to general graphs the transitive reduction of a DAG is unique [17], which is the reason for our restriction. This way we can guarantee that the *del *function is well defined and injective. This gives rise to the following lemma:

#### Lemma 2

*The operations add *: Tn×ℰ1→T′n
 MathType@MTEF@5@5@+=feaafiart1ev1aaatCvAUfKttLearuWrP9MDH5MBPbIqV92AaeXatLxBI9gBaebbnrfifHhDYfgasaacH8akY=wiFfYdH8Gipec8Eeeu0xXdbba9frFj0=OqFfea0dXdd9vqai=hGuQ8kuc9pgc9s8qqaq=dirpe0xb9q8qiLsFr0=vr0=vr0dc8meaabaqaciaacaGaaeqabaqabeGadaaakeaat0uy0HwzTfgDPnwy1egaryqtHrhAL1wy0L2yHvdaiqaacqWFtepvdaWgaaWcbaGaemOBa4gabeaakiabgEna0kab=btifnaaBaaaleaacqaIXaqmaeqaaOGaeyOKH4Qaf83eXtLbauaadaWgaaWcbaGaemOBa4gabeaaaaa@43A0@*and del *: Tn×ℰ2→T′n
 MathType@MTEF@5@5@+=feaafiart1ev1aaatCvAUfKttLearuWrP9MDH5MBPbIqV92AaeXatLxBI9gBaebbnrfifHhDYfgasaacH8akY=wiFfYdH8Gipec8Eeeu0xXdbba9frFj0=OqFfea0dXdd9vqai=hGuQ8kuc9pgc9s8qqaq=dirpe0xb9q8qiLsFr0=vr0=vr0dc8meaabaqaciaacaGaaeqabaqabeGadaaakeaat0uy0HwzTfgDPnwy1egaryqtHrhAL1wy0L2yHvdaiqaacqWFtepvdaWgaaWcbaGaemOBa4gabeaakiabgEna0kab=btifnaaBaaaleaacqaIYaGmaeqaaOGaeyOKH4Qaf83eXtLbauaadaWgaaWcbaGaemOBa4gabeaaaaa@43A2@*have the following inverse property to each other: For any G *∈ Tn
 MathType@MTEF@5@5@+=feaafiart1ev1aaatCvAUfKttLearuWrP9MDH5MBPbIqV92AaeXatLxBI9gBaebbnrfifHhDYfgasaacH8akY=wiFfYdH8Gipec8Eeeu0xXdbba9frFj0=OqFfea0dXdd9vqai=hGuQ8kuc9pgc9s8qqaq=dirpe0xb9q8qiLsFr0=vr0=vr0dc8meaabaqaciaacaGaaeqabaqabeGadaaakeaat0uy0HwzTfgDPnwy1egaryqtHrhAL1wy0L2yHvdaiqaacqWFtepvdaWgaaWcbaGaemOBa4gabeaaaaa@39D7@*del*(*add*(*G*, *i*, *j*), *i*, *j*) = *G, provided that edge i, j does not exist in G and does not violate the DAG property. Likewise, add*(*del*(*G*, *i*, *j*), *i*, *j*) = *G, provided that edge i, j exists in G*.

*Proof*. The transitive reduction of a transitively closed DAG is unique [17]. Hence, the *del *operation is a well defined injective function. Additionally note that in the *add *operation we can never insert an edge, which lies in the transitive hull of *G *∈ Tn
 MathType@MTEF@5@5@+=feaafiart1ev1aaatCvAUfKttLearuWrP9MDH5MBPbIqV92AaeXatLxBI9gBaebbnrfifHhDYfgasaacH8akY=wiFfYdH8Gipec8Eeeu0xXdbba9frFj0=OqFfea0dXdd9vqai=hGuQ8kuc9pgc9s8qqaq=dirpe0xb9q8qiLsFr0=vr0=vr0dc8meaabaqaciaacaGaaeqabaqabeGadaaakeaat0uy0HwzTfgDPnwy1egaryqtHrhAL1wy0L2yHvdaiqaacqWFtepvdaWgaaWcbaGaemOBa4gabeaaaaa@39D7@ (and can thus be removed by a transitive reduction), since otherwise it would have been there already (because Tn
 MathType@MTEF@5@5@+=feaafiart1ev1aaatCvAUfKttLearuWrP9MDH5MBPbIqV92AaeXatLxBI9gBaebbnrfifHhDYfgasaacH8akY=wiFfYdH8Gipec8Eeeu0xXdbba9frFj0=OqFfea0dXdd9vqai=hGuQ8kuc9pgc9s8qqaq=dirpe0xb9q8qiLsFr0=vr0=vr0dc8meaabaqaciaacaGaaeqabaqabeGadaaakeaat0uy0HwzTfgDPnwy1egaryqtHrhAL1wy0L2yHvdaiqaacqWFtepvdaWgaaWcbaGaemOBa4gabeaaaaa@39D7@ consists of transitively closed networks only). Therefore, for any *G *∈ Tn
 MathType@MTEF@5@5@+=feaafiart1ev1aaatCvAUfKttLearuWrP9MDH5MBPbIqV92AaeXatLxBI9gBaebbnrfifHhDYfgasaacH8akY=wiFfYdH8Gipec8Eeeu0xXdbba9frFj0=OqFfea0dXdd9vqai=hGuQ8kuc9pgc9s8qqaq=dirpe0xb9q8qiLsFr0=vr0=vr0dc8meaabaqaciaacaGaaeqabaqabeGadaaakeaat0uy0HwzTfgDPnwy1egaryqtHrhAL1wy0L2yHvdaiqaacqWFtepvdaWgaaWcbaGaemOBa4gabeaaaaa@39D7@*del*(*add*(*G*, *i*, *j*), *i*, *j*) = *G*, provided that edge *i*, *j *does not exist in *G *and does not violate the DAG property. Likewise, *add*(*del*(*G*, *i*, *j*), *i*, *j*) = *G*, provided that edge *i*, *j *exists in *G*.   □

#### Theorem 3

*From any graph A *∈ Tn
 MathType@MTEF@5@5@+=feaafiart1ev1aaatCvAUfKttLearuWrP9MDH5MBPbIqV92AaeXatLxBI9gBaebbnrfifHhDYfgasaacH8akY=wiFfYdH8Gipec8Eeeu0xXdbba9frFj0=OqFfea0dXdd9vqai=hGuQ8kuc9pgc9s8qqaq=dirpe0xb9q8qiLsFr0=vr0=vr0dc8meaabaqaciaacaGaaeqabaqabeGadaaakeaat0uy0HwzTfgDPnwy1egaryqtHrhAL1wy0L2yHvdaiqaacqWFtepvdaWgaaWcbaGaemOBa4gabeaaaaa@39D7@*in the set of all transitively closed DAGs we can reach any other graph B *∈ Tn
 MathType@MTEF@5@5@+=feaafiart1ev1aaatCvAUfKttLearuWrP9MDH5MBPbIqV92AaeXatLxBI9gBaebbnrfifHhDYfgasaacH8akY=wiFfYdH8Gipec8Eeeu0xXdbba9frFj0=OqFfea0dXdd9vqai=hGuQ8kuc9pgc9s8qqaq=dirpe0xb9q8qiLsFr0=vr0=vr0dc8meaabaqaciaacaGaaeqabaqabeGadaaakeaat0uy0HwzTfgDPnwy1egaryqtHrhAL1wy0L2yHvdaiqaacqWFtepvdaWgaaWcbaGaemOBa4gabeaaaaa@39D7@*using add and del operations*.

*Proof*. Let 0 ∈ Tn
 MathType@MTEF@5@5@+=feaafiart1ev1aaatCvAUfKttLearuWrP9MDH5MBPbIqV92AaeXatLxBI9gBaebbnrfifHhDYfgasaacH8akY=wiFfYdH8Gipec8Eeeu0xXdbba9frFj0=OqFfea0dXdd9vqai=hGuQ8kuc9pgc9s8qqaq=dirpe0xb9q8qiLsFr0=vr0=vr0dc8meaabaqaciaacaGaaeqabaqabeGadaaakeaat0uy0HwzTfgDPnwy1egaryqtHrhAL1wy0L2yHvdaiqaacqWFtepvdaWgaaWcbaGaemOBa4gabeaaaaa@39D7@ be the DAG without any edges (but with *n *nodes). Let us denote by *X *→ *Y *that from DAG *X *we can get DAG *Y *using *add *and *del *operations on appropriate edges. Note that we have *A *→ 0 and *B *→ 0, because we can use *del *to remove edges successively until no edges are left. (The reasons for this property is that *del *removes edges from the transitive reduction of a DAG, which can thus not be inserted in the following transitive closure any more.) Because *add *and *del *are inverse operations to each other according to the lemma, we have 0 → *A*, 0 → *B*. Hence, we get *A *→ 0 → *B*, which proves the theorem.   □

Still, the SA approach suffers from a potential problem: Both, the *add *and the *del *operation, at the bottom line perform a whole cascade of changes on the original graph. Thus there may be harsh changes in the scoring function when applying such an operation to a given candidate network. This may make it difficult to come close to the optimal network hypothesis.

#### Module Networks

Rather than looking for a complete network hypothesis at once the idea of the module network is to build up a graph from smaller subgraphs, called *modules *in the following. The module network is thus a divide and conquer approach: We first split the complete node set into smaller subgroups. This can be done by PAM clustering [18] on the *p*-value density profiles of the S-genes. The idea is that S-genes with a similar E-gene response profile (here: with regard to the Manhattan distance) should be close in the signaling path. The number of clusters for the PAM clustering is chosen between 2 and half of the number of S-genes such that the average silhouette index becomes maximal. The silhouette value for each point in a cluster is a measure of how similar that point is to points in its own cluster vs. points in other clusters, and ranges from -1 to +1 [19]. It is defined as:

S(i)=min⁡j(d¯B(i,j))−d¯W(i)max⁡(d¯W(i),min⁡j(d¯B(i,j))
 MathType@MTEF@5@5@+=feaafiart1ev1aaatCvAUfKttLearuWrP9MDH5MBPbIqV92AaeXatLxBI9gBaebbnrfifHhDYfgasaacH8akY=wiFfYdH8Gipec8Eeeu0xXdbba9frFj0=OqFfea0dXdd9vqai=hGuQ8kuc9pgc9s8qqaq=dirpe0xb9q8qiLsFr0=vr0=vr0dc8meaabaqaciaacaGaaeqabaqabeGadaaakeaacqWGtbWucqGGOaakcqWGPbqAcqGGPaqkcqGH9aqpdaWcaaqaaiGbc2gaTjabcMgaPjabc6gaUnaaBaaaleaacqWGQbGAaeqaaOGaeiikaGIafmizaqMbaebadaWgaaWcbaGaemOqaieabeaakiabcIcaOiabdMgaPjabcYcaSiabdQgaQjabcMcaPiabcMcaPiabgkHiTiqbdsgaKzaaraWaaSbaaSqaaiabdEfaxbqabaGccqGGOaakcqWGPbqAcqGGPaqkaeaacyGGTbqBcqGGHbqycqGG4baEcqGGOaakcuWGKbazgaqeamaaBaaaleaacqWGxbWvaeqaaOGaeiikaGIaemyAaKMaeiykaKIaeiilaWIagiyBa0MaeiyAaKMaeiOBa42aaSbaaSqaaiabdQgaQbqabaGccqGGOaakcuWGKbazgaqeamaaBaaaleaacqWGcbGqaeqaaOGaeiikaGIaemyAaKMaeiilaWIaemOAaOMaeiykaKIaeiykaKcaaaaa@6349@

where d¯W(i)
 MathType@MTEF@5@5@+=feaafiart1ev1aaatCvAUfKttLearuWrP9MDH5MBPbIqV92AaeXatLxBI9gBaebbnrfifHhDYfgasaacH8akY=wiFfYdH8Gipec8Eeeu0xXdbba9frFj0=OqFfea0dXdd9vqai=hGuQ8kuc9pgc9s8qqaq=dirpe0xb9q8qiLsFr0=vr0=vr0dc8meaabaqaciaacaGaaeqabaqabeGadaaakeaacuWGKbazgaqeamaaBaaaleaacqWGxbWvaeqaaOGaeiikaGIaemyAaKMaeiykaKcaaa@328F@ is the average distance from the *i*-th point to the other points in its own cluster, and d¯B(i,j)
 MathType@MTEF@5@5@+=feaafiart1ev1aaatCvAUfKttLearuWrP9MDH5MBPbIqV92AaeXatLxBI9gBaebbnrfifHhDYfgasaacH8akY=wiFfYdH8Gipec8Eeeu0xXdbba9frFj0=OqFfea0dXdd9vqai=hGuQ8kuc9pgc9s8qqaq=dirpe0xb9q8qiLsFr0=vr0=vr0dc8meaabaqaciaacaGaaeqabaqabeGadaaakeaacuWGKbazgaqeamaaBaaaleaacqWGcbGqaeqaaOGaeiikaGIaemyAaKMaeiilaWIaemOAaOMaeiykaKcaaa@34A2@ is the average distance from the *i*-th point to points in another cluster *j*.

Each cluster of S-genes now forms one module. These modules are eventually further subdivided into smaller submodules until each submodule contains only 4 S-genes at most. This way we obtain a tree structure of modules, where each node (module) has children (submodules). We begin with estimating the leaves in the module tree. As each leaf module can contain 4 S-genes at maximum this can be done by enumerating all possible transitively closed network hypotheses and taking the highest scoring one. After the leaves in the module tree have been built, their connection is estimated. For this purpose we score all pairwise connections between any pair of S-genes from leaves *L*_1 _and *L*_2_. Denoting by |*L*_1_| and |*L*_2_| the number of S-genes in both leaves, these are 4. |*L*_1_|·|*L*_2_| tests altogether, because between any pair of S-genes (*n*_1_, *n*_2_) we can either have no edge, an edge from *n*_1 _to *n*_2_, an edge from *n*_2 _to *n*_1 _or an edge in both directions. After the best connection between *L*_1 _and *L*_2 _has been estimated, the corresponding subgraph is transitively closed. After all connections between leaves belonging to the same submodule in the module tree have been established, we recursively continue with connecting submodules in the same fashion as we did for leaf modules until the topology for the total network is completed.

### Generalized Inference Framework: Proof of Principle

To show the correctness of our generalized inference framework, we conducted experiments on the Drosophila dataset by Boutros *et al*. [I]. This dataset was also employed by Markowetz *et al*. [2] as a proof of principle with discretized data. The dataset consists of expression profiles from 16 Affymetrix microarrays: 4 genes (*tak, rel, key, mkk4/hep*) were stimulated by lipopolysaccharide (LPS) for 60 minutes and then knocked-down by RNAi with 2 replicates for each expression profile. Additionally there were 4 replicates of control experiments without LPS and RNAi and 4 replicates of expression profiling with LPS but without RNAi. The dataset is available in a preprocessed form as a supplement of [2].

**Figure 2:**
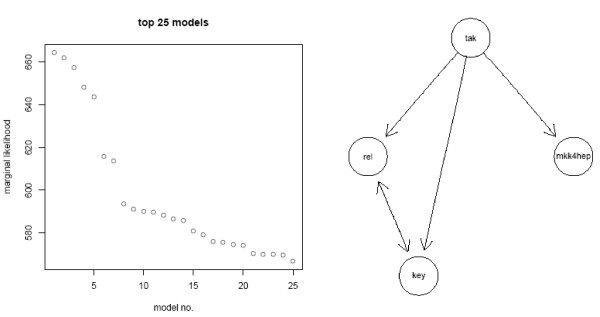
Scores of the top 25 models and the best model.

We took the same 68 genes showing a secondary effect (E-genes) as used in this publication and calculated *p*-values for differential gene expression between LPS stimulated and knock-down conditions by fitting an empirical Bayes model using the *limma *package in the *R *statistical computing environment [4]. We enumerated all possible 355 transitively closed network topologies and calculated their scores using (Eq. 7). The scores of the top 25 models and the best model are depicted in Figure 2. The score distribution of the 25 top models is slightly different, because of our modified inference scheme. We had a closer look at the best 4 models and found them to be identical to those shown in [2] (see also additional file 1). The second best model differs from the best model only in the missing edge *key *→ *rel*. The next two models are either missing the edge *tak → rel *or *tak → key*. The key feature is preserved in all of them: The signal runs through *tak *before splitting into two pathway branches, one containing *mkk4/hep*, the other both *key *and *rel*. This fits exactly to the findings of Boutros *et al*. [1].

### Large Scale Inference: Evaluation on Artificial Networks

To test our methods and to get better insights into the performance of our large scale inference methods, we generated data from artificial random networks. The sampling procedure for artificial networks is described in Section "Methods". We sampled networks with *n *= 4, 8,12 S-genes. For each number of S-genes we varied the number *m *= 4, 8,..., 4*n *of E-genes and the parameter *β *= 10, 50,100 describing the Beta(1, *β*) component of the *f*_1 _distribution (Eq. 10). We compared the SA approach with the module network. We evaluated both methods in terms of average sensitivity (i.e. ratio of correctly learned edges to total number of edges in the original network) and specificity (i.e. ratio of correctly unconnected genes to total number of unconnected genes in the original network) over 100 generated networks for each parameter combination (*n, m, β*). The initial temperature for the SA was set to 1000 and the maximum number of iterations to 100*n*. The initial network structure was always the graph with no edges. A logarithmic temperature cooling scheme according to [20] was used.

**Figure 3:**
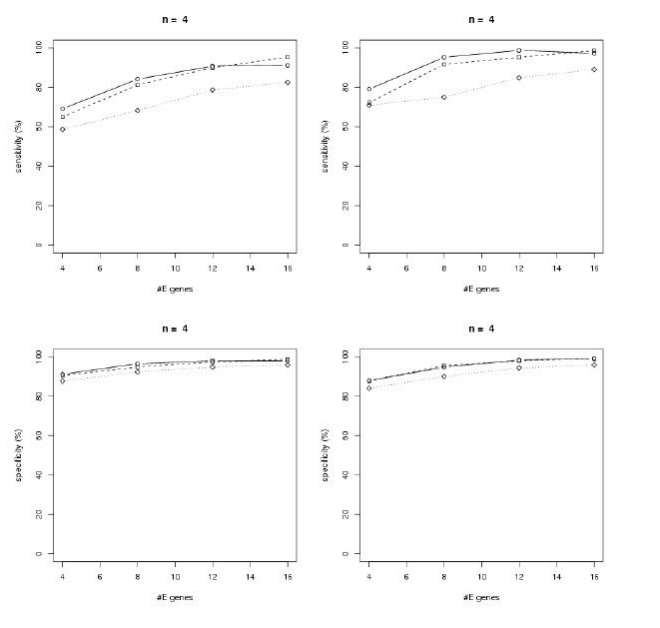
Sensitivity (top) and specificity (bottom) analysis for randomly generated networks with *n *= 4 S-genes: *β *= 100 (solid), *β *= 50 (dashed), *β *= 10 (dotted). Left: simulated annealing, right: module network.

The results are shown in Figure 3 – Figure 5. In general all methods achieve a higher specificity than sensitivity, which is due to our "*p*-value" sampling strategy, and they show a high robustness against a varying number of E-genes. All in all the module network approach shows a superiority to the SA approach, especially in terms of sensitivity. Using module networks the sensitivity and specificity for *n *= 4 goes up to almost 100%. For *n *= 8,12 the sensitivity lies around 80%, while the specificity reaches more than 90%. Moreover, for all tested values of *β *the curves are relatively close together. We also compared the computation times for both approaches and found the module network to be substantially faster for *n *= 8,12 (Figure 6). The average running time for network inference with *n *= 12 nodes was only 4s with the module network on our AMD dual core Opteron 2 GHz machine. In conclusion we think that the module network offers the most reliable and fast mechanism for large scale network inference among our tested approaches and is therefore taken as our inference method in the following section.

**Figure 4:**
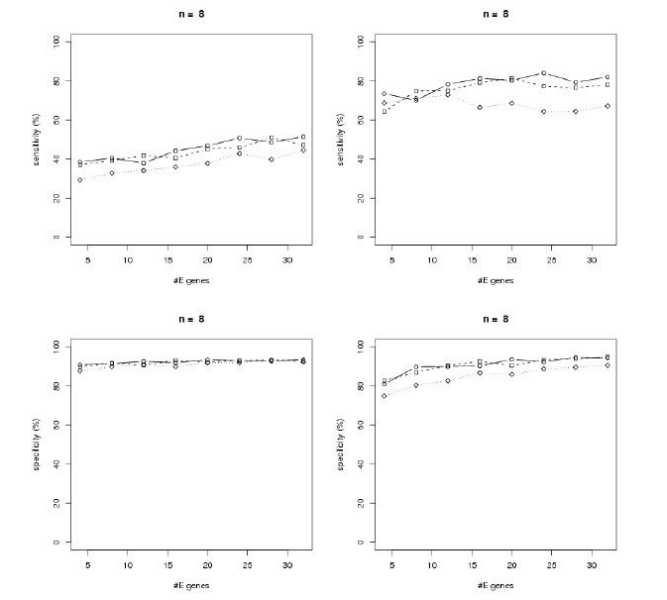
Sensitivity (top) and specificity (bottom) analysis for randomly generated networks with *n *= 8 S-genes: *β *= 100 (solid), *β *= 50 (dashed), *β *= 10 (dotted). Left: simulated annealing, right: module network.

**Figure 5:**
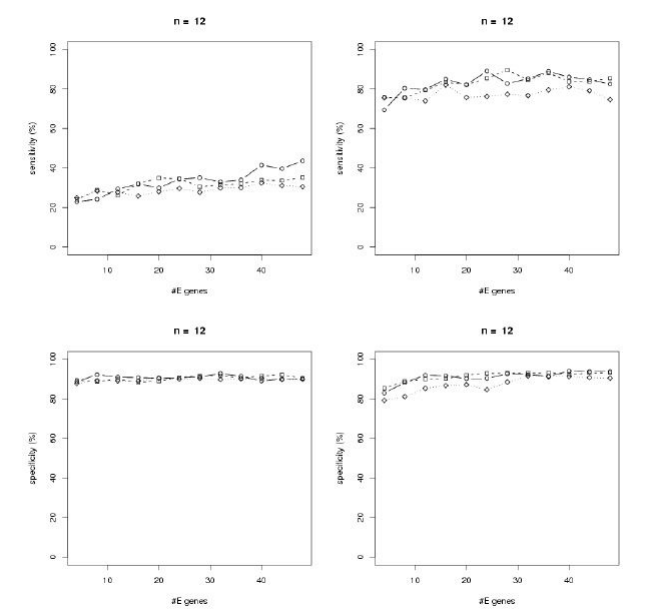
Sensitivity (top) and specificity (bottom) analysis for randomly generated networks with *n *= 12 S-genes: *β *= 100 (solid), *β *= 50 (dashed), *β *= 10 (dotted). Left: simulated annealing, right: module network.

**Figure 6:**
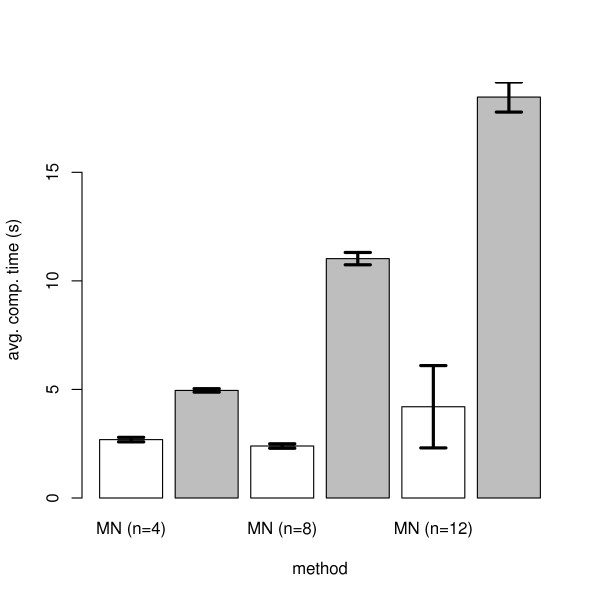
Computation times (s) for the module network (white) and the simulated annealing (gray) approach.

### Application to RNAi Data from Human ER-*α *pathway

We applied the module network to infer the complete topology for a network of 13 silenced genes (Table 1) in the ER-*α *pathway. The 13 genes were selected from previous microarray studies in our department to be influenced by ER status in breast cancer patients. Each of the 13 genes was silenced individually using two different siRNAs, and the effect on gene expression was studied on whole genome cDNA microarrays. The data were generated in our department, details on the data generation and preprocessing steps are described in Section "Methods".

**Table 1:** Differential genes in complete dataset and among E-genes: The first column shows the number of all genes with Benjamini-Hochberg false discovery rate [8] ≤ 10%; the second column the number of all genes with at least 1.5-fold disregulation; the third column the number of all genes with *f*_1_-density > 1 (effected genes). The last two columns show statistics among the selected E-genes (see Methods Section): the number of E-genes with false discovery rate ≤ 10% and with *f*_1_-density > 1

**Gene**	*fdr *≤ 10%	≥ 1.5-**fold disreg**.	*f*_1 _> 1	*fdr *≤ 10%(**E-genes**)	*f*_1 _> 1(**E-genes**)
AKT1	3	5	511	3	26
AKT2	2276	33	2278	63	63
BCL2	18	6	525	4	29
CCNG2	2904	10	2904	59	59
DDR1	0	2	217	0	23
ESR1	357	45	746	59	64
FOXA1	53	17	530	24	35
GDF15	3	2	298	3	26
GPR30	2	20	191	2	14
HSPB8	0	1	284	0	4
LOC120224	1141	3	1516	24	27
STC2	1447	18	1609	14	15
XPB1	3	4	642	3	46

**Figure 7:**
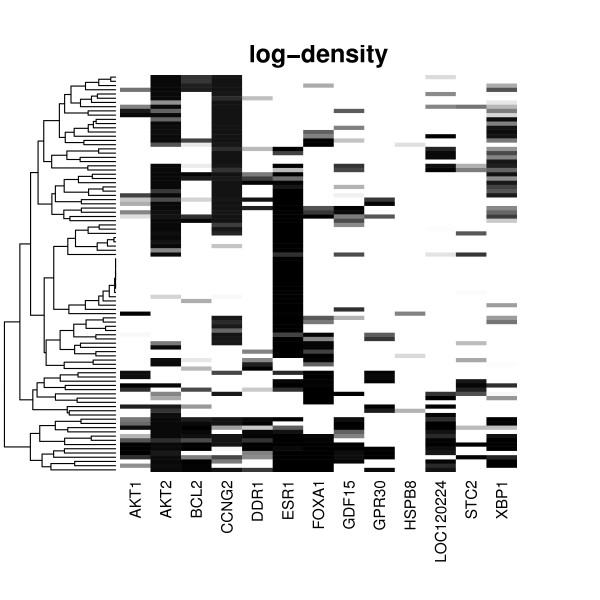
Interdepencencies of 13 genes in the ER-*α *pathway drawn as transitvely reduced graphs: a) literature knowledge (Ingenuity™), b) inferred without prior knowledge, c) inferred with prior knowledge on some E-gene – S-gene connections, d) inferred with additional knowledge from a). Figure b) – d) only show edges, which where found in more than 50% of all bootstrap sets. The corresponding fraction is reported at each edge.

We found several known interdependencies between E- and S-genes as well as among S-genes by an intensive literature screen. The corresponding information was obtained from the Ingenuity™ software and can be visualized in form of a interdependency graph (Figure 7a). It represents some prior knowledge, which can be used for the network inference with our module networks algorithm via the regularization technique (c.f. Section "Regularization").

We considered 3 situations for the network inference: 1. no prior knowledge (complete trust in data), 2. inclusion of known interdependencies between E- and S-genes (Table 2): For known interdependencies we set *P*(*θ*_*i *_= *j*|Φ) = 1 and* P*(*θ*_*i *_≠ *j*|Φ) = 0, while otherwise we have a uniform prior *P*(*θ*_*i *_= *j*|Φ) = 1n
 MathType@MTEF@5@5@+=feaafiart1ev1aaatCvAUfKttLearuWrP9MDH5MBPbIqV92AaeXatLxBI9gBaebbnrfifHhDYfgasaacH8akY=wiFfYdH8Gipec8Eeeu0xXdbba9frFj0=OqFfea0dXdd9vqai=hGuQ8kuc9pgc9s8qqaq=dirpe0xb9q8qiLsFr0=vr0=vr0dc8meaabaqaciaacaGaaeqabaqabeGadaaakeaadaWcaaqaaiabigdaXaqaaiabd6gaUbaaaaa@2F11@. In case of several interdependencies for one E-gene *P*(*θ*_*i *_= *j*|Φ) is rescaled appropriately. 3. additional inclusion of literature knowledge for interdependencies between S-genes with parameter *λ *chosen from the set {10^2^,10^1^,..., 10^-2^} via the AIC criterion (c.f. Section "Regularization"). The prior for the network structure  is chosen such that  = 1, if an interaction between S-gene *i *and S-gene *j *is known and  = 0.25 otherwise. That means missing a known interdependency is punished more than introducing an edge where nothing is known.

**Table 2:** Known interdependencies between S-genes and E-genes

**S-gene**	**E-gene**
*AKT*1	*ARL6IP, XBP*1
*AKT2*	*ARL6IP, XBP*1
*CCNG2*	*ARL6IP, XBP*1
*ESR*1	*ARL6IP, XBP*1

To ensure the statistical stability of the inferred network we employed bootstrapping: We sampled *m *E-genes from the total set of E-genes 50 times with replacement and each time ran the module network for topology induction. At the end we only considered edges, which were found in more than 50% of all bootstrap trials.

**Figure 8:**
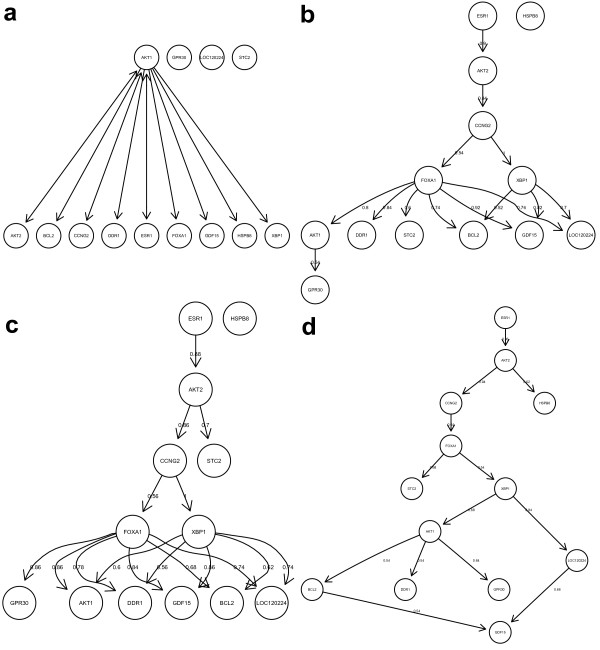
Heatmap showing the secondary effects of individual knock-downs (columns) on E-genes (rows) as log-*f*_1 _density (cutoff 0, darker = stronger effect). Our method tries to resolve the nested structure of these secondary effects.

Figure 7b–d) shows our obtained networks drawn as transitively reduced graphs for these three scenarios: As seen, a common motif in all three networks was the dependency cascade *ESR*1 → *AKT*2 → *CCNG*2 → *FOXA*1, which was found with high consistency and was also in agreement with the literature network (Figure 7a). A little bit more astonishing was the dependency of *AKT*1 from either *FOX A*1 or *XPB*1, which did, however, fit well to our data (c.f. Figure 8). The rest of the pathway reconstruction varied slightly among our three scenarios, but was in agreement with the data as well as with the literature.

## Conclusion

We proposed a method for reconstructing signaling pathways from secondary effects, which were observed on microarray after silencing genes of interest via RNAi. Our approach systematically extends and generalizes previous work by Markowetz et *al: *An inference scheme was developed, which can deal with *p*-values for differential gene expression and does not rely on discretized data only. Regularization was employed to incorporate prior assumptions on the network architecture into our framework. Finally, new algorithms for large scale inference of signaling pathways were developed and evaluated in a systematic fashion on artificially created data. Thereby, our module network, which recursively build up the complete topology from smaller pieces, revealed the best performance in terms of sensitivity and specificity. We used the module network to infer the signaling pathway for 13 genes in the ER-*α *pathway in human MCF-7 breast cancer cells and used a bootstrapping approach to ensure the statistical stability of the result. The induced edges in our inferred network were found with good consistency and could in many cases be also confirmed by the literature. Future biological experiments are planned to validate our reconstructed network in a systematic way. In conclusion of our results we think that our approach offers a scale-able, reliable and fairly general way for large scale inference of signaling pathways from secondary effects and therefore provides researchers with a valuable tool to gain insight into complex cellular processes.

The code for the module network inference method is available in the latest version of the -*R*-package *nem*, which can be obtained from the Bioconductor homepage (see additional file 3).

## Methods

### RNAi knockdown and microarray experiments

RNAi knock down experiments were conducted on 13 S-genes (Table 1), which were supposedly connected in signaling pathways in human MCF-7 breast cancer cells (ATCC, Manassas, VA). These cells were cultured in Gibco MEM medium with phenol red supplemented with 10% fetal bovine serum (FBS), 50 *μ*g/ml streptomycin, 50 U/ml penicillin, 1% MEM non essential amino acids (100×) and 100 *μ*g/ml insulin bovine (all reagents provided by Invitrogen).

Cells were split every 3–4 days to ensure exponential growth. MCF-7 cells were transiently transfected with at least two different chemically synthesized small interfering (si)RNA (50 nM) against one of the 13 genes in order to minimize off-target effects. Control silencing was done in the same experiment using control (non-silencing) siRNA (50 nM). All used siRNAs were provided by Qiagen (Hilden, Germany). Transfection was performed in antibiotic-free medium according to Qiagen HiPerFect standard transfection protocol. Therefore 1 × 10^4 ^cells/well were seeded onto a 96 well plate 24 h prior to transfection. After preincubation transfection was carried on for 42 h and total RNA was isolated using RNeasy mini prep Kit. Every knock-down experiment was performed in 2–4 independent replicates, and the mRNA level of each targeted gene was measured using qRT-PCR. Only experiments showing more than 70% silencing of the mRNA of interest were used for following studies. For global gene expression analysis 2 *μ*g of isolated total RNA was amplified using the Agilent Low RNA Input Fluorescent Linear Amplification Kit and hybridized in dye-swap design on at least 4 (according to number of used replicates) home made whole genome cDNA microarrys containing 37.500 genes from the RZPD Unigene 3.1 clone collection. The complete dataset was submitted to the GEO database (GEO ID: GSE7033). A manuscript describing the biological implication of the data and analysis in more detail is in preparation.

### Data Preprocessing

The microarray data was normalized on probe level using a variance stabilization transformation [21]. We calculated *p*-values for differential gene expression by fitting an empirical Bayes model using the *limma *package in the *R *statistical computing environment [4]. For each knock-down experiment we selected the top 100 ranked genes, which showed an at least 1.5-fold absolute change in expression level in at least one experiment. This gave us *m *= 94 E-genes altogether, which were the basis for our network inference (see additional file 2).

### Generation of Artificial Networks

To get better insights into the performance of our large scale inference methods way we generated data from artificial random networks. This was done as follows: A network topology was created by randomly connecting *n *signaling genes (S-genes) with *q *edges. The number *q *was itself a random number between 1 and 25% of all possible edges. It thus covered extremely sparse up to relatively dense topologies. No loops between a node and itself were allowed. After defining the core topology, the network was transitively closed. Because the Simulated Annealing (SA) method can only deal with Directed Acyclic Graphs (DAGs), in this case we additionally restricted ourselves to randomly generated transitively closed DAGs. After creating the network between S-genes, we attached *m *E-genes uniform randomly over all S-genes. We then simulated knock-downs of the individual S-genes. For the genes effected by the knock-down (E-genes) "*p*-values" from the distribution *f*_1 _were sampled (c.f. Subsection "Our Approach"). For those E-genes, where no effects were expected, the "*p*-values" were drawn uniform randomly from [0,1]. Afterwards, all sampled "*p*-values" were processed by the *f*_1 _density function.

### Implementation

All methods were implemented and computation and testing was performed using the statistical computing environment of *R*. The implementations of our methods have been integrated in the *R *package "Nested Effect Models" (*nem*) together with the original methods by Markowetz *et al*. [2]. The package and source code is publicly available via the Bioconductor repository.

## Authors' contributions

Algorithm development and computational analysis was performed by HF and TB. Biological experiments were carried out by MF and HS with substantial advise by AP. All authors have read and approved the final version of the manuscript.

## Supplementary Material

Additional file 1 top25solutionsBoutrosData. 25 highest scoring network structures for the data by Boutros *et al*. Click here for file

Additional file 2 Egenes. list of E-genes used for network inference for our data Click here for file

Additional file 3 nem_2.0.0. *R *package for nested effect models Click here for file

## References

[B1] Boutros M, Agaisse H, Perrimon N (2002). Sequential activation of signaling pathways during innate immune responses in *Drosophila*. Developmental Cell.

[B2] Markowetz F, Bloch J, Spang R (2005). Non-transcriptional pathway features reconstructed from secondary effects of RNA interference. Bioinformatics.

[B3] Tikhonov A, Arsenin V (1977). Solutions of ill-posed problems.

[B4] Smyth G (2004). Linear models and empirical Bayes methods for assessing differential expression in microarray experiments. Statistical Applications in Genetics and Molecular Biology.

[B5] Liao J, Lin Y, Selvanayagam Z, Shih W (2004). A mixture model for estimating the local false discovery rate in DNA microarray analysis. Bioinformatics.

[B6] Pounds S, Morris S (2003). Estimating the occurence of false positives and false negatives in microarray studies by approximating and partitioning the empirical distribution of p-values. Bioinformatics.

[B7] Efron B, Tibshirani R (2002). Empirical Bayes methods and false discovery rates for microarrays. Genetic Epidemiology.

[B8] Benjamini Y, Hochberg Y (1995). Controlling the False Discovery Rate: a Practical and Powerful Approach to Multiple Testing. J Royal Statist Soc.

[B9] Dempster A, Laird N, Rubin D (1977). Maximum likelihood from incomplete data via the EM algorithm. J Royal Statistical Soc Series B.

[B10] Fröhlich H, Fellmann M, Sültmann H, Poustka A, Beissbarth T (2007). Estimating Large Scale Signaling Networks through Nested Effects Models from Intervention Effects in Microarray Data. Proc German Conf on Bioinformatics.

[B11] Schölkopf B, Smola AJ (2002). Learning with Kernels.

[B12] Hastie T, Tibshirani R, Friedman J (2001). The Elements of Statistical Learning.

[B13] Kirkpatrick S, Gelatt CD, Vecchi MP (1983). Optimization by Simulated Annealing. Science.

[B14] Berg B (2004). Markov Chain Monte Carlo Simulations and Their Statistical Analysis.

[B15] Lukashin AV, Fuchs R (2001). Analysis of temporal gene expression profiles: clustering by simulated annealing and determining the optimal number of clusters. Bioinformatics.

[B16] Gonzalez OR, Kuper C, Jung K, Naval J, Prospero C, Mendoza E (2006). Parameter estimation using Simulated Annealing for S-system models of biochemical networks. Bioinformatics.

[B17] Poutre JL, van Leeuwen J (1987). Maintenance of Transitive Closures and Transitive Reductions of Graphs. Tech Rep RUU-CS-87-25, Rijksuniversiteit Utrecht.

[B18] Kaufman L, Rousseeuw P (1990). Finding Groups in Data: An Introduction to Cluster Analysis.

[B19] Rousseeuw P (1987). Silhouettes: a graphical aid to the interpretation and validation of cluster analysis. J Comp and Applied Mathematics.

[B20] Belisle CJP (1992). Convergence theorems for a class of simulated annealing algorithms. J Applied Probability.

[B21] Huber W, Heydebreck A, Sültmann H, Poustka A, Vingron M (2002). Variance Stabilization Applied to Microarray Data Calibration and to the Quantification of Differential Expression. Bioinformatics.

