# Supplementary material for: Large scale statistical inference of signaling pathways from RNAi and microarray data
Source: BMC Bioinformatics. 2007 Oct 15;8:386. doi: 10.1186/1471-2105-8-386 (PMC2241646; doi:10.1186/1471-2105-8-386)
Supplement: Additional file 1 — top25solutionsBoutrosData. 25 highest scoring network structures for the data by Boutros et al. [file 1471-2105-8-386-S1.gz › nem/inst/doc/markowetz-thesis-2006.tmp]

Probabilistic Models for Gene Silencing Data

Florian Markowetz

Dezember 2005

Dissertation zur Erlangung des Grades
eines Doktors der Naturwissenschaften (Dr. rer. nat.)
am Fachbereich Mathematik und Informatik
der Freien Universität Berlin

1. Referent: Prof. Dr. Martin Vingron
2. Referent: Prof. Dr. Klaus-Robert Müller

Tag der Promotion: 26. April 2006

Preface

Acknowledgements This work was carried out in the *Computational Diagnostics* group of the Department of Computational Molecular Biology at the Max Planck Institute for Molecular Genetics in Berlin. I thank all past and present colleagues for the good working atmosphere and the scientific—and sometimes maybe not so scientific—discussions.

Especially, I am grateful to my supervisor *Rainer Spang* for suggesting the topic, his scientific support, and the opportunity to write this thesis under his guidance. I thank *Michael Boutros* for providing the expression data and for introducing me to the world of RNAi when I visited his lab at the DKFZ in Heidelberg. I thank *Anja von Heydebreck*, *Jörg Schultz*, and *Martin Vingron* for their advice and counsel as members of my PhD commitee.

During the time I worked on this thesis, I enjoyed fruitful discussions with many people. In particular, I gratefully acknowledge *Jacques Bloch*, *Steffen Grossmann*, *Achim Tresch*, and *Chen-Hsiang Yeang* for their contributions. Special thanks go to *Viola Gesellchen*, *Britta Koch*, *Stefanie Scheid*, *Stefan Bentink*, *Stefan Haas*, *Dennis Kostka*, and *Stefan Röpcke*, who read drafts of this thesis and greatly improved it by their comments.

Publications Parts of this thesis have been published before. Chapter 2 grew out of lectures I gave in 2005 at the *Institute for Theoretical Physics and Mathematics* (IPM) in Tehran, Iran, and at the *German Conference on Bioinformatics* (GCB), Hamburg, Germany. I thank Prof. Mehrdad Shahshahani (Tehran) and Prof. Stefan Kurtz (Hamburg) for inviting me. Chapter 3 gathers results of two conference papers: the first one at the *Workshop on Distributed Statistical Computing* (DSC 2003) in Vienna, Austria [82], and the second one at the *Conference on Artifical Intelligence and Statistics* (AISTATS), in Barbados, 2005 [81]. Parts of chapter 4 were previously published in the journal *Bioinformatics* [80].

Figures This thesis reproduces three figures from other publications. I thank Prof. Danny Reinberg and Prof. Jules Hoffmann for the friendly permissions to reproduce Fig. 1.1 and Fig. 1.2, respectively. Fig. 1.3 is reproduced with permission from `www.ambion.com`.

Florian Markowetz Berlin, December 2005

Contents

Preface **i**

1 Introduction **1**
1.1 Signal transduction and gene regulation . . . 1
1.2 Gene silencing by RNA interference . . . 4
1.3 Thesis organization . . . 7

2 Statistical models of cellular networks **9**
2.1 Conditional independence models . . . 9
2.2 Bayesian networks . . . 14
2.3 Score based structure learning . . . 17
2.4 Benchmarking . . . 24
2.5 A roadmap to network reconstruction . . . 25

3 Inferring transcriptional regulatory networks **27**
3.1 Graphical models for interventional data . . . 27
3.2 Ideal interventions and mechanism changes . . . 29
3.3 Pushing interventions at single nodes . . . 32
3.4 Pushing in conditional Gaussian networks . . . 36

4 Inferring signal transduction pathways **45**
4.1 Non-transcriptional modules in signaling pathways . . . 45
4.2 Gene silencing with transcriptional phenotypes . . . 50
4.3 Accuracy and sample size requirements . . . 58
4.4 Application to *Drosophila* immune response . . . 60

5 Summary and outlook **65**

Bibliography **69**

Notation and Definitions **83**

Zusammenfassung **85**

Curriculum Vitae **87**

Chapter 1

Introduction

This thesis is concerned with signaling pathways leading to regulation of gene expression. I develop methodology to address two problems specific to gene silencing experiments: First, gene perturbation effects cannot be controlled deterministically and have to be modeled stochastically. Second, direct observations of intervention effects on other pathway components are often not available. This first chapter gives a concise background on gene regulation and cell signaling and explains the experimental technique of RNA interference (RNAi). Gene silencing by RNAi has drastically reduced the time required for genome-wide screens for gene function, but no work has been done so far to adapt statistical methodology to the specific needs of RNAi data.

1.1 Signal transduction and gene regulation

The success of genome sequencing projects has led to the identification of almost all the genes responsible for the biological complexity of several organisms. The next important task is to assign a function to each of these genes. Genes do not work in an isolated way. They are connected in highly structured networks of information flow through the cell. Inference of such cellular networks is the main topic of this thesis.

Eukaryotic cells Eukaryotes are organisms with cells containing nuclei, in which the genetic material is organized. Eukaryotes comprise multicellular animals, plants, and fungi as well as unicellular organisms. In contrast, *prokaryotes*, such as bacteria, lack nuclei and other complex cell structures. All cells have a membrane, which envelopes the cell and separates its interior from its environment. Inside the membrane, the salty *cytoplasm* takes up most of the cell volume. The most prominent structure inside the eukaryotic cell is the *nucleus* containing *DNA*, the carrier of genetic information. Deoxyribonucleic acid (DNA) is a *double-helix* formed by two anti-parallel complementary strands composed of the *nucleotides* adenine, guanine, cytosine, and thymine. The double-helix is packaged into a highly organized and compact nucleoprotein structure called *chromatin*. The fundamental dogma of molecular biology is that DNA produces *ribonucleic acid* (RNA) which in turn produces *proteins*. The functional units in the DNA that code for RNA or proteins are called *genes*. The

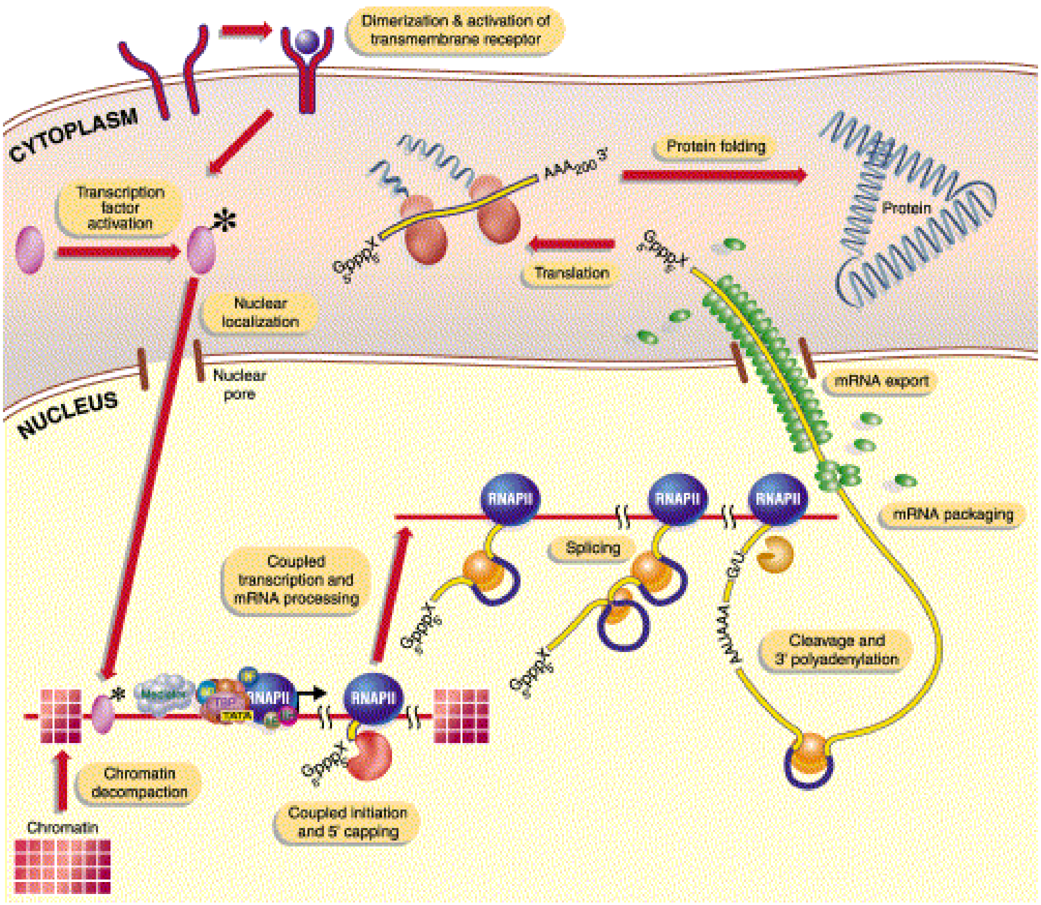

Figure 1.1: *Gene expression in a nutshell. A protein is produced in response to an external signal. See text for details. Reproduced from [94].*

DNA is the same in all cells, but the amount of gene products is not. The diversity of cell types and tissues in complex organisms like humans results from different genes being active.

Gene activity Gene expression is a highly regulated process by which a cell can answer to external signals and adapt to changes in the environment. Fig. 1.1 shows the basic principles of gene expression in eukaryotic cells. In the upper left part of the figure, a signal reaches the cell membrane and is recognized by a *transmembrane receptor*. Binding of a ligand to a receptor initiates an intracellular response. In this way receptors play a unique and important role in cellular communication and signal transduction. In our example, the signal activates a *transcription factor* protein in the cytoplasm. The activated transcription factor enters the cell nucleus and acts on the *promoter region* of a gene in the genome. The promoter region contains the information to turn the gene on or off. Depending on its function the bound transcription factor activates or inhibits gene expression. In the case of an activator, a process called *transcription* is started. A protein called *RNA polymerase II* (RNAP II) starts to copy the information contained in the gene into *messenger RNA* (mRNA).

The nuclear mRNA contains two kinds of regions: *exons*, which are exported from the nucleus as part of the mature mRNA, and *introns*, which are removed from the mature mRNA by a process called *splicing*. The spliced mRNA is transported from the nucleus into the cytoplasm. There it is *translated* into a protein poly-peptide sequence, which then folds into a three-dimensional protein structure.

Fig. 1.1 depicts the expression of a single gene and does not show the influence of other genes and proteins on the expression state. Regulation takes place at all levels, *e.g.*, in signal propagation, in transcription, in translation, and in protein degradation. At each single step many regulatory processes can concur. A transcription factor, for example, can be regulated transcriptionally and non-transcriptionally. Transcriptional regulation means control of the transcription factor mRNA level. Non-transcriptional regulation means controlling the activity level of the transcription factor protein by binding to a ligand, by dissociation of an inhibitor protein, by a protein modification like phosphorylation, or by cleavage of a larger precursor [71]. Of particular interest for this thesis are *transcriptional regulatory networks* and *signal transduction pathways*.

Transcriptional regulatory networks The process described in Fig. 1.1 can be iterated if the protein produced is again a transcription factor, which enters the nucleus and starts to activate or inhibit gene expression of other genes in the genome. Networks of transcription factors and their targets, which again could be transcription factors, are called *transcriptional regulatory networks* or *gene regulatory networks*. Reconstruction of regulatory networks is a prospering field in bioinformatics. This is mainly due to the availability of genome-wide measurements of gene-expression by microarrays, which provide a bird's eye view on gene activity in the cell and promise new insights into regulatory relationships [95, 118, 41].

Signal transduction pathways The second important process is indicated by a single arrow in the upper left corner of Fig. 1.1 leading from the receptor to the activation of a transcription factor. This arrow represents complex biochemical signal transduction pathways, which connect external signals to a transcriptional response. The main steps in signal propagation are protein interactions and modifications that do not act on a transcriptional level. We will explain essential parts of signaling pathways by the example of the *immune deficiency pathway* (Imd), which governs defense reactions against Gram-negative bacteria in *Drosophila melanogaster*. It is related to the mammalian tumor necrosis factor signaling pathways, as it uses structurally and functionally similar components [59]. The Imd pathway will play a central role in the application of the methodology developed in this thesis to a study of *Drosophila* immune response in chapter 4. Fig. 1.2 shows a schematic sketch of this pathway [111].

Immune induction of genes encoding antibacterial peptides like *Diptericins* relies on a transcription factor called Relish. In its inactive state Relish carries inhibitory sequences in the form of several ankyrin repeat domains. To activate Relish, it has to be phosphorylated and then cleaved from these inhibitory domains. Here we see a clear

difference to gene regulatory networks. Relish is not regulated on a transcriptional level, it just changes from an inactive into an active form, while the total amount of protein stays the same. This principle is often found in biology and ensures a quick response of the cell to a stimulus. Many pathway components mediating between the receptor at the cell membrane and activation of Relish are known. The phosphorylation of Relish before proteolytic cleavage is mediated by the IKK complex, which can directly phosphorylate Relish *in vitro*. TAK1 is a candidate for activation of the signalosome-equivalent IKKβ-IKKγ. IMD is a partner of an extensive receptor-adaptor complex, which detects infection by Gram-negative pathogens [111]. However, the precise roles of pathway components are often unknown and the object of intense research at present. Fig 1.2 also shows that signaling cascades form cycles and forks, and that different pathways may be connected by sharing components. Boutros *et al.* [12] found a fork in the signaling pathway below TAK1 leading to a Relish-independent response of cytoskeletal regulators via the JNK-pathway.

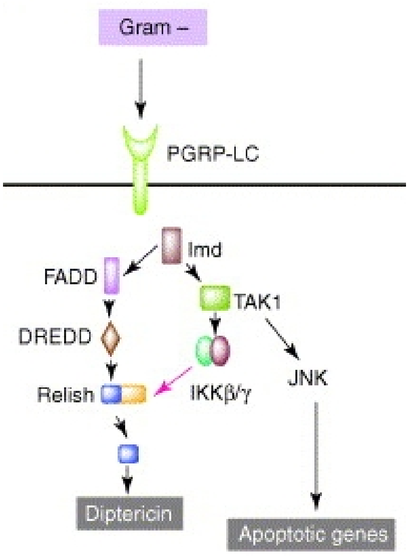

Figure 1.2: *The Imd pathway in Drosophila. Reproduced from [111]*

Cellular signaling pathways regulate essential processes in living cells. In many cases, alterations of these molecular mechanisms cause serious diseases including cancer. Understanding the organization of signaling pathways is hence a principal problem in modern biology. The next section describes RNA interference, which can be used in genome-wide screens to identify new pathway components and to order pathways in regulatory hierarchies.

1.2 Gene silencing by RNA interference

Physicist Richard Feynman once said: “What I cannot create, I do not understand”. This quote stresses the importance of action for understanding. A complex system is not understood solely by passive contemplation, it needs active manipulation by the researcher. In biology this fact is long known. Functional genomics has a long tradition of inferring the inner working of a cell—by breaking it. “What I cannot break, I do not understand” is the credo of functional genomics research.

Until recently external interventions have been labor intensive and time consuming. With methods making use of RNA interference (RNAi), this situation has changed. RNAi [38] is a cellular mechanism of post-transcriptional gene silencing. It is prominent in functional genomics research for two reasons. The first one is the physiological role it plays in gene regulation. The traditional role of RNA was a passive

intermediate in the translation of information from genes to proteins. Discovering its regulatory function is arguably one of the most important advances in molecular biology in decades. The second reason is that screens triggering RNAi of target genes can be applied on a genomic scale and allow rapid identification of genes contributing to cellular processes and pathways [19].

The RNAi mechanism RNAi is the disruption of a gene's expression by a double stranded RNA (dsRNA) in which one strand is complementary to a section of the gene's mRNA. It is described in detail in several recent reviews [85, 92, 15]. Fig. 1.3 gives an overview over the RNAi pathway. In an RNAi assay dsRNAs get introduced into the cell. In the cytoplasm they are processed by an enzyme of the Dicer family into small interfering RNAs (siRNAs). In mammals dsRNA molecules longer than 30 bp provoke interferon response, an antiviral defense mechanism, which results in the global shutdown of protein synthesis. RNAi can still be started by introducing siRNA molecules directly. Next, siRNA is assembled into an RNA-induced silencing complex (RISC). In fruitflies and mammals, the antisense strand is directly incorporated into RISC and activates it. In worms and plants the antisense strand might first be used in an amplification process, in which new long dsRNAs are synthesized, which are again cleaved by Dicer. Finally, antisense siRNA strands guide the RISCs to complementary RNA molecules, where they cleave and destroy the cognate RNA. This process leaves the genomic DNA intact but suppresses gene expression by RNA degradation.

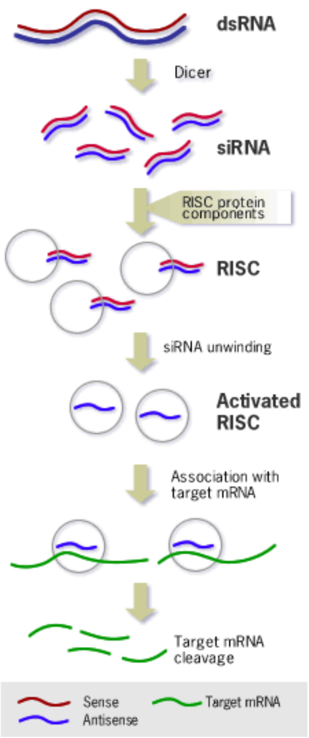

Figure 1.3: *The RNAi pathway. Reproduced from* `www.ambion.com`.

Bioinformatic challenges of RNAi RNA interference poses many challenges to research in computational biology. The first one is a better understanding of the RNAi mechanism by mathematical modeling and simulations [51]. Other challenges are specific to analyzing large-scale RNAi screens and include (i.) storage and preprocessing of data from RNAi experiments [113], (ii.) sequence analysis to identify unique siRNA targets and guard against off-target effects [91], and (iii.) ordering pathway components into regulatory hierarchies from phenotypic effects in RNAi silencing assays. This thesis contributes to the latter challenge. It proposes probabilistic models to infer pathway topologies from RNAi gene silencing data. Experimental techniques using the RNAi mechanism have drastically reduced the time required for testing downstream effects of gene silencing [19], but no work has been published so far to adapt statistical methodology to the specific needs of RNAi data. We will focus on two problems peculiar to RNAi. The first becomes apparent when comparing RNAi knockdowns to DNA knockouts, the second when deciding which phenotypes to observe.

Knockouts and knockdowns Genetic studies can be divided into forward or reverse screens [122]. In a *forward screen*, genes are mutated at random. To attribute a phenotype to a specific gene, the mutation must first be identified. This process is time-consuming and not easily applicable for all species. Additionally, some genes may always be missed by random sampling [19]. In contrast to random mutagenesis, *reverse screens* target specifically chosen genes for down regulation. This is what we will be concerned with in this thesis. The most direct way to silence a gene is by a gene knockout at the DNA level. Gene knockouts create animals or cell lines in which the target gene is non-functional [61]. It is difficult to interpret data from knockout mutants and to decide whether the phenotype is a direct effect of the non-functional gene or whether it is the result of the cell trying to compensate for the gene-loss. The danger of compensatory effects is less prominent for intervention techniques which allow faster down-regulation of target genes. In most cases, silencing genes by RNAi results in almost complete protein depletion after only a few days. Compared to gene knockouts, this makes silencing by RNAi more applicable in genome-wide screens and reduces compensatory effects at the same time. Two features make RNAi kockdowns "softer" than DNA knockouts. First, in an RNAi experiment the protein is not necessarily eliminated from the cells completely. A small amount of mRNA might escape degradation and protein can last a long time in the cell, if protein turnover is slow. This may mask or weaken phenotypes. On the other hand, this phenomenon may be useful in cases where a fully silenced gene would be lethal. Then the softer silencing by RNAi may still allow observations of phenotypes of the living cell. Second, even though transfection efficiency is typically high in RNAi experiments, transfection of cultured cells often results in a mixed population of cells, where some escape the RNAi effect. The observed phenotype is then an average over affected and not-affected cells.

In summary, all perturbation experiments *push* a gene's expression level towards a "no expression" state. Only in knockouts, however, the intervention leads to a completely non-functional gene. In RNAi experiments the gene is still active, but silenced. It is less active than normal due to human intervention. Hence, we do not fix the state of the gene, but push it towards lower activities. In addition this pushing is randomized to some extent: the experimentalist knows that he has silenced the gene, but in large-scale screens he cannot quantify the effect. This is the first problem approached in this thesis.

Phenotypic readout The term "phenotype" can refer to any morphologic, biochemical, physiological or behavioral characteristic of an organism. A number of phenotypes can be observed as results of perturbations [19]. Many genetic studies use *cell proliferation* versus *cell death* as a binary phenotype to screen for essential genes. Recently, large-scale identification of "synthetic lethal" phenotypes among nonessential genes, in which the combination of mutations in two genes causes cell death, provided a means for mapping genetic interactions [26]. To find genes essential for a pathway of interest, *reporter genes* or fluorescent markers are used to monitor activity of a signaling pathway [50]. Alternatively, visible phenotypes like *cell growth and viability* are screened for [13]. A global view of intervention effects

can be achieved by transcriptional phenotypes measured on microarrays. These can either be global time courses in development [31] or differential expression of single genes [61, 12]. Also other cellular features like activation or modification states of proteins could be used as phenotypes of interventions. What singles out the phenotypes described above is that they are accessible to large scale screens by high-throughput techniques.

Primary and secondary effects To describe the second problem tackled in this thesis, we need to distinguish between primary and secondary effects of interventions. We speak of a *primary effect*, if perturbing a pathway component results in an observable change at another pathway component. To achieve this change a complex machinery could have been involved. Thus, primary effects are not indicators of direct interactions between molecules. They are primary in the sense that they only involve pathway components and allow direct observations of information flow in the network. A primary effect can, *e.g.*, be observed in a transcriptional regulatory network when silencing a transcription factor leads to an expression change at its target genes. Unfortunately, in the case of signaling pathways primary effects will mostly not be visible in large-scale datasets. For example, when silencing a kinase we might not be able to observe changes in the activation states of other proteins involved in the pathway. The only information we may get is that genes downstream of the pathway show expression changes, or that cell proliferation or growth changed. Effects, which are not observable at other pathway components, but only as phenotypical features downstream the pathway, will be called *secondary effects*. Secondary effects provide only indirect information about information flow and pathway structure. Reconstructing features of signaling pathways from secondary effects is the second problem addressed in this thesis.

Why probabilistic models? There are several reasons to use probabilistic models for regulatory networks and signaling pathways. First of all, the measurement noise in todays experimental techniques is notoriously high. Second, gene perturbation experiments always entail uncertainty of experimental effects. The most important reason for probabilistic models comes from the biological system itself. Signal transduction, gene expression and its regulation are a stochastic processes [106, 110, 98]. There are two types of noise: *intrinsic* noise due to stochastic events during gene expression, and *extrensic* noise due to cellular heterogeneity [106]. Intrinsic noise is responsible for differences between identical reporters in *the same* cell, and extrensic noise for differences between identical reporters in *different* cells. Probabilistic models take care of all these kinds of noise.

1.3 Thesis organization

In summary, there are two problems to be addressed when modelling data from RNAi experiments. First, how to account for the uncertainty of intervention effects in a noisy environment. Second, how to infer signaling pathways if direct observations of

gene silencing effects on other network components may not be visible in the data. This thesis proposes novel methodology to address both questions. It is organized as follows.

Statistical models of cellular networks Chapter 2 gives an overview of recent approaches to visualize the dependency structure between genes. Even though reverse engineering is a fast developing area of research, the methods used can be organized by a few basic concepts. Statistical network methods encode statements of *conditional independence*: can the correlation observed between two genes be attributed to other genes in the model? Methods implementing this idea include graphical Gaussian models and Bayesian networks. Bayesian networks are the most powerful and flexible statistical model encoding the highest resolution of dependency structure. The methodology described here will be the basis for building models for interventional data in the following chapters.

Inferring transcriptional regulatory networks In chapter 3, we develop a theory of learning from gene perturbations in the framework of conditional Gaussian networks. The basic assumption is that effects of silencing genes in the model can be observed at other genes in the model. To model the uncertainty involved in real biological experiments, perturbations are modelled stochastically—and not deterministically as in classical theory. This answers the first question raised by RNAi data.

Inferring signal transduction pathways The methods described so far elucidate the dependence structure between observed mRNA quantities. Chapter 4 goes one step further. It shows that expression data from perturbation experiments allows to infer even features of signaling pathways acting by non-transcriptional control. The signaling pathway is reconstructed from indirect observations. This answers the second question raised by RNAi data. The proposed algorithm reconstructs pathway features from the nested structure of affected downstream genes. Pathway features are encoded as silencing schemes. They contain all information to predict a cell's behaviour to an external intervention. Simulation studies confirm small sample size requirements and high accuracy. Limits of pathway reconstruction only result from the information content of indirect observations. The practical use is exemplified by analyzing an RNAi data set investigating the response to microbial challenge in *Drosophila melanogaster*.

Chapter 2

Statistical models of cellular networks

In this chapter I describe statistical models to visualize the correlations structure of genes. The methods can be distinguished by how deeply they purge influences of other genes from the observed correlations (section 2.1). The most prominent models are Bayesian networks (section 2.2). To learn them from data I discuss score based approaches in section 2.3. Section 2.4 reviews benchmarking of models and section 2.5 shows how my own approaches developed in the following chapters relate to recent developments in literature.

2.1 Conditional independence models

Let a set V of p network components be given. In probabilistic models we treat each component $v \in V$ as a random variable X_v and the set of all components in the model as a random vector $\mathbf{X} = (X_1, \ldots, X_p)$. The dataset M consists of N measurements, that is, realizations $\mathbf{x}^1, \ldots, \mathbf{x}^N$ of the random vector $\mathbf{X}$. We think of it as a $p \times N$ matrix with genes corresponding to rows and measurements to columns.

Network components are identified with nodes in a graph. The goal will be to find an edge set $\mathcal{E}$ representing the dependency structure of the network components. We will call the graph $T = (V, \mathcal{E})$ the topology of the cellular network. Depending on the model, T can be directed or undirected, cyclic or acyclic. In the important special case, where T is a directed acyclic graph (DAG), we call it D. The biological meaning of a "network component" depends on what kind of data we analyze. Most of the time it will be microarray data and the network is a transcriptional gene regulatory network. So, we will mostly speak of network components as genes. But the same methods can also be applied to protein data, even though only few examples can be found in literature [148, 68, 114].

2.1.1 Coexpression networks

Biological processes result from concerted action of interacting molecules. On this general observation builds a simple idea, which underlies the first approaches to cluster expression profiles [37, 126] and is still widely used in functional genomics. It is called the *guilt-by-association heuristic*: if two genes show similar expression profiles, they are supposed to follow the same regulatory regime. To put it more pointedly: coexpression hints at coregulation. Coexpression networks are constructed by computing a similarity score for each pair of genes. If similarity is above a certain threshold, the gene pair gets connected in the graph, if not, it remains unconnected. Wolfe *et al.* [147] argue that networks of coexpressed genes provide a widely applicable framework for assigning gene function. They show that coexpression agrees well with functional similarity as it is encoded in the Gene Ontology [5].

Building coexpression networks The first critical point in building a coexpression network is how to formalize the notion of similarity of expression profiles. Several measures have been proposed. The most simple similarity measure is correlation. In a Gaussian model, zero correlation corresponds to statistical independence. Correlation networks are easy to interpret and can be accurately estimated even if $p \gg N$, that is, the number of genes is much larger than the number of samples. Stuart *et al.* [133] build a graph from coexpression across multiple organisms (humans, flies, worms and yeast). They find many coexpression relationships to be conserved over evolution. This implies a selective advantage and thus functional relationship between these gene-pairs. Bickel [10] generalizes correlation networks to time series data by introducing a time-lag for correlation.

Correlation is a linear measure of independence, non-linear dependencies between genes are not necessarily found. This problem can be avoided using networks built from pair-wise mutual information [18]. Another flexible similarity measure are kernel-functions [116], which are extensively used in wide parts of Machine Learning. Yamanishi *et al.* [148] use kernel functions for supervised network reconstruction. They show that the kernel formalism gives a unified framework for integrating different types of data including expression profiles and protein-interaction graphs. Then, they tune kernel parameters in known parts of a protein-interaction graph and use them to infer unknown parts. Kato *et al.* [68] weight the different data sources according to noise and information content when combining them in the kernel.

When comparing different types of tissues, e.g., healthy cells versus tumor cells, it may be interesting to find genes highly correlated under one condition, but losing this correlation under the second condition. Kostka and Spang [70] call this behaviour *differential coexpression* and interpret it as gain or loss of a regulatory mechanism. They introduce a correlation-based method to identify sets of differentially coexpressed genes.

The second critical point is how to assess significance of results. Many pairs of genes will show similar behaviour in expression profiles by chance even though they are

not biologically related. A practical, though time-consuming strategy consists in permuting the data matrix and comparing the network obtained on real data with the distribution of similarity scores achieved in the permutations. Bickel [10] uses permutations to estimate the false discovery rate of spurious connections. In the supervised setting of Yamanishi *et al.* [148] cross-validation can be applied to choose optimal parameters.

Problems of coexpression based approaches Fig. 2.1 shows several reasons, why three genes X, Y and Z can be found to be coexpressed. We cannot distinguish direct from indirect dependencies by just looking at similar expression patterns. High similarity of expression tells us little about the underlying biological mechanisms.

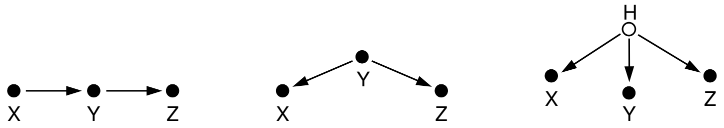

Figure 2.1: *Three reasons, why X, Y, and Z are coexpressed. They could be regulated in a cascade (left), or one regulates both others (middle), or there is a common "hidden" regulator (right), which is not part of the model.*

There are two possible solutions. Functional genomics has a long tradition of perturbing the natural state of a cell to infer gene-function from the observed effects. Interventions allow to decide between the three models in Fig. 2.1, because each one results in different predictions of effects, which can be compared to those obtained in experiments. Statisticians devised a different cure. Statistical methods search for correlations which cannot be explained by other variables. The theoretical background is the notion of *conditional independence*. Statistical methods filter out correlations, which can be attributed to other genes.

Conditional independence Conditional independence is defined as follows: Let X, Y, Z be random variables with joint distribution P. We say that X *is conditionally independent of* Y *given* Z (and write $X \perp Y \mid Z$) if and only if

$$P(X = x, Y = y \mid Z = z) \; = \; P(X = x \mid Z = z) \cdot P(Y = y \mid Z = z) \qquad (2.1)$$

This is the same as saying

$$P(X = x \mid Y = y, Z = z) \; = \; P(X = x \mid Z = z)$$

and is a direct generalization of the independence condition for X and Y, namely,

$$P(X = x, Y = y) \; = \; P(X = x) \cdot P(Y = y).$$

The same definitions hold if conditioning is not on a single variable Z but on a set of variables $\mathbf{Z}$. For an interpretation, we can think of random variables as abstract pieces of knowledge obtained from, say, reading books [72]. Then $X \perp Y \mid Z$ means:

"Knowing Z, reading Y is irrelevant for reading X"; or in other words: "If I already know Z, then Y offers me no new information to understand X." Variable Z can explain the correlation between X and Y.

The statistical models we discuss in the following all build on conditional independence. To decide on an edge between X and Y in the graph, they ask questions of the form "Is X independent of Y given $\mathbf{Z}$?", but differ with respect to what $\mathbf{Z}$ stands for: either all other variables except for X and Y, or single third variables, or any subset of all the other variables. Coexpression networks can be seen as the special case $\mathbf{Z} = \emptyset$, which encodes marginal dependencies.

2.1.2 Full conditional models

Full conditional models ask: "Can the correlation observed between two genes be explained by *all other genes* in the model?" Nodes i and j are connected by an edge if and only if

$$X_i \not\perp X_j \mid \mathbf{X}_{\text{rest}}. \tag{2.2}$$

where "rest" denotes the set of all variables in V without i and j. Full conditional models become especially simple in a Gaussian setting. Assume that $\mathbf{X} \sim \mathrm{N}(\mu, \Sigma)$, where Σ is invertible. Let $K = \Sigma^{-1}$ be the *concentration matrix* of the distribution (also called the *precision matrix*). The value $-k_{ij}/\sqrt{k_{ii}k_{jj}}$ is called the *partial correlation coefficient* between genes i and j [72]. Then, it holds for $i, j \in V$ with $i \neq j$ that

$$X_i \perp X_j \mid \mathbf{X}_{\text{rest}} \quad \Leftrightarrow \quad k_{ij} = 0. \tag{2.3}$$

This relation is used to define Gaussian graphical models (GGMs) [72, 35]. A GGM is an undirected graph on vertex set V. To each vertex $i \in V$ corresponds a random variable $X_i \in \mathbf{X}$. The edge set of a GGM is defined by vanishing partial correlations. Vertices i and j are adjacent if and only if $k_{ij} \neq 0$. An example is shown in Fig. 2.2.

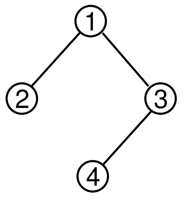

Figure 2.2: *Example of a full conditional model. Missing edges between nodes indicate independencies of the form* $X_i \perp X_j \mid \mathbf{X}_{rest}$. *We can read from the graph that* $X_1 \perp X_4 \mid \{X_2, X_3\}$ *and* $X_2 \perp X_3 \mid \{X_1, X_4\}$ *and* $X_2 \perp X_4 \mid \{X_1, X_3\}$.

The estimation of a GGM from data is a three-step process. First estimate the covariance matrix Σ, *e.g.*, by the sample covariance matrix $\hat{\Sigma} = \frac{1}{N-1}(M - \bar{M})(M - \bar{M})^T$, where $\bar{M}$ denotes the sample mean. Then, invert $\hat{\Sigma}$ to obtain an estimate $\hat{K}$ of the precision matrix K. Finally, employ statistical tests [72, 124, 33, 32] to decide, which entries in $\hat{K}$ are significantly different from zero.

Comparison to correlation networks Correlation graphs visualize the structure encoded in the correlation matrix Σ, which tells us about the similarity of expression

profiles. In GGMs, we model via the precision matrix $K = \Sigma^{-1}$, which tells us, how much correlation remains after we corrected for the influence of all other genes. GGMs not only filter out high correlations, which can be attributed to other genes, but may also draw attention to genes which are only very weakly correlated with a gene of interest, but highly related in terms of partial correlations in the context of the other neighboring genes in the GGM. These genes can be overlooked in correlation networks [30, 84].

GGMs have another clear advantage over correlation networks. Directly or indirectly, almost all genes will be correlated. Thus, the *correlation coefficient* is a weak criterion for dependence, but zero correlation is a strong indicator for independence. On the other hand, *partial correlation coefficients* usually vanish. They provide a strong measure of dependence and, correspondingly, only a weak criterion of independence [115].

Problems of GGMs Full conditional relationships can only be accurately estimated if the number of samples N is relatively large compared to the number of variables p. If the number of genes to be analyzed exceeds the number of distinct expression measurements (that is, if $p \gg N$), the correlation matrix of expression profiles between genes does not have full rank and cannot be inverted [115]. The $p \gg N$-situation is true for almost all genomic applications of graphical models. There are basically two ways out: either improve the estimators of partial correlations or resort to a simpler model. The basic idea in all of these approaches is that biological data are high-dimensional but *sparse*, in the sense that only a small number of genes will regulate one specific gene of interest. We end this section with examples of improved estimators and describe more strongly regularized models in the following section.

Several papers suggest ways to estimate GGMs in a $p \gg N$-situation. Kishino and Waddell [69] propose gene selection by setting very low partial correlation coefficents to zero. As they state, the estimate still remains unstable. Schäfer and Strimmer [115] improve all three steps of GGM construction. First they sample with replacement from the dataset to obtain many bootstrap [36] samples. Then, they estimate Σ by the mean covariance matrix achieved over all bootstrap replicates. Instead of the usual matrix inverse, they use the Moore-Penrose pseudoinverse, which is based on a singular value decomposition of $\hat{\Sigma}$ and can be applied also to singular matrices. Finally, they use false discovery rate multiple testing for the selection of edges to be included in the GGM.

2.1.3 First order conditional independence

First order conditional independence models ask: "Can the correlation between two genes be explained by a single third gene?" In contrast to GGMs, first order conditional independence models condition not on the whole rest, but only on single third

genes. Draw an edge between vertices i and j $(i \neq j)$ if and only if the correlation coefficient $\rho_{ij} \neq 0$ and no third variable can explain the correlation:

$$X_i \not\perp X_j \mid X_k \quad \text{for all } k \in V \setminus \{i, j\}, \tag{2.4}$$

This general idea can be implemented in different ways: Basso *et al.* [7] build a model based on conditional mutual information. The resulting method is called ARACNe and was successfully applied to expression profiles of human B cells. In a Gaussian setting, first order conditional independence models were proposed by several authors [144, 145, 79, 27]. Testing for first order conditional independence involves only triples of genes at a time. Thus, the problem for GGMs in high dimensions no longer exists. Wille and Bühlmann [144] prove: if the full conditional independence graph (the GGM) contains no cycles, then the first order conditional independence graph coincides with the full conditional independence graph. Wille *et al.* [145] use sparse Gaussian graphical modelling to identify modules of closely related genes and candidate genes for cross-talk between pathways in the Isoprenoid gene network in *Arabidopsis thaliana*.

2.2 Bayesian networks

In the last sections we have seen methods to build graphs from

$$\begin{aligned} \text{marginal dependencies} \quad & X_i \not\perp X_j, \\ \text{full conditional dependencies} \quad & X_i \not\perp X_j \mid \mathbf{X}_{\text{rest}}, \\ \text{first order dependencies} \quad & X_i \not\perp X_j \mid X_k \quad \text{for all } k \in \text{rest}. \end{aligned}$$

The logcial next step is to ask for independencies *of all orders*. In the resulting graph, two vertices i and j are connected if *no subset* of the other variables can explain the correlation, that is, if

$$X_i \not\perp X_j \mid \mathbf{X}_S \quad \text{for all } S \subseteq V \setminus \{i, j\}. \tag{2.5}$$

This includes testing marginal, first order and full conditional independencies. Thus, the number of edges will be less compared to the models in the previous sections. The graph encoding independence statements of the form (2.5) for all pairs of nodes is still undirected. It can be shown that knowing independences of all orders gives a more advanced picture of correlation structure. The collection of independence statements already implies directions of some of the edges in the graph [96, 97, 127]. The resulting directed probabilistic model is called a *Bayesian network*.

Definition A (static) Bayesian network is a graphical representation of the dependency structure between the components of a random vector $\mathbf{X}$. The individual random variables are associated with the vertices of a directed acyclic graph (DAG) D, which describes the dependency structure. Each node is descibed by a local probability distribution (LPD) and the joint distribution $p(\mathbf{x})$ over all nodes factors as

$$p(\mathbf{x}) = \prod_{v \in V} p(x_v \mid \mathbf{x}_{pa(v)}, \theta_v), \tag{2.6}$$

where θ_v denotes the parametrization of the local distribution. The DAG structure implies an ordering of the variables. The parents of each node are those varibles that render it independent of all other predecessors. The factorization of the joint distribution in Eq. 2.6 is the key property of Bayesian networks. It allows to segment the set of variables into families, which can be treated individually. This basic definition of Bayesian networks poses a number of further questions, which will be answered in the following:

1. How do the local probability distributions $p(x_v \mid \mathbf{x}_{pa(v)}, \theta_v)$ look like?
2. How is conditional independence defined for DAGs?
3. How can we learn a Bayesian network structure from data?
4. Are there natural limits to structure learning?

Local probability distributions (LPDs) Bayesian network models differ with respect to assumptions on the local probability distributions $p(x_v|\mathbf{x}_{pa(v)}, \theta_v)$ attached to each node $v \in V$. Basically, there are two types of parametric LPDs used in practice: multinomial distributions for discrete nodes and Gaussian distributions (normal distributions) for continuous nodes. The general model in statistics is a mixture of a discrete and a continuous part. Additionally, there are approaches to use non-parametric regression models linking parents to children. In the following, we will shortly introduce each of these models.

- *Discrete LPDs.* A discrete node v with discrete parents $pa(v)$ follows a multinomial distribution:
$$X_v \mid \mathbf{x}_{pa(v)}, \theta_v \;\sim\; \text{Multin}(1, \theta_{v|\mathbf{x}_{pa(v)}}) \tag{2.7}$$
It is parametrized by a set of probability vectors $\theta_v = \{\theta_{v|\mathbf{x}_{pa(v)}}\}$, one for each configuration $\mathbf{x}_{pa(v)}$ of parents of v.
- *Gaussian LPDs.* A continuous node v with continuous parents $pa(v)$ follows a normal distribution:
$$X_v \mid \mathbf{x}_{pa(v)}, \theta_v \;\sim\; \text{N}(\mu_v, \sigma_v^2), \tag{2.8}$$
where the mean $\mu_v = \beta_v^{(0)} + \sum_{i \in pa(v)} \beta_v^{(i)} x_i$ is a linear combination of parent states. The normal distribution is parametrized by a vector $\theta_v = (\beta_v, \sigma_v^2)$ containing regression coefficients $\beta_v = (\beta_v^{(i)})_{i \in pa(v)}$ for each parent node and a variance for X_v.
- *Conditional Gaussian (CG) networks.* CG networks are a combination of discrete and Gaussian networks. Continuous nodes follow a Gaussian distribution and are allowed discrete and continuous parents, while discrete nodes follow a multinomial distribution and are restricted to discrete parents. Thus, the network can be divided into a completely discrete part and a mixed part containing discrete and continuous nodes. CG networks constitute the general class of graphical models studied in statistics [72].
- *Regression trees.* Segal *et al.* [119, 120] use regression trees as LPDs. These capture the local structure in the data [42, 21], whereas the DAG describes the global structure. Each regression tree is a rooted binary tree with parents in the DAG as

internal nodes. Each leaf node of the tree is associated to a univariate Gaussian distribution.

- *Non-parametric regression.* Instead of the parametric approaches discussed so far, the relationship between parents and children in the DAG can also be modeled by non-parametric regression models [64, 65, 66, 134]. The result is a non-linear continuous model. This is an advantage over multinomial or Gaussian Bayesian networks, which are either discrete or linear.
- *Boolean logic LPDs.* Bulashevska and Eils [16] constrain LPDs to noisy logic functions like OR, AND for activatory parent-child relations or NOR, NAND for inhibitory. This has the advantage of simplifying and regularizing the model, while at the same time making it easier to interpret.
- *Kinetic modeling.* Nachman *et al.* [89] use non-linear Michaelis-Mentens dynamics to model how the transcription rate of a gene depends on its regulators. This approach combines Bayesian networks with a biochemically realistic quantitative model of gene regulation.

Conditional independence in directed graphs In Fig. 2.2 we saw how to read off independence statements from a full conditional independence graph. How does this work in the case of Bayesian networks? The answer is given by the definition of *d-separation* [97] ("d" for directed). A path q in a DAG D is said to be d-separated (or blocked) by a set of nodes $\mathbf{S}$ if and only if at least one of the following two conditions holds:

1. q contains a chain $i \to m \to j$ or a fork $i \leftarrow m \to j$ such that the middle node m is in $\mathbf{S}$, or
2. q contains an inverted fork (or collider) $i \to m \leftarrow j$ such that the middle node m is **not** in $\mathbf{S}$ and such that no descendent of m is in $\mathbf{S}$.

If all paths between i and j are blocked by $\mathbf{S}$ then (and only then) holds $X_i \perp X_j \mid X_{\mathbf{S}}$. The three archetypical situations can be seen in Fig. 2.3. The definition of d-separation, also called the *Global Markov condition*, allows to read statements of statistical indepence off the DAG structure.

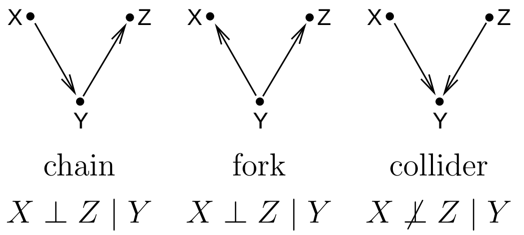

Figure 2.3: *The three archetypical situations in the definition of d-separation. In the chain and the fork, conditioning on the middle node makes the others independent. In a collider, X and Z are marginally independent, but get dependent once Y is known.*

Markov equivalence Many Bayesian networks may represent the same statements of conditional independence. They are statistically undistinguishable and we call them *Markov equivalent*. All equivalent networks share the same underlying undirect graph (called the *skeleton*) but may differ in the direction of edges, which are not

part of a *v-structure*, that is, a child with unmarried parents (same as a collider in Fig. 2.3). This was shown by Verma and Pearl [139]. It poses a theoretical limit on structure learning from data: even with infinitely many samples, we cannot resolve the structures in an equivalence class.

Acyclicity in a cyclic world Bayesian networks allow the highest resolution of correlation structure. Still, they suffer from a severe shortcoming: they are acyclic. With cycles, we cannot decompose the joint distribution as in Eq. 2.6. Biological networks are all known to contain feedback loops and cycles [4]. Modeling the cell cycle with an acyclic model [44] may not be the best idea. Fortunately, the cycle problem can be solved by assuming that the system evolves over time. This is shown in Fig. 2.4. We no longer model a static random vector $\mathbf{X}$ but a time series $\mathbf{X}[1], \ldots, \mathbf{X}[T]$ of observing $\mathbf{X}$ at T timepoints. If we assume that X_v at time $t+1$ can only have parents at time t, then cycles "unroll" and the resulting model is again acyclic and tractable: it is called a *Dynamic Bayesian network* (DBN) [45, 87]. DBNs found many applications in computational biology [154, 9, 157]. They are often combined with hidden variables [101], which can also capture non-transcriptional effects [8, 104, 105, 89, 93].

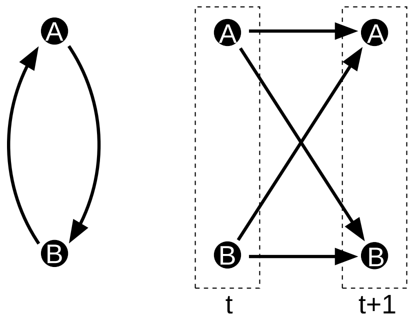

Figure 2.4: *The cycle unrolls into an acyclic graph over different time slices.*

2.3 Score based structure learning

In correlation networks, GGMs and sparse GGMs we use statistical tests for each gene pair to decide whether the data support an edge or not. The number of tests to be done in these models is limited, even though it can be big in the case of sparse GGMs. For Bayesian networks we would have to test independence of a gene pair for every subset of the other genes. This is called *constraint-based* learning of Bayesian networks. The examples discussed in [97, 127] involve only a handful of variables. For bigger problems testing gets infeasible very quickly. In applications in computational biology the network structure is thus mostly estimated by score based techniques.

2.3.1 Maximum likelihood scores

Maximum likelihood A straight-forward idea for model selection is to choose the DAG D, which allows the best fit to data M. This means maximizing the likelihood $p(M|D,\theta)$ as a function of θ. A score for DAG D is then given by

$$\text{score}_{ML}(D) \;=\; \max_{\theta}\; p(M|D,\theta) \qquad (2.9)$$

Unfortunately, the likelihood is not an appropriate score to decide between models since it tends to overfitting. Richer models with more edges will provably have better likelihood than simpler ones. A standard solution to this problem is to penalize the maximum likelihood score according to model complexity. An often used example of this general strategy is scoring with the Bayesian information criterion.

Bayesian information criterion (BIC) Contrary to what the name suggests, the BIC score [117] is not a Bayesian score. It is a regularized maximum likelihood estimate, which penalizes the maximal likelihood of the model with respect to the number of model parameters to control overfitting. It is defined as

$$\text{score}_{BIC}(D) \;=\; \max_{\theta}\; p(M|D,\theta) - \frac{d}{2}\log N, \tag{2.10}$$

where d is the number of parameters. The BIC score can also be used to learn Bayesian networks with missing values or hidden variables. The likelihood has then to be maximized via the Expectation-Maximization (EM) algorithm. In such a scenario, the BIC score was used by Nachman *et al.* [89] to learn kinetic models of transcription factors and their targets. They treated protein activities and kinetic constants as hidden variables. In cases, where the likelihood is accessible to conjugate analysis, a full Bayesian approach is preferred over ML or BIC.

2.3.2 Bayesian scores

In Bayesian structure learning we evaluate the posterior probability of model topology D given data M:

$$\text{score}_{Bayes} \;=\; p(D|M) \;=\; \frac{p(M|D)\cdot p(D)}{p(M)} \tag{2.11}$$

The term $p(M)$ is an average of data likelihoods over all possible models. We do not need to compute it for relative model scoring. The term $p(D)$ is a prior over model structures. The main term is the marginal likelihood $p(M|D)$, which equals the full model likelihood averaged over parameters of local probability distributions, that is,

$$p(M|D) = \int_{\Theta} p(M|D,\theta)p(\theta|D)\; \mathrm{d}\Theta. \tag{2.12}$$

This is the reason, why the LPD parameters θ do not enter Eq. 2.11. They are treated as *nuisance parameters* and have been integrated out. It is important to note that the LPD parameters were not maximized as would be done in a maximum likelihood estimate or in a BIC score. Averaging instead of maximizing prevents the Bayesian score from overfitting.

Marginal likelihood of network structure The marginal likelihood $p(M|D)$ is the key component of Bayesian scoring metrics. Its computation depends on the choice of local probability distributions and local priors in the Bayesian network model. To

solve integral (2.12) analytically, the prior $p(\theta|D)$ must fit to the likelihood $p(M|D, \theta)$. Statistically, this fit is called "conjugacy". A prior distribution is called *conjugate* to a likelihood, if the posterior is of the same distributional form as the prior [49]. If no conjugate prior is available, the marginal likelihood has to be approximated. We shortly discuss the LPDs introduced in section 2.2.

- *Discrete LPDs.* The marginal likelihood for discrete Bayesian networks was first computed by Cooper and Herskovits [23]. It is further discussed by Heckerman *et al.* [58]. The conjugate prior for the multinomial distribution is the Dirichlet prior [49]. Assuming independence of the prior for each node and each parent configuration, the score decomposes into independent contributions for each family of nodes.
- *Gaussian LPDs.* Corresponding results exist for Gaussion networks using a Normal-Wishart prior [48]. The marginal likelihood again decomposes into node-wise contributions.
- *CG networks.* Conditional Gaussian networks are a mix of discrete and Gaussian nodes [11]. We discuss the computation of marginal likelihood in detail in section 3.4.2. Discrete and Gaussian marginal likelihoods are treated there as special cases.
- *Regression trees.* The marginal likelihood at each node of the DAG further splits into independent components for each leaf of the local regression tree. Conjugate analysis and analytic results are possible using normal-gamma priors for each leaf node [42, 21].
- *Non-parametric regression.* Conjugate analysis and analytic computation of the marginal likelihood are not possible. Imoto *et al.* [64] use a Laplace approximation to approach the true marginal likelihood.
- *Boolean logic LPDs.* Conjugate analysis and analytic computation of the marginal likelihood are not possible. Instead, Bulashevska and Eils [16] use Gibbs sampling to estimate the model posterior $p(D|M)$ and the parameter posterior $p(\theta|M)$.
- *Kinetic modeling.* Again, conjugate analysis is not possible. Nachman *et al.* [89] use the BIC score for model selection.

Likelihood equivalence It is sensible to postulate that DAGs in the same equivalence class get the same score. The score should not distinguish between undistinguishable models. This requirement limits the choice of permissible prior parameters when computing the marginal likelihood. We discuss here the discrete case of a multinomial node with a Dirichlet prior [58]. The Dirichlet parameters are a set $\{\alpha_{i_\delta|\mathbf{i}_{pa(\delta)}}\}$, each element corresponding to a discrete node δ in state i_δ with discrete parent configuration $\mathbf{i}_{pa(\delta)}$. Likelihood equivalence constrains the Dirichlet parameters to the form

$$\alpha_{i_\delta|\mathbf{i}_{pa(\delta)}} = \alpha \cdot P(I_\delta = i_\delta, \mathbf{I}_{pa(\delta)} = \mathbf{i}_{pa(\delta)}), \tag{2.13}$$

where P is a prior distribution over the joint states of node δ and its parents [58]. The scale parameter α of the Dirichlet prior—often interpreted as "equivalent sample size" or "prior strength"—is positive and independent of δ. It plays an important

role for regularization of network structure (see section 2.3.3). Two *ad hoc* choices are: for all $i_\delta \in \mathcal{I}_\delta$ and $\mathbf{i}_{pa(\delta)} \in \mathcal{I}_{pa(\delta)}$ set

$$\alpha_{i_\delta|\mathbf{i}_{pa(\delta)}} = \begin{cases} 1 & [23], \\ \alpha/|\mathcal{I}_\delta||\mathcal{I}_{pa(\delta)}| & [17]. \end{cases}$$

Both choices result in different scoring metrics. Heckerman *et al.* [58] call the first score the *K2 metric* after the K2 algorithm introduced in [23]. It is not likelihood equivalent. Heckerman calls the second score a *BDeu metric*. The name is an acronym for a *B*ayesian score using a *D*irichlet prior, which is likelihood *e*quivalent and *u*niform. It corresponds to the choice of a uniform prior in Eq. 2.13. How can likelihood equivalence be guaranteed generally? Heckerman *et al.* [58] and Geiger and Heckerman [48] introduce methods to deduce the parameter priors for all possible networks from one joint prior distribution in the discrete and continuous case, respectively. Bøttcher [11] generalizes the results to CG networks.

Structure prior Structure priors $p(D)$ help to focus inference on reasonable models by including biological prior knowledge or integrating different data sources. In some applications the task is not to learn a structure from scratch but to refine a prior network built from biological prior knowledge. The first idea is to restrict the search space to a—conveniently defined—vicinity $\mathcal{V}(\mathcal{P})$ of the prior network $\mathcal{P}$. All the DAGs in the restricted search space are considered equally likely. This can be interpreted as a rigid structure prior of the form

$$p(D) = \begin{cases} 1/|\mathcal{V}(\mathcal{P})| & \text{if } D \in \mathcal{V}(\mathcal{P}) \\ 0 & \text{else} \end{cases} \tag{2.14}$$

A smoother way to guarantee that DAGs similar to the prior network $\mathcal{P}$ get higher prior probability is the following. We measure the confidence of edge (v, w) by a value $0 < \kappa_{vw} \leq 1$. A structure prior can then be defined proportional to a product of weights κ_{vw} over all edges (v, w):

$$p(D) \propto \prod_{v,w \in V} \kappa_{vw}. \tag{2.15}$$

The normalization constant, which would be necessary to make the right-hand side a density, can be ignored when computing relative posterior probabilities. What are smart choices of κ_{vw}? There are several approaches suggested in literature, which are shortly described here.

1. Heckerman *et al.* [58] assume constant penalty $\kappa_{vw} \equiv \kappa$ for all edges, in which D and $\mathcal{P}$ differ. Thus, $p(D) \propto \kappa^\epsilon$ where ϵ is the number of edges in which D differs from the prior DAG $\mathcal{P}$.
2. Another approach [65, 134] uses a network prior in an iterative scheme. They construct a Bayesian network from microarray data, propose putative transcription factors from the network structure, and search for common motifs in the DNA

sequences of children and grand-children of transcription factors. Then, they re-learn the network by penalizing edges without motif evidence harder than edges with motif evidence.

3. Bernard *et al.* [9] define weights from p-values of binding location data. They assume that p-values follow an exponential distribution if the edge is present and a uniform distribution if it is not. By Bayes' rule they derive probabilities for an edge to be present given the p-values from the location data. The free parameter of the exponential distribution is then integrated out. The final probabilities $\mathcal{P}_{vw}$ are used as weights in a structure prior.

Fig. 2.5 shows a comparison of these three prior definitions. They can be organized by the weights κ_{vw} they give for the presence or absence of an edge given prior information in.

	[58]	D = 1	D = 0
Prior $\mathcal{P}$	1	1	κ
	0	κ	1

[65]	D = 1	D = 0
1	$e^{-\xi_1}$	1
0	$e^{-\xi_2}$	1

[9]	D = 1	D = 0
p-value	$\mathcal{P}_{vw}$	$1 - \mathcal{P}_{vw}$

Figure 2.5: *Comparison of edge weights suggested by Heckerman* et al. *[58], Imoto* et al. *[65] and Bernard* et al. *[9]. Rows correspond to prior information. In the left two examples the prior can be described binary, on the right it is expressed as a p-value derived from a second data set. In the middle table holds $\xi_1 < \xi_2$, i.e. edges with motif evidence contribute more than edges without.*

Discretization Most often used in applications is the Bayesian score for discrete data. When learning gene regulatory networks from microarray data, we first need to preprocess the continuous gene expression values and discretize them. In general, discretization may be carried out for computational efficiency, or because background knowledge suggests that the underlying variables are indeed discrete. Discretizing continuous variables results in a loss of information. At the same time, this can be a loss of noise. Discretized data can be more stable with respect to random variations of the mRNA measurements. Several methods to discretize microarray data were proposed in literature:

1. Friedman *et al.* [44] discretize expression values into three categories, depending on whether the expression rate is significantly lower than, similar to, or greater than control, respectively.
2. Pe'er *et al.* [99] introduce an adaptive discretization procedure. They model the expression level of a gene in different experiments as samples from a mixture of normal distributions, where each normal component corresponds to a specific state. Then they use standard k-means clustering to estimate such a mixture.
3. Hartemink *et al.* [56] use a discretization coalescence method, which incrementally reduces the number of discretization levels for each gene while preserving as much total mutual information between genes as possible.

4. In the previous three approaches, expression levels were discretized before and independently of structure learning. Suboptimal discretization policies will lead to degraded network structure. To avoid this, Steck and Jaakkola [129] derive a scoring function to efficiently *jointly* optimize the discretization policy and the structure of the graphical model.

This section provides us with all the methodology we need to decide between candidate regulatory structures by Bayesian scoring. Once we have decided on a discretization policy and on the value of Dirichlet parameters, we need to compute the marginal likelihood of the data for every candidate structure. Biological prior knowledge can be incorporated via a structure prior to bias our choice towards reasonable models. Chapter 3 will give a detailed account of how to compute the marginal likelihood for discrete and Gaussian networks on observational and interventional data.

2.3.3 Regularization

Regularization is a technique used in Machine Learning to ensure uniqueness of solution and to fight overfitting by constraining admissible models [116, 83]. Regularization is always needed in $p \gg N$- situations. We already saw examples of regularization in section 2.1, when Gaussian graphical models were adapted to the $p \gg N$-situation [115, 144]. Different methods were proposed for Bayesian networks.

1. Steck and Jaakkola [128] show that a small scale parameter α in Eq. 2.13 leads to a strong regularization of the model structure and a sparse graph given a sufficiently large data set. In particular, the empty graph is obtained in the limit of a vanishing scale parameter. This is diametrically opposite to what one may expect in this limit, namely the complete graph from an unregularized maximum likelihood estimate.
2. Another way to regularize Bayesian networks is to constrain the forms, the local probability distributions can take. Bulashevska and Eils [16] suggest learning noisy logic gates for parent-child relationships. The drawback is that Bayesian conjugate analysis, which leads to the analytic solution of the marginal likelihood, is no longer possible and Gibbs sampling has to be applied.
3. Module networks [119, 120] constrain the number of parameters in the model by assuming that groups of genes (so called *modules*) share the same dependence on regulators. Learning module networks involves an iteration of assigning genes to modules and searching for dependencies between modules.

2.3.4 Model selection and assessment

Exhaustive search To search for the DAG with highest score is mathematically trivial: compute the score for every possible DAG and choose the one that achieves

the highest value. What makes exhaustive search computationally infeasible is the huge number of DAGs. The number of DAGs on n edges is

$$a_n = \sum_{k=1}^{n} (-1)^{k-1} \binom{n}{k} 2^{k(n-k)} a_{n-k} \tag{2.16}$$

with $a_0 = 1$ [108]. The number of DAGs increases explosively, as the first few steps in the recursion show: 1, 1, 3, 25, 543, 29 281, 3 781 503, 1 138 779 265. That means, we have to think of some heuristic strategy to find high-scoring Bayesian networks without enumerating all possible ones.

Defining search space First we need to decide how to describe models of interest. This defines the model space, in which we search for models describing the data well. To apply search heuristics we have to equip search space with a neighborhood relation, that is, operators to move from one point of the search space to the next one.

1. The most simple search space results from defining a neighborhood relation on DAGs. Two DAGs are neighbors if they differ by one edge, which is either missing in one of them or directed the other way round.
2. Madigan *et al.* [78] and Chickering [20] restrict the search space to Markov equivalence classes of DAGs which uniquely describe a joint distribution. Thus, no time is lost in evaluating DAG models which are equivalent anyway.
3. Friedman and Koller [43] search over orders of nodes rather than over network structures. They argue that the space of orders is smaller and more regular than the space of structures, and has a much smoother posterior landscape.

Search heuristics Most of the following search algorithms can be applied to all search spaces, even though they are usually applied to DAGs. They return a single best network.

1. A simple and fast but still powerful method is *hillclimbing* by greedy search. First, choose a point in search space to start from, e.g. a random graph or the empty graph. Compute the posterior probability for all graphs in the neighborhood of the current graph. Select the graph with highest score. Iterate until no graph in the neighborhood has a larger score than the current graph. This procedure gets you to local maxima of the Bayesian scoring metric. The K2-algorithm [23] is a variant of greedy search, which assumes that the order of nodes is known.
2. The *sparse candidate algorithm* [46] restricts the number of possible parents for each node by searching for pairs of nodes which are highly dependent.
3. The *ideal parent algorithm* [90, 89] constructs a parent profile perfectly explaining the child behaviour and uses it to guide parent selection and to restrict the search space.
4. Peña *et al.* [100] grow Bayesian networks starting from a target gene of interest. They iteratively add to the Bayesian network parents and children of all the genes

already included in it. The algorithm stops after a predefined number of steps and thus, intuitively, highlights the surrounding area of the seed gene without having to compute the complete Bayesian network over all genes.

5. Friedman [39, 40] introduces the *structural EM algorithm* to learn Bayesian networks in the presence of missing values or hidden variables. It is an extension of the Expectation-Maximization (EM) algorithm that performs structure search *inside* the EM procedure.

Assessing uncertainty The problem with optimal models is, as Edwards [35] puts it: "Any method (or statistician) that takes a complex multivariate dataset and, from it, claims to identify one true model, is both naive and misleading". The emphasis is on "one true model". Better than choosing a single best model is to explore the whole posterior distribution. Direct sampling from the posterior is impossible due to the intractability of the denominator in Eq. 2.11, but there are other methods available.

1. The most we know about the data distribution is the empirical distribution of observations in the dataset. A classical approach to assess variability in the data is bootstrapping [36]. The strategy is to sample with replacement from the observations in the data set to get a number of bootstrap datasets, and then learn a network on every bootstrap dataset. The relative frequency of network features in the resulting network structures can be used as a measure of reliability [44, 99].
2. Bootstrap samples can contain multiple copies of identical data points. This implies strong statistical dependencies between variables when given a small dataset. As a consequence, the resulting network structure can be considerably biased towards denser graphs. Steck and Jaakkola [131] propose a correction for this bias.
3. As a simple way to avoid the bootstrap-bias Steck and Jaakkola [129] use the *leave-k-out* method. Instead of resampling with replacement, k cases are left out of the dataset when estimating a model. Repeating this many times also gives an estimate of model variability.
4. Markov Chain Monte Carlo (MCMC) is a simulation technique, which can be used to sample from the posterior $p(D|M)$. Given a network structure, a new neighboring structure is proposed. This new structure is accepted with the Metropolis Hastings acceptance criterion [57]. The iteration of this procedure produces a Markov chain that under fairly general conditions converges in distribution to the true posterior. MCMC is used by Husmeier [62] to learn dynamic Bayesian networks. Madigan *et al.* [78] use MCMC over Markov equivalence classes and Friedman and Koller [43] over orders of nodes.

2.4 Benchmarking

Graphical models visualize a multivariate dependency structure. They can only answer biological questions if they succeed in reliably and accurately reconstructing bi-

ologically relevant features of cellular networks. Unfortunately, rigorous assessment and benchmarking of methods are still rare.

- One of the first evaluation studies is by Smith *et al.* [125]. They sample data from a songbird's brain model and report excellent recovery success when learning a Bayesian network from it.
- Zak *et al.* [155] develop a realistic 10 gene network, where the biological processes at the different levels of transcription, translation and post-translational modifications were modeled with systems of differential equations. They show that linear and log-linear methods fail to recover the network structure.
- Husmeier [62] uses the same simulation network [155] to specify sensitivity and specificity of dynamic Bayesian networks. He demonstrates how the network inference performance varies with the training set size, the degree of inadequacy of prior assumptions, and the experimental sampling strategy. By analyzing ROC curves Husmeier can show fair performance of DBNs.
- Wimberly *et al.* [146] test 10 algorithms, including Boolean and Bayesian networks, on a simulation [14] of the genetic network of the sea urchin embryo [25]. They report that reconstruction is unreliable with all methods and that the performance of the better algorithms quickly degrades as simulations become more realistic.
- Basso *et al.* [7] show that their own method, ARACNe, compares favorably against static Bayesian networks on a simulated network with 19 nodes [154]—but only if the dataset includes several hundreds of observations. On the other hand, Hartemink [55] finds *dynamic* Bayesian networks to be even more accurate than ARACNe on the same dataset.

All in all the results are not promising. Graphical models from microarray data need a big sample size and capture only parts of biologically relevant networks. One reason for this shortcoming is that the models we discussed so far all use purely observational data, where the cellular network was not perturbed experimentally. In simulations [156, 82] and on real data [114] it was found that data from perturbation experiments greatly improve performance in network reconstruction. Thus, the following section 3 will introduce methodology for learning from effects of interventions in a probabilistic framework suitable to capture the noise inherent in biological experiments. This helps to improve the accuracy of network reconstruction.

2.5 A roadmap to network reconstruction

Fig. 2.6 organizes network reconstruction methods with respect to basic questions: Does the data include gene knockout or knockdown experiments? If not, we call it *purely observational data*; if yes, we call it *interventional data*. Is the model probabilistic or deterministic? Does the model allow for changes over time? If yes, we call it *dynamic*, else *static*. Does the model describe transcriptional regulatory networks? And if yes: are additional non-transcriptional effects taken into account?

In the leaf nodes of the decision tree methods fall together that are methodologically similar. Some branches in the tree are missing. Mostly, the reason is not that it would be impossible to follow them, but simply that we found no approach doing it.

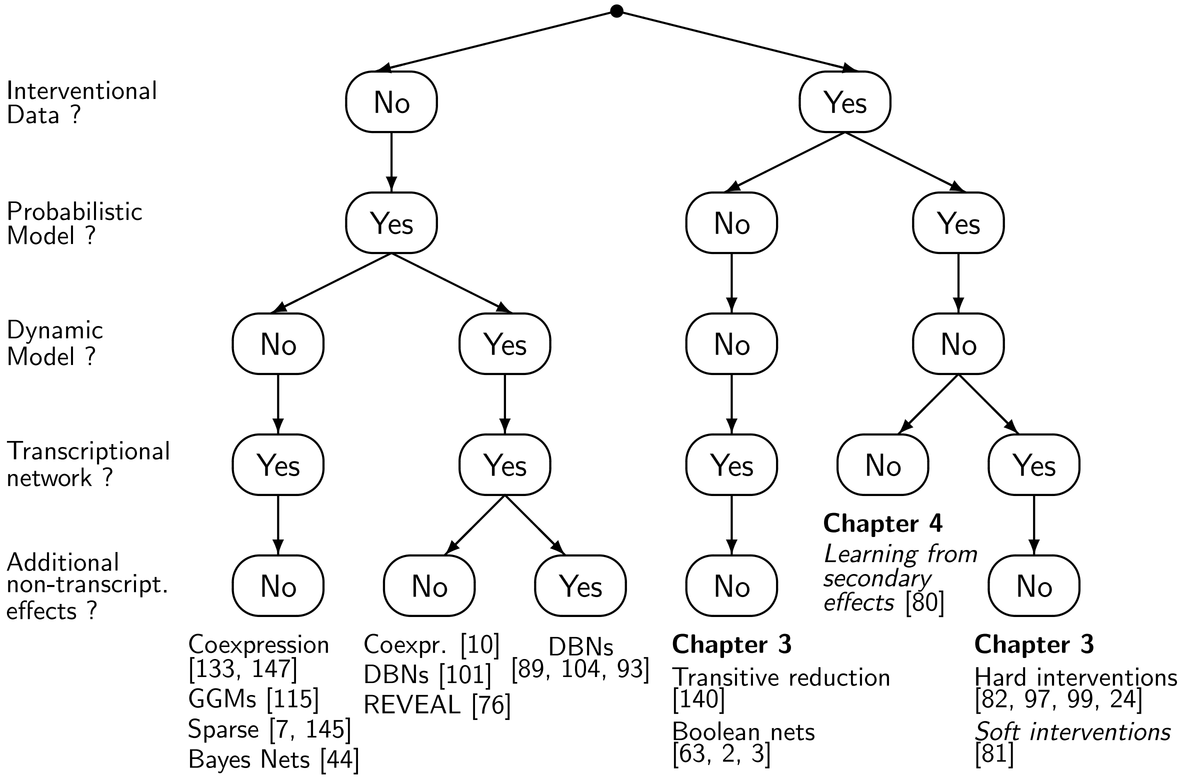

Figure 2.6: *A guide to the literature on network reconstruction. The methods discussed in this section all fall into the left branch of the tree. The next two sections will deal with learning transcriptional regulatory networks and non-transcriptional pathways from interventions. The main contributions of this dissertation are* soft interventions *and* learning from secondary effects.

Fig. 2.6 shows representative examples and relates our own methods to other approaches. The main contributions of this dissertation are soft interventions and learning from secondary effects. They can be found in the right-most branch of the tree. Both are static probabilistic models for interventional data. Soft interventions are used for gene regulation networks, in which effects of interventions can be observed at the other genes in the model. Learning from secondary effects infers non-transcriptional pathway features from expression data. This model expands the mRNA centered view of graphical models to non-transcriptional parts of signaling pathways.

Chapter 3

Inferring transcriptional regulatory networks

The last chapter described statistical models to infer the topology of cellular networks by elucidating the correlation structure of pathway components. This chapter extends these models to include direct observations of intervention effects at other pathway components (section 3.1). The main contribution is a general concept of probabilistic interventions in Bayesian networks. My approach generalizes deterministic interventions, which fix nodes to certain states (section 3.2). I propose "pushing" variables in the direction of target states without fixing them (section 3.3) and formalize this idea in a Bayesian framework based on conditional Gaussian networks (section 3.4).

3.1 Graphical models for interventional data

In modern biology, the key to inferring gene function and regulatory pathways are experiments with interventions into the normal course of action in a cell. A common technique is to perturb a gene of interest experimentally and to study which other genes' activities are affected. A number of deterministic and probabilistic techniques have been proposed to infer regulatory dependencies from primary effects. In this section, we will give an overview over recent approaches, which are extensions of the methods discussed in the last chapter.

Linking causes with effects Rung *et al.* [112] build a directed graph by drawing an edge (i, j) if perturbing gene i results in a significant expression change at gene j. The authors focus on features of the network that are robust over a range of significance cutoffs. The inferred networks do not distinguish between direct and indirect effects. In this sense they are similar to co-expression networks. Fig. 3.1 shows the difference between a causal network and a network of affected components. In graph-theoretic terminology, the second network is the transitive closure of the first one.

Distinguishing direct from indirect effects A transitively closed network can be used as a starting point for further analysis. Wagner [142, 141, 140] uses graph-theoretic methods of *transitive reduction* [1, 75] to find the most parsimonious sub-graph explaining all observed effects. These methods are deterministic and do not

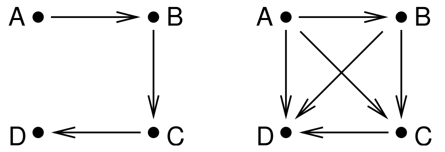

Figure 3.1: *From the causal network (left) it is easy to deduce how effects spread through the pathway (right). The harder problem is to deduce the causal pathway from observing effects of interventions (going from right to left).*

account for measurement noise. Wang and Cooper [143] describe a Bayesian generalization of the Wagner algorithm [140] yielding a distribution over possible causal relationships between genes.

Boolean networks A simple deterministic model of regulatory networks are Boolean networks: they are defined by a directed (and possibly cyclic) graph. Nodes correspond to genes and can take values 0 and 1. For each node exists a boolean function relating parent states to the child state. Perturbations allow to infer the structure and the logic of Boolean networks [63, 2, 3].

Correlation Rice *et al.* [107] build correlation graphs on knockout data. They assume that the data contain measurements of the unperturbed cell and several replicates of measurements for every gene knockout. For each gene i, they combine the wild-type data with the intervention data of this gene and compute on the joint data the correlation of gene i to all other genes. In the final graph, there is an arrow (i, j) whenever gene j was highly correlated to gene i. Since the correlation was computed on knockout data, the graph encodes causation and not only correlation. The big disadvantage of the method is the need for many ($\geq$ 10) replicates of knockout experiments for every gene in the model. Data are used more efficiently by several regression methods.

Regression Rogers and Girolami [109] use sparse Bayesian regression based on a Gaussian linear model. They regress each gene onto all other genes by combining all the data corresponding to knockouts of genes other than the particular gene of interest. The measurements of the knockout gene are ignored when predicting this gene's expression from the other genes. In the next section we will see that this strategy is the same as Pearl's *ideal interventions* used in Bayesian networks [97]. A prior on model parameters constrains most regression coefficients to zero and enforces a sparse solution. Non-zero regression coefficients are indicated by arrows in the regulation network. The resulting graph is a special case of a Gaussian graphical model where directed edges are justified because the dataset contained knockouts of predictor variables.

Other regression methods for network reconstruction are derived from a branch of engineering called *system identification* [77]. Functional relations between network components are inferred from measurements of system dynamics. Several papers [151, 47, 28, 29] use multiple regression to model the response of genes and proteins to external perturbations.

Bayesian networks Bayesian networks represent the finest resolution of correlation structure. As shown in section 2.2, they present a prominent approach to derive

a theoretical model for regulatory networks and pathways. Genes are represented by vertices of a network and the task is to find a topology, which explains dependencies between the genes. When learning from observational data only, groups of Bayesian networks may be statistically indistinguishable [139] as discussed in section 2.2. Information about effects of interventions helps to resolve such equivalence classes by including causal knowledge into the model [136, 137]. The final goal is to learn a graph structure, which not only represents statistical dependencies, but also causal relations between genes.

The following sections develop a theory for learning Bayesian network structure when data from different gene perturbation experiments is available. Section 3.2 reviews classical theory on modelling interventions in Bayesian networks. It shows that these concepts do not fit to realistic biological situations. A more appropriate model is introduced in section 3.3. It develops a theory of *soft interventions*, which push an LPD towards a target state without fixing it. A soft intervention can be realized by introducing a "pushing parameter" into the local prior distribution, which captures the pushing strength. We propose a concrete parametrization of the pushing parameter in the classical cases of discrete and Gaussian networks. Ideal interventions, which have been formally described by choosing a Dirac prior [137], can then be interpreted as infinite pushing.

Section 3.4 summarizes the results in the general setting of conditional Gaussian networks. This extends the existing theory on learning with hard interventions in discrete networks to learning with soft interventions in networks containing discrete and Gaussian variables. The concluding Section 3.4.3 deals with *probabilistic* soft interventions. In this set-up the pushing parameter becomes a random variable and we assign a prior to it. Hence, we account for the experimentalist's lack of knowledge on the actual strength of intervention by weighted averaging over all possible values.

3.2 Ideal interventions and mechanism changes

It is crucial that models reflect the way data was generated in the perturbation experiments. In Bayesian structure learning, Tian and Pearl [137] show that interventions can be modeled by imposing different parameter priors when the gene is actively perturbed or passively observed. They only distinguish between two kinds of interventions: most generally, interventions that change the local probability distribution of the node within a given family of distributions, and as a special case, interventions that fix the state of the variable deterministically. The first is called a *mechanism change*. It does not assume any prior information on *how* the local probability distribution changes. The second type of intervention, which fixes the state of the variable, is called a *do-operator* [97]. We will shortly describe both approaches to motivate our own model, which can be seen as lying intermediate these two extremes.

Ideal interventions Pearl [97] proposes an idealized *do-operator* model, in which the manipulation completely controls the node distribution. The influence of parent

nodes is removed and the LPD $p(x_v|\mathbf{x}_{pa(v)}, \theta_v)$ degenerates to a point mass at the target state x'_v, that is,

$$p(x_v|\mathbf{x}_{pa(v)}, \theta_v) \xrightarrow{\mathrm{do}(X_v = x'_v)} p(x_v) = \begin{cases} 1 & \text{if } x_v = x'_v \\ 0 & \text{else.} \end{cases} \tag{3.1}$$

Fixing a variable to a state tells us nothing about its "natural" behaviour. When considering a single variable, data in which it was experimentally fixed has to be omitted. Cooper and Yoo [24] show: the marginal likelihood for data including interventional cases is of the same form as for observational cases only, but the counts go only over observations where a node was not fixed by external manipulation. We will discuss this result more deeply in section 3.4.

We will call Pearl's model a *hard (pushing) intervention*: it is directed to a target state and fixes the LPD deterministically. Hard interventions are used in almost all applications of interventional learning in Bayesian networks [152, 153, 130, 138, 99, 88, 22, 24].

A simulation study To test the effect of ideal interventions on structure learning, we conducted a simulation study on a small network of five nodes. Here, exhaustive enumeration is still possible and we can assess the complete score landscape. The simulation evaluated reconstruction accuracy with varying levels of noise and three different dataset sizes. The LPDs are convex combinations of signal and noise regulated by a parameter κ. The technical set-up is summarized in Fig. 3.2.

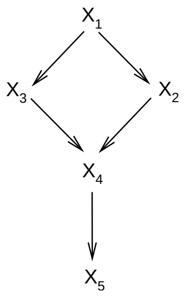

Figure 3.2: *The network topology used in the simulation. All random variables can take three values. For each parent state, the LPDs are a convex combination* $\kappa \cdot$ signal $+ (1 - \kappa) \cdot$ noise, *where "noise" is a uniform distribution over the three states and "signal" propagates the parent state. If X_2 and X_3 disagree, X_4 chooses uniformly between the two signals. More technical details are found in [82].*

Varying κ in steps of 0.1 in the intervall $[0, 0.9]$ we sampled two datasets of the same size: one only containing passive observations, and one sampled after ideal interventions at each node with equal number of replicates for each intervention experiment. On both datasets we scored all possible DAGs on 5 nodes and counted differences between the true and the top scoring topology. As errors we counted missing and spurious edges and also false edge directions. All these features are important when interpreting network topologies biologically.

The results of 5 repetitions can be seen in Fig. 3.3. The more data and the clearer the signal, the more pronounced is the advantage of active interventional learning over purely observational learning. While observational learning results in three equivalent topologies with the same high score, interventional learning resolves these ambiguities and yields a single best model. In summary, interventions are critical for effective

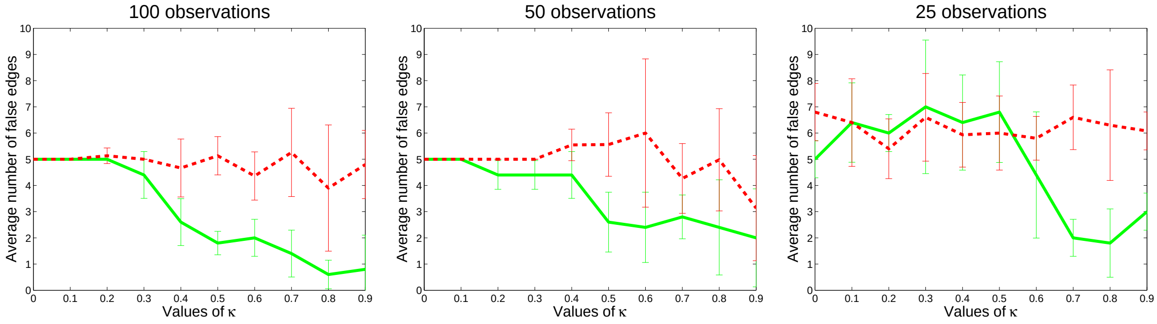

Figure 3.3: *Results of simulation experiments. The red dashed line corresponds to learning from observational data, the green solid line from learning with interventions. The bigger the sample-size and the clearer the signal, the larger is the gap between both lines.*

inference, particularly to establish directionality of the connections. Recently, this finding has been confirmed in other simulations [156] and on real data [114].

Mechanism changes Tian and Pearl [137] propose a model for local spontaneous changes that alter LPDs. They assume that no knowledge is available on the nature of the change, its location, or even whether it took place. Tian and Pearl derive a Bayesian score for structure learning by splitting the marginal likelihood for a node, at which a local change occurred, into two parts: one for the cases obtained before the change and one for the cases obtained after the change. A hard intervention as in Eq. (3.1) can be incorporated in this framework by assigning an informative prior distribution to the second part of the marginal likelihood. Tian and Pearl [137] show that the assumption (or knowledge) that only a *single* causal mechanism has changed, increases power in structure learning. Previously indistinguishable equivalent topologies may now be distinguished.

Problems Both hard interventions and mechanism changes face problems when being applied to real biological data from gene silencing experiments. Pearl's model of ideal interventions contains a number of idealizations: manipulations only affect single genes and results can be controlled deterministically. The first assumption may not be true for drug treatment and even in the case of single-gene knockouts there may be compensatory effects involving other genes. The second assumption is also very limiting in realistic biological scenarios. Often the experimentalist lacks knowledge about the exact size of perturbation effects. Due to measurement error or noise inherent in the observed system it may often happen that a variable, at which an intervention took place, is observed in a state different from the target state. In Pearl's framework, a single observation of this kind results in a marginal likelihood of zero. Mechanism changes, on the other hand, are also not suited to model real biological experiments, even though they capture uncertainty on intervention strength and accuracy. In real applications to reverse screens, at least the target of intervention is known and there is an expected response of the target to the intervention. Gene perturbations are *directed* in the sense that the experimental technique used tells us whether we should expect more or less functional target mRNA in the cell.

In summary, we need interventional data for successfull small-sample network reconstruction. Hard interventions (do-operations) are deterministic, mechanism changes are undirected. Both frameworks do not fit realistic biological situations. If we treat gene perturbation experiments as unfocussed mechanism changes we lose valuable information about what kind of intervention was performed. If we model them by a do-operator, we underestimate the stochastic nature of biological experiments. Thus, we need a concept of interventions, which is more directed than general mechanism changes, but still softer than deterministic fixing of variables. In the following, we focus on interventions, which specifically concentrate the local distribution at a certain node around some target state. We will call them *pushing interventions*, they are examples of mechanism changes with prior knowledge. We generalize hard pushing interventions (do-operator) to *soft pushing interventions*: the local probability distribution only centers more around the target value without being fixed. We follow Tian and Pearl [137] in splitting the marginal likelihood locally in two parts and assigning informative prior distributions. All interventions we will discuss are external manipulations of *single* nodes. None of them models global changes in the environment, which would change the dependency structure over the whole network and not just in a single family of nodes. Thus, we can start explaining soft interventions in the next section by concentrating on a single node in a Bayesian network.

3.3 Pushing interventions at single nodes

A Bayesian network is a graphical representation of the dependency structure between the components of a random vector $\mathbf{X}$. The individual random variables are associated with the vertices of a directed acyclic graph (DAG) D, which describes the dependency structure. Once the states of its parents are given, the probability distribution of a given node is fixed. Thus, the Bayesian network is completely specified by the DAG and the local probability distributions (LPDs). Although this definition is quite general, there are basically three types of Bayesian networks which are used in practice: discrete, Gaussian and conditional Gaussian (CG) networks. CG networks are a combination of the former two and will be treated in more detail in Section 3.4, for the rest of this section we focus on discrete and Gaussian networks. In discrete and Gaussian networks, LPDs are taken from the family of the multinomial and normal distribution, respectively. In the theory of Bayesian structure learning, the parameters of these distributions are not fixed, but instead a prior distribution is assumed [23, 48, 11]. The priors usually chosen because of conjugacy are the Dirichlet distribution in the discrete case and the Normal-inverse-χ^2 distribution in the Gaussian case. Averaging the likelihood over these priors yields the marginal likelihood – the key quantity in structure learning (see section 2.3.2).

An intervention at a certain node in the network can in this setting easily be modeled by a change in the LPDs' prior. When focusing on (soft) pushing interventions, this change should result in an increased concentration of the node's LPD around the

target value. We model this concentration by introducing a pushing parameter w, which measures the strength of the pushing. A higher value of w results in a stronger concentration of the LPD. We now explain in more detail how this is done for discrete and Gaussian networks. Since the intervention only affects single variables and the joint distribution $p(\mathbf{x})$ in a Bayesian network factors according to the DAG structure in terms only involving a single node and its parents, it will suffice to treat families of discrete and Gaussian nodes separately.

3.3.1 Pushing by Dirichlet priors

We denote the set of discrete nodes by Δ and a discrete random variable at node $\delta \in \Delta$ by I_δ. The set of possible states of I_δ is $\mathcal{I}_\delta$. The parametrization of the discrete LPD at node δ is called θ_δ. For every configuration $\mathbf{i}_{pa(\delta)}$ of parents, θ_δ contains a vector of probabilities for each state $i_\delta \in \mathcal{I}_\delta$. Realizations of discrete random variables are multinomially distributed with parameters depending on the state of discrete parents. The conjugate prior is Dirichlet with parameters also depending on the state of discrete parents:

$$\begin{aligned} I_\delta \mid \mathbf{i}_{pa(\delta)}, \theta_\delta &\sim \text{Multin}(1, \theta_{\delta|\mathbf{i}_{pa(\delta)}}), \\ \theta_{\delta|\mathbf{i}_{pa(\delta)}} &\sim \text{Dirichlet}(\alpha_{\delta|\mathbf{i}_{pa(\delta)}}). \end{aligned} \tag{3.2}$$

We assume that the $\alpha_{\delta|\mathbf{i}_{pa(\delta)}}$ are chosen to respect likelihood equivalence [58]. A pushing intervention at node δ amounts to changing the prior parameters $\alpha_{\delta|\mathbf{i}_{pa(\delta)}}$ such that the multinomial density concentrates at some target value j. We formalize this by introducing a pushing operator $\mathcal{P}$ defined by

$$\mathcal{P}(\alpha_{\delta|\mathbf{i}_{pa(\delta)}}, w_\delta, j) \;=\; \alpha_{\delta|\mathbf{i}_{pa(\delta)}} + w_\delta \cdot \mathbf{1}_j, \tag{3.3}$$

where $\mathbf{1}_j$ is a vector of length $|\mathcal{I}_\delta|$ with all entries zero except for a single 1 at state j. The pushing parameter $w_\delta \in [0, \infty]$ determines the strength of intervention at node δ: if $w_\delta = 0$ the prior remains unchanged, if $w_\delta = \infty$ the Dirichlet prior degenerates to a Dirac distribution and fixes the LPD to the target state j. Figure 3.4 shows a three-dimensional example of increasing pushing strength w_δ.

3.3.2 Pushing by Normal-inverse-χ^2 priors

The set of Gaussian nodes will be called Γ and we denote a Gaussian random variable at node $\gamma \in \Gamma$ by Y_γ. In the purely Gaussian case it depends on the values of parents $\mathbf{Y}_{pa(\gamma)}$ via a vector of regression coefficients β_γ. If we assume that β_γ contains a first entry $\beta_\gamma^{(0)}$, the parent-independent contribution of Y_γ, and attach to $\mathbf{Y}_{pa(\gamma)}$ a leading 1, we can write for Y_γ the following regression model

$$\begin{aligned} Y_\gamma \mid \beta_\gamma, \sigma_\gamma^2 &\sim \text{N}(\mathbf{Y}_{pa(\gamma)}^\top \beta_\gamma,\ \sigma_\gamma^2), \\ \beta_\gamma \mid \sigma_\gamma^2 &\sim \text{N}(\mathbf{m}_\gamma,\ \sigma_\gamma^2 \mathbf{M}_\gamma^{-1}), \\ \sigma_\gamma^2 &\sim \text{Inv-}\chi^2(\nu_\gamma,\ s_\gamma^2). \end{aligned} \tag{3.4}$$

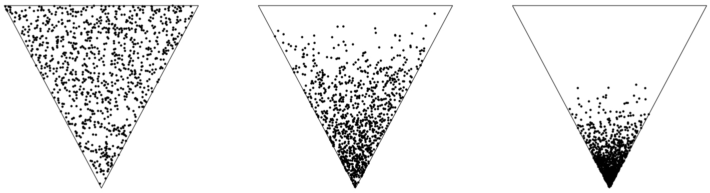

Figure 3.4: *Examples of pushing a discrete variable with three states. Each triangle represents the sample space of the three-dimensional Dirichlet distribution (which is the parameter space of the multinomial likelihood of the node). The left plot shows a uniform distribution with Dirichlet parameter $\alpha = (1, 1, 1)$. The other two plots show effects of pushing with increasing weight: $w = 3$ in the middle and $w = 10$ at the right. In each plot 1000 points were sampled.*

The regression coefficients follow a multivariate normal distribution with mean $\mathbf{m}_\gamma$ and covariance matrix $\sigma^2_\gamma \mathbf{M}_\gamma^{-1}$, where σ^2_γ is the variance of node Y_γ. The variance follows an inverse-χ^2 distribution. We assume that the prior parameters $\mathbf{m}_\gamma, \mathbf{M}_\gamma, \nu_\gamma, s^2_\gamma$ are chosen as in ref. [11].

As for discrete nodes, we implement a pushing intervention by adapting the prior distributions of model parameters. Pushing the distribution of Y_γ to target value k involves moving the mean by adapting the distribution of regression coefficients and concentrating the distribution by decreasing the variance σ^2_γ. To this end, we propose to exchange $\mathbf{m}_\gamma$ and s^2_γ by $(\bar{\mathbf{m}}_\gamma, \bar{s}^2_\gamma) = \mathcal{P}((\mathbf{m}_\gamma, s^2_\gamma), w_\gamma, k)$ defined by

$$\begin{aligned} \bar{\mathbf{m}}_\gamma &= e^{-w_\gamma} \cdot \mathbf{m}_\gamma + (1 - e^{-w_\gamma}) \cdot k\mathbf{1}_1, \\ \bar{s}^2_\gamma &= s^2_\gamma / (w_\gamma + 1), \end{aligned} \tag{3.5}$$

where $k\mathbf{1}_1$ is a vector of length $|\mathbf{i}_{pa(\gamma)}| + 1$ with all entries zero except the first, which is k. We use $\mathcal{P}$ for the pushing operator as in the case of discrete nodes; which one to use will be clear from the context. Again $w_\gamma \in [0, \infty]$ represents intervention strength. The exponential function maps the real valued w into the interval $[0, 1]$. The interventional prior mean $\bar{\mathbf{m}}$ is a convex combination of the original mean $\mathbf{m}$ with a "pushing" represented by $k\mathbf{1}_1$. If $w_\gamma = 0$ the mean of the normal prior and the scale of the inverse-χ^2 prior remain unchanged. As $w_\gamma \to \infty$ the scale $\bar{s}^2$ goes to 0, so the prior for σ^2 tightens at 0. At the same time, the regression coefficients of the parents converge to 0 and β_0 approaches target value k. All in all, with increasing w_γ the distribution of Y_γ peaks more and more sharply at $Y_\gamma = k$. Note that the discrete pushing parameter w_δ and the Gaussian pushing parameter w_γ live on different scales and will need to be calibrated individually.

3.3.3 Hard pushing

Hard pushing means to make sure that a certain node's LPD produces almost surely a certain target value. It has been proposed by Tian and Pearl [137] to model this by

imposing a Dirac prior on the LPD of the node. Although the Dirac prior is no direct member of neither the Dirichlet nor the Normal-inverse-χ^2 family of distributions it arises for both of them when taking the limit $w \to \infty$ for the pushing strength. Tian and Pearl [137] give an example for discrete networks by

$$p(\theta_{\delta|\mathbf{i}_{pa(\delta)}} \mid \mathrm{do}(X_\delta = x'_\delta)) = d(\theta_{i'_\delta|\mathbf{i}_{pa(\delta)}} - 1) \prod_{i_\delta \neq i'_\delta} d(\theta_{i_\delta|\mathbf{i}_{pa(\delta)}}), \tag{3.6}$$

where $d(\cdot)$ is the Dirac function: $d(x) = 1$, if $x = 0$, and $d(x) = 0$ else. This choice of the local prior distribution ensures that

$$\theta_{i_\delta|\mathbf{i}_{pa(\delta)}} = \begin{cases} 1 & \text{for } I_\delta = i'_\delta, \\ 0 & \text{else,} \end{cases}$$

in agreement with the definition of hard interventions in Eq. (3.1). We can easily extend this approach to Gaussian networks by defining a prior density as

$$p(\beta_\gamma, \sigma^2_\gamma \mid \mathrm{do}(Y_\gamma = k)) = d(\beta^{(0)}_\gamma - k) \prod_{i \in pa(\gamma)} d(\beta^{(i)}_\gamma) \cdot d(\sigma^2_\gamma). \tag{3.7}$$

Averaging over this prior sets the variance and the regression coefficients to zero, while $\beta^{(0)}_\gamma$ is set to k. Thus, the marginal distribution of Y_γ is fixed to state k with probability one.

3.3.4 Modeling interventions by policy variables

Hard interventions can be modeled by introducing a policy variable as an additional parent node of the variable at which the intervention is occuring [97, 127, 73]. In the same way we can use policy variables to incorporate soft interventions. For each node v, we introduce an additional parent node F_v ("F" for "force"), which is keeping track of whether an intervention was performed at X_v or not, and if yes, what the target state was. For a discrete variable I_δ, the policy variable F_δ has state space $\mathcal{I}_\delta \cup \emptyset$ and we can write

$$p(\theta_{\delta|\mathbf{i}_{pa(\delta)}, f_\delta}) = \begin{cases} \mathrm{Dirichlet}(\alpha_{\delta|\mathbf{i}_{pa(\delta)}}) & \text{if } F_\delta = \emptyset, \\ \mathrm{Dirichlet}(\bar{\alpha}_{\delta|\mathbf{i}_{pa(\delta)}}) & \text{if } F_\delta = j, \end{cases} \tag{3.8}$$

where $\bar{\alpha}_{\delta|\mathbf{i}_{pa(\delta)}} = \mathcal{P}(\alpha_{\delta|\mathbf{i}_{pa(\delta)}}, w_\delta, j)$ is derived from $\alpha_{\delta|\mathbf{i}_{pa(\delta)}}$ as defined in Eq. (3.3). For a continuous variable Y_γ, the policy variable F_γ has state space $\mathrm{I\!R} \cup \emptyset$ and we can write

$$p(\beta_{\gamma|f_\gamma}, \sigma^2_{\gamma|f_\gamma}) = \begin{cases} \mathrm{N}(\mathbf{m}_\gamma, \sigma^2_\gamma \mathbf{M}^{-1}_\gamma) \cdot \mathrm{Inv}\text{-}\chi^2(\nu_\gamma, s^2_\gamma) & \text{if } F_\gamma = \emptyset, \\ \mathrm{N}(\bar{\mathbf{m}}_\gamma, \sigma^2_\gamma \mathbf{M}^{-1}_\gamma) \cdot \mathrm{Inv}\text{-}\chi^2(\nu_\gamma, \bar{s}^2_\gamma) & \text{if } F_\gamma = k, \end{cases} \tag{3.9}$$

where $(\bar{\mathbf{m}}_\gamma, \bar{s}^2_\gamma) = \mathcal{P}((\mathbf{m}_\gamma, s^2_\gamma), w_\gamma, k)$ as defined in Eq. (3.5). Equations (3.8) and (3.9) will be used in section 3.4.2 to compute the marginal likelihood of conditional Gaussian networks from a mix of interventional and non-interventional data.

3.4 Pushing in conditional Gaussian networks

We summarize the results of the last section in the general framework of conditional Gaussian networks and compute the marginal likelihood for learning from soft interventions.

3.4.1 Conditional Gaussian networks

Conditional Gaussian (CG) networks are Bayesian networks encoding a joint distribution over discrete and continuous variables. We consider a random vector $\mathbf{X}$ splitting into two subsets: $\mathbf{I}$ containing discrete variables and $\mathbf{Y}$ containing continuous ones. The dependencies between individual variables in $\mathbf{X}$ can be represented by a directed acyclic graph (DAG) D with node set V and edge set E. The node set V is partitioned as $V = \Delta \cup \Gamma$ into nodes of discrete (Δ) and continuous (Γ) type. Each discrete variable corresponds to a node in Δ and each continuous variable to a node in Γ. The distribution of a variable X_v at node v only depends on variables $\mathbf{X}_{pa(v)}$ at parent nodes $pa(v)$. Thus, the joint density $p(\mathbf{x})$ decomposes as

$$\begin{aligned} p(\mathbf{x}) &= p(\mathbf{i}, \mathbf{y}) = p(\mathbf{i})p(\mathbf{y}|\mathbf{i}) \\ &= \prod_{\delta \in \Delta} p(i_\delta | \mathbf{i}_{pa(\delta)}) \cdot \prod_{\gamma \in \Gamma} p(y_\gamma | \mathbf{y}_{pa(\gamma)}, \mathbf{i}_{pa(\gamma)}). \end{aligned} \tag{3.10}$$

The discrete part, $p(\mathbf{i})$, is given by an unrestricted discrete distribution. The distribution of continuous random variables given discrete variables, $p(\mathbf{y}|\mathbf{i})$, is multivariate normal with mean and covariance matrix depending on the configuration of discrete variables. Since discrete variables do not depend on continuous variables, the DAG D contains no edges from nodes in Γ to nodes in Δ.

For discrete nodes, the situation in CG networks is exactly the same as in the pure case discussed in Section 3.3: The distribution of $I_\delta | \mathbf{i}_{pa(\delta)}$ is multinomial and parametrized by θ_δ. Compared to the purely Gaussian case treated in Section 3.3, we have for Gaussian nodes in CG networks an additional dependency on discrete parents. This dependency shows in the regression coefficients and the variance, which now not only depend on the node, but also on the state of the discrete parents:

$$Y_\gamma \mid \beta_{\gamma|\mathbf{i}_{pa(\gamma)}}, \sigma^2_{\gamma|\mathbf{i}_{pa(\gamma)}} \sim \mathrm{N}(\mathbf{Y}^\top_{pa(\gamma)} \beta_{\gamma|\mathbf{i}_{pa(\gamma)}},\ \sigma^2_{\gamma|\mathbf{i}_{pa(\gamma)}}). \tag{3.11}$$

As a prior distribution we again take the conjugate normal-inverse-χ^2 distribution as in Eq. (3.4). For further details on CG networks we refer to references [72, 11].

3.4.2 Learning from interventional and non-interventional data

Assuming an uniform prior over network structures D, the central quantity to be calculated is the *marginal likelihood* $p(M|D)$. In the case of only one type of data it can be written as

$$p(M|D) \;=\; \int_{\Theta} p(M|D,\theta)\; p(\theta|D)\; \mathrm{d}\theta. \tag{3.12}$$

Here $p(\theta|D)$ is the prior on the parameters θ of the LPDs. If the dataset contains both interventional and non-interventional cases, the basic idea is to choose parameter priors locally for each node as in Eq. (3.8) and Eq. (3.9) according to whether a variable was perturbed in a certain case or not. We will see that this strategy effectively leads to a local split of the marginal likelihood into an interventional and a non-interventional part.

A family-wise view of marginal likelihood To compute the marginal likelihood of CG networks on interventional and non-interventional data, we rewrite Eq. (3.12) in terms of single nodes such that the theory of (soft) pushing from Section 3.3 can be used. In the computation we will use the following technical utilities:

1. The dataset M consists of N cases $\mathbf{x}^1, \ldots, \mathbf{x}^N$, which are sampled independently. Thus we can write $p(M|D,\theta)$ as a product over all single case likelihoods $p(\mathbf{x}^c|D,\theta)$ for $c = 1, \ldots, N$.
2. The joint density $p(\mathbf{x})$ factors according to the DAG D as in Eq. (3.10). Thus, for each case $\mathbf{x}^c$ we can write $p(\mathbf{x}^c|D,\theta)$ as a product over node contributions $p(x_v^c|\mathbf{x}_{pa(v)}^c, \theta_v)$ for all $v \in V$.
3. We assume *parameter independence*: the parameters associated with one variable are independent of the parameters associated with other variables, and the parameters are independent for each configuration of the discrete parents [58]. Thus, all dependencies between variables are encoded in the network structure. Parameter independence allows us to decompose the prior $p(\theta|D)$ in Eq. (3.12) into node-wise priors $p(\theta_{v|\mathbf{i}_{pa(v)}}|D)$ for a given parent configuration $\mathbf{i}_{pa(v)}$.
4. All interventions are soft pushing. For a given node, intervention strength and target state stay the same in all cases in the data, but of course different nodes may have different pushing strengths and target values. This constraint just helps us to keep the following formulas simple and can easily be dropped.

These four assumptions allow a family-wise view of the marginal likelihood. Before we present it in a formula, it will be helpful to introduce a *batch notation.* In CG networks, the parameters of the LPD at a certain node depend only on the configuration of discrete parents. This holds for both discrete and Gaussian nodes. Thus, when evaluating the likelihood of data at a certain node, it is reasonable to collect

all cases in a batch, which correspond to the same parent configuration:

$$\begin{aligned} p(M|D,\theta) &= \prod_{c\in M} p(\mathbf{x}^c|D,\theta) \\ &= \prod_{c\in M}\prod_{v\in V} p(x_v^c|\mathbf{x}^c_{pa(v)},\theta_v) \\ &= \prod_{v\in V}\prod_{\mathbf{i}_{pa(v)}\in\mathcal{I}_{pa(v)}}\prod_{c:\mathbf{i}^c_{pa(v)}=\mathbf{i}_{pa(v)}} p(x_v^c|\mathbf{i}^c_{pa(v)},\mathbf{y}_{pa(v)},\theta_v) \end{aligned} \quad (3.13)$$

The last formula is somewhat technical: If the node v is discrete, then $\mathbf{y}_{pa(v)}$ will be empty, and usually not all parent configuration $\mathbf{i}_{pa(v)}$ are found in the data, so some terms of the product will be missing. For each node we gather the cases with the same joint parent state in a batch $B_{\mathbf{i}_{pa(v)}} = \{c \in 1,\dots,N \ : \ \mathbf{i}^c_{pa(v)} = \mathbf{i}_{pa(v)}\}$. When learning with interventional data, we have to distinguish further between observations of a variable which were obtained passively and those that are result of intervention. Thus, for each node v we split the batch $B_{\mathbf{i}_{pa(v)}}$ into one containing all observational cases and one containing the interventional cases:

$$\begin{aligned} B^{obs}_{\mathbf{i}_{pa(v)}} &= \{c \in 1,\dots,N \ : \mathbf{i}^c_{pa(v)} = \mathbf{i}_{pa(v)} \text{ and no intervention at } v\}, \\ B^{int}_{\mathbf{i}_{pa(v)}} &= \{c \in 1,\dots,N \ : \mathbf{i}^c_{pa(v)} = \mathbf{i}_{pa(v)} \text{ and intervention at } v\}. \end{aligned}$$

If there is more than one type of intervention applied to node v, the batch containing interventional cases has to be split accordingly. Using this notation we can now write down the marginal likelihood for CG networks in terms of single nodes and parents:

$$\begin{aligned} p(M|D) = &\prod_{v\in V}\prod_{\mathbf{i}_{pa(v)}}\int_\Theta \prod_{o\in B^{obs}_{\mathbf{i}_{pa(v)}}} p(x_v^o|\mathbf{i}_{pa(v)},\mathbf{y}^o_{pa(v)},\theta_v)\ p'(\theta_v|D)\ \mathrm{d}\theta_v \ \times \\ &\prod_{v\in V}\prod_{\mathbf{i}_{pa(v)}}\int_\Theta \prod_{e\in B^{int}_{\mathbf{i}_{pa(v)}}} p(x_v^e|\mathbf{i}_{pa(v)},\mathbf{y}^e_{pa(v)},\theta_v)\ p''(\theta_v|D,w_v)\ \mathrm{d}\theta_v. \end{aligned} \quad (3.14)$$

At each node, we use distributions and priors as defined in Eq. (3.8) for discrete nodes and Eq. (3.9) for Gaussian nodes. The non-interventional prior p' corresponds to $F_v = \emptyset$ and the interventional prior p'' corresponds to F_v equalling some target value. We denoted the intervention strength explicitly in the formula, since we will focus on it further when discussing *probabilistic* soft interventions in Section 3.4.3. Equation (3.14) consists of an observational and an interventional part. Both can further be split into a discrete and a Gaussian part, so we end up with four terms to consider.

Discrete observational part To write down the marginal likelihood of discrete observational data, we denote by $n_{i_\delta|\mathbf{i}_{pa(\delta)}}$ the number of times we passively observe $I_\delta = i_\delta$ in batch $B^{obs}_{\mathbf{i}_{pa(\delta)}}$, and by $\alpha_{i_\delta|\mathbf{i}_{pa(\delta)}}$ the corresponding pseudo-counts of the Dirichlet prior. Summation of $\alpha_{i_\delta|\mathbf{i}_{pa(\delta)}}$ and $n_{i_\delta|\mathbf{i}_{pa(\delta)}}$ over all $i_\delta \in \mathcal{I}_\delta$ is abbreviated by $\alpha_{\mathbf{i}_{pa(\delta)}}$

and $n_{\mathbf{i}_{pa(\delta)}}$, respectively. Then, the integral in the observational part of Eq. (3.14) can be computed as follows:

$$\int_\Theta \prod_{o \in B^{obs}_{\mathbf{i}_{pa(v)}}} p(x_v^o | \mathbf{i}_{pa(v)}, \mathbf{y}^o_{pa(v)}, \theta_v)\; p'(\theta_v|D)\; \mathrm{d}\theta_v \;=$$

$$= \int_\Theta \left(\prod_{i_\delta \in \mathcal{I}_\delta} \theta_{i_\delta|\mathbf{i}_{pa(\delta)}}^{n_{i_\delta|\mathbf{i}_{pa(\delta)}}} \right) \left(\frac{\Gamma(\alpha_{\mathbf{i}_{pa(\delta)}})}{\prod_{i_\delta} \Gamma(\alpha_{i_\delta|\mathbf{i}_{pa(\delta)}})} \prod_{i_\delta \in \mathcal{I}_\delta} \theta_{i_\delta|\mathbf{i}_{pa(\delta)}}^{\alpha_{i_\delta|\mathbf{i}_{pa(\delta)}}-1} \right) \mathrm{d}\theta_v$$

$$= \frac{\Gamma(\alpha_{\mathbf{i}_{pa(\delta)}})}{\prod_{i_\delta} \Gamma(\alpha_{i_\delta|\mathbf{i}_{pa(\delta)}})} \int_\Theta \prod_{i_\delta \in \mathcal{I}_\delta} \theta_{i_\delta|\mathbf{i}_{pa(\delta)}}^{\alpha_{i_\delta|\mathbf{i}_{pa(\delta)}}+n_{i_\delta|\mathbf{i}_{pa(\delta)}}-1} \mathrm{d}\theta_v \tag{3.15}$$

$$= \frac{\Gamma(\alpha_{\mathbf{i}_{pa(\delta)}})}{\prod_{i_\delta} \Gamma(\alpha_{i_\delta|\mathbf{i}_{pa(\delta)}})} \cdot \frac{\prod_{i_\delta} \Gamma(\alpha_{i_\delta|\mathbf{i}_{pa(\delta)}} + n_{i_\delta|\mathbf{i}_{pa(\delta)}})}{\Gamma(\alpha_{\mathbf{i}_{pa(\delta)}} + n_{\mathbf{i}_{pa(\delta)}})} \tag{3.16}$$

The first equations follow from substituting the densities of likelihood and prior into the integral. The last equation results from the fact that the Dirichlet distribution integrates to one and thus the Dirichlet integral in line (3.15) is equal to the inverse normalizing constant of Dirichlet$(\alpha_{i_\delta|\mathbf{i}_{pa(\delta)}} + n_{i_\delta|\mathbf{i}_{pa(\delta)}})$.

The formula in Eq. 3.16 describes the score constribution of a single node with fixed parent configuration. The marginal likelihood of the discrete data M_Δ can be written as the local contributions of Eq. (3.16) multiplied over all possible nodes and parent configurations, that is,

$$p_{obs}(M_\Delta \mid D) = \prod_{\delta \in \Delta} \prod_{\mathbf{i}_{pa(\delta)}} \left(\frac{\Gamma(\alpha_{\mathbf{i}_{pa(\delta)}})}{\Gamma(\alpha_{\mathbf{i}_{pa(\delta)}} + n_{\mathbf{i}_{pa(\delta)}})} \prod_{i_\delta \in \mathcal{I}_\delta} \frac{\Gamma(\alpha_{i_\delta|\mathbf{i}_{pa(\delta)}} + n_{i_\delta|\mathbf{i}_{pa(\delta)}})}{\Gamma(\alpha_{i_\delta|\mathbf{i}_{pa(\delta)}})} \right). \tag{3.17}$$

This result was first obtained by Cooper and Herskovits [23] and is further discussed by Heckerman *et al.* [58].

Discrete interventional part Since interventions are just changes in the prior, the marginal likelihood of the interventional part of discrete data is of the same form as Eq. (3.17). The prior parameters $\alpha_{i_\delta|\mathbf{i}_{pa(\delta)}}$ are exchanged by $\alpha'_{i_\delta|\mathbf{i}_{pa(\delta)}} = \mathcal{P}(\alpha_{i_\delta|\mathbf{i}_{pa(\delta)}}, w_\delta, j)$ as given by Eq. (3.3), and the counts $n_{i_\delta|\mathbf{i}_{pa(\delta)}}$ are exchanged by $n'_{i_\delta|\mathbf{i}_{pa(\delta)}}$ taken from batch $B^{int}_{\mathbf{i}_{pa(\delta)}}$.

In the limit $w_\delta \to \infty$ this part converges to one and vanishs from the overall marginal likelihood $p(M|D)$. Thus, in the limit we achieve the result of Cooper and Yoo [24] and Tian and Pearl [137].

Gaussian observational part All cases in batch $B^{obs}_{\mathbf{i}_{pa(\gamma)}}$ are sampled independently from a normal distribution with fixed parameters. If we gather them in a vector $\mathbf{y}_\gamma$ (of length $b = |B^{obs}_{\mathbf{i}_{pa(\gamma)}}|$) and the corresponding states of continuous parents as rows in a matrix $\mathbf{P}_\gamma$ (of dimension $b \times (|pa(\gamma)|+1)$), we yield the standard regression scenario

$$\mathbf{y}_\gamma \mid \beta_\gamma, \sigma^2_\gamma \sim \mathrm{N}(\mathbf{P}_\gamma \beta_\gamma, \sigma^2_\gamma \mathbf{I}), \tag{3.18}$$

where $\mathbf{I}$ is the $b \times b$ identity matrix. As a prior distribution over regression coefficients β_γ and variance σ_γ^2 we choose normal-inverse-χ^2 as shown in Eq. (3.4). Marginalizing with respect to β_γ and σ_γ^2 yields a multivariate t-distribution of dimension b, with location vector $\mathbf{P}_\gamma \mathbf{m}_\gamma$, scale matrix $s(\mathbf{I} + \mathbf{P}_\gamma \mathbf{M}_\gamma^{-1} \mathbf{P}_\gamma^\top)$, and ν_γ degrees of freedom. This can be seen by the following argument. To increase readability, we drop the index "γ" in the following equations. Then, Eq. (3.18) can be rewritten as

$$\mathbf{y} \;=\; \mathbf{P}\beta + \varepsilon \quad \text{with } \varepsilon \sim \mathrm{N}(0, \sigma^2 \mathbf{I}). \tag{3.19}$$

The prior distribution of $\beta|\sigma^2$ is Gaussian with mean $\mathbf{m}$ and variance $\sigma^2 \mathbf{M}^{-1}$. Thus we can write

$$\mathbf{P}\beta \mid \sigma^2 \;\sim\; \mathrm{N}(\mathbf{Pm}, \sigma^2 \mathbf{P}\mathbf{M}^{-1}\mathbf{P}^\top) \tag{3.20}$$

Since ε is independent of β when conditioning on σ^2 we conclude that

$$\mathbf{y} \mid \sigma^2 \;\sim\; \mathrm{N}(\mathbf{Pm}, \sigma^2(\mathbf{I} + \mathbf{P}\mathbf{M}^{-1}\mathbf{P}^\top)). \tag{3.21}$$

The prior for σ^2 is inverse-χ^2 with scale s and ν degrees of freedom. Marginalizing with respect to σ^2 yields

$$\mathbf{y} \;\sim\; t_b(\mathbf{Pm}, s(\mathbf{I} + \mathbf{P}\mathbf{M}^{-1}\mathbf{P}^\top), \nu). \tag{3.22}$$

Note that all the distribution parameters above are specific for node γ. When using data from different batches, every parameter additionally carries an index "$\mathbf{i}_{pa(\gamma)}$" indicating that it depends on the state of the discrete parents of the Gaussian node γ. Multiplying t-densities for all nodes and configurations of discrete parents—the outer double-product in Eq. (3.14)—yields the marginal likelihood of the Gaussian part.

Gaussian interventional part Here we consider cases in batch $B^{int}_{\mathbf{i}_{pa(\gamma)}}$. We collect them in a vector and can again write a regression model like in Eq. (3.18). The difference to the observational Gaussian case lies in the prior parameters. They are now given by Eq. (3.5). The result of marginalization is again a t-density with parameters as above. The only difference is that the pair $(\mathbf{m}, s)$ is exchanged by $(\mathbf{m}', s') = \mathcal{P}((\mathbf{m}, s), w_\gamma, k)$. The Gaussian interventional part is then given by a product of such t-densities over nodes and discrete parent configurations.

If we use the hard intervention prior in Eq. (3.7) instead, the Gaussian interventional part integrates to one and vanishs from the marginal likelihood in Eq. (3.14). Thus, we extended the results by Cooper and Yoo [24] to Gaussian networks.

3.4.3 Probabilistic soft interventions

In Section 3.3 we introduced the pushing operator $\mathcal{P}(\cdot, w_v, t_v)$ to model a soft intervention at a discrete or Gaussian node v. The intervention strength w_v is a parameter, which has to be chosen before network learning. There are several possibilities, how to do it. If there is solid experimental experience on how powerful interventions are,

this can be reflected in an appropriate choice of w_v. An obvious problem is that w_v needs to be determined on a scale that is compatible with the Bayesian network model. If there is prior knowledge on parts of the network topology, the parameter w_v can be tuned until the result of network learning fits the prior knowledge. Note again that by the parametrization of pushing given in Section 3.3, the pushing strengths for discrete and Gaussian nodes live on different scales and have to be calibrated separately.

However, a closer inspection of the biological background in chapter 1, which motivated the theory of soft pushing interventions, suggests to treat the intervention strength w_v as a random variable. In gene silencing an inhibiting molecule (a double-stranded RNA in case of RNAi) is introduced into the cell. This usually works in a high percentage of affected cells. In the case of success, the inhibitor still has to spread throughout the cell to silence the target gene. This diffusion process is stochastic and consequently causes experimental variance in the strength of the silencing effect.

These observations suggest to assign a prior distribution $p(w_v)$ to the intervention strength. That is, we drop the assumption of having one intervention strength in all cases, but instead average over possible values of w_v. For simplicity we assume there is only a limited number of possible values of w_v, say, $w_v^{(1)}, \dots, w_v^{(k)}$, with an arbitrary discrete distribution assigned to them. Then we can express our inability to control the pushing strength in the experiment deterministically by using a mixed prior of the form

$$p(\theta_v|D) = \sum_{i=1}^{k} q_k \, p(\theta_v|D, w_v^{(k)}). \tag{3.23}$$

Here, the mixture coefficients $q_k = p(w_v^{(k)})$ are the prior probabilities of each possible pushing strength. The terms $p(\theta_v|D, w_v^{(k)})$ correspond to Dirichlet densities in the discrete case and Normal-inverse-χ^2 densities in the Gaussian case. In RNAi experiments, $w_v^{(1)}, \dots, w_v^{(k)}$ can be estimated from the empirical distribution of measured RNA degradation efficiencies in repeated assays. Mixed priors as in Eq. (3.23) are often used in biological sequence analysis to express prior knowledge which is not easily forced into a single distribution. See Durbin *et al.* [34] for details. If we substitute the prior $p''(\theta_v|D, w_v)$ in the interventional part of Eq. (3.14) with the mixture prior in Eq. (3.23), the marginal likelihood of a family of nodes is a mixture of marginal likelihoods corresponding to certain values $w_v^{(k)}$ weighted by mixture coefficients q_k.

Discussion

Our work extends structure learning from interventional data into two directions: from learning discrete networks to learning mixed networks and from learning with hard interventions to learning with soft interventions. Soft interventions are focussed on a specific target value of the variable of interest and concentrate the local probability distribution there. We proposed parametrizations for pushing discrete and

continuous variables using Dirichlet and Normal-inverse-χ^2 priors, respectively. We computed the marginal likelihood of CG networks for data containing both observational and (soft) interventional cases. In Bayesian structure learning, the marginal likelihood is the key quantity to compute from data. Using it (and possibly a prior over network structures) as a scoring function, we can start model search over possible network structures. For a survey of search heuristics see section 2.3.4.

Since in biological settings the pushing strength is unknown, we proposed using a mixture prior on it, resulting in a mixture marginal likelihood. This makes the score for each network more time-consuming to compute. But in applications there is often a large amount of biological prior knowledge, which limits the number of pathway candidates from the beginning. When learning network structure we usually don't have to optimize the score over the space of all possible DAGs but are limited to a few candidate networks, which are to be compared. This corresponds to a very rigid structure prior.

Modeling interventions as soft pushing makes structure learning more robust against noise. Soft interventions handle major sources of noise inherent in real biological systems. This is a central benefit of our approach.

Beyond transcriptional networks At the end of chapter 2 we found that visualizing the correlation structure of gene expression may not give us a biologically meaningful answer. As a first reason for this shortcoming we discussed the need for interventional data. To address this issue, the present chapter introduced a novel model of interventions in Bayesian networks. But there is also a second reason, why a visualization of correlation structure on expression data may not give us the full picture. We need to have a second look at the rationale, which made us use graphical models in the first place.

The application of graphical models is motivated by the following consideration: if the expression of gene A is regulated by proteins B and C, then A's expression level is a stochastic function of the joint activity levels of B and C. Expression levels of genes are taken as a proxy for the activity level of the proteins they encode. This is the rationale leading to the application of Bayesian networks to expression data [41]. It relies on the assumption that both the regulator and its targets must be transcriptionally regulated, resulting in detectable changes in their expression. Indeed, recent large-scale analyses of the regulatory networks of *Escherichia coli* [121] and *S. cerevisiae* [74, 86] found a number of cases in which the regulators are themselves transcriptionally regulated. Simon *et al.* [123] show direct dependencies of cell cycle transcriptional regulators in yeast between different cell cycle stages. Regulators that function during one stage of the cell cycle contribute to the regulation of transcriptional activators active in the next stage. These studies show the importance of transcriptional regulation in controlling gene expression.

On the other hand, these observations cannot obscure the fact that models of correlation structure of mRNA levels have only limited explanatory value, as can be seen by the two following studies. Gygi *et al.* [54] found that correlation between mRNA

and protein levels was poor in yeast. Quantitative mRNA data was insufficient to predict protein expression levels. They found cases where the protein levels varied by more than 20-fold, even if the mRNA levels stayed the same. Additionally, activation or silencing of a regulator is in most cases carried out by posttranscriptional protein modifications [71]. Thus, even knowing the correct expression state is not enough, we also need to know the activation state of the protein. In summary, activation levels of proteins cannot be approximated well by expression levels of corresponding genes. However, the next chapter will show that the situation is not hopeless. We will show that secondary effects of interventions are visible as expression changes on microarray data. Transcriptional effects allow to infer regulatory hierarchies in non-transcriptional parts of a pathway.

Chapter 4

Inferring signal transduction pathways

The last chapter dealt with models of primary effects. We assumed that perturbing one pathway component leads to detectable changes at other pathway components. In this chapter I introduce a method designed for indirect observations of pathway activity by secondary effects at downstream genes (section 4.1). I present an algorithm to infer non-transcriptional pathway features based on differential gene expression in silencing assays. The main contribution is a score linking models to data (section 4.2). I demonstrate its power in the controlled setting of simulation studies (section 4.3) and explain its practical use in the context of an RNAi data set investigating the response to microbial challenge in Drosophila melanogaster *(section 4.4).*

4.1 Non-transcriptional modules in signaling pathways

A cell's response to an external stimulus is complex. The stimulus is propagated via signal transduction to activate transcription factors, which bind to promoters thus activating or repressing the transcription and translation of genes, which in turn can activate secondary signaling pathways, and so on. We distinguish between the transcriptional level of signal transduction known as gene regulation and the non-transcriptional level, which is mostly mediated by post-translational modifications. While gene regulation leaves direct traces on expression profiles, non-transcriptional signaling does not. Thus, on microarray data gene regulatory networks can be modelled by methods described in chapters 2 and 3, while non-transcriptional pathways can not. However, reflections of signaling activity can be perceived in expression levels of other genes. We explain this in a simplified pathway model and in a real world example in *Drosophila*.

A hypothetical pathway Fig. 4.1 shows a hypothetical biochemical pathway adapted from Wagner [140]. It consists of two transcription factors, a protein kinase and a protein phosphatase and the genes encoding these proteins. The figure

shows the three biological levels of interest: genome, transcriptome and proteome. The thick arrows show information flow through the pathway. The transcription factor expressed by gene *1* binds to the promoter region of gene *2* and activates it. Gene *2* encodes a protein kinase, which phosphorylates a protein phosphatase (expressed by gene *3*). This event activates the protein phosphatase, which now dephosphorylates the transcription factor produced by gene *4*. It binds to gene *5* and induces expression.

The three biological levels of DNA, mRNA and protein are condensed into a graph model on five nodes. Gene expression data only shows the mRNA level. A model inferred from expression data will only have two edges, connecting gene *1* to gene *2* and then gene *2* to gene *5*. Since genes *3* and *4* only contribute on the protein level, a model based on correlations on the mRNA level will ignore them. This holds true for all models descibed in chapter 2.

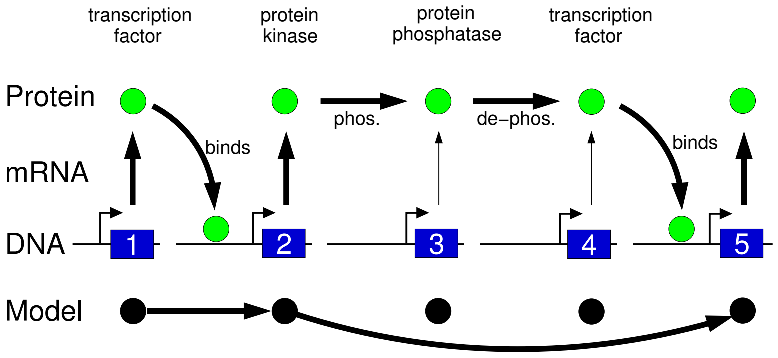

Figure 4.1: *A hypothetical biochemical pathway adapted from Wagner [140]. It shows four levels of interest: three biological and one of modeling. Inference from gene expression data alone only gives a very limited model of the pathway. The contributions of genes* 3 *and* 4 *are overlooked.*

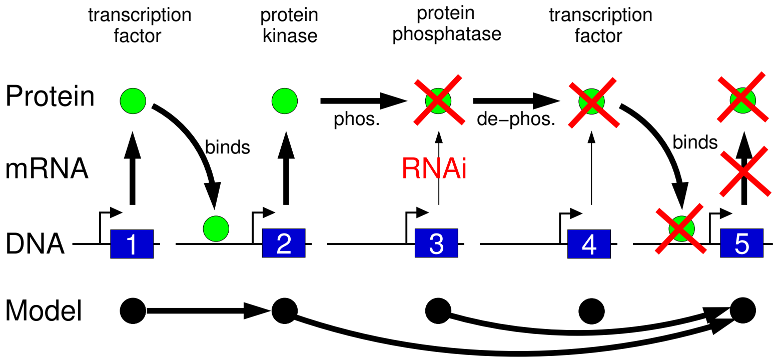

Figure 4.2: *The situation changes if we can use interventional data for model building. Silencing gene* 3 *by RNAi will cut information flow in the pathway and result in an expression change at gene* 5*. This is visible on the mRNA level and can be integrated in the model. Thus, the expanded model shows an edge from gene* 3 *to gene* 5*.*

Interventions at genes in the pathway shed light on the pathway topology. This is exemplified by an RNAi intervention at gene *3* in Fig. 4.2. Silencing gene *3* will cut information flow in the pathway and result in an expression change at gene *5*. This is reflected in the model by extending it to include an edge from gene *3* to gene *5*. Note that we have no observation of direct effects of the intervention at gene *4* in mRNA data. The only information we have are secondary effects at the transcriptional end of the pathway. This chapter will introduce novel methodology to order genes in regulatory hierarchies from secondary effects. The procedure is motivated by the logic underlying a study in *Drosophila* conducted by Michael Boutros and coworkers.

An example in Drosophila Boutros *et al.* [12] investigate the response to microbial challenge in *Drosophila melanogaster*. They treat *Drosophila* cells with lipopolysaccharides (LPS), the principal cell wall components of gram-negative bacteria. Sixty minutes after applying LPS, a number of genes show a strong reaction. Which genes and gene products were involved in propagating the signal in the cell? To answer this question a number of signaling genes are silenced by RNAi. The effects on the LPS-induced genes are measured by microarrays. The observations are: with only one exception, the signaling genes show no change in expression when other signaling genes are silenced. They stay "flat" on the microarrays. Differential expression is only observed in genes downstream of the signaling pathway: silencing *tak* reduces expression of all LPS-inducible transcripts, silencing *rel* or *mkk4/hep* reduces expression of disjoint subsets of induced transcripts, silencing *key* results in profiles similar to silencing *rel*. Gene *tak* codes for protein TAK1 in Fig. 1.2, *key* for IKKγ, and *rel* is the transcription factor Relish, already discussed in the introduction in chapter 1.

Boutros *et al.* [12] explain this observation by a fork in the signaling pathway with *tak* above the fork, *mkk4/hep* in one branch, and both *key* and *rel* in the other branch. The interpretation is a Relish-independent response to LPS, which is also triggered by IMD and TAK but then branches off the Imd pathway. Note that this pathway topology was found in an indirect way: no information is coming from the expression levels of the signaling genes. Silencing candidate genes interrupts the information flow in the pathway, the topology is then revealed by the nested structure of affected gene sets downstream the pathway of interest. The computational challenge we address is to derive an algorithm for systematic inference from indirect observations.

Models for primary effects cannot be applied here In chapter 3, we discussed models to explain primary effects of silencing genes on other genes in the pathway. Some are deterministic and graph based, some are probabilistic and able to handle noise in the data. All of them aim for transcriptional networks and are unable to capture non-transcriptional modulation. Some approaches use hidden variables to capture non-transcriptional effects [89, 104, 105] without making use of interventional data. To keep model selection feasible they have to introduce a number of simplifying assumptions: either the hidden nodes do not regulate each other, or the hidden structure is not identifiable. In both cases, the models do not allow inference of non-transcriptional pathways. In graphical models with hidden variables non-transcriptional effects are considered nuisance, not the main target of pathway

reconstruction. In summary, none of the methods designed to infer transcriptional networks can be applied to reconstruct non-transcriptional pathways from microarray data. The major problem is: these algorithms require direct observations of expression changes of signaling genes, which are not fully available in datasets like that of [12]. There exist only two methodologies comparable to ours in being able to identify non-transcriptional pathway features from microarray data: *physical network models* and *epistasis analysis*.

Physical network models Yeang *et al.* [149] introduce a maximum likelihood based approach to combine three different yeast datasets: protein–DNA, protein–protein, and single gene knock-out data. The first two data sources indicate direct interactions, while the knock-out data only contains indirect functional information. The algorithm searches for topologies which are consistent with observed downstream effects of interventions. While it is not confined to the transcriptional level of regulation, it also requires that most signaling genes show effects when perturbing others. It is not designed for a dataset like that of Boutros *et al.* [12] described above.

Epistasis analysis Our general objective is similar to epistasis analysis with global transcriptional phenotypes. Regulatory hierarchies can be identified by comparing single-knockout phenotypes to double-knockout phenotypes. Driessche *et al.* [31] use gene expression time-courses as phenotypes and reconstruct a regulatory system in the development of *Dictyostelium discoideum*, a soil-living amoeba. Yet, there are several important differences. First, we model whole pathways and not only single gene-gene interactions. Second, we treat an expression profile not as one global phenotype but as a collection of single-gene phenotypes. This will be made clear in the following overview.

How to learn from secondary effects We present a computational framework for the systematic reconstruction of pathway features from expression profiles relating to external interventions. The approach is based on the nested structure of affected downstream genes, which are themselves not part of the model. Here we give a short overview of the method before presenting it in all details in section 4.2. The model distinguishes two kinds of genes: the candidate pathway genes, which are silenced by RNAi, and the genes, which show effects of such interventions in expression profiles. We call the first ones *S-genes* (S for "silenced" or "signaling") and the second ones *E-genes* (E for "effects"). Because large parts of signaling pathways are non-transcriptional, there will be little or no overlap between S-genes and E-genes. Elucidating relationships between S-genes is the focus of our analysis, the E-genes are only needed as reporters for signal flow in the pathway. E-genes can be considered as transcriptional phenotypes. S-genes have to be chosen depending on the specific question and pathway of interest. E-genes are identified by comparing measurements of the stimulated and non-stimulated pathway: genes with a high expression change are taken as E-genes.

The basic idea is to model how interventions interrupt the information flow through the pathway. Thus, S-genes are silenced while the pathway is stimulated to see which E-genes are still reached by the signal. Optimally, the gene expression experiments are

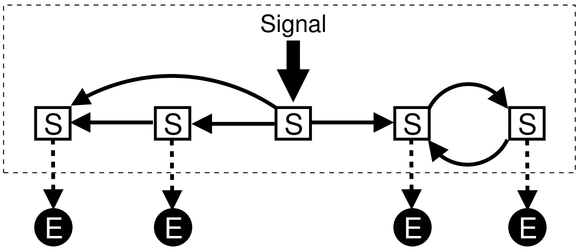

Figure 4.3: *A schematic summary of our model. The dashed box indicates one hypothesis: it contains a directed graph T on genes contributing to a signaling pathway (S-genes). A signal enters the pathway at one (or possibly more than one) specified position. Interventions at S-genes interrupt signal flow through the pathway. S-genes regulate E-genes on the second level. Together the S- and E-genes form an extended topology T'. We observe noisy measurements of expression changes of E-genes. The objective is to reconstruct relationships between S-genes from observations of E-genes in silencing experiments.*

replicated several times. This results in a data set representing every signaling gene by one or more microarrays. These requirements are the same as in epistasis analysis [6], but they are not satisfied in all datasets monitoring intervention effects. In the Rosetta yeast compendium [61], for example, there is no external stimulus by which the interruption of signal flow through a pathway of interest could be measured.

The main contribution of this chapter is a scoring function, which measures how well hypotheses about pathway topology are supported by experimental data. *Input* to the algorithm is a list of hypotheses about the candidate pathway genes. A hypothesis is characterized by (1.) a directed graph with S-genes as nodes and (2.) the possibly many entry points of signal into the pathway. This setting is summarized in Fig. 4.3. The model is based on the expected response of an intervention given a candidate topology of S-genes and the position of the intervention in the topology. Pathways with different topology can show the same downstream response to interventions. All pathways, which make the same predictions of intervention effects on downstream genes, are identified by one so called *silencing scheme*. Sorting silencing schemes by our scoring function shows how well candidate pathways agree with experimental data. *Output* of the algorithm is a strongly reduced list of candidate pathways. The algorithm is a filter, which helps to direct further research.

Applications beyond RNAi Our motivation to develop this algorithm results from the novel challenges the RNAi technology poses to bioinformatics. At present RNAi appears to be the most efficient technology for producing large-scale gene-intervention data. However, our framework is flexible and any type of external interventions can be used, which reduces information flow in the pathway. This includes traditional knock-out experiments and specific protein inhibiting drugs. An important requirement for any perturbation technique used is high specificity. Off-target effects impair our method since intervention effects can no longer be uniquely predicted.

4.2 Gene silencing with transcriptional phenotypes

First, we describe our model for signaling pathways with transcriptional phenotypes. Predictions from pathway hypotheses are summarized in a silencing scheme. In the main part of the section, we develop a Bayesian method to estimate a silencing scheme from data.

4.2.1 Signaling pathway model

Core topology on S-genes The set of E-genes is denoted by $\mathbf{E} = \{E_1, \ldots, E_m\}$, and the set of S-genes by $\mathbf{S} = \{S_1, \ldots, S_p\}$. As a pathway model, we assume a directed graph T on vertex set $\mathbf{S}$. The structure of T is not further restricted: there may be cycles and it may decompose into several subgraphs. The external stimulus acts on one or more of the S-genes as specified by the hypothesis. S-genes can take values `1` and `0` according to whether signaling is interrupted or not. State `0` corresponds to a node, which is reached by the information flow through the pathway. This is the natural state when the pathway is stimulated. State `1` describes a node, which is no longer reached by the signal, because the flow of information is cut by an intervention at some node upstream in the pathway. An S-gene in state `1` is in the same state as if the pathway had not been stimulated. While the pathway is stimulated, experimental interventions break the information flow in the pathway. An intervention at a particular S-gene first puts this S-gene's state to `1`. The silencing effect is then propagated along the directed edges of T.

From pathways to silencing schemes We call the subset of S-genes, which are in state `1` when S-gene S is silenced, the *influence region of* S. The set of all influence regions is called a *silencing scheme* Φ. It summarizes the effects of interventions predicted from the pathway hypothesis. Mathematically, a silencing scheme is the transitive closure of pathway T implying a partial order on $\mathbf{S}$. Drawn as a graph, Φ contains an edge between two nodes whenever they are connected by a directed path in T. Different pathway models can result in the same silencing scheme. An example is given in Fig. 4.4. Note that the E-genes do not appear in Φ, which only describes interactions between S-genes. The E-genes come into play when inferring silencing schemes. Reduced signaling strength of S-genes due to interventions in the pathway cannot be observed directly on a microarray, but secondary effects are visible on E-genes.

Secondary effects on E-genes The extended topology on $\mathbf{S} \cup \mathbf{E}$ is called T'. We assume that each E-gene has a single parent in $\mathbf{S}$. In particular, the E-genes do not interact with each other. We interpret the set of E-genes attached to one S-gene as a regulatory module, which is under the common control of the S-gene. The reaction of E-genes to interventions in the pathway depends on where the parent S-gene is located in the silencing scheme. E-genes are set to state `1` if their parent S-gene is in the influence region of an intervention; else they are in state `0`. The state of E-genes

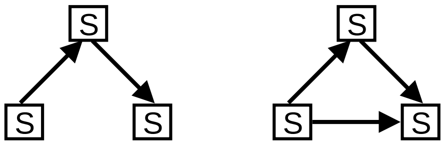

Figure 4.4: *Transitive closure. The right topology is the transitive closure of the left topology. When adding an entry point for signal, both are valid pathway hypotheses. Both are represented by a silencing scheme, which has the same topology as the right graph.*

can be experimentally observed as differential expression on microarrays. Due to the observational noise or stochastic effects in signal transduction, we expect a number of false positive and false negative observations.

4.2.2 Likelihood of a silencing scheme

Data In each experiment, one S-gene is silenced by RNAi and effects on E-genes are measured by microarrays. Each S-gene needs to be silenced at least once, but ideally the silencing assays are repeated and several microarrays per silenced gene are included in the dataset. Microarrays are indexed by $k = 1, \ldots, l$. The expression data are assumed to be discretized to `1` and `0` — indicating whether interruption of signal flow was observed at a particular gene or not. The result is a binary matrix $M = (e_{ik})$, where $e_{ik} = 1$ if E-gene E_i shows an effect in experiment k. Thus, our data only consists in coarse qualitative information. We do not consider whether an E-gene was up- or down-regulated or how strong an effect was. Each single observation e_{ik} relates the intervention done in experiment k to the state of E_i. In the following, the index "i" always refers to an E-gene, the index "j" to an S-gene, and the index "k" to an experiment.

Likelihood The positions of E-genes are included as model parameters $\Theta = \{\theta_i\}_{i=1}^m$ with $\theta_i \in \{1, \ldots, n\}$ and $\theta_i = j$ if E_i is attached to S_j. Let us first consider a fixed extension T' of T, that is, the parameters Θ are assumed to be known. For each E-gene, T' encodes to which S-gene it is connected. In a silencing experiment T' predicts effects at all E-genes, which are attached to an S-gene in the influence region. Expected effects can be compared to observed effects in the data to choose the topology, which fits the data best. Due to measurement noise no topology T' is expected to be in complete agreement with all observations. Deviations from predicted effects are allowed by introducing global error probabilities α and β for false positive and negative calls, respectively.

The expression levels of E-genes on the various microarrays are modelled as binary random variables E_{ik}. The distribution of E_{ik} is determined by the silencing scheme Φ and the error probabilities α and β. For all E-genes and targets of intervention, the conditional probability of E-gene state e_{ik} given silencing scheme Φ can then be

written in tabular form as

$$p(e_{ik}|\Phi,\theta_i=j)=\begin{cases} & \begin{array}{cc} e_{ik}=1 & e_{ik}=0 \\ \hline \alpha & 1-\alpha \\ 1-\beta & \beta \end{array} \begin{array}{l} \\ \text{if } S_j=0 \\ \text{if } S_j=1 \end{array}\end{cases} \tag{4.1}$$

This means: if the parent of E_i is not in the influence region of the S-gene silenced in experiment k, the probability of observing $E_{ik}=1$ is α (probability of false alarm, type-I error). The probability to miss an effect and observe $E_{ik}=0$ even though E_i lies in the influence region is β (type-II error). The likelihood $p(M|\Phi,\Theta)$ of the data is then a product of terms from the table for every observation, that is,

$$p(M|\Phi,\Theta) \;=\; \prod_{i=1}^{m}\prod_{k=1}^{l} p(e_{ik}|\Phi,\theta_i) \;=\; \alpha^{n_{10}}\beta^{n_{01}}(1-\alpha)^{n_{00}}(1-\beta)^{n_{11}}, \tag{4.2}$$

where n_{se} is the number of times we observed E-genes in state e when their parent S-gene in Φ was in state s.

However, in reality the "correct" extension T' of a candidate topology T is unknown. The positions of E-genes are unknown and they may be regulated by more than one S-gene. We also do not aim to infer extended topologies from the data: the model space of extended topologies is huge, and model inference is unstable. We are only interested in the silencing scheme Φ of S-genes. To deal with these issues, we interpret the position of edges between S- and E-genes as *nuisance parameters*, and average over them to obtain a marginal likelihood. This is described next.

4.2.3 Marginal likelihood of a silencing scheme

This section defines a scoring function to link models with observations. It evaluates how well a given silencing scheme Φ fits the experimental data. For now, we assume the silencing scheme Φ and the error probabilities α and β to be fixed. But in contrast to the last section, the position parameters Θ are unknown. By Bayes' formula the posterior of silencing scheme Φ given data M can be written as

$$p(\Phi|M)=\frac{p(M|\Phi)p(\Phi)}{p(M)}. \tag{4.3}$$

The normalizing constant $p(M)$ is the same for all silencing schemes, it can be neglected for relative model comparison. The model prior $p(\Phi)$ can be chosen to incorporate biological prior knowledge. In the following, we assume it to be uniform over all possible models. What remains is the marginal likelihood $p(M|\Phi)$. It equals the likelihood $p(M|\Phi,\Theta)$ averaged over the nuisance parameters Θ. To compute it, we make three assumptions:

1. Given silencing scheme Φ and fixed positions of E-genes Θ, the observations in M are sampled independently and distributed identically:
$$p(M|\Phi,\Theta) = \prod_{i=1}^{m} p(M_i|\Phi,\theta_i) = \prod_{i=1}^{m}\prod_{k=1}^{l} p(e_{ik}|\Phi,\theta_i),$$
where M_i is the ith row in data matrix M.
2. Parameter independence. The position of one E-gene is independent of the positions of all the other E-genes:
$$p(\Theta|\Phi) = \prod_{i=1}^{m} p(\theta_i|\Phi).$$
3. Uniform prior distribution. The prior probability to attach an E-gene is uniform over all S-genes:
$$P(\theta_i = j|\Phi) = \frac{1}{p} \quad \text{for all } i \text{ and } j.$$

The last assumption can easily be dropped to include existing biological prior knowledge about regulatory modules. With the assumptions above, the marginal likelihood can be calculated as follows. The numbers above the equality sign indicate which assumption was used in each step.

$$\begin{aligned}
p_{\alpha,\beta}(M|\Phi) &= \int p_{\alpha,\beta}(M|\Phi,\Theta)\ p(\Theta|\Phi)\ \mathrm{d}\Theta \\
&\overset{[1,2]}{=} \prod_{i=1}^{m} \int p_{\alpha,\beta}(M_i|\Phi,\theta_i)\ p(\theta_i|\Phi)\ \mathrm{d}\theta_i \\
&\overset{[3]}{=} \frac{1}{p^m}\prod_{i=1}^{m}\sum_{j=1}^{p} p_{\alpha,\beta}(M_i|\Phi,\theta_i=j) \\
&\overset{[1]}{=} \frac{1}{p^m}\prod_{i=1}^{m}\sum_{j=1}^{p}\prod_{k=1}^{l} p_{\alpha,\beta}(e_{ik}|\Phi,\theta_i=j). \qquad (4.4)
\end{aligned}$$

The marginal likelihood in Eq. (4.4) contains the error probabilities α and β as free parameters to be chosen by the user. This is indicated by subscripts. In section 4.4 we will show how to estimate these parameters when discretizing the data.

Estimated position of E-genes Given a silencing scheme Φ, the posterior probability for an edge between S_j and E_i is given by

$$P_{\alpha,\beta}(\theta_i = j|\Phi,M) = \frac{p(\theta_i = j \mid \Phi)}{p_{\alpha,\beta}(M_i \mid \Phi)} \prod_{k=1}^{l} p_{\alpha,\beta}(e_{ik} \mid \Phi,\theta_i = j) \qquad (4.5)$$

where the prior $p(\theta_i = j|\Phi)$ is again chosen to be uniform. In general, the prior could take any other form as long as it is the same as in the computation of marginal likelihood above. The E-genes attached with high probabilty to an S-gene are interpreted as a regulatory module, which is under the common control of the S-gene.

4.2.4 Averaging over error probabilities α and β

The likelihood in Eq. (4.4) is a polynomial in α and β. In a full Bayesian approach we would again average over possible values of α and β given a prior distribution. This problem can be cast in a way accessible to standard Bayesian theory, as it is also used when averaging over LPD parameters to gain the marginal likelihood in Bayesian network structure learning (see section 2.12). So far, we assumed that all E-genes share the distribution specified in Eq. (4.1) and α and β are indeed global parameters applicable to every E-gene. This simplifying assumption was introduced to keep inference feasible. Else, we would have to estimate parameters (α_i, β_i) for every E-gene E_i. When averaging over LPD parameters, we will drop the assumption of parameter sharing. Instead we augment the three assumptions above by three additional ones.

First we define $\eta_i = (\eta_{i0}, \eta_{i1}) = (\alpha_i, 1 - \beta_i)$, then for one E-gene E with parent S holds $\eta_{is} = P(E_i = 1|S_{\theta_i} = s)$. We make the following assumptions on the prior distribution $p(\eta|\Phi, \Theta)$ of $\eta = (\eta_i)_{i=1,\dots,m}$:

4. Global and local parameter independence. Parameters are independent for every E-gene E_i and for different states of the parent S-gene, that is,

$$p(\eta|\Phi,\Theta) \;=\; \prod_{i=1}^{m} p(\eta_i|\Phi,\theta_i) \;=\; \prod_{i=1}^{m}\prod_{s\in\{0,1\}} p(\eta_{is}|\Phi,\theta_i).$$

5. The prior $p(\eta_{is}|\Phi, \theta_i)$ is chosen as a beta distribution, which is conjugate to the multinomial distribution of the E_i [49], that is,

$$p(\eta_{is}|\Phi,\theta_i) \;=\; \eta_{is}^{a_{is}-1}(1-\eta_{is})^{b_{is}-1}.$$

6. All local priors $p(\eta_{is}|\Phi, \theta_i)$ share the same parameters, that is,

$$a_{is} = a_s \quad \text{and} \quad b_{is} = b_s \quad \text{for all } i = 1,\dots,m.$$

The last assumption limits the number of parameters. It is parameter sharing not on the level of distribution parameters but on the level of parameters of prior distributions, which are themselves independent. With these assumptions we can compute the marginal likelihood with respect to position parameters Θ and effect probabilities η by

$$\begin{aligned} p(M|\Phi) \;&=\; \iint p(M|\Phi,\Theta,\eta)\; p(\eta|\Phi,\Theta)\; p(\Theta|\Phi)\; \mathrm{d}\eta\; \mathrm{d}\theta \\ &\overset{[4]}{=}\; \prod_{i=1}^{m}\int\left(\int p(M_i|\Phi,\theta_i,\eta_i)\; p(\eta_i|\Phi,\theta_i)\; \mathrm{d}\eta_i\right) p(\theta_i|\Phi)\; \mathrm{d}\theta_i. \qquad (4.6)\end{aligned}$$

We first concentrate on one fixed E_i. Then Φ and θ_i specify the parent S-gene S_{θ_i} and its state $S_{\theta_i} = s$. The data M_i split into two subsets M_i^s and M_i^{1-s}, where

$M_i^s = \{e_{ik} | S_{\theta_i} = s\}$. Each batch of data follows the same binomial distribution in Eq. (4.1). The inner integral in Eq. (4.6) splits into two integrals, one for each parent state s, which can be computed as follows:

$$\begin{aligned}\int p(M_i^s|\Phi, \theta_i, \eta_{is}) p(\eta_{is}|\Phi, \theta_i)\, \mathrm{d}\eta_{is} &= \\ &\overset{[5,6]}{=} \frac{\Gamma(a_s + b_s)}{\Gamma(a_s)\Gamma(b_s)} \int \eta_{is}^{n_{is1}+a_s-1} (1-\eta_{is})^{n_{is0}+b_s-1}\, \mathrm{d}\eta_{is} \\ &= \frac{\Gamma(a_s + b_s)}{\Gamma(a_s)\Gamma(b_s)} \cdot \frac{\Gamma(n_{is1} + a_s)\Gamma(n_{is0} + b_s)}{\Gamma(n_{is1} + n_{is0} + a_s + b_s)}, \end{aligned} \tag{4.7}$$

where the counts n_{ise} denote the number of experiments, in which we observed $E_i = e$ while the parent S-gene S_{θ_i} was in state s. Note that this computation is identical to marginalizing LPD parameters in discrete Bayesian networks (section 3.4.2). The reason is that our model can be viewed as a highly restricted Bayesian network, in which the LPDs at S-genes are deterministic and the E-genes follow a conditional binomial distribution.

The data likelihood $p(M_i|\Phi, \theta_i)$ for gene E_i is a product of terms on the right hand side of Eq. (4.7) for both S-gene states. The marginalization over E-gene positions Θ works exactely as in section 4.2.3 and results in the following full marginal likelihood:

$$p(D|\Phi) = \frac{1}{p^m} \prod_{i=1}^{m} \sum_{j=1}^{p} \prod_{s\in\{0,1\}} \frac{\Gamma(a_s + b_s)\Gamma(n_{is1} + a_s)\Gamma(n_{is0} + b_s)}{\Gamma(a_s)\Gamma(b_s)\Gamma(n_{is1} + n_{is0} + a_s + b_s)}. \tag{4.8}$$

Estimated position of E-genes Similar to Eq. (4.5), the posterior probability for an edge between S_j and E_i with marginalization over α and β is given by

$$\begin{aligned} P(\theta_i = j|\Phi, M) &= \frac{1}{Z} \prod_{k=1}^{l} p(e_{ik} \mid \Phi, \theta_i = j) \\ &= \frac{1}{Z} \prod_{s\in\{0,1\}} \frac{\Gamma(a_s + b_s)\Gamma(n_{is1} + a_s)\Gamma(n_{is0} + b_s)}{\Gamma(a_s)\Gamma(b_s)\Gamma(n_{is1} + n_{is0} + a_s + b_s)}. \end{aligned} \tag{4.9}$$

where Z is a normalizing constant ensuring that the sum over all S-genes is 1. This equation allows to estimate E-gene positions given the beta prior on the local distribution parameters of E_i.

Summary of parameters Table 4.1 gives an overview of the ingredients to the formulas developed in this section. It shows counts, distribution parameters and prior parameters for the four possible combinations of E-gene state and parent S-gene state. The counts are E-gene specific, while the parameters (α, β) and prior parameters (a_0, b_0, a_1, b_1) apply to all E-genes. Having four prior parameters to specify, while before there were only two distribution parameters, may seem as a disadvantage of marginalization. But there are two considerations to keep in mind. First, a model is much more stable against choices of prior parameters than of distribution parameters.

	E_i			Eq. (4.4)			Eq. (4.8)	
	1	0		1	0		1	0
S 0	n_{i01}	n_{i00}	0	α	$1-\alpha$	0	a_o	b_0
1	n_{i11}	n_{i10}	1	$1-\beta$	β	1	a_1	b_1

Table 4.1: *The table describes the main terms of the marginal likelihoods computed in this section. It focusses on one E-gene (columns) and its parent S-gene (rows). The left table contains the counts from the data for the four possible combinations of E-gene and parent state. They are E-gene specific and used in all formulas. To compute the marginal likelihood of Eq. (4.4) error probabilities α and β need to be specified, which are the same for all E-genes. For the full marginal likelihood of Eq. (4.8) the user needs to choose prior parameters (a_0, b_0) and (a_1, b_1), which are shared by all E-genes.*

In situations with little knowledge on error rates in experiments it is safer to use the full marginal likelihood of Eq. (4.8) than the marginal likelihood of Eq. (4.4). Second, the four prior parameters fall in two categories: (a_0, b_1) give weights for observing errors, while (a_1, b_0) give weights for observing the predicted state. This motivates to use only two values for the prior parameters: one for a_0 and b_1, and another one for a_1 and b_0. Because we expect there to be more signal than noise in the data, the value of $a_0 = b_1$ should be considerably smaller than that of $a_1 = b_0$. We will see an example in the application to *Drosophila* data in section 4.4.

4.2.5 Limits of learning from secondary effects

The method we described can only reconstruct features of the pathway, not the full topology. This stems from inherent limits of reconstruction from indirect observations. We discuss here *prediction equivalence* and *data equivalence*.

Prediction equivalence More than one pathway hypothesis result in the same silencing scheme if they only differ in transitive edges. An example is given in Fig. 4.4. Both topologies there can be considered as pathway hypotheses, but only the right one is transitively closed and thus a silencing scheme. Since our score is defined on silencing schemes and not on topologies directly, the hypotheses with the same silencing scheme are not distinguishable. Assuming parsimony, each silencing scheme can uniquely be represented by a graph with minimal number of edges. This technique is called *transitive reduction* [1, 75, 142, 140].

Data equivalence There exist cases, where two hypotheses with different silencing schemes produce identical data. Fig. 4.5 shows an example with a cycle of S-genes and a linear cascade, where all E-genes are attached at the downstream end. In both pathways, all E-genes react to interventions at every S-gene. In this case, the data does not prefer one silencing scheme over the other.

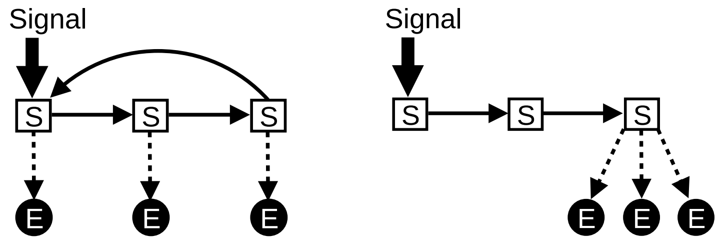

Figure 4.5: *Data equivalence: The two plots show different topologies of S-genes with two distinct silencing schemes. However, both pathways will produce the same data: All E-genes react to interventions at every S-gene.*

4.2.6 Extending the basic model

Epistatic effects The model described above is very simple. Additional constraints are imposed by epistatic effects: one gene can mask the effect of another gene. These effects can be included into the model by introducing a set of boolean functions $F = \{f_S, S \in \mathbf{S}\}$. Each $f_S \in F$ determines the state of S-gene S given the states of its parents in T. Two simple examples of local functions f_S are AND- and OR-logics. In an AND-logic, all parent nodes must be affected by an intervention (*i.e.* have state 1) to propagate the silencing effect to the child. This describes redundancy in the pathway: if two genes fulfill alternative functions, both have to be silenced to stop signal flow through the pathway. In an OR-logic, one affected parent node is enough to set the child's state to 1. This describes a set of genes jointly regulating the child node; silencing one of the parents destroys the collaboration. The topology T together with the set of functions F defines a deterministic Boolean network on $\mathbf{S}$. Fig. 4.6 gives an example, how local logics constrain influence regions and change silencing schemes.

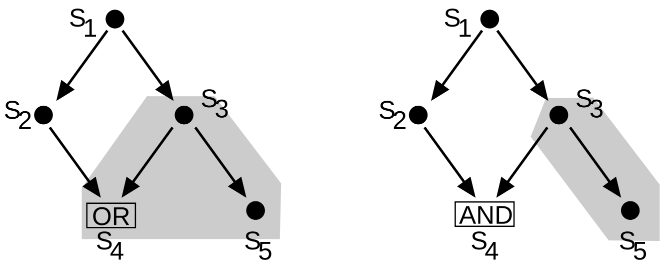

Figure 4.6: *Influence regions are constrained by local logics. The left plot shows in grey the influence region of S_3 if S_4 is reigned by an OR-logic. If the logic changes to an AND, S_4 lies no longer in the influence region of S_3, because the second parent S_2 lies outside of it.*

Multiple knockouts Since epistatic effects involve more than one gene, they cannot be deduced from single knock-out experiments. The model has to be extended to data attained by silencing more than one gene at the same time. This will not change the scoring function, but more sophisticated silencing schemes have to be developed, which encode predictions both from single-gene and multi-gene knockouts. Since the

number of possible multiple knockouts increases exponentionally, tools to choose the most informative experiments are needed. Experimental design or *active learning* deals with deciding which interventions to perform to learn the structure of a model as quickly as possible and to discriminate optimally between alternative models. This is an active area of research in Machine Learning [138, 88]. For reconstruction of regulatory networks, a number of methods have been proposed in different frameworks: for Bayesian networks [103, 152], physical network models [150], Boolean networks [63], and dynamical modeling [135].

4.3 Accuracy and sample size requirements

Section 4.2 introduced a Bayesian score to find silencing schemes explaining the data well. We will demonstrate its potential in two steps. First, we investigate accuracy and sample size requirements in a controlled simulation setting. In a second step, we show that our approach is also useful in a real biological scenario by applying it to a dataset on *Drosophila* immune response. This section evaluates how our algorithm responds to different levels of noise in the data, how accurate it is and how many replicates of intervention screens are needed for reliable pathway reconstruction. To answer these questions, we performed simulations consisting of five steps:

1. We randomly generated a directed acyclic graph T with 20 nodes and 40 edges. This is the core topology of S-genes.
2. Then, we connected 40 E-genes to the core T of S-genes. Together they form an extended topology T'. To evaluate how the position of E-genes affects the results, we implemented three different ways of attaching E-genes to S-genes: either two E-genes are assigned to each S-gene, or E-gene positions are distributed uniformly, or positions are chosen preferentially downstream (also random but with a higher probability for S-genes at the end of pathways).
3. From the extended topology T' we generated random datasets using eight different repetition numbers per knockout experiment ($r \in \{1, \dots, 5, 8, 12, 16\}$). The experiment then consists of $20 \cdot r$ “microarrays”, each corresponding to one of r repeated knockouts of one of the 20 signaling genes. For each knockout experiment the response of all E-genes is simulated from T' using error probabilities α_{data} and β_{data}. The false negative rate is fixed to $\beta_{\text{data}} = 0.05$ and the false positive rate α_{data} is varied from 0.1 to 0.5.
4. We randomly selected three existing edges in the graph T and three pairs of non-connected nodes. Using these six edges, there are $2^6 = 64$ possible modifications of T, including the original pathway T itself. Some of the selected edges in T may be missing and some new links may be added. The 64 pathways were used as input hypotheses of our algorithm.
5. We scored the 64 pathway hypotheses by the marginal likelihood of Eq. (4.4) with parameters $\alpha_{\text{score}} = 0.1$ and $\beta_{\text{score}} = 0.3$. Note that these (arbitrarily chosen)

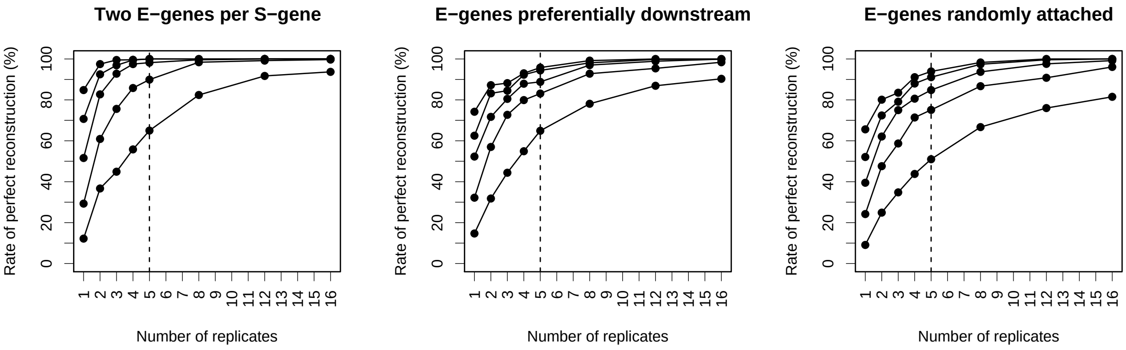

Figure 4.7: *Results of simulation experiments on random graphs. The number of replicates r in the data are on the x-axis, while the y-axis corresponds to the rate of perfect reconstructions in* 1000 *runs. Each plot corresponds to a different way of attaching E-genes to S-genes. The curves in each plot correspond to $\alpha_{data} = 0.1, \ldots, 0.5$ in descending order: the lower the curve, the higher the noise in data generation. The dashed vertical line indicates performance with $r = 5$ replicates—a practical upper limit for most microarray studies. The plots show excellent results for low noise levels. Even with $\alpha_{data} = 0.5$ the method does not break down, but identifies the complete true pathway in more than half of all simulation runs.*

values are different from $(\alpha_{\text{data}}, \beta_{\text{data}})$ used for data generation. If the best score is achieved by the original pathway T this is counted as a perfect reconstruction. Even with a single incorrect edge the reconstruction is counted as failed.

Simulation results Fig. 4.7 depicts the average number of perfect reconstructions for every $(\alpha_{\text{data}}, r)$-pair over 1000 simulation runs. The plots show: rates of perfect reconstruction are best when each S-gene has two E-genes as reporters and worst for purely random E-gene connections. The frequency to identify the correct pathway quickly increases with the number of replicates. With five replicates and low noise levels, the rate of perfect reconstruction is above 90% in all simulations. Even with a noise level of 50% the algorithm correctly identified the right hypothesis in more than half of the runs.

The impact of these simulation results becomes apparent when comparing it to results by graphical models of the correlation structure of expression values. Basso *et al.* [7] show that their own method, ARACNe, compares favorably against static Bayesian networks on a simulated network with 19 nodes. The smallest sample size used in the comparison is 100 observations, the biggest 2000. They show a steady increase in performance, which levels off at around 1000 observations. Hartemink [55] finds dynamical Bayesian networks to be even more accurate than ARACNe on the same simulation network with the same dataset sizes. In summary, at least 1000 observations are needed to reliably reconstruct a 19 node network by Bayesian networks or ARACNe. Our simulations show that less than 100 samples are needed to reconstruct a network of the same size when using gene silencing screens. This is one order of magnitude less. For 20 nodes, 100 observations correspond to five replicates

per intervention, which give an almost consummate rate of perfect reconstruction in Fig. 4.7.

4.4 Application to Drosophila immune response

We applied our method to data from a study on innate immune response in *Drosophila* [12], which was already described as an example in the introduction. Selectively removing signaling components (S-genes in our terminology) blocked induction of all, or only parts, of the transcriptional response to LPS (E-genes in our terminology).

Data preprocessing The dataset consists of 16 Affymetrix-microarrays: 4 replicates of control experiments without LPS and without RNAi (negative controls), 4 replicates of expression profiling after stimulation with LPS but without RNAi (positive controls), and 2 replicates each of expression profiling after applying LPS and silencing one of the four candidate genes *tak*, *key*, *rel*, and *mkk4/hep*. For preprocessing, we performed normalization on probe level using a variance stabilizing transformation [60], and probe set summarization using a median polish fit of an additive model [67]. In this data, 68 genes show a more than 2-fold up-regulation between control and LPS stimulation. We used them as E-genes in the model.

Adaptive discretization Next, we transformed the continuous expression data to binary values. An E-gene's state in an RNAi experiment is set to 1 if its expression value is sufficiently far from the mean of the positive controls, *i.e.* if the intervention interrupted the information flow. If the E-genes expression is close to the mean of positive controls, we set its state to 0. Formally, this strategy is implemented as follows. Let C_{ik} be the continuous expression level of E_i in experiment k. Let μ_i^+ be the mean of positive controls for E_i, and μ_i^- the mean of negative controls. To derive binary data E_{ik}, we defined individual cutoffs for every gene E_i by:

$$E_{ik} = \begin{cases} 1 & \text{if } C_{ik} < \kappa \cdot \mu_i^+ + (1-\kappa) \cdot \mu_i^-, \\ 0 & \text{else.} \end{cases} \tag{4.10}$$

We tried values of κ from 0 to 1 in steps of 0.1. Fig. 4.8 shows the results. To control the false negative rate, we chose $\kappa = 0.7$: It is the smallest value where all negative controls are correctly recognized.

Figure 4.9 shows the continuous and discretized data as used in the analysis. Silencing *tak* affects almost all E-genes. A subset of E-genes is additionally affected by silencing *mkk4/hep*, another disjoint subset by silencing *rel* and *key*. Note that expression profiles of *rel* and *key* silencing are almost indistinguishable both in the continuous and discrete data matrix. The subset structure observed by Boutros *et al.* [12] is visible, but obscured by noise. Some of it can be attributed to noise inherent in biolgical systems and to measurement noise. Some of it may be due to our selection of E-genes. Including more biological knowledge on regulatory modules in *Drosophila* immune response would help to clarify the picture. The following results show that even

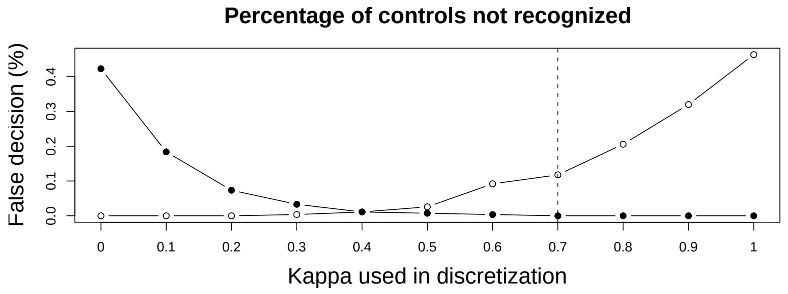

Figure 4.8: *Discretizing according to Eq. (4.10) with κ varying from 0 to 1 (x-axis). The black dots show, which percentage of negative controls was not recognized,* i.e. *set to 0 instead of 1. The circles show, which percentage of positive controls wrongly assigned to state 1. The dashed line indicates the smallest value of κ, at which all negative controls were correctly identified (the black dots hit zero).*

from noisy data the dominant biological features of the dataset can be reconstructed without having to rely on prior knowledge.

Score parameters We used the two scoring functions developed in this chapter. To compute the marginal likelihood of Eq. (4.4) we need to specify the global error rates α and β. The discretization is consistent with a small value of false negative rate β. We set it to $\beta = 0.05$. The false positive rate α was estimated from the positive controls: The relative frequency of negative calls there was just below 15%. Thus we set $\alpha = 0.15$. Trying different values of α and β did not change the results qualitatively, except when very large und unrealistic error probabilities were chosen. We compare these results with the results obtained from using the full marginal likelihood of Eq. (4.8). There we have to specify four prior parameters. We set $a_0 = b_1 = 1$. Both values correspond to false observations (see Table 4.1) and should be small compared to the other two weights, if there is a clear signal in the data. We chose a_1 and b_0 to be equal and varied their value from 1 to 10.

Results Input hypotheses to the algorithm were all silencing schemes on four genes. The four S-genes can form $2^{12} = 4096$ pathways, which result in 355 different silencing schemes. Fig. 4.10 compares the result from applying both scoring functions. The distribution of marginal likelihood from Eq. (4.4) over the 30 top ranked silencing schemes in Fig. 4.10 shows a clear peak: A single silencing scheme achieves the best score. It is well separated from a group of four silencing schemes having almost the same second-best score. Only after a wide gap all other silencing schemes follow. The ranking of silencing schemes is stable, when using different values of α and β, but the gap is sometimes less pronounced.

For the full marginal likelihood of Eq. (4.8) and low values of a_1 and b_0, we get a fully connected graph as the best model: no structure was found in the data. When the value increases, the scoring landscape looks more and more similar to the results obtained from Eq. (4.4). For $a_1 = b_0 = 5$, both scores result in the same winning

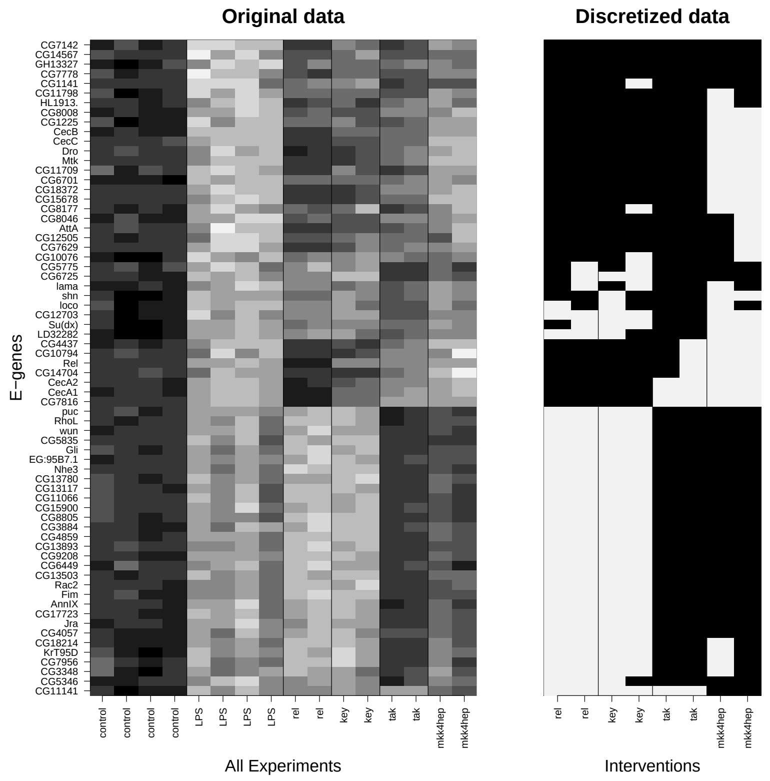

Figure 4.9: *Data on* Drosophila *immune response.* **Left:** *the normalized, gene-wise scaled data from [12]. Black stands for low expression and white for high expression. Rows are E-genes selected for differential expression after LPS stimulation (as seen in the first eight colums).* **Right:** *The data from silencing experiments after discretization ($\kappa = 0.7$) as used in our analysis. We only show the eight columns in the data matrix corresponding to RNAi experiments. The subset structure is visible, but obscured by noise.*

model. In the right plot of Fig. 4.10 we show the result for $a_1 = b_0 = 9$. It is the smallest value for which both scores agree on the five highest ranked models.

The topology of the best silencing scheme obtained from both scoring functions is shown in Fig. 4.11. It can be constructed from three different pathway hypotheses: One is the topology shown in Fig. 4.11, which is transitively closed, the other two miss either the edge from *tak* to *rel* or from *tak* to *key*. This is an example of prediction equivalence. The key features of the data are preserved in all three pathway topologies. The signal runs through *tak* before splitting into two pathway branches, one containing *mkk4/hep*, the other both *key* and *rel*. There is no hint of cross-talk between the two branches of the pathway. All in all, our result fits exactly to the conclusions Boutros *et al.* [12] drew from the data.

Fig. 4.12 shows the expected position of E-genes given the optimal silencing scheme of Fig. 4.11. Both predictions agree very well and show only subtle differences. The double-headed arrow in Fig. 4.11 indicates that the order of *key* and *rel* cannot be

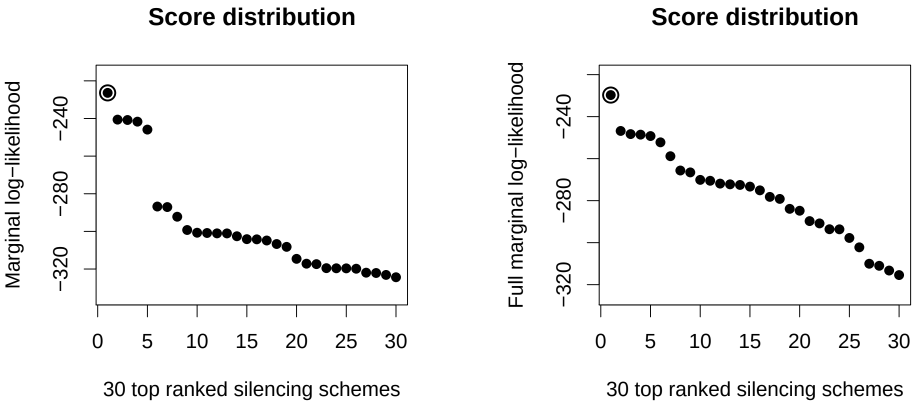

Figure 4.10: *The score distribution over the* 30 *top scoring silencing schemes. The same silencing scheme (circeled) achieves the best score in both plots. In the left plot (Eq. 4.4,* $\alpha = 0.15$, $\beta = 0.05$*), it is well separated from a small group of four lagging behind with a pronounced gap to the rest. In the right plot (Eq. 4.8,* $a_0 = b_1 = 1$, $a_1 = b_0 = 9$*), the distribution is more continuous. The five top ranking silencing schemes are the same for both scoring functions. If the value of* a_1 *and* b_0 *is further increased, the right plot converges towards the left one and shows a clear gap between the best ranking silencing schemes and the rest.*

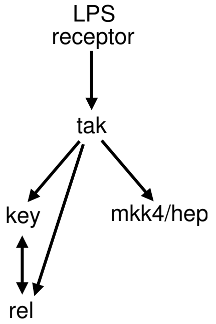

Figure 4.11: *Topology of the top-scoring silencing scheme on the* Drosophila *data. It clearly shows the fork below* tak *with* key *and* rel *on one side and* mkk4/hep *on the other. The double-headed arrow between* key *and* rel *indicates that they are undistinguishable from this data.*

resolved from this dataset, which was to be expected from the nearly identical profiles in Fig. 4.9. This is also the reason, why the posterior position of E-genes in the upper half of Fig. 4.12 is distributed equally on both S-genes. The data is undecided about the relative position of *key* and *rel*, and so is the posterior. However, it is known that *rel* is the transcription factor regulating the downstream genes (see chapter 1). This knowledge could have been easily introduced into a model prior $p(\Phi)$ penalizing topologies not showing *rel* below *key*. We refused to do this on purpose. The results here show how well pathway features can be reconstructed just based on experimental data, without any biological prior knowledge.

A measure of uncertainty In Bayesian terminology, maximizing the marginal likelihood is equivalent to calculating the mode of the posterior distribution on model space, assuming a uniform prior. When scoring all possible pathways, we have derived a complete posterior distribution on model space, which does not only estimate a

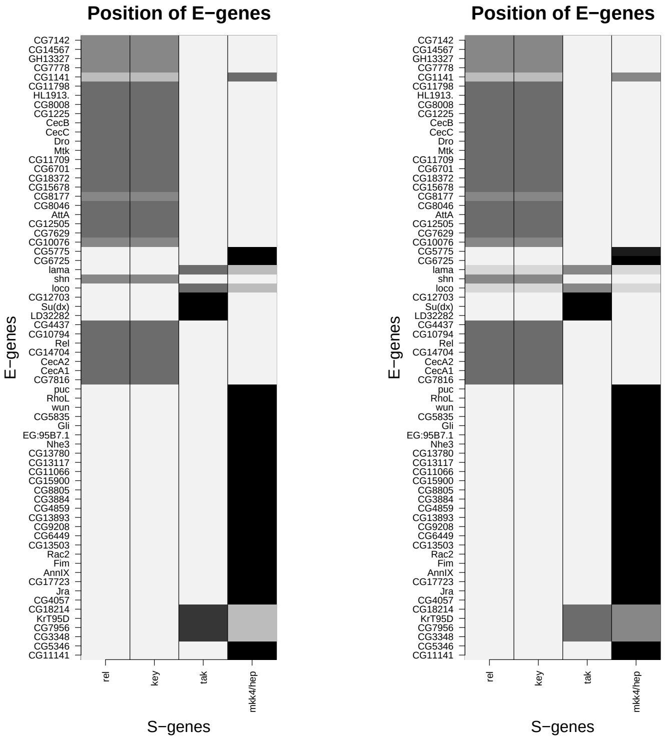

Figure 4.12: *Expected position of E-genes on the* Drosophila *data.* **Left:** *The expected position of E-genes given the silencing scheme with highest marginal likelihood of the data computed from Eq. (4.5). The lower half of E-genes is attributed to* mkk4/hep, *the upper half mostly to* key *and* rel, *which show almost the same intervention profiles (see Fig. 4.9).* **Right:** *Expected position of E-genes computed from Eq. 4.9.*

single pathway model, but also accurately describes the uncertainties involved in the reconstruction process. A flat posterior distribution indicates ambiguities in reconstructing the pathway. What Fig. 4.10 shows is a well pronounced maximium for both scores. This indicates that we found the dominant structure in the data with high certainty. This conclusion is strengthened by inspecting the four silencing schemes achieving the second best score in both plots in Fig. 4.10. They all share the fork beneath *tak* and only differ from the best solution in Fig. 4.11 by missing one or two of the edges between *tak*, *key* and *rel*. All of them represent well the key features of the data.

Chapter 5

Summary and outlook

Genome-scale gene silencing screens pose novel challenges to computational biology. At present, RNA interference appears to be the most efficient technology for producing large-scale gene intervention data. This dissertation developed methodology to tackle two problems peculiar to gene silencing data:

1. Gene perturbation effects cannot be controlled deterministically and have to be modeled stochastically. The uncertainty of intervention effects in a noisy environment is modeled by choosing informative prior distributions for the relationship between regulators and their targets. We formalize this approach in the general framework of conditional Gaussian networks in chapter 3.
2. Direct observations of intervention effects on other pathway components are often not available. Large-scale datasets may only contain observations of secondary downstream effects. Learning from secondary effects is implemented via a two-leveled model of an unobserved pathway with observable downstream reporters. In chapter 4 we develop a Bayesian scoring function to evaluate models with respect to data.

Each of these two problems becomes aparent in different modeling situations. Accounting for stochasticity of interventions is of special importance when reconstructing transcriptional regulatory networks from microarray data. In this setting we assume that expression states of gene coding for transcription factors are good approximations of the activation state of the trascription factor protein. Under this assumption, the correlation structure of genes in different conditions allows conclusions about transcriptional regulators and their targets. Silencing a gene leads to primary effects at other genes in the model and increases the accuracy of network reconstruction.

The second challenge is learning from indirect information and secondary effects. This becomes important when inferring signal transduction pathways from phenotypical changes after interventions. In the cell, a signal is propagated on protein level and mRNA concentrations mostly stay constant for all pathway components. Thus, interventions do not lead to primary effects observable at other pathway components. Instead, reflections of signaling activity perceived in expression levels of downstream genes after pathway perturbations can be used to reconstruct non-transcriptional

features of signaling pathways. Single reporter genes below the pathway of interest can be used as transcriptional phenotypes. Subset patterns on observed phenotype changes allow inference of regulatory hierarchies. In simulation studies we confirmed small sample size requirements and high reconstruction accuracy for the Bayesian score devised to evaluate candidate models. The usefulness of our approach on real data was shown by analyzing a study of *Drosophila* innate immune response.

Non-transcriptional phenotypes In chapter 4 we used reporter genes downstream the pathway of interest to reconstructed a regulatory hierarchy. Expression changes of reporter genes can be interpreted as transcriptional phenotypes. In fact, any other kind of binary phenotype could also be used in our analysis. The only requirement is that the number of phenotypes is large enough and contains a meaningful subset structure. We plan to extend our approach to data from large-scale screens in *C. elegans* [102, 52]. Phenotypes measured there include “no developing embryos seen 48 hours after dsRNA injection”, “Reduced fecundity of injected worm”, “osmotically pressure sensitivity”, or “multiple cavities”. Until now, genes in the *C. elegans* genome have only been clustered according to phenotype similarities [53]. Elucidating regulatory hierarchies remains an open question.

Scaling up model size In its present form, the algorithm proposed in chapter 4 can be applied to filter (several thousands of) pathway hypotheses and to find those well supported by experimental data. The hypotheses build on existing biological expertise. This constrained search space can be interpreted as the result of a rigid structure prior focussing on biological relevant hypotheses. To apply our method to large-scale intervention data with thousands of silenced genes and little biological prior knowledge, model search will have to be improved. There seem to be two promising avenues for further research. One could combine optimal subnetworks to big networks, as it is done in quartett-puzzling algorithms in phylogeny [132]. Another strategy is to define a neighborhood relation on the set of silencing schemes and use techniques of combinatorial optimization to explore the score landscape. The contribution of this thesis is a scoring function to link data with models. Efficient search heuristics are the topic of future research.

The need for a holistic view The internal organization of the cell comprises many layers. The *genome* refers to the collection of information stored in the DNA, while the *transcriptome* includes all gene transcripts. On the next level the *proteome* covers the set of all proteins. The *metabolome* contains small molecules—sugars, salts, amino acids, and nucleotides—that participate in metabolic reactions required for the maintenance and normal function of a cell. Results of internal reactions are features of the cell like growth or viability, which can be taken as *phenotypes* to study gene function. To understand the complexity of living cells future research will need to build models including all these layers. Statistical inference on parts of the system will not provide the mechanistic insights functional genomics is seeking for. Recent research concentrates on combining information from genome, transcriptome and proteome, *e.g.* by building models jointly on expression and protein-DNA binding data. This is a necessary step into the right direction. However, these models will still

be fragmentary if they not include (and predict) phenotypical changes of interventions into the normal course of action in the cell. We will only understand what we can break.

It's the biology, stupid! This thesis explored how to recover features of cellular pathways from gene expression data. All in all, this thesis shows: pathway reconstruction is not an issue of more advanced models and more sophisticated inference techniques. Pathway reconstruction is a matter of careful experimental planning and design. Well designed experiments focus on a pathway of interest and probe information flow by interventions. Only a small sample size and simple statistics are then needed to extract the relevant information from data.

Bibliography

[1] Alfred V. Aho, M.R. Garey, and Jeffrey D. Ullman. The transitive reduction of a directed graph. *SIAM J. Comput.*, 1(2):131 – 137, 1972.

[2] Tatsuya Akutsu, Satoru Kuhara, Osamu Maruyama, and Satoru Miyano. Identification of gene regulatory networks by strategic gene disruptions and gene overexpressions. In *Proc. 9th Annual ACM-SIAM Symposium on Discrete Algorithms*, pages 695–702, 1998.

[3] Tatsuya Akutsu, Satoru Kuhara, Osamu Maruyama, and Satoru Miyano. A system for identifying genetic networks from gene expression patterns produced by gene disruptions and overexpressions. In Satoru Miyano and T. Takagi, editors, *Genome Informatics 9*, pages 151–160, Tokyo, 1998. Universal Academy Press.

[4] Bruce Alberts, Alexander Johnson, Julian Lewis, Martin Raff, Keith Roberts, and Peter Walter. *Molecular Biology of the Cell.* Garland Science, New York, 4 edition, 2002.

[5] M Ashburner, CA Ball, JA Blake, D Botstein, H Butler, JM Cherry, AP Davis, K Dolinski, SS Dwight, JT Eppig, MA Harris, DP Hill, L Issel-Tarver, A Kasarskis, S Lewis, JC Matese, JE Richardson, M Ringwald, GM Rubin, and G Sherlock. Gene ontology: tool for the unification of biology. The Gene Ontology Consortium. *Nat Genet*, 25(1):25–9, May 2000.

[6] L Avery and S Wasserman. Ordering gene function: the interpretation of epistasis in regulatory hierarchies. *Trends Genet*, 8(9):312–6, Sep 1992.

[7] Katia Basso, Adam A Margolin, Gustavo Stolovitzky, Ulf Klein, Riccardo Dalla-Favera, and Andrea Califano. Reverse engineering of regulatory networks in human B cells. *Nat Genet*, Mar 2005.

[8] Matthew J. Beal, Francesco Falciani, Zoubin Ghahramani, Claudia Rangel, and David L. Wild. A Bayesian approach to reconstructing genetic regulatory networks with hidden factors. *Bioinformatics*, 21(3):349–356, 2005.

[9] Allister Bernard and Alexander J Hartemink. Informative structure priors: joint learning of dynamic regulatory networks from multiple types of data. *Pac Symp Biocomput*, pages 459–70, 2005.

[10] David R Bickel. Probabilities of spurious connections in gene networks: application to expression time series. *Bioinformatics*, 21(7):1121–8, Apr 2005.

[11] Susanne G. Bøttcher. *Learning Bayesian Networks with Mixed Variables.* PhD thesis, Aalborg University, Denmark, 2004.

[12] Michael Boutros, Hervé Agaisse, and Norbert Perrimon. Sequential activation of signaling pathways during innate immune responses in Drosophila. *Dev Cell*, 3(5):711–22, Nov 2002.

[13] Michael Boutros, Amy A. Kiger, Susan Armknecht, Kim Kerr, Marc Hild, Britta Koch, Stefan A. Haas, Heidelberg Fly Array Consortium, Renato Paro, and Norbert Perrimon. Genome-Wide RNAi Analysis of Growth and Viability in Drosophila Cells. *Science*, 303(5659):832–835, 2004. DOI: 10.1126/science.1091266.

[14] C.T. Brown, A.G. Rust, P.J.C. Clarke, Z. Pan, M.J. Schilstra, T.D. Buysscher, G. Griffin, B.J. Wold, R.A. Cameron, E.H. Davidson, and H. Bolouri. New computational approaches for analysis of cis-regulatory networks. *Developmental Biology*, (246):86–102, 2002.

[15] Thijn R. Brummelkamp and René Bernards. Innovation: New tools for functional mammalian cancer genetics. *Nature Reviews Cancer*, 3(10):781–789, 2003.

[16] Svetlana Bulashevska and Roland Eils. Inferring genetic regulatory logic from expression data. *Bioinformatics*, Mar 2005.

[17] Wray L. Buntine. Theory refinement of Bayesian networks. In *Uncertainty in Artificial Intelligence*, 1991.

[18] AJ Butte and IS Kohane. Mutual information relevance networks: functional genomic clustering using pairwise entropy measurements. *Pac Symp Biocomput*, pages 418–29, 2000.

[19] Anne E Carpenter and David M Sabatini. Systematic genome-wide screens of gene function. *Nat Rev Genet*, 5(1):11–22, Jan 2004.

[20] David M. Chickering. Learning equivalence classes of Bayesian network structures. In *Proceedings of Twelfth Conference on Uncertainty in Artificial Intelligence, Portland, OR*, pages 150–157. Morgan Kaufmann, August 1996.

[21] David M. Chickering, David Heckerman, and Christopher Meek. A Bayesian approach to learning Bayesian networks with local structure. In *Proceedings of Thirteenth Conference on Uncertainty in Artificial Intelligence*, Providence, RI, 1997. Morgan Kaufmann.

[22] Gregory F. Cooper. A Bayesian Method for Causal Modeling and Discovery Under Selection. In C. Boutilier and M. Goldszmidt, editors, *Uncertainty in Artificial Intelligence; Proceedings of the Sixteenth Conference*, pages 98–106, San Mateo, California, 2000. Morgan Kaufmann.

[23] Gregory F. Cooper and Edward Herskovits. A Bayesian Method for the Induction of Probabilistic Networks from Data. *Machine Learning*, 9:309–347, 1992.

[24] Gregory F. Cooper and Changwon Yoo. Causal discovery from a mixture of experimental and observational data. In K. Laskey and H. Prade, editors, *Proc. Fifthteenth Conference on Uncertainty in Artificial Intelligence (UAI '99)*, pages 116–125, San Francisco, Calif., 1999. Morgan Kaufman.

[25] Eric H. Davidson, Jonathan P. Rast, Paola Oliveri, Andrew Ransick, Cristina Calestani, Chiou-Hwa Yuh, Takuya Minokawa, Gabriele Amore, Veronica Hinman, Cesar Arenas-Mena, Ochan Otim, C. Titus Brown, Carolina B. Livi, Pei Yun Lee, Roger Revilla, Alistair G. Rust, Zheng jun Pan, Maria J. Schilstra, Peter J. C. Clarke, Maria I. Arnone, Lee Rowen, R. Andrew Cameron, David R. McClay, Leroy Hood, and Hamid Bolouri. A Genomic Regulatory Network for Development. *Science*, 295(5560):1669–1678, 2002.

[26] Armaity P Davierwala, Jennifer Haynes, Zhijian Li, Rene L Brost, Mark D Robinson, Lisa Yu, Sanie Mnaimneh, Huiming Ding, Hongwei Zhu, Yiqun Chen, Xin Cheng, Grant W Brown, Charles Boone, Brenda J Andrews, and Timothy R Hughes. The synthetic genetic interaction spectrum of essential genes. *Nat Genet*, 37(10):1147–52, Oct 2005.

[27] Alberto de la Fuente, Nan Bing, Ina Hoeschele, and Pedro Mendes. Discovery of meaningful associations in genomic data using partial correlation coefficients. *Bioinformatics*, 20(18):3565–3574, 2004.

[28] Diego di Bernardo, Gardner Timothy S, and James J Collins. Robust identification of large genetic networks. *Pac Symp Biocomput*, pages 486–97, 2004.

[29] Diego di Bernardo, Michael J Thompson, Timothy S Gardner, Sarah E Chobot, Erin L Eastwood, Andrew P Wojtovich, Sean J Elliott, Scott E Schaus, and James J Collins. Chemogenomic profiling on a genome-wide scale using reverse-engineered gene networks. *Nat Biotechnol*, 23(3):377–83, Mar 2005.

[30] Adrian Dobra, Chris Hans, Beatrix Jones, Joseph R. Nevins, Guang Yao, and Mike West. Sparse graphical models for exploring gene expression data. *Journal of Multivariate Analysis*, 90(1):196–212, July 2004.

[31] Nancy Van Driessche, Janez Demsar, Ezgi O Booth, Paul Hill, Peter Juvan, Blaz Zupan, Adam Kuspa, and Gad Shaulsky. Epistasis analysis with global transcriptional phenotypes. *Nat Genet*, 37(5):471–7, May 2005.

[32] Mathias Drton and Michael D. Perlman. Model selection for gaussian concentration graphs. *Biometrika*, 91(3), 2004.

[33] Mathias Drton and Michael D. Perlman. A SINful approach to gaussian graphical model selection. *Submitted to special issue of Statistical Science*, 2004.

[34] Richard Durbin, Sean Eddy, Anders Krogh, and Graeme Mitchison. *Biological sequence analysis.* Cambridge University Press, 1998.

[35] David Edwards. *Introduction to Graphical Modelling.* Springer, 2000.

[36] Bradley Efron and Robert J. Tibshirani. *An introduction to the boostrap.* Chapman and Hall, 1993.

[37] MB Eisen, PT Spellman, PO Brown, and D Botstein. Cluster analysis and display of genome-wide expression patterns. *Proc Natl Acad Sci U S A*, 95(25):14863–8, Dec 1998.

[38] Andrew Fire, SiQun Xu, Mary K. Montgomery, Steven A. Kostas, Samuel E. Driver, and Craig C. Mello. Potent and specific genetic interference by double-stranded RNA in caenorhabditis elegans. *Nature*, 391(6669):806 – 811, Feb 1998.

[39] Nir Friedman. Learning belief networks in the presence of missing values and hidden variables. In D. Fisher, editor, *Proc. of the Fourteenth Inter. Conf. on Machine Learning (ICML97)*, pages 125–133, San Francisco, CA, 1997. Morgan Kaufmann.

[40] Nir Friedman. The Bayesian Structural EM Algorithm. In G. F. Cooper and S. Moral, editors, *Proc. of the Fourteenth Conf. on Uncertainty in Artificial Intelligence (UAI'98)*, pages 129–138, San Francisco, CA, 1998. Morgan Kaufmann.

[41] Nir Friedman. Inferring Cellular Networks Using Probabilistic Graphical Models. *Science*, 303(5659):799–805, 2004.

[42] Nir Friedman and Moises Goldszmidt. Learning Bayesian networks with local structure. In Michael I. Jordan, editor, *Learning in Graphical Models*, pages 421–459. MIT Press, Cambridge, MA, 1998.

[43] Nir Friedman and Daphne Koller. Being Bayesian about network structure: A Bayesian approach to structure discovery in Bayesian networks. *Machine Learning*, 50:95–126, 2003.

[44] Nir Friedman, Michal Linial, Iftach Nachman, and Dana Pe'er. Using Bayesian networks to analyze expression data. *Journal of Computational Biology*, 7(3):601–620, August 2000.

[45] Nir Friedman, Kevin Murphy, and Stuart Russell. Learning the structure of dynamic probabilistic networks. In *Proceedings of the 14th Annual Conference on Uncertainty in Artificial Intelligence (UAI-98)*, pages 139–147, San Francisco, CA, 1998. Morgan Kaufmann Publishers.

[46] Nir Friedman, Iftach Nachman, and Dana Peer. Learning bayesian network structures from massive datasets: The sparse candidate algorithm. In *Proc. of Uncertainty in Artificial Intelligence*, 1999.

[47] Timothy S. Gardner, Diego di Bernardo, David Lorenz, and James J. Collins. Inferring genetic networks and identifying compound mode of action via expression profiling. *Science*, 301(5629):102–105, 2003.

[48] Dan Geiger and David Heckerman. Learning Gaussian Networks. In Ramon López de Mántaras and David Poole, editors, *Proceedings of the Tenth Annual Conference on Uncertainty in Artificial Intelligence*, pages 235–243, Seattle, Washington, USA, July 29-31 1994. Morgan Kaufmann.

[49] Andrew Gelman, John B. Carlin, Hal S. Stern, and Donald B. Rubin. *Bayesian Data Analysis*. Chapman and Hall-CRC, 1996.

[50] Viola Gesellchen, David Kuttenkeuler, Michael Steckel, Nadge Pelte, and Michael Boutros. An RNA interference screen identifies Inhibitor of Apoptosis Protein 2 as a regulator of innate immune signalling in Drosophila. *EMBO Rep*, 6(10):979–84, Oct 2005.

[51] Marian A. C. Groenenboom, Athanasius F. M. Marée, Paulien Hogeweg, and Simon Levin. The RNA Silencing Pathway: The Bits and Pieces That Matter. *PLoS Computational Biology*, 1(2):e21, 2005.

[52] Kristin C Gunsalus, Hui Ge, Aaron J Schetter, Debra S Goldberg, Jing-Dong J Han, Tong Hao, Gabriel F Berriz, Nicolas Bertin, Jerry Huang, Ling-Shiang Chuang, Ning Li, Ramamurthy Mani, Anthony A Hyman, Birte Snnichsen, Christophe J Echeverri, Frederick P Roth, Marc Vidal, and Fabio Piano. Predictive models of molecular machines involved in Caenorhabditis elegans early embryogenesis. *Nature*, 436(7052):861–5, Aug 2005.

[53] Kristin C Gunsalus, Wan-Chen Yueh, Philip MacMenamin, and Fabio Piano. RNAiDB and PhenoBlast: web tools for genome-wide phenotypic mapping projects. *Nucleic Acids Res*, 32(Database issue):D406–10, Jan 2004.

[54] SP Gygi, Y Rochon, BR Franza, and R Aebersold. Correlation between protein and mRNA abundance in yeast. *Mol Cell Biol*, 19(3):1720–30, Mar 1999.

[55] Alexander J. Hartemink. Reverse engineering gene regulatory networks. *Nat Biotechnol*, 23(5):554–5, May 2005.

[56] Alexander J. Hartemink, Daniel K. Gifford, Tommi S. Jaakkola, and Richard A. Young. Combining location and expression data for principled discovery of genetic regulatory network models. In *Proceedings of Pacific Symposium on Biocomputing 7:437-449*, 2002.

[57] W.K. Hastings. Monte carlo sampling methods using markov chains and their applications. *Biometrika*, 57:97–109, 1970.

[58] David Heckerman, Dan Geiger, and David Maxwell Chickering. Learning Bayesian Networks: The Combination of Knowledge and Statistical Data. *Machine Learning*, 20(3):197–243, Sep. 1995.

[59] Jules A Hoffmann. The immune response of Drosophila. *Nature*, 426(6962):33–8, Nov 2003.

[60] Wolfgang Huber, Anja von Heydebreck, Holger Sltmann, Annemarie Poustka, and Martin Vingron. Variance stabilization applied to microarray data calibration and to the quantification of differential expression. *Bioinformatics*, 18(Suppl 1):S96–104, 2002.

[61] Timothy R. Hughes, Matthew J. Marton, Allan R. Jones, Christopher J. Roberts, Roland Stoughton, Christopher D. Armour, Holly A. Bennett, Ernest Coffey, Hongyue Dai, Yudong D. He, Matthew J. Kidd, Amy M. King, Michael R. Meyer, David Slade, Pek Y. Lum, Sergey B. Stepaniants, Daniel D. Shoemaker, Daniel Gachotte, Kalpana Chakraburtty, Julian Simon, Martin Bard, and Stephen H. Friend. Functional discovery via a compendium of expression profiles. *Cell*, 102:109–126, July 2000.

[62] Dirk Husmeier. Sensitivity and specificity of inferring genetic regulatory interactions from microarray experiments with dynamic Bayesian networks. *Bioinformatics*, 19(17):2271–2282, 2003.

[63] Trey Ideker, Vesteinn Thorsson, and Richard M. Karp. Discovery of regulatory interactions through perturbation: inference and experimental design. In *Proc. of the Pacic Symp. on Biocomputing*, volume 5, pages 302–313, 2000.

[64] Seiya Imoto, T. Goto, and S. Miyano. Estimation of genetic networks and functional structures between genes by using Bayesian network and nonparametric regression. In *Pacific Symposium on Biocomputing*, volume 7, pages 175–186, 2002.

[65] Seiya Imoto, T. Higuchi, T. Goto, K. Tashiro, S. Kuhara, and S. Miyano. Combining microarrays and biological knowledge for estimating gene networks via Bayesian networks. In *Proc. 2nd Computational Systems Bioinformatics*, pages 104–113, 2003.

[66] Seiya Imoto, Sunyong Kim, Takao Goto, Satoru Miyano, Sachiyo Aburatani, Kousuke Tashiro, and Satoru Kuhara. Bayesian network and nonparametric heteroscedastic regression for nonlinear modeling of genetic network. *J Bioinform Comput Biol*, 1(2):231–52, Jul 2003.

[67] Rafael A Irizarry, Benjamin M Bolstad, Francois Collin, Leslie M Cope, Bridget Hobbs, and Terence P Speed. Summaries of Affymetrix GeneChip probe level data. *Nucleic Acids Res*, 31(4):e15, Feb 2003.

[68] Tsuyoshi Kato, Koji Tsuda, and Kiyoshi Asai. Selective integration of multiple biological data for supervised network inference. *Bioinformatics*, Feb 2005.

[69] Hirohisa Kishino and Peter J. Waddell. Correspondence analysis of genes and tissue types and finding genetic links from microarray data. In A.K. Dunker, A. Konagaya, S. Miyano, and T.Takagi, editors, *Genome Informatics*, Tokyo, 2000. Universal Academy Press.

[70] Dennis Kostka and Rainer Spang. Finding disease specific alterations in the co-expression of genes. *Bioinformatics*, 20 Suppl 1:I194–I199, Aug 2004.

[71] David Latchman. *Gene regulation – A eukaryotic perspective.* Stanley Thornes, 2002.

[72] Steffen L. Lauritzen. *Graphical Models.* Clarendon Press, Oxford, 1996.

[73] Steffen L. Lauritzen. Causal inference from graphical models, 1999.

[74] Tong Ihn Lee, Nicola J Rinaldi, Franois Robert, Duncan T Odom, Ziv Bar-Joseph, Georg K Gerber, Nancy M Hannett, Christopher T Harbison, Craig M Thompson, Itamar Simon, Julia Zeitlinger, Ezra G Jennings, Heather L Murray, D Benjamin Gordon, Bing Ren, John J Wyrick, Jean-Bosco Tagne, Thomas L Volkert, Ernest Fraenkel, David K Gifford, and Richard A Young. Transcriptional regulatory networks in Saccharomyces cerevisiae. *Science*, 298(5594):799–804, Oct 2002.

[75] Jan van Leeuwen. Graph algorithms, 1990. in: Handbook of Theoretical Computer Science, Elsevier, 525–632.

[76] Shoudan Liang, Stefanie Fuhrmann, and Roland Somogyi. REVEAL, a general reverse engineering algorithm for inference of genetic network architectures. In *Proc. of Pacific Symposium on Biocomputing*, number 3, pages 18–29, 1998.

[77] Lennard Ljung. *System Identification – Theory for the User.* Prentice Hall, 2nd edition, 1999.

[78] D. Madigan, S. Andersson, M. Perlman, and C. Volinsky. Bayesian model averaging and model selection for markov equivalence classes of acyclic graphs. *Communications in Statistics: Theory and Methods*, 25:2493–2519, 1996.

[79] Paul M Magwene and Junhyong Kim. Estimating genomic coexpression networks using first-order conditional independence. *Genome Biol*, 5(12):R100, 2004.

[80] Florian Markowetz, Jacques Bloch, and Rainer Spang. Non-transcriptional pathway features reconstructed from secondary effects of RNA interference. *Bioinformatics*, 21(21):4026–4032, 2005.

[81] Florian Markowetz, Steffen Grossmann, and Rainer Spang. Probabilistic soft interventions in conditional Gaussian networks. In Robert Cowell and Zoubin Ghahramani, editors, *Proc. Tenth International Workshop on Artificial Intelligence and Statistics*, Jan 2005.

[82] Florian Markowetz and Rainer Spang. Evaluating the effect of perturbations in reconstructing network topologies. In Kurt Hornik, Friedrich Leisch, and Achim Zeileis, editors, *Proceedings of the 3rd International Workshop on Distributed Statistical Computing (DSC 2003)*, 2003.

[83] Florian Markowetz and Rainer Spang. Molecular diagnosis: classification, model selection, and performance evaluation. *Methods of Information in Medicine*, 44(3):438–43, 2005.

[84] Nicolai Meinshausen and Peter Bühlmann. High dimensional graphs and variable selection with the lasso. *Annals of Statistics*, ?(?):?, 2005.

[85] Gunter Meister and Thomas Tuschl. Mechanisms of gene silencing by double-stranded RNA. *Nature*, 431(7006):343–9, Sep 2004.

[86] Ron Milo, Shai S. Shen-Orr, Shalev Itzkovitz, Nadav Kashtan, Dmitri Chklovskii, and Uri Alon. Network Motifs: Simple Building Blocks of Complex Networks. *Science*, 298(5594):824–827, 2002.

[87] K. Murphy and S. Mian. Modelling gene expression data using dynamic Bayesian networks. Technical report, Computer Science Division, University of California, Berkeley, CA, 1999.

[88] Kevin P. Murphy. Active Learning of Causal Bayes Net Structure, 2001.

[89] I. Nachman, A. Regev, and N. Friedman. Inferring quantitative models of regulatory networks from expression data. *Bioinformatics*, 20(suppl.1):i248–256, 2004.

[90] Iftach Nachman, Gal Elidan, and Nir Friedman. "ideal parent"structure learning for continuous variable networks. In *AUAI '04: Proceedings of the 20th conference on Uncertainty in artificial intelligence*, pages 400–409, Arlington, Virginia, United States, 2004. AUAI Press.

[91] Yuki Naito, Tomoyuki Yamada, Takahiro Matsumiya, Kumiko Ui-Tei, Kaoru Saigo, and Shinichi Morishita. dsCheck: highly sensitive off-target search software for double-stranded RNA-mediated RNA interference. *Nucleic Acids Res*, 33(Web Server issue):W589–91, Jul 2005.

[92] Carl D Novina and Phillip A Sharp. The RNAi revolution. *Nature*, 430(6996):161–4, Jul 2004.

[93] Irene M Ong, Jeremy D Glasner, and David Page. Modelling regulatory pathways in E. coli from time series expression profiles. *Bioinformatics*, 18 Suppl 1:S241–8, 2002.

[94] George Orphanides and Danny Reinberg. A unified theory of gene expression. *Cell*, 108(4):439–51, Feb 2002.

[95] Jason A. Papin, Tony Hunter, Bernhard O. Palsson, and Shankar Subramaniam. Reconstruction of cellular signalling networks and analysis of their properties. *Nat Rev Mol Cell Biol*, 6(2):99–111, 2005.

[96] Judea Pearl. *Probabilistic Reasoning in Intelligent Systems: networks of plausible inference.* Morgan Kaufmann, 1988.

[97] Judea Pearl. *Causality: Models, Reasoning and Inference.* Cambridge University Press, Cambridge, 2000.

[98] Juan M Pedraza and Alexander van Oudenaarden. Noise propagation in gene networks. *Science*, 307(5717):1965–9, Mar 2005.

[99] Dana Pe'er, Aviv Regev, Gal Elidan, and Nir Friedman. Inferring subnetworks from perturbed expression profiles. *Bioinformatics*, 17(90001):S215–S224, 2001.

[100] JM Peña, J Bjrkegren, and J Tegnér. Growing Bayesian network models of gene networks from seed genes. *Bioinformatics*, 21 Suppl 2:ii224–ii229, Sep 2005.

[101] Bruno-Edouard Perrin, Liva Ralaivola, Aurelien Mazurie, Samuele Bottani, Jacques Mallet, and Florence d'Alche Buc. Gene networks inference using dynamic Bayesian networks. *Bioinformatics*, 19(90002):138ii–148, 2003.

[102] Fabio Piano, Aaron J Schetter, Diane G Morton, Kristin C Gunsalus, Valerie Reinke, Stuart K Kim, and Kenneth J Kemphues. Gene clustering based on RNAi phenotypes of ovary-enriched genes in C. elegans. *Curr Biol*, 12(22):1959–64, Nov 2002.

[103] Iosifina Pournara and Lorenz Wernisch. Reconstruction of gene networks using Bayesian learning and manipulation experiments. *Bioinformatics*, 20(17):2934–2942, 2004.

[104] Claudia Rangel, John Angus, Zoubin Ghahramani, Maria Lioumi, Elizabeth Sotheran, Alessia Gaiba, David L. Wild, and Francesco Falciani. Modeling T-cell activation using gene expression profiling and state-space models. *Bioinformatics*, 20(9):1361–1372, 2004.

[105] Claudia Rangel, David L. Wild, Francesco Falciani, Zoubin Ghahramani, and Alessia Gaiba. Modeling biological responses using gene expression profiling and linear dynamical systems. In *Proceedings of the 2nd International Conference on Systems Biology*, pages 248–256, Madison, WI, 2001. Omnipress.

[106] Jonathan M Raser and Erin K O'Shea. Control of stochasticity in eukaryotic gene expression. *Science*, 304(5678):1811–4, Jun 2004.

[107] John Jeremy Rice, Yuhai Tu, and Gustavo Stolovitzky. Reconstructing biological networks using conditional correlation analysis. *Bioinformatics*, 10 2004.

[108] Robert W. Robinson. Counting labeled acyclic digraphs. In F. Harary, editor, *New Directions in the Theory of Graphs*, pages 239–273. Academic Press, New York, 1973.

[109] Simon Rogers and Mark Girolami. A Bayesian regression approach to the inference of regulatory networks from gene expression data. *Bioinformatics*, May 2005.

[110] Nitzan Rosenfeld, Jonathan W Young, Uri Alon, Peter S Swain, and Michael B Elowitz. Gene regulation at the single-cell level. *Science*, 307(5717):1962–5, Mar 2005.

[111] Julien Royet, Jean-Marc Reichhart, and Jules A Hoffmann. Sensing and signaling during infection in Drosophila. *Curr Opin Immunol*, 17(1):11–7, Feb 2005.

[112] J. Rung, T. Schlitt, A. Brazma, K. Freivalds, and J. Vilo. Building and analysing genome-wide gene disruption networks. *Bioinformatics*, 18(90002):202S–210, 2002.

[113] Ravi Sachidanandam. RNAi as a bioinformatics consumer. *Brief Bioinform*, 6(2):146–62, Jun 2005.

[114] Karen Sachs, Omar Perez, Dana Pe'er, Douglas A Lauffenburger, and Garry P Nolan. Causal protein-signaling networks derived from multiparameter single-cell data. *Science*, 308(5721):523–9, Apr 2005.

[115] Juliane Schäfer and Korbinian Strimmer. An empirical Bayes approach to inferring large-scale gene association networks. *Bioinformatics*, 21(6):754–64, Mar 2005.

[116] Bernhard Schölkopf and Alexander J. Smola. *Learning with kernels.* The MIT Press, Cambridge, MA, 2002.

[117] Gideon Schwarz. Estimating the dimension of a model. *Annals of Statistics*, 6(2):461–464, Mar 1978.

[118] Eran Segal, Nir Friedman, Naftali Kaminski, Aviv Regev, and Daphne Koller. From signatures to models: understanding cancer using microarrays. *Nat Genet*, 37 Suppl:S38–45, Jun 2005.

[119] Eran Segal, Dana Pe'er, Aviv Regev, Daphne Koller, and Nir Friedman. Learning module networks. *Journal of Machine Learing Research*, 6(Apr):557–588, 2005.

[120] Eran Segal, Michael Shapira, Aviv Regev, Dana Pe'er, David Botstein, Daphne Koller, and Nir Friedman. Module networks: identifying regulatory modules and their condition-specific regulators from gene expression data. *Nature Genetics*, 34(2):166–176, 2003.

[121] Shai S. Shen-Orr, Ron Milo, Shmoolik Mangan, and Uri Alon. Network motifs in the transcriptional regulation network of Escherichia coli. *Nature Genetics*, 31(1):64 – 68, April 2002. doi:10.1038/ng881.

[122] Jose Silva, Kenneth Chang, Gregory J Hannon, and Fabiola V Rivas. RNA-interference-based functional genomics in mammalian cells: reverse genetics coming of age. *Oncogene*, 23(51):8401–9, Nov 2004.

[123] I Simon, J Barnett, N Hannett, CT Harbison, NJ Rinaldi, TL Volkert, JJ Wyrick, J Zeitlinger, DK Gifford, TS Jaakkola, and RA Young. Serial regulation of transcriptional regulators in the yeast cell cycle. *Cell*, 106(6):697–708, Sep 2001.

[124] Peter W. F. Smith and Joe Whittaker. Edge exclusion tests for graphical Gaussian models. In Michael Jordan, editor, *Learning in Graphical Models*, pages 555 – 574. MIT Press, 1999.

[125] V. Anne Smith, Erich D. Jarvis, and Alexander J. Hartemink. Evaluating functional network inference using simulations of complex biological systems. *Bioinformatics*, 18(90001):216S–224, 2002.

[126] PT Spellman, G Sherlock, MQ Zhang, VR Iyer, K Anders, MB Eisen, PO Brown, D Botstein, and B Futcher. Comprehensive identification of cell cycle-regulated genes of the yeast Saccharomyces cerevisiae by microarray hybridization. *Mol Biol Cell*, 9(12):3273–97, Dec 1998.

[127] Peter Spirtes, Clark Glymour, and Richard Scheines. *Causation, Prediction, and Search.* MIT Press, Cambridge, MA, second edition, 2000.

[128] Harald Steck and Tommi Jaakkola. On the dirichlet prior and Bayesian regularization. In *Advances in Neural Information Processing Systems 15*, Cambridge, MA, 2002. MIT Press.

[129] Harald Steck and Tommi Jaakkola. (Semi-)predictive discretization during model selection. Technical Report AI Memo AIM-2003-002, MIT, 2003.

[130] Harald Steck and Tommi S. Jaakkola. Unsupervised active learning in large domains. In *Proceedings of the Eighteenth Annual Conference on Uncertainty in Artificial Intelligence*, 2002.

[131] Harald Steck and Tommi S. Jaakkola. Bias-corrected bootstrap and model uncertainty. In Sebastian Thrun, Lawrence Saul, and Bernhard Schölkopf, editors, *Advances in Neural Information Processing Systems 16*. MIT Press, Cambridge, MA, 2004.

[132] Korbinian Strimmer and Arndt von Haeseler. Quartet Puzzling: A Quartet Maximum-Likelihood Method for Reconstructing Tree Topologies. *Mol Biol Evol*, 13(7):964–969, 1996.

[133] Joshua M Stuart, Eran Segal, Daphne Koller, and Stuart K Kim. A gene-coexpression network for global discovery of conserved genetic modules. *Science*, 302(5643):249–55, Oct 2003.

[134] Yoshinori Tamada, SunYong Kim, Hideo Bannai, Seiya Imoto, Kousuke Tashiro, Satoru Kuhara, and Satoru Miyano. Estimating gene networks from gene expression data by combining Bayesian network model with promoter element detection. *Bioinformatics*, 19(90002):227ii–236, 2003.

[135] Jesper Tegner, M K Stephen Yeung, Jeff Hasty, and James J Collins. Reverse engineering gene networks: integrating genetic perturbations with dynamical modeling. *Proc Natl Acad Sci U S A*, 100(10):5944–9, May 2003.

[136] Jin Tian and Judea Pearl. Causal discovery from changes. In Jack S. Breese and Daphne Koller, editors, *Proceedings of the 17th Conference in Uncertainty in Artificial Intelligence*, pages 512–521, Seattle, Washington, USA, 2001. Morgan Kaufmann. Part 1 of a two-part paper.

[137] Jin Tian and Judea Pearl. Causal discovery from changes: a Bayesian approach. In Jack S. Breese and Daphne Koller, editors, *Proceedings of the 17th Conference in Uncertainty in Artificial Intelligence*, pages 512–521, Seattle, Washington, USA, 2001. Morgan Kaufmann. Part 2 of a two-part paper.

[138] Simon Tong and Daphne Koller. Active Learning for Structure in Bayesian Networks. In *Proceedings of the Seventeenth International Joint Conference on Artifical Intelligence (IJCAI)*, Seattle, Washington, August 2001.

[139] Thomas S. Verma and Judea Pearl. Equivalence and synthesis of causal models. In P. B. Bonissone, M. Henrion, L. N. Kanal, and J. F. Lemmer, editors, *Proc. Sixth Conf. on Uncertainty in Artificial Intelligence*, pages 255–268. North-Holland, Amsterdam, 1990.

[140] Andreas Wagner. How to reconstruct a large genetic network from n gene perturbations in fewer than n^2 easy steps. *Bioinformatics*, 17(12):1183–1197, 2001.

[141] Andreas Wagner. Estimating Coarse Gene Network Structure from Large-Scale Gene Perturbation Data. *Genome Res.*, 12(2):309–315, 2002.

[142] Andreas Wagner. Reconstructing pathways in large genetic networks from genetic perturbations. *Journal of Computational Biology*, 11(1):53–60, 2004.

[143] Wei Wang and Gregory F. Cooper. An bayesian method for biological pathway discovery from high-throughput experimental data. In *Proc. 3rd International IEEE Computer Society Computational Systems Bioinformatics Conference (CSB 2004)*, pages 645–646, Stanford, CA, USA, 2004. IEEE Computer Society.

[144] Anja Wille and Peter Bühlmann. Tri-graph: a novel graphical model with application to genetic regulatory networks. Technical report, Seminar for Statistics, ETH Zrich, 2004.

[145] Anja Wille, Philip Zimmermann, Eva Vranová, Andreas Fürholz, Oliver Laule, Stefan Bleuler, Lars Hennig, Amela Prelic, Peter von Rohr, Lothar Thiele, Eckart Zitzler, Wilhelm Gruissem, and Peter Bühlmann. Sparse graphical Gaussian modeling of the isoprenoid gene network in Arabidopsis thaliana. *Genome Biol*, 5(11):R92, 2004.

[146] Frank C. Wimberly, Thomas Heiman, Joseph Ramsey, and Clark Glymour. Experiments on the accuracy of algorithms for inferring the structure of genetic regulatory networks from microarray expression levels. In *Proc. IJCAI 2003 Bioinformatics Workshop*, 2003.

[147] Cecily J. Wolfe, Isaac S. Kohane, and Atul J. Butte. Systematic survey reveals general applicability of "guilt-by-association" within gene coexpression networks. *BMC Bioinformatics 2005*, 6:227, 2005.

[148] Y. Yamanishi, J.-P. Vert, and M. Kanehisa. Protein network inference from multiple genomic data: a supervised approach. *Bioinformatics*, 20(suppl.1):i363–370, 2004.

[149] Chen-Hsiang Yeang, Trey Ideker, and Tommi Jaakkola. Physical network models. *Journal of Computational Biology*, 11(2):243 – 262, 2004.

[150] Chen-Hsiang Yeang, H Craig Mak, Scott McCuine, Christopher Workman, Tommi Jaakkola, and Trey Ideker. Validation and refinement of gene-regulatory pathways on a network of physical interactions. *Genome Biology*, 6(R62), 2005.

[151] Stephen Yeung, Jesper Tegnér, and James J Collins. Reverse engineering gene networks using singular value decomposition and robust regression. *Proc Natl Acad Sci U S A*, 99(9):6163–8, Apr 2002.

[152] C. Yoo and G. F. Cooper. An evaluation of a system that recommends microarray experiments to perform to discover gene-regulation pathways. *Journal Artificial Intelligence in Medicine*, 31(2):169–182, 2004.

[153] Changwon Yoo, Vesteinn Thorsson, and Gregory F. Cooper. Discovery of causal relationships in a generegulation pathway from a mixture of experimental and oberservational DNA microarray data. In *Proceedings of Pacific Symposium on Biocomputing 7:498-509*, 2002.

[154] Jing Yu, V. Anne Smith, Paul P. Wang, Alexander J. Hartemink, and Erich D. Jarvis. Advances to Bayesian network inference for generating causal networks from observational biological data. *Bioinformatics*, Jul 2004.

[155] Daniel E. Zak, Francis J. Doyle, Gregory E. Gonye, and James S. Schwaber. Simulation studies for the identification of genetic networks from cDNA array and regulatory activity data. In *Proceedings of the Second International Conference on Systems Biology*, pages 231–238, 2001.

[156] Daniel E. Zak, Gregory E. Gonye, James S. Schwaber, and Francis J. Doyle. Importance of Input Perturbations and Stochastic Gene Expression in the Reverse Engineering of Genetic Regulatory Networks: Insights From an Identifiability Analysis of an In Silico Network. *Genome Res.*, 13(11):2396–2405, 2003.

[157] Min Zou and Suzanne D. Conzen. A new dynamic Bayesian network (DBN) approach for identifying gene regulatory networks from time course microarray data. *Bioinformatics*, 21(1):71–79, 2005.

Notation and Definitions

Here I list often used abbreviations and notations for quick reference. The notation in chapter 3 complies to Steffen Lauritzens book [72], the statistical standard reference on graphical models.

Chapter 1

DNA	Deoxyribonucleic acid
RNA	Ribonucleic acid
mRNA	messenger RNA
RNAi	RNA interference

Chapter 2

V	set of graph vertices representing network components
p	number of pathway components, $p = \|V\|$.
$T = (V, \mathcal{E})$	network topology on vertices V and edge set $\mathcal{E}$
D	special case: T is a directed acyclic graph
X, x	a random variable and its realization
$\mathbf{X}$, $\mathbf{x}$	a set or vector of random variables and its realization
M	data matrix $M = \{\mathbf{x}^1, \dots, \mathbf{x}^N\}$
N	sample size
$P(X = x) \equiv p(x)$	if no confusion can arise
$X \perp Y$	X and Y are independent random variables
$X \perp Y \mid Z$	X and Y are independent given Z
Σ, $\hat{\Sigma}$	covariance matrix and its estimator
K	precision matrix, inverse covariance matrix, $K = \Sigma^{-1}$
$\theta_{v\|pa(v)}$	the parameters of random variable X_v given the values of its parents $\mathbf{X}_{pa(v)}$ in the Bayesian network DAG
$\alpha_{i_\delta\|\mathbf{i}_{pa(\delta)}}$	Dirichlet parameters for a discrete node $\delta \in V$ with parent state $\mathbf{i}_{pa(\delta)}$ in a Bayesian network.
DAG	directed acyclic graph
GGM	Gaussian graphical model
DBN	dynamic Bayesian network
LPD	local probability distribution

Chapter 3

$do(X_v = x'_v)$	do-operator: X_v is fixed to state x'_v
$\mathrm{d}(x)$	Dirac-function, point mass at $x = 0$
Δ	the set of discrete vertices
Γ	the set of Gaussian vertices
$\mathbf{X} = (\mathbf{I}, \mathbf{Y})$	the set of variables splits into discrete ones ($\mathbf{I}$) and continuous ones ($\mathbf{Y}$).
I_δ, i_δ	a discrete random variable ($\delta \in \Delta$) and its realization
Y_γ, y_γ	a Gaussian random variable ($\gamma \in \Gamma$) and its realization
$\mathcal{I}_\delta$, $\mathcal{I}_{pa(\delta)}$	the state space of I_δ and its parents $\mathbf{I}_{pa(\delta)}$
$\mathcal{P}(\theta, w, t)$	pushing operator applied to parameters θ with strength w towards target state t
$\theta_{\delta\|\mathbf{i}_{pa(\delta)}}$	parameters of discrete variable I_δ
$\theta_{\gamma\|\mathbf{i}_{pa(\gamma)}}$	parameters of Gaussian variable Y_γ depending on the values $\mathbf{i}_{pa(\gamma)}$ of discrete parents

Chapter 4

$V = \mathbf{E} \cup \mathbf{S}$	vertices correspond to signaling genes (S-genes) and reporter genes (E-genes)
E_i	reporter genes ($i = 1, \ldots, m$)
S_j	signaling genes ($j = 1, \ldots, p$)
E_{ik}, e_{ik}	binomial random variable corresponding to the state of E_i in experiment k and its realization
C_{ik}	continuous expression states
T	pathway topology on S-genes
T'	extended topoloy including S-genes and E-genes
Φ	silencing scheme
α	probability to observe a false positive effect
β	probability to observe a false negative effect
$\theta_i = j$	position parameter: S_j is the parent of E_i in T'
η_i	probability to observe an effect at E_i
η_{is}	probability to observe an effect at E_i given parent state s
M_i^s	data of E_i when parent was in state s
n_{ise}	number of observations $e_{ik} = e$ when parent state is s

Zusammenfassung

Die vorliegende Arbeit beschreibt, wie sich regulatorische Netze und Signalwege rekonstruieren lassen, indem die Expression einzelner Gene gezielt unterdrückt wird. Die Arbeit widmet sich im Besonderen zwei statistischen Problemen:

1. Die Stärke einer Intervention ist meist unbekannt und unterliegt stochastischen Einflüssen in der Zelle. Ich demonstriere eine stochastische Modellierung der Auswirkung eines Experiments auf das Ziel-Gen in Kapitel 3.
2. Gene, die zu einem Signalweg beitragen, zeigen keine veränderte Expression, wenn andere Teile des Signalwegs gestört werden. Ich zeige in Kapitel 4, wie ein Signalweg aus sekundären Effekten rekonstruiert werden kann.

Kapitel 1: Biologische Einführung Nach Grundlagen der Genexpression in eukaryotischen Zellen klärt das erste Kapitel die beiden zentralen Begriffe dieser Arbeit: *transkriptionelle regulatorische Netzwerke* und *molekulare Signalwege*. Regulatorische Netze bestehen aus Transkriptionsfaktoren und den Genen, an die sie binden. Signalwege geben durch Proteininteraktionen und -modifikationen Reize von der Zellmembran an den Zellkern weiter.

Kapitel 2: Statistische Verfahren der Netzwerk-Rekonstruktion Das zweite Kapitel legt die mathematischen und statistischen Grundlagen für die folgenden Teile der Arbeit. Es baut auf dem Begriff der bedingten Unabhängigkeit auf und gibt einen Überblick über statistische Modelle, die zur Netzwerk-Rekonstruktion eingesetzt werden. Unter anderem behandelt das Kapitel Korrelationsgraphen, Gaußsche graphische Modelle und Bayessche Netzwerke.

Kapitel 3: Rekonstruktion transkriptioneller regulatorischer Netzwerke Ich entwickele ein statistisches Modell für Daten aus *gene silencing* Experimenten. In Experimenten lässt sich nur schwer bestimmen, wie weit die Expression des Ziel-Gens tatsächlich unterdrückt wurde. Ich modelliere dieses stochastische Verhalten, indem ich lokale *a priori*-Verteilungen anpassen. Das Ergebnis des Kapitels ist eine Theorie sogennanter *probabilistic soft interventions*.

Kapitel 4: Rekonstruktion von Protein-Signalwegen Die Unterdrückung von Proteinen, die zu Beginn von Signalketten stehen, resultiert in mehr Phänotypen als das Ausschalten von Proteinen am unteren Ende der Hierarchie. Ich formalisiere diese Idee in einem mehrstufigen Modell. Es enthält eine unbeobachtbare regulatorische Hierarchie von Signalmolekülen, deren *knockdown* zu beobachtbaren Phänotypen führt.

Teilmengen-Beziehungen auf der Menge der beobachteten Phänotypen ermöglichen es, die regulatorische Hierarchie zu rekonstruieren. Ich demonstriere den Nutzen unserer Methode in Simulationsexperimenten und an einem biologischen Beispiel in *Drosophila melanogaster*.

Curriculum Vitae

Florian Markowetz
Johanniterstrasse 25
10961 Berlin
Tel: (030) 69 56 49 85
`florian.markowetz@molgen.mpg.de`

geboren am 06. November 1976
Geburtsort: München
Staatsangehörigkeit: Deutsch
Familienstand: ledig

Akademischer Grad

Diplom-Mathematiker

am	25. September 2001
an der	Ruprecht-Karls Universität Heidelberg
Betreuer:	Prof. Dr. E. Mammen (Universität Heidelberg)
	Prof. Dr. M. Vingron (DKFZ Heidelberg, jetzt MPI-MG Berlin)
Arbeit:	Support Vector Machines in Bioinformatics

Magister Artium in Philosophie

am	07. Juni 2002
an der	Ruprecht-Karls Universität Heidelberg
Betreuer:	Prof. Dr. A. Kemmerling
Arbeit:	Freiheit und Bedingtheit des Willens - zwischen Neurowissenschaft und Philosophie

Ausbildung

Seit Jan. 2002	Doktorand in der Gruppe *Computational Diagnostics* der Abteilung *Computational Molecular Biology* am Max-Planck-Institut für molekulare Genetik, Berlin.
04/1997–03/2002	Universitätsstudium der Philosophie an der Ruprecht-Karls Universität Heidelberg.
09/2000–06/2001	Diplomarbeit in der Arbeitsgruppe *Theoretische Bioinformatik* am Deutschen Krebsforschungszentrum (DKFZ), Heidelberg.
06/1996–03/1997	zum Militärdienst beurlaubt von der Universität Heidelberg.
10/1995–09/2001	Universitätsstudium der Mathematik an der Ruprecht-Karls Universität Heidelberg.
06/1986–06/1995	Altes Kurfürstliches Gymnasium Bensheim

Ehrenwörtliche Erklärung

Hiermit erkläre ich, dass ich diese Arbeit selbstständig verfasst und keine anderen als die angegebenen Hilfsmittel und Quellen verwendet habe.

Berlin, Dezember 2005 Florian Markowetz
