# Supplementary material for: Large scale statistical inference of signaling pathways from RNAi and microarray data
Source: BMC Bioinformatics. 2007 Oct 15;8:386. doi: 10.1186/1471-2105-8-386 (PMC2241646; doi:10.1186/1471-2105-8-386)
Supplement: Additional file 1 — top25solutionsBoutrosData. 25 highest scoring network structures for the data by Boutros et al. [file 1471-2105-8-386-S1.gz › nem/inst/doc/nem.tmp]

# — Nested Effects Models —
# An example in *Drosophila* immune response

Florian Markowetz* Holger Fröhlich†

October 8, 2007

**Abstract**

Cellular signaling pathways, which are not modulated on a transcriptional level, cannot be directly deduced from expression profiling experiments. The situation changes, when external interventions like RNA interference or gene knock-outs come into play.

In [5], [4], [6] and [2] we introduced an algorithm to infer non-transcriptional pathway features based on differential gene expression in silencing assays. The method is implemented in the Bioconductor package `nem`. Here we demonstrate its practical use in the context of an RNAi data set investigating the response to microbial challenge in Drosophila melanogaster.

We show in detail how the data is pre-processed and discretized, how the pathway can be reconstructed by different approaches, and how the final result can be post-processed to increase interpretability.

## 1 Drosophila RNAi data

We applied our method to data from a study on innate immune response in *Drosophila* [1]. Selectively removing signaling components blocked induction of all, or only parts, of the transcriptional response to LPS.

**Dataset summary** The dataset consists of 16 Affymetrix-microarrays: 4 replicates of control experiments without LPS and without RNAi (negative controls), 4 replicates of expression profiling after stimulation with LPS

*Lewis-Sigler Institute for Integrative Genomics, Pinceton, NJ 08544, USA. eMail: florian@genomics.princeton.edu; URL: http://genomics.princeton.edu/∼florian

†German Cancer Research Center, Im Neuenheimer Feld 580, 69120 Heidelberg, Germany. eMail: h.froehlich@dkfz-heidelberg.de

but without RNAi (positive controls), and 2 replicates each of expression profiling after applying LPS and silencing one of the four candidate genes *tak*, *key*, *rel*, and *mkk4/hep*.

**Preprocessing and E-gene selection** For preprocessing, we perform normalization on probe level using a variance stabilizing transformation (Huber *et al.*, 2002), and probe set summarization using a median polish fit of an additive model (Irizarry *et al.*, 2003). The result is included as a dataset in the package `nem`.

```
> library(nem)
> data("BoutrosRNAi2002")
```

The function `nem.discretize` implements the following two preprocessing steps: First, we select the genes as effect reporters (E-genes), which are more then two-fold upregulated by LPS treatment. Next, we transform the continuous expression data to binary values. We set an E-genes state in an RNAi experiment to `1` if its expression value is sufficiently far from the mean of the positive controls, *i.e.* if the intervention interrupted the information flow. If the E-genes expression is close to the mean of positive controls, we set its state to `0`.

Let $C_{ik}$ be the continuous expression level of $E_i$ in experiment $k$. Let $\mu_i^+$ be the mean of positive controls for $E_i$, and $\mu_i^-$ the mean of negative controls. To derive binary data $E_{ik}$, we defined individual cutoffs for every gene $E_i$ by:

$$E_{ik} = \begin{cases} 1 & \text{if } C_{ik} < \kappa \cdot \mu_i^+ + (1-\kappa) \cdot \mu_i^-, \\ 0 & \text{else.} \end{cases} \quad (1)$$

```
> res.disc <- nem.discretize(BoutrosRNAiExpression, neg.control = 1:4,
+     pos.control = 5:8, cutoff = 0.7)

discretizing with respect to POS and NEG controls
```

**Estimating error probabilities** From the positive and negative controls, we can estimate the error probabilities $\alpha$ and $\beta$. The type I error $\alpha$ is the number of positive controls discretized to state `1`, and the type II error $\beta$ is the number of negative controls in state `0`. To guard against unrealistically low estimates we add pseudo counts. The error estimates are included into the discretization results:

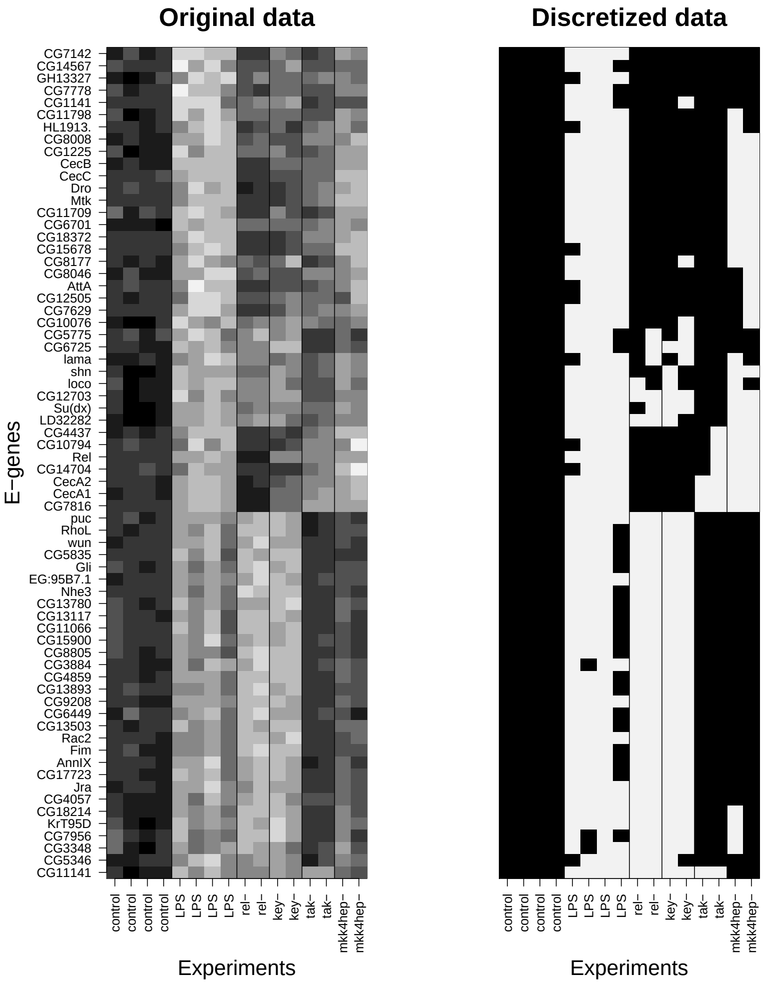


Figure 1: Continuous and discretized data

```
> res.disc$para
```

```
   a    b
0.19 0.07
```

## 2 Applying Nested Effects Models

Which model explains the data best? With only four S-genes, we can exhaustively enumerate all pathway models and search the whole space for the best-fitting model. To score these models use either the marginal likelihood depending on $\alpha$ and $\beta$ (details found in Markowetz *et al.* (2005)) or the full marginal likelihood depending on hyperparameters (details in Markowetz, 2006).

In cases, where exhaustive search over model space is infeasible (i.e. when we have more than 4 or 5 perturbed genes) several heuristics have been developed and integrated into the *nem* package:

- edge-wise and triplets learning [6]
- greedy hillclimbing
- module networks [2]

An interface to all inference techniques is provided by the function `nem()`.

### 2.1 Exhaustive search by marginal likelihood

Scoring models by marginal log-likelihood is implemented in function `score`. Input consists of models and data, the type of the score (`"mLL"` or `"FULLmLL"`), the corresponding paramters (`para`) or hyperparameters (`hyperpara`) and a prior for E-gene positions (`P`).

```
> result <- nem(res.disc$dat, type = "mLL", para = res.disc$para,
+     inference = "search", verbose = FALSE)
> result

Object of class 'score' generated by 'score()'

$graph:  phenotypic hierarchy on genes
$mLL:    a vector of length 355
$pos:    a list   of length 355
$mappos: a vector of length 355
$lambda: 0
```

The output is the highest scoring model (`result$graph`), a vector of scores (`result$mLL`) and a list of E-gene position posteriors (`result$pos`), and a MAP estimate of E-gene positions (`result$mappos`). We can plot the results using the commands:

```
> plot(result, what = "graph")
> plot(result, what = "mLL")
> plot(result, what = "pos")
```

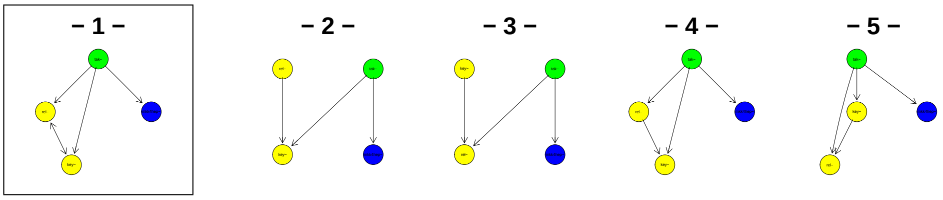


Figure 2: The five silencing schemes getting high scores in Fig. 3. It takes a second to see it, but Nr.2 to 5 are not that different from Nr.1. The main feature, ie. the branching downstream of *tak* is conserved in all of them.

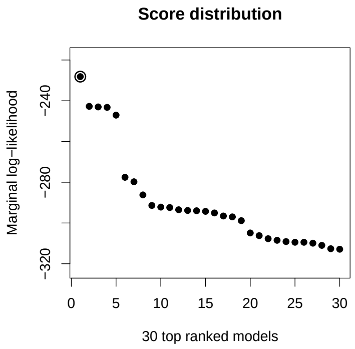


Figure 3: The best 30 scores

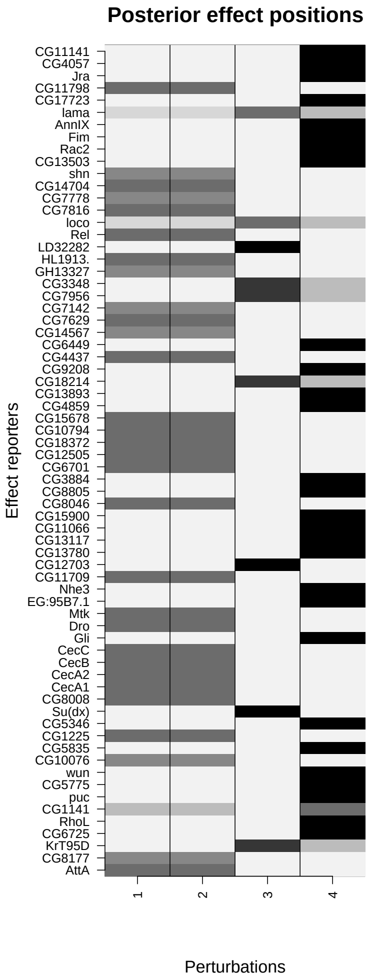


Figure 4: Posterior distributions of E-gene positions given the highest scoring silencing scheme (Nr. 1 in Fig. 2). The MAP estimate corresponds to the row-wise maximum.

## 2.2 Exhaustive search Full marginal likelihood

Additionally to what we did in the paper [5] the PhD thesis [4] contains equations for a "full marginal likelihood" in which error probabilities $\alpha$ and $\beta$ are integrated out. This section shows that using this score we learn the same pathways as before.

```
> result2 <- nem(res.disc$dat, type = "FULLmLL", hyperpara = c(1,
+     9, 9, 1), inference = "search", verbose = FALSE)
> result2
```

```
Object of class 'score' generated by 'score()'

$graph:  phenotypic hierarchy on genes
$mLL:    a vector of length 355
$pos:    a list   of length 355
$mappos: a vector of length 355
$lambda: 0
```

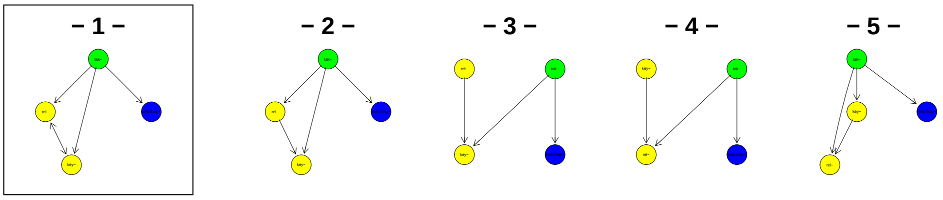


Figure 5: Same topologies as before.

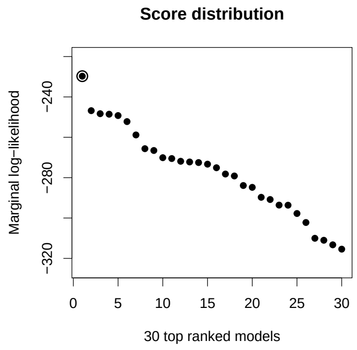


Figure 6: The best 30 scores by full marginal likelihood

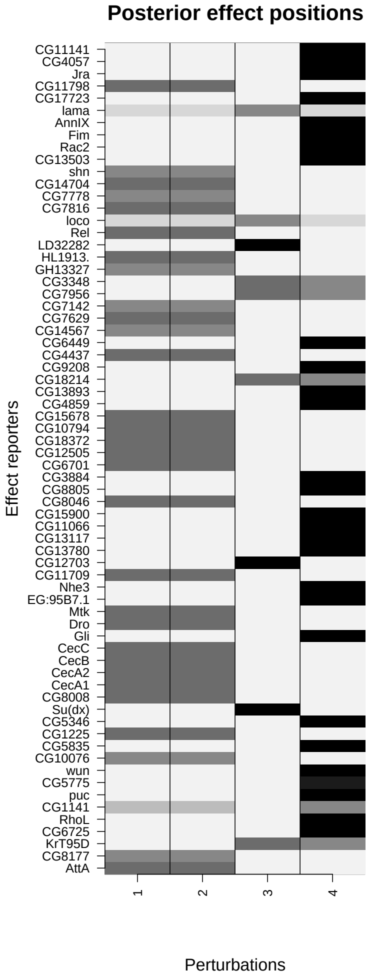


Figure 7: Posterior distributions of E-gene positions given the highest scoring silencing scheme (Nr. 1 in Fig. 5). The MAP estimate corresponds to the row-wise maximum.

## 2.3 Edge-wise learning

Instead of scoring whole pathways, we can learn the model edge by edge [6]. For each pair of genes $A$ and $B$ we infer the best of four possible models: $A \cdot \cdot B$ (unconnected), $A \rightarrow B$ (effects of A are superset of effects of B), $A \leftarrow B$ (subset), and $A \leftrightarrow B$ (undistinguishable effects).

```
> resultPairs <- nem(res.disc$dat, para = res.disc$para, inference = "pairwise",
+     verbose = FALSE)
> resultPairs
```

```
Object of class 'pairwise' generated by 'pairwise.posterior()'

$graph:  phenotypic hierarchy on genes)
$scores: posterior distributions of local models

Summary of MAP estimates:
all  ..  -> <->
  6   2   3   1
```

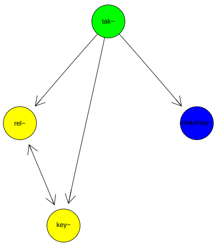


Figure 8: Result of edge-wise learning. Compare this to the result from global search. It looks exactely the same.

## 2.4 Inference from triples

Edge-wise learning assumes independence of edges. But this is not true in transitively closed graphs, where a direct edge must exist whenever there is a longer path between two nodes. Natural extension of edge-wise learning is inference from triples of nodes [6]. In the package `nem` we do it by

```
> resultTriples <- nem(res.disc$dat, para = res.disc$para, inference = "triples",
+     verbose = FALSE)
> resultTriples
```

```
Object of class 'triples' generated by 'triples.posterior()'

$graph:  phenotypic hierarchy on genes
$avg:    matrix of edge frequencies in triple models
```

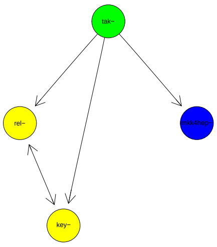


Figure 9: Result of triple learning. Compare this to the result from global search and pairwise learning

## 2.5 Inference with greedy hillclimbing

Greedy hillclimbing is a general search and optimization strategy known from the literature [7]. Given an initial network hypothesis (usually an empty graph) we want to arrive at a local maximum of the likelihood function by successively adding that edge, which adds the most benefit to the current network's likelihood. This procedure is continued until no improving edge can be found any more.

```
> resultGreedy <- nem(res.disc$dat, para = res.disc$para, inference = "nem.greedy",
+     verbose = FALSE)
```

```
Greedy hillclimber for 4 S-genes (lambda = 0 )...
```

```
> resultGreedy
```

```
Object of class 'nem.greedy' generated by 'nem.greedy()'

$graph:  phenotypic hierarchy on genes)
```

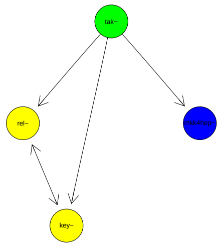


Figure 10: Result of greedy hillclimbing. It is exactly the same as for the exhaustive search.

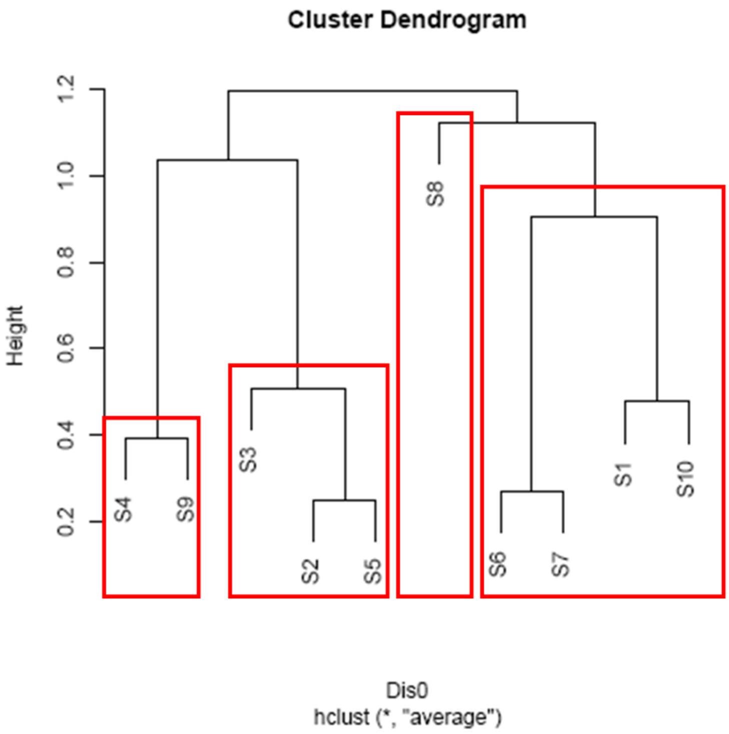


Figure 11: Basic idea of module networks: By successively moving down the cluster hierarchy we identify the clusters (modules) of S-genes, which are marked in red. They contain 4 S-genes at most and can be estimated via exhaustive search.

## 2.6 Inference with module networks

Rather than looking for a complete network hypothesis at once the idea of the module network is to build up a graph from smaller subgraphs, called *modules* in the following [2]. The module network is thus a divide and conquer approach: We first perform a hierarchical clustering of the preprocessed expression profiles of all S-genes, e.g. via average linkage. The idea is that S-genes with a similar E-gene response profile (here: with regard to the Pearson correlation similarity) should be close in the signaling path. We now successively move down the cluster tree hierarchy until we find a cluster with only 4 S-genes at most. Figure 11 illustrates the idea with an assumed network of 10 S-genes. At the beginning we find $S_8$ as a cluster singleton. Then by successively moving down the hierarchy we identify clusters $S_6, S_7, S_1, S_10$, $S_3, S_2, S_5$ and $S_4, S_9$. All these clusters (modules) contain 4 S-genes at most and can thus be estimated by taking the highest scoring of all possible network hypotheses.

Once all modules have been estimated their connections are constructed. This is done in a constraint greedy hillcimbing fashion: We successively add that edge between any pair of S-genes being contained in different modules, which increases the likelihood of the complete network most. This procedure is continued until no improvement can be gained any more, i.e. we have reached a local maximum of the likelihood function.

In the package `nem` we call the module network by

```
> resultMN <- nem(res.disc$dat, para = res.disc$para, inference = "ModuleNetwork",
+     verbose = FALSE)
```

```
Estimating module network of 4 S-genes (lambda = 0 )...
```

```
> resultMN
```

```
Object of class 'ModuleNetwork' generated by 'moduleNetwork()'

$graph:  phenotypic hierarchy on genes)
```

## 2.7 Incorporating prior Assumptions

### 2.7.1 Regularization

The `nem` package allows to specify a prior on the network structure itself. This can be thought of biasing the score of possible network hypotheses towards prior knowledge. It is crucial to understand that in principle in any inference scheme there exist two competing goals: Belief in prior assumptions / prior knowledge versus belief in data. Only trusting the data itself may lead to overfitting, whereas only trusting in prior assumptions does not give any new information and prevents learning. Therefore, we need a trade-off between both goals via a regularization constant $\lambda > 0$, which specifies the belief in our prior assumptions. In the simplest case our assumption could be that the true network structure is sparse, i.e. there are only very few edges. More complex priors could involve separate prior edge probabilities.

```
> resultRegularization <- nem(res.disc$dat, para = res.disc$para,
+     Pm = matrix(0, ncol = 4, nrow = 4), lambda = 10, inference = "search",
+     verbose = FALSE)
> resultRegularization

Object of class 'score' generated by 'score()'

$graph:  phenotypic hierarchy on genes
$mLL:    a vector of length 355
$pos:    a list   of length 355
$mappos: a vector of length 355
$lambda: 10
```

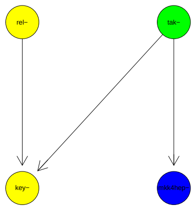


Figure 12: Result of module network learning with regularization towards sparse graph structures ($\lambda = 10$).

In practice we would like to choose a $\lambda$ in an automated fashion. This leads to an instance of the classical *model selection* problem (e.g. [3]) in

statistical learning. One way of dealing with it is to tune $\lambda$ such that the *Akaike information criterion* (AIC)

$$AIC(\lambda, \Phi_{opt}) = -2 \log P(D|\Phi_{opt}) + 2d(\lambda, \Phi_{opt}) \tag{2}$$

becomes minimal [3]. Here $d(\lambda, \Phi_{opt})$ denotes the number of parameters (i.e. the number of unknown edges) in the highest scoring network structure $\Phi_{opt}$.

```
> resultModsel <- nemModelSelection(c(0.1, 1, 10), res.disc$dat,
+     para = res.disc$para, Pm = matrix(0, ncol = 4, nrow = 4),
+     inference = "search", verbose = FALSE)
```

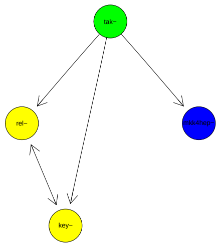

Figure 13: Result of module network learning with regularization towards sparse graph structures and automatic model selection.

#### 2.7.2 Bayesian Model Selection

Searching for an optimal regularization constant relates to a frequentistic point of view to incorporate prior knowledge. Instead, from a Bayesian perspective one should define a prior on the regularization parameter and integrate it out. Here, this is done by assuming an inverse gamma distribution prior on $\nu = \frac{1}{2\lambda}$ with hyperparameters $1, 0.5$, which leads to a simple closed form of the full prior [2]. An advantage of the Bayesian perspective is that no explicit model selection step is needed.

```
> resultBayes <- nem(res.disc$dat, para = res.disc$para, Pm = matrix(0,
+     ncol = 4, nrow = 4), inference = "search", verbose = FALSE)
> resultBayes

Object of class 'score' generated by 'score()'

$graph:  phenotypic hierarchy on genes
```

```
$mLL:    a vector of length 355
$pos:    a list   of length 355
$mappos: a vector of length 355
$lambda: 0
```

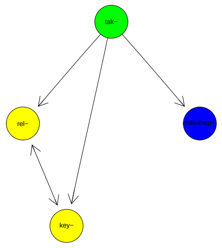


Figure 14: Result of module network learning with a Bayesian network prior favoring sparse graphs.

### 2.8 Omitting the Data Discretization Step

In general performing a data discretization on the expression profiles as described in Sec. 1 can be critical. An alternative is given by taking the raw $p$-value profiles obtained from testing for differential gene expression. In this situation we assume the individual $p$-values in the data matrix to be drawn from a mixture of a uniform, a Beta$(1, \beta)$ and a Beta$(\alpha, 1)$ distribution. The parameters of the distribution are fitted via an EM algorithm [2]. The `nem` package supports such a data format using the option `type = "CONTmLLDens"` in the call of the function `nem`. Moreover there is a function `getDensityMatrix`, which conveniently does all the fitting of the $p$-value profiles and produces diagnostic plots into a user specified directory.

A second possibility to omit the data discretization step is to calculate the effect probability for each gene based on given the empirical distributions of the controls.

```
> densities = getDensityMatrix(myPValueMatrix, dirname = "DiagnosticPlots")
> nem(densities[res.disc$sel, ], type = "CONTmLLDens", inference = "search")
> preprocessed <- nem.cont.preprocess(BoutrosRNAiExpression, neg.control = 1:4,
+     pos.control = 5:8)
> nem(preprocessed$prob.influenced, type = "CONTmLL", inference = "search")
```

# 3 Visualization

```
> plot.effects(res.disc$dat, result)
```

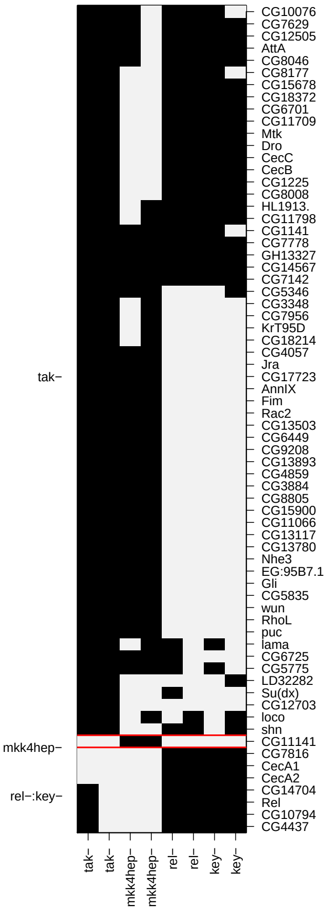


Figure 15: plotting data according to inferred hierarchy

# 4 Post-processing of results

**Combining strongly connected components** First, we identify all nodes/genes which are not distinguishable given the data. This amounts to finding the strongly connected components in the graph. Relish and Key are now combined into one node.

```
> result.scc <- SCCgraph(result$graph, name = TRUE)
> plot(result.scc$graph)
```

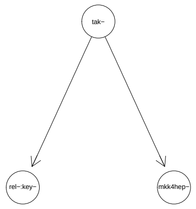


Figure 16: The undistinguishable profiles of *key* and *rel* are summarized into a single node.

**Transitive reduction** Additionally, in bigger graphs `transitive.reduction()` helps to see the structure of the network better. In this simple example there are no shortcuts to remove.

## References

[1] M. Boutros, H. Agaisse, and N. Perrimon. Sequential activation of signaling pathways during innate immune responses in *Drosophila*. *Developmental Cell*, 3(5):711 – 722, 2002.

[2] H. Fröhlich, M. Fellmann, H. Sültmann, A. Poustka, and T. Beißbarth. Estimating large scale signaling networks through nested effects models from intervention effects in microarray data. In *Proc. German Conf. on Bioinformatics*, 2007.

[3] T. Hastie, R. Tibshirani, and J. Friedman. *The Elements of Statistical Learning*. Springer, 2001.

[4] F. Markowetz. *Probabilistic Models for Gene Silencing Data*. PhD thesis, Free University Berlin, 2006.

[5] F. Markowetz, J. Bloch, and R. Spang. Non-transcriptional pathway features reconstructed from secondary effects of rna interference. *Bioinformatics*, 21(21):4026 – 4032, 2005.

[6] F. Markowetz, D. Kostka, O. Troyanskaya, and R. Spang. Nested effects models for high-dimensional phenotyping screens. In *Proc. Int. Conf. on Int. Syst. for Mol. Biol. (ISMB)*, 2007. in press.

[7] S. Russel and P. Norvig. *Artificial Intelligence - A Modern Approach*. Prentice Hall Inc., New Jersey, 1995.

## Session Information

The version number of R and packages loaded for generating the vignette were:

- R version 2.6.0 (2007-10-03), `x86_64-unknown-linux-gnu`
- Locale: `LC_CTYPE=de_DE@euro;LC_NUMERIC=C;LC_TIME=de_DE@euro;LC_COLLATE=de_DE@eur`
- Base packages: base, datasets, graphics, grDevices, methods, stats, tools, utils
- Other packages: class 7.2-37, cluster 1.11.9, e1071 1.5-16, graph 1.16.1, nem 2.0.0, RBGL 1.14.0, Rgraphviz 1.16.0, time 1.0
