# Supplementary material for: Large scale statistical inference of signaling pathways from RNAi and microarray data
Source: BMC Bioinformatics. 2007 Oct 15;8:386. doi: 10.1186/1471-2105-8-386 (PMC2241646; doi:10.1186/1471-2105-8-386)
Supplement: Additional file 1 — top25solutionsBoutrosData. 25 highest scoring network structures for the data by Boutros et al. [file 1471-2105-8-386-S1.gz › nem/..Rcheck/nem/html/internal.html]

R: internal functions

|  |  |
| --- | --- |
| internal {nem} | R Documentation |

## internal functions

### Description

internal functions: do not call these functions directly.

### Usage

```
        various
```

### Arguments

various

### Details

### Value

various

### Author(s)

Holger Froehlich

---

[Package *nem* version 1.4.2 Index]
