# Supplementary material for: Large scale statistical inference of signaling pathways from RNAi and microarray data
Source: BMC Bioinformatics. 2007 Oct 15;8:386. doi: 10.1186/1471-2105-8-386 (PMC2241646; doi:10.1186/1471-2105-8-386)
Supplement: Additional file 1 — top25solutionsBoutrosData. 25 highest scoring network structures for the data by Boutros et al. [file 1471-2105-8-386-S1.gz › nem/..Rcheck/nem/html/CONTmLL.html]

R: Marginal likelihood of a phenotypic hierarchy with continuous data

|  |  |
| --- | --- |
| CONTmLL {nem} | R Documentation |

## Marginal likelihood of a phenotypic hierarchy with continuous data

### Description

computes the marginal likelihood of observed phenotypic data given a phenotypic hierarchy.

### Usage

```
CONTmLL(Phi, prob.inf, Pe)
```

### Arguments

|  |  |
| --- | --- |
| `Phi` | an adjacency matrix with unit main diagonal |
| `prob.inf` | matrix of influence probabilities |
| `Pe` | prior of effect reporter positions in the phenotypic hierarchy |

### Details

It computes the marginal likelihood of a single phenotypic hierarchy.
Usually called from within the function `score`.

### Value

|  |  |
| --- | --- |
| `mLL` | marginal likelihood of a phenotypic hierarchy |
| `pos` | posterior distribution of effect positions in the hierarchy |
| `mappos` | Maximum aposteriori estimate of effect positions |

### Author(s)

Matthias Maneck

### References

### See Also

`score`, `mLL`, `FULLmLL`, `nem.cont.preprocess`

### Examples

```
   data("BoutrosRNAi2002")
   pre <- nem.cont.preprocess(BoutrosRNAiExpression,neg.control=1:4,pos.control=5:8)
   result <- nem(pre$prob.influenced, type="CONTmLL")
```

---

[Package *nem* version 1.4.2 Index]
