# Supplementary material for: Large scale statistical inference of signaling pathways from RNAi and microarray data
Source: BMC Bioinformatics. 2007 Oct 15;8:386. doi: 10.1186/1471-2105-8-386 (PMC2241646; doi:10.1186/1471-2105-8-386)
Supplement: Additional file 1 — top25solutionsBoutrosData. 25 highest scoring network structures for the data by Boutros et al. [file 1471-2105-8-386-S1.gz › nem/..Rcheck/nem/html/prune.graph.html]

R: Prunes spurious edges in a phenotypic hierarchy

|  |  |
| --- | --- |
| prune.graph {nem} | R Documentation |

## Prunes spurious edges in a phenotypic hierarchy

### Description

A heuristic to prune spurious edges in a pehnotypic hierarchy

### Usage

```
prune.graph(g,cutIN=NULL,cutOUT=NULL,quant=.95,verbose=TRUE)
```

### Arguments

|  |  |
| --- | --- |
| `g` | an adjacency matrix or a 'graphNEL' object |
| `cutIN` | minimum number of missing in-edges required to cut all in-edges. Default |
| `cutOUT` | minimum number of missing out-edges required to cut all out-edges |
| `quant` | if 'cutIN' or 'cutOUT' are not assigned, a quantile 'quant' of the distribution of missing in- or out-edges for all nodes is used |
| `verbose` | Default: TRUE |

### Details

`prune.graph` provides a heuristic approach to prune surious edges.
`prune.graph` compares the input graph to its transitive closure, and counts for each node how many incoming and outgoing edges are missing.
If the number is bigger than a user-defined cutoff, all incoming (outgoing) edges are removed.

### Value

|  |  |
| --- | --- |
| `graph` | the pruned phenotypic hierarchy (a 'graphNEL' object) |
| `removed` | number of removed edges |
| `missing.in` | number of missing in-edges for each node |
| `missing.out` | number of missing out-edges for each node |

### Author(s)

Florian Markowetz <URL: http://genomics.princeton.edu/~florian>

### Examples

```
    # a transitively closed core with two spurious edges
    g <- matrix(0,5,5)
    g[1,2] <- 1
    g[2,c(3,4)] <- 1
    g[3,4] <- 1
    g[4,5] <- 1
    dimnames(g) <- list(LETTERS[1:5],LETTERS[1:5])
    g <- as(g,"graphNEL")
    
    # prune graph
    gP <- prune.graph(g)
    
    # plot
    par(mfrow=c(1,2))
    plot(g,main="two spurious edges")
    plot(gP$graph,main="pruned")
```

---

[Package *nem* version 1.4.2 Index]
