# Supplementary material for: Large scale statistical inference of signaling pathways from RNAi and microarray data
Source: BMC Bioinformatics. 2007 Oct 15;8:386. doi: 10.1186/1471-2105-8-386 (PMC2241646; doi:10.1186/1471-2105-8-386)
Supplement: Additional file 1 — top25solutionsBoutrosData. 25 highest scoring network structures for the data by Boutros et al. [file 1471-2105-8-386-S1.gz › nem/..Rcheck/nem/html/transitive.reduction.html]

R: Computes the transitive reduction of a graph

|  |  |
| --- | --- |
| transitive.reduction {nem} | R Documentation |

## Computes the transitive reduction of a graph

### Description

`transitive.reduction` removes direct edges, which can be explained by another path in the graph.

### Usage

```
transitive.reduction(g)
```

### Arguments

|  |  |
| --- | --- |
| `g` | graphNEL object |

### Details

`transitive.reduction` implements an interative algorithm in spirit of Wagner (2001).

### Value

returns a graph object with shortcuts removed

### Author(s)

Florian Markowetz <URL: http://genomics.princeton.edu/~florian>

### References

Wagner A, How to reconstruct a large genetic network from $n$ gene perturbations in fewer than $n^2$ easy steps. Bioinformatics 17(12): 1183-1197 (2001)

### See Also

`transitive.closure`

### Examples

```
   V <- LETTERS[1:3]
   edL <- list(A=list(edges=c("B","C")),B=list(edges="C"),C=list(edges=NULL))
   gc <- new("graphNEL",nodes=V,edgeL=edL,edgemode="directed")
   g <- transitive.reduction(gc)
    
   par(mfrow=c(1,2))
   plot(gc,main="shortcut A->C")
   plot(g,main="shortcut removed")
```

---

[Package *nem* version 1.4.2 Index]
