# Supplementary material for: Large scale statistical inference of signaling pathways from RNAi and microarray data
Source: BMC Bioinformatics. 2007 Oct 15;8:386. doi: 10.1186/1471-2105-8-386 (PMC2241646; doi:10.1186/1471-2105-8-386)
Supplement: Additional file 1 — top25solutionsBoutrosData. 25 highest scoring network structures for the data by Boutros et al. [file 1471-2105-8-386-S1.gz › nem/..Rcheck/nem/html/score.html]

R: Computes the marginal likelihood of phenotypic hierarchies

|  |  |
| --- | --- |
| score {nem} | R Documentation |

## Computes the marginal likelihood of phenotypic hierarchies

### Description

Function to compute the marginal likelihood of a set of phenotypic hierarchies.

### Usage

```
score(models, D, type="mLL", para=NULL, hyperpara=NULL, Pe=NULL, Pm=NULL, lambda=0, selEGenes=FALSE, verbose=TRUE)

# S3 metehods for class 'score'
print.score(x, ...)

PhiDistr(Phi, Pm, a=4, b=0.1)
```

### Arguments

|  |  |
| --- | --- |
| `models` | a list of adjacency matrices with unit main diagonal |
| `D` | data matrix. Columns correspond to the nodes in the silencing scheme. Rows are effect reporters. |
| `type` | (1.) marginal likelihood "mLL" (only for cout matrix D), or (2.) full marginal likelihood "FULLmLL" integrated over a and b and depending on hyperparameters a0, a1, b0, b1 (only for count matrix D), or (3.) "CONTmLL" marginal likelihood for probability matrices, or (4.) "CONTmLLDens" marginal likelihood for probability density matrices |
| `para` | Vector with parameters `a` and `b` (for "mLL" with count data) |
| `hyperpara` | Vector with hyperparameters `a0`, `b0`, `a1`, `b1` for "FULLmLL" |
| `Pe` | prior position of effect reporters. Default: uniform over nodes in silencing scheme |
| `Pm` | prior on model graph (n x n matrix) with entries 0 <= priorPhi[i,j] <= 1 describing the probability of an edge between gene i and gene j. |
| `lambda` | regularization parameter to incorporate prior assumptions. |
| `selEGenes` | optimize selection of E-genes for each model |
| `verbose` | output while running or not |
| `x` | nem object |
| `...` | other arguments to pass |
| `Phi` | adjacency matrix |
| `a` | parameter of the inverse gamma prior for v=1/lambda |
| `b` | parameter of the inverse gamma prior for v=1/lambda |

### Details

Scoring models by marginal log-likelihood is implemented in function
`score`. Input consists of models and data, the type of the score
(`"mLL"`, `"FULLmLL"`, `"CONTmLL"` or `"CONTmLLDens"`), the corresponding paramters
(`para`) or hyperparameters (`hyperpara`), a prior for phenotype
positions (`Pe`) and model structures `Pm` with regularization parameter `lambda`. If a structure prior `Pm` is provided, but no regularization parameter `lambda`, Bayesian model averaging with an inverse gamma prior on 1/lambda is performed.

`score` is usually called within function `nem`.

### Value

|  |  |
| --- | --- |
| `graph` | the model with highest marginal likelihood (graphNEL object) |
| `mLL` | vector of marginal likelihoods for all models |
| `ppost` | vector of posterior probabilities for all models |
| `pos` | a list of estimated positions of effect reporters for each model |
| `mappos` | a list of maximum aposteriori estimates of effect positions for each model |
| `type` | as used in function call |
| `para` | as used in function call |
| `hyperpara` | as used in function call |
| `lambda` | as in function call |

### Author(s)

Florian Markowetz <URL: http://genomics.princeton.edu/~florian>

### References

[1]
:   Markowetz F, Bloch J, Spang R, Non-transcriptional pathway features reconstructed from secondary effects of RNA interference, Bioinformatics, 2005

[2]
:   Markowetz F, Probabilistic Models for Gene Silencing Data, PhD thesis, Free University Berlin, 2006

### See Also

`nem`, `mLL`, `FULLmLL`, `enumerate.models`

### Examples

```
   # Drosophila RNAi and Microarray Data from Boutros et al, 2002
   data("BoutrosRNAi2002")
   D <- BoutrosRNAiDiscrete[,9:16]

   # enumerate all possible models for 4 genes
   models <- enumerate.models(unique(colnames(D)))

   # score models with marginal likelihood
   result <- score(models,D,type="mLL",para=c(.13,.05))
   
   # plot graph
   plot(result,what="graph")

   # plot scores
   plot(result,what="mLL") 
    
   # plot posterior of E-gene positions
   plot(result,what="pos")
   
   # MAP estimate of effect positions
   result$mappos[[which.max(result$mLL)]]
```

---

[Package *nem* version 1.4.2 Index]
