# Supplementary material for: Large scale statistical inference of signaling pathways from RNAi and microarray data
Source: BMC Bioinformatics. 2007 Oct 15;8:386. doi: 10.1186/1471-2105-8-386 (PMC2241646; doi:10.1186/1471-2105-8-386)
Supplement: Additional file 1 — top25solutionsBoutrosData. 25 highest scoring network structures for the data by Boutros et al. [file 1471-2105-8-386-S1.gz › nem/..Rcheck/nem/html/transitive.closure.html]

R: Computes the transitive closure of a directed graph

|  |  |
| --- | --- |
| transitive.closure {nem} | R Documentation |

## Computes the transitive closure of a directed graph

### Description

Computes the transitive closure of a graph. Introduces a direct edge whenever
there is a path between two nodes in a digraph.

### Usage

```
transitive.closure(g, mat=FALSE, loops=TRUE)
```

### Arguments

|  |  |
| --- | --- |
| `g` | graphNEL object or adjacency matrix. |
| `mat` | convert result to adjacency matrix. |
| `loops` | Add loops from each node to itself? |

### Details

This function calculates the transitive closure of a given
graph.
If the input graph is a 'graphNEL' object, `transitive.reduction` is essentially a wrapper around the `RBGL::transitive.closure`,
to make its output more convenient for our purpose.
If the input is an adjacency matrix we use the matrix exponential for a quicker way to find the transitive closure.

### Value

returns a graphNEL object or adjacency matrix

### Author(s)

Florian Markowetz <URL: http://genomics.princeton.edu/~florian>

### See Also

`enumerate.models`, `transitive.reduction`

### Examples

```
   V <- LETTERS[1:3]
   edL <- list(A=list(edges="B"),B=list(edges="C"),C=list(edges=NULL))
   g <- new("graphNEL",nodes=V,edgeL=edL,edgemode="directed")
   gc <- transitive.closure(g,loops=FALSE)
    
   par(mfrow=c(1,2))
   plot(g,main="NOT transitively closed")
   plot(gc,main="transitively closed")
```

---

[Package *nem* version 1.4.2 Index]
