# Supplementary material for: Large scale statistical inference of signaling pathways from RNAi and microarray data
Source: BMC Bioinformatics. 2007 Oct 15;8:386. doi: 10.1186/1471-2105-8-386 (PMC2241646; doi:10.1186/1471-2105-8-386)
Supplement: Additional file 1 — top25solutionsBoutrosData. 25 highest scoring network structures for the data by Boutros et al. [file 1471-2105-8-386-S1.gz › nem/..Rcheck/nem/html/plot.effects.html]

R: Plots data according to a phenotypic hierarchy

|  |  |
| --- | --- |
| plot.effects {nem} | R Documentation |

## Plots data according to a phenotypic hierarchy

### Description

`plot.effects` visualizes the subset structure in the data by reordering rows and columns according to the topological order given by a phenotypic hierarchy.

### Usage

```
plot.effects(x,nem,border=TRUE,...)
```

### Arguments

|  |  |
| --- | --- |
| `x` | data matrix |
| `nem` | phenotypic hierarchy (object of class 'score' or 'pairwise') |
| `border` | draw red lines to indicate gene-specific effect reporters. Default: TRUE |
| `...` | additional parameters for the plotting function |

### Details

The experiments in the columns are reordered according to the topological order given by a phenotypic hierarchy.
The effect reporters in the rows are grouped together by their position in the hierarchy.
The groups are then arranged by topological order.
Within each group the rows are hierarchically clustered.

### Author(s)

Florian Markowetz <URL: http://genomics.princeton.edu/~florian>

### Examples

```
   data("BoutrosRNAi2002")
   D <- BoutrosRNAiDiscrete[,9:16]
   res <- nem(D,para=c(.13,.05))
   plot.effects(D,res)
```

---

[Package *nem* version 1.4.2 Index]
