# Supplementary material for: Large scale statistical inference of signaling pathways from RNAi and microarray data
Source: BMC Bioinformatics. 2007 Oct 15;8:386. doi: 10.1186/1471-2105-8-386 (PMC2241646; doi:10.1186/1471-2105-8-386)
Supplement: Additional file 1 — top25solutionsBoutrosData. 25 highest scoring network structures for the data by Boutros et al. [file 1471-2105-8-386-S1.gz › nem/..Rcheck/nem/html/network.AIC.html]

R: AIC criterion for network graph

|  |  |
| --- | --- |
| network.AIC {nem} | R Documentation |

## AIC criterion for network graph

### Description

calclate AIC for a given network graph (should be transitively closed)

### Usage

```
        network.AIC(network,k=2,verbose=TRUE)
```

### Arguments

|  |  |
| --- | --- |
| `network` | a nem object (e.g. 'pairwise') |
| `k` | penalty per parameter in the AIC calculation. Default = 2 for classical AIC |
| `verbose` | print out the result |

### Details

For k = log(n) the BIC (Schwarz criterion) is computed. Usually this function is not called directly but from `nemModelSelection`

### Value

AIC value

### Author(s)

Holger Froehlich

### See Also

`nemModelSelection`

### Examples

```
   data("BoutrosRNAi2002") 
   res1 <- moduleNetwork(BoutrosRNAiDiscrete[,9:16],para=c(.13,.05))
   network.AIC(res1)
   res2 <- moduleNetwork(BoutrosRNAiDiscrete[,9:16],para=c(.13,.05),Pm=matrix(0,ncol=4,nrow=4),lambda=10)
   network.AIC(res2)
```

---

[Package *nem* version 1.4.2 Index]
