# Supplementary material for: Large scale statistical inference of signaling pathways from RNAi and microarray data
Source: BMC Bioinformatics. 2007 Oct 15;8:386. doi: 10.1186/1471-2105-8-386 (PMC2241646; doi:10.1186/1471-2105-8-386)
Supplement: Additional file 1 — top25solutionsBoutrosData. 25 highest scoring network structures for the data by Boutros et al. [file 1471-2105-8-386-S1.gz › nem/..Rcheck/nem/html/enumerate.models.html]

R: Exhaustive enumeration of models

|  |  |
| --- | --- |
| enumerate.models {nem} | R Documentation |

## Exhaustive enumeration of models

### Description

The function `enumerate.models` is used to create the model space for inference by exhaustive enumeration.
It computes a list of all transitively closed directed graphs on a given number of nodes.

### Usage

```
enumerate.models(x,name=NULL,verbose=TRUE)
```

### Arguments

|  |  |
| --- | --- |
| `x` | either the number of nodes or a vector of node names. |
| `name` | optionally the nodenames, if they are not provided in x |
| `verbose` | if TRUE outputs number of (unique) models. Default: TRUE |

### Details

The model space of Nested Effects Models consists of all transitively closed directed graphs.
The function `enumerate.models` creates them in three steps: (1.) build all directed graphs on `x` (or `length(x)`) nodes,
(2.) transitively close each one of them, and (3.) remove redundant models to yield a unique set.
So far, enumeration is limited to up to 5 nodes.

I'm aware that this is inefficient!
It would be very desirable to enumerate the models directly (i.e. without creating all directed graphs as an intermediate step).

### Value

a list of models. Each entry is a transitively closed adjacency matrix with unit main diagonal.

### Author(s)

Florian Markowetz <URL: http://genomics.princeton.edu/~florian>

### See Also

`score`, `nem`

### Examples

```
enumerate.models(2)
enumerate.models(c("Anna","Bert"))
```

---

[Package *nem* version 1.4.2 Index]
