# Supplementary material for: Large scale statistical inference of signaling pathways from RNAi and microarray data
Source: BMC Bioinformatics. 2007 Oct 15;8:386. doi: 10.1186/1471-2105-8-386 (PMC2241646; doi:10.1186/1471-2105-8-386)
Supplement: Additional file 1 — top25solutionsBoutrosData. 25 highest scoring network structures for the data by Boutros et al. [file 1471-2105-8-386-S1.gz › nem/..Rcheck/nem/html/mLL.html]

R: Marginal likelihood of a phenotypic hierarchy

|  |  |
| --- | --- |
| mLL {nem} | R Documentation |

## Marginal likelihood of a phenotypic hierarchy

### Description

computes the marginal likelihood of observed phenotypic data given a phenotypic hierarchy.

### Usage

```
mLL(Phi,D1,D0=NULL,a=0.05,b=0.15,Pe=NULL,Pm=NULL,lambda=0,type="mLL")
```

### Arguments

|  |  |
| --- | --- |
| `Phi` | an adjacency matrix with unit main diagonal |
| `D1` | (i) count matrix for discrete data: phenotypes x genes. How often did we see an effect after interventions? (ii) matrix describing the probabilities of an effect (iii) probability density matrix discribing the strength of an effect |
| `D0` | count matrix: phenotypes x genes. How often did we NOT see an effect after intervention? Not used for continious data |
| `a` | false positive rate: how probable is it to miss an effect? (for count matrix) |
| `b` | false negative rate: how probable is it to see a spurious effect? (for count matrix) |
| `Pe` | prior of effect reporter positions in the phenotypic hierarchy |
| `Pm` | prior on model graph (n x n matrix) with entries 0 <= priorPhi[i,j] <= 1 describing the probability of an edge between gene i and gene j. |
| `lambda` | regularization parameter to incorporate prior assumptions. |
| `type` | (i) "mLL" = marginal likelihood using count matrices (ii) "CONTmLL" = marginal likelihood for probability matrices (iii) "CONTmLLDens" = marginal likelihood for probability density matrices |

### Details

It computes the marginal likelihood of a single phenotypic hierarchy.
Usually called from within the function `score`.

### Value

|  |  |
| --- | --- |
| `mLL` | marginal likelihood of a phenotypic hierarchy |
| `pos` | posterior distribution of effect positions in the hierarchy |
| `mappos` | Maximum aposteriori estimate of effect positions |

### Author(s)

Florian Markowetz <URL: http://genomics.princeton.edu/~florian>

### References

Markowetz F, Bloch J, Spang R, Non-transcriptional pathway features reconstructed from secondary effects of RNA interference, Bioinformatics, 2005

### See Also

`score`, `FULLmLL`

### Examples

```
   data("BoutrosRNAi2002")
   result <- nem(BoutrosRNAiDiscrete[,9:16],type="mLL",para=c(.15,.05))
```

---

[Package *nem* version 1.4.2 Index]
