# Supplementary material for: Large scale statistical inference of signaling pathways from RNAi and microarray data
Source: BMC Bioinformatics. 2007 Oct 15;8:386. doi: 10.1186/1471-2105-8-386 (PMC2241646; doi:10.1186/1471-2105-8-386)
Supplement: Additional file 1 — top25solutionsBoutrosData. 25 highest scoring network structures for the data by Boutros et al. [file 1471-2105-8-386-S1.gz › nem/..Rcheck/nem/html/FULLmLL.html]

R: Full marginal likelihood of a phenotypic hierarchy

|  |  |
| --- | --- |
| FULLmLL {nem} | R Documentation |

## Full marginal likelihood of a phenotypic hierarchy

### Description

The function the full marginal likelihood of a phenotypic hierarchy.
The full marginal likelihood equals the marginal likelihood `mLL`
averaged over the error probabilities $α$ and $β$.

### Usage

```
FULLmLL(Phi, D1, D0, a0, b0, a1, b1, Pe, Pm=NULL, lambda=0)
```

### Arguments

|  |  |
| --- | --- |
| `Phi` | an adjacency matrix with unit main diagonal |
| `D1` | count matrix: phenotypes x genes. How often did we see an effect after interventions? |
| `D0` | count matrix: phenotypes x genes. How often did we NOT see an effect after intervention? |
| `a0, b0, a1, b1` | Hyperparameters |
| `Pe` | prior of effect positions in the hierarchy. A matrix of size phenotypes x genes, where each row contains positive numbers summing to 1. |
| `Pm` | prior on model graph (n x n matrix) with entries 0 <= priorPhi[i,j] <= 1 describing the probability of an edge between gene i and gene j. |
| `lambda` | regularization parameter to incorporate prior assumptions. |

### Details

Additionally to the marginal likelihood introduced in Markowetz et al (2005),
we can average over the error probabilities $α$ and $β$ assuming Beta priors.
The parameters of the two Beta priors are hyperparameters of the full marginal likelihood score.
The four hyperparameters fall into two categories:
`a1` and `b0` are weights for observing the predicted state, while `a0` and `b1`
are weights for observing errors.
We suggest setting `a1=b0` and `a0=b1`.
The ratio between the two values should correspond to our assessment of the noise level.
See the example section for an application.
The function `FULLmLL` is usually called from within function `score`.

### Value

|  |  |
| --- | --- |
| `mLL` | full marginal likelihood of a model |
| `pos` | posterior distribution of effect positions in the hierarchy |
| `mappos` | maximum aposteriori estimate of effect positions |

### Author(s)

Florian Markowetz <URL: http://genomics.princeton.edu/~florian>

### References

Markowetz F, Probabilistic Models for Gene Silencing
Data. PhD thesis, Free University Berlin, 2006.

### See Also

`score`, `mLL`

### Examples

```
   data("BoutrosRNAi2002")
   res <- nem(BoutrosRNAiDiscrete[,9:16],type="FULLmLL",hyperpara=c(1,9,9,1))
```

---

[Package *nem* version 1.4.2 Index]
