# Supplementary material for: Large scale statistical inference of signaling pathways from RNAi and microarray data
Source: BMC Bioinformatics. 2007 Oct 15;8:386. doi: 10.1186/1471-2105-8-386 (PMC2241646; doi:10.1186/1471-2105-8-386)
Supplement: Additional file 1 — top25solutionsBoutrosData. 25 highest scoring network structures for the data by Boutros et al. [file 1471-2105-8-386-S1.gz › nem/..Rcheck/nem/html/local.model.prior.html]

R: Computes a prior to be used for edge-wise model inference

|  |  |
| --- | --- |
| local.model.prior {nem} | R Documentation |

## Computes a prior to be used for edge-wise model inference

### Description

The function `pairwise.posterior` infers a phenotypic hierarchy edge by edge by
choosing between four models (unconnected, subset, superset, undistinguishable).
For each edge, `local.model.prior` computes a prior distribution over the four models.
It can be used to ensure sparsity of the graph and high confidence in results.

### Usage

```
local.model.prior(size,n,bias)
```

### Arguments

|  |  |
| --- | --- |
| `size` | expected number of edges in the graph. |
| `n` | number of perturbed genes in the dataset, number of nodes in the graph |
| `bias` | the factor by which the double-headed edge is preferred over the single-headed edges |

### Details

A graph on `n` nodes has `N=n*(n-1)/2` possible directed edges (one- or bi-directional).
If each edge occurs with probability $p$, we expect to see $Np$ edges in the graph.
The function `local.model.prior` takes the number of genes (`n`) and the
expected number of edges (`size`) as an input and computes a prior distribution
for edge occurrence: no edge with probability `size/N`, and the probability for
edge existence being split over the three edge models with a bias towards the conservative
double-headed model specified by `bias`. To ensure sparsity, the `size` should
be chosen small compared to the number of possible edges.

### Value

a distribution over four states: a vector of four positive real numbers summing to one

### Note

### Author(s)

Florian Markowetz <URL: http://genomics.princeton.edu/~florian>

### References

### See Also

`pairwise.posterior`, `nem`

### Examples

```
# uniform over the 3 edge models
local.model.prior(4,4,1)
# bias towards <->
local.model.prior(4,4,2)
```

---

[Package *nem* version 1.4.2 Index]
