# Supplementary material for: Large scale statistical inference of signaling pathways from RNAi and microarray data
Source: BMC Bioinformatics. 2007 Oct 15;8:386. doi: 10.1186/1471-2105-8-386 (PMC2241646; doi:10.1186/1471-2105-8-386)
Supplement: Additional file 1 — top25solutionsBoutrosData. 25 highest scoring network structures for the data by Boutros et al. [file 1471-2105-8-386-S1.gz › nem/..Rcheck/nem/html/BoutrosRNAi2002.html]

R: RNAi data on Drosophila innate immune response

|  |  |
| --- | --- |
| BoutrosRNAi2002 {nem} | R Documentation |

## RNAi data on Drosophila innate immune response

### Description

Data from a study on innate immune response in {em Drosophila} (Boutros
et al, 2002). Selectively removing signaling components by RNAi blocked induction of all, or only parts, of the
transcriptional response to LPS. The nested structure of perturbation effects allows to reconstruct a
branching in the Imd pathway.

### Usage

```
data(BoutrosRNAi2002)
```

### Format

BoutrosRNAiExpression: data matrix: 14010 x 16
BoutrosRNAiDiscrete: binary matrix: 68 x 16

### Details

The dataset consists of 16 Affymetrix-microarrays: 4 replicates of control
experiments without LPS and without RNAi (negative controls), 4 replicates of
expression profiling after stimulation with LPS but without RNAi (positive
controls), and 2 replicates each of expression profiling after applying LPS and
silencing one of the four candidate genes tak, key, rel, and mkk4/hep.

`BoutrosRNAiExpression`: For preprocessing we performed normalization on probe level using a variance
stabilizing transformation (Huber et al, 2002), and probe set summarization
using a median polish fit of an additive model (Irizarry et al, 2003).

`BoutrosRNAiDiscrete`: contains only the 68 genes more than two-fold up-regulated between negative and positive controls.
The continuous expression values are discretized to `1` (effect: closer to negative controls) and `0` (no effect: closer to positive controls).

### References

Boutros M, Agaisse H, Perrimon N, Sequential activation of signaling pathways
during innate immune responses in Drosophila. Developmental
Cell. 3(5):711-722, 2002

### See Also

`nem.discretize`

### Examples

```
    data("BoutrosRNAi2002")
    dim(BoutrosRNAiExpression)
    dim(BoutrosRNAiDiscrete)
```

---

[Package *nem* version 1.4.2 Index]
