# Supplementary material for: Large scale statistical inference of signaling pathways from RNAi and microarray data
Source: BMC Bioinformatics. 2007 Oct 15;8:386. doi: 10.1186/1471-2105-8-386 (PMC2241646; doi:10.1186/1471-2105-8-386)
Supplement: Additional file 1 — top25solutionsBoutrosData. 25 highest scoring network structures for the data by Boutros et al. [file 1471-2105-8-386-S1.gz › nem/..Rcheck/nem/html/nem.html]

R: Nested Effects Models - main function

|  |  |
| --- | --- |
| nem {nem} | R Documentation |

## Nested Effects Models - main function

### Description

The main function to infer a phenotypic hierarchy from data

### Usage

```
nem(D,inference="pairwise",models=NULL,type="mLL",para=NULL,hyperpara=NULL,Pe=NULL,Pm=NULL,Pmlocal=NULL,local.prior.size=length(unique(colnames(D))),local.prior.bias=1,triples.thrsh=0.5,lambda=0,selEGenes=FALSE,verbose=TRUE)
```

### Arguments

|  |  |
| --- | --- |
| `D` | data matrix with experiments in the columns (binary or continious) |
| `inference` | `search` to use exhaustive enumeration; or `triples` for triple-based inference; or `pairwise` for the pairwise heuristic; or `ModuleNetwork` for the module based inference |
| `models` | a list of adjacency matrices for model search. If NULL, `enumerate.models` is used for exhaustive enumeration of all possible models. |
| `type` | `mLL` or `FULLmLL` or `CONTmLL` or `CONTmLLDens` |
| `para` | vector of length two: false positive rate and false negative rate for non-binary data. Used by `mLL()` |
| `hyperpara` | vector of length four: used by `FULLmLL()` for binary data |
| `Pe` | prior of effect reporter positions in the phenotypic hierarchy (same dimension as D) |
| `Pm` | prior over models (n x n matrix) |
| `Pmlocal` | local model prior for pairwise and triple learning. For pairwise learning generated by `local.model.prior()` according to arguments `local.prior.size` and `local.prior.bias` |
| `local.prior.size` | prior expected number of edges in the graph (for pairwise learning) |
| `local.prior.bias` | bias towards double-headed edges. Default: 1 (no bias; for pairwise learning) |
| `triples.thrsh` | threshold for model averaging to combine triple models for each edge |
| `lambda` | regularization parameter to incorporate prior assumptions. Default: 0 (no regularization) |
| `selEGenes` | optimize selection of E-genes for each model |
| `verbose` | do you want to see progression statements" Default: TRUE |

### Details

`nem` is an interface to the functions `score()`, `pairwise.posterior()`, `triple.posterior`, `moduleNetwork`.

`plot.nem` plots the inferred phenotypic hierarchy as a directed graph, the likelihood distribution of the models (only for exhaustive search) or the posterior position of the effected genes.

### Value

An object of class 'score' or 'pairwise' or 'triples' or 'ModuleNetwork' containing slots

|  |  |
| --- | --- |
| `graph` | the inferred phenotypic hierarchy |
| `pos` | posterior distribution of positions of effect reporters |
| `mappos` | estimated position of effects in the phenotypic hierarchy |
| `type` | see above |
| `para` | see above |
| `hyperpara` | see above |
| `lambda` | see above |

and additional ones according to the function used for inference.

### Author(s)

Florian Markowetz <URL: http://genomics.princeton.edu/~florian>

### See Also

`score`, `moduleNetwork`, `triples.posterior`, `pairwise.posterior`, `local.model.prior`, `enumerate.models`, `plot.nem`

### Examples

```
   data("BoutrosRNAi2002")
   D <- BoutrosRNAiDiscrete[,9:16]
   p <- c(.13,.05)
   res1 <- nem(D,para=p,inference="search")
   res2 <- nem(D,para=p,inference="pairwise")
   res3 <- nem(D,para=p,inference="triples")
   res4 <- nem(D,para=p,inference="ModuleNetwork")
   

   par(mfrow=c(1,4))
   plot(res1,main="exhaustive search")
   plot(res2,main="pairs")
   plot(res3,main="triples")
   plot(res4,main="module network")
```

---

[Package *nem* version 1.4.2 Index]
