# Supplementary material for: Large scale statistical inference of signaling pathways from RNAi and microarray data
Source: BMC Bioinformatics. 2007 Oct 15;8:386. doi: 10.1186/1471-2105-8-386 (PMC2241646; doi:10.1186/1471-2105-8-386)
Supplement: Additional file 1 — top25solutionsBoutrosData. 25 highest scoring network structures for the data by Boutros et al. [file 1471-2105-8-386-S1.gz › nem/..Rcheck/nem/html/triples.posterior.html]

R: Infers a phenotypic hierarchy from triples

|  |  |
| --- | --- |
| triples.posterior {nem} | R Documentation |

## Infers a phenotypic hierarchy from triples

### Description

Function `triples.posterior` estimates the hierarchy triple-wise. In each step only a triple of nodes
is involved and no exhaustive enumeration of model space is needed as in function `score`.

### Usage

```
triples.posterior(D, type="mLL",para=NULL, hyperpara=NULL,Pe=NULL,Pmlocal=NULL,Pm=NULL,lambda=0, triples.thrsh=.5, selEGenes=FALSE, verbose=TRUE)

#S3 methods for class 'triples'
print.triples(x,...)
```

### Arguments

|  |  |
| --- | --- |
| `D` | data matrix. Columns correspond to the nodes in the silencing scheme. Rows are phenotypes. |
| `type` | (1.) marginal likelihood "mLL" (only for cout matrix D), or (2.) full marginal likelihood "FULLmLL" integrated over a and b and depending on hyperparameters a0, a1, b0, b1 (only for count matrix D), or (3.) "CONTmLL" marginal likelihood for probability matrices, or (4.) "CONTmLLDens" marginal likelihood for probability density matrices |
| `para` | vector with parameters a and b for "mLL", if count matrices are used |
| `hyperpara` | vector with hyperparameters a0, b0, a1, b1 for "FULLmLL" |
| `Pe` | prior position of effect reporters. Default: uniform over nodes in hierarchy |
| `Pmlocal` | local model prior for the four models tested at each node: a vector of length 4 with positive entries summing to one |
| `triples.thrsh` | threshold used when combining tripel models for each edge. Default: only edges appearing in more than half of triples are included in the final graph. |
| `Pm` | prior on model graph (n x n matrix) with entries 0 <= priorPhi[i,j] <= 1 describing the probability of an edge between gene i and gene j. |
| `lambda` | regularization parameter to incorporate prior assumptions. |
| `selEGenes` | optimize selection of E-genes for each model |
| `verbose` | do you want to see progress statements printed or not? Default: TRUE |
| `x` | nem object |
| `...` | other arguments to pass |

### Details

`triples.posterior` is an alternative to exhaustive search
by the function `score` and more accurate than `pairwise.posterior`.
For each triple of perturbed genes
it chooses between the 29 possible models. It then uses model averaging to combine the triple-models into a final graph.

`print.triples` gives an overview over the 'triples' object.

### Value

|  |  |
| --- | --- |
| `graph` | the inferred directed graph (graphNEL object) |
| `avg` | matrix of edge frequencies in triple models |
| `pos` | posterior over effect positions |
| `mappos` | MAP estimate of effect positions |
| `type` | as used in function call |
| `para` | as used in function call |
| `hyperpara` | as used in function call |
| `lambda` | as in function call |

### Author(s)

Florian Markowetz <URL: http://genomics.princeton.edu/~florian>

### See Also

`score`, `nem`

### Examples

```
   data("BoutrosRNAi2002") 
   res <- triples.posterior(BoutrosRNAiDiscrete[,9:16],para=c(.13,.05))
   
   # plot graph
   plot(res,what="graph")
   
   # plot posterior over effect positions
   plot(res,what="pos")
   
   # estimate of effect positions
   res$mappos
```

---

[Package *nem* version 1.4.2 Index]
