# Supplementary material for: Large scale statistical inference of signaling pathways from RNAi and microarray data
Source: BMC Bioinformatics. 2007 Oct 15;8:386. doi: 10.1186/1471-2105-8-386 (PMC2241646; doi:10.1186/1471-2105-8-386)
Supplement: Additional file 1 — top25solutionsBoutrosData. 25 highest scoring network structures for the data by Boutros et al. [file 1471-2105-8-386-S1.gz › nem/..Rcheck/nem/html/BFSlevel.html]

R: Build (generalized) hierarchy by Breath-First Search

|  |  |
| --- | --- |
| BFSlevel {nem} | R Documentation |

## Build (generalized) hierarchy by Breath-First Search

### Description

`BFSlevel` builds a (generalized) hierarchy by Breath-First Search as described in (Yu and Gerstein, 2006)

### Usage

```
BFSlevel(g,verbose=TRUE)
```

### Arguments

|  |  |
| --- | --- |
| `g` | graphNEL object |
| `verbose` | Default: TRUE |

### Details

Haiyuan Yu and Mark Gerstein: Genomic analysis of the hierarchical structure of regulatory networks, PNAS 103(40):14724-14731, 2006

### Value

|  |  |
| --- | --- |
| `level` | vector of levels for each node |

### Author(s)

Florian Markowetz <URL: http://genomics.princeton.edu/~florian>

### See Also

### Examples

```
 ## bla
```

---

[Package *nem* version 1.4.2 Index]
