# Supplementary material for: Large scale statistical inference of signaling pathways from RNAi and microarray data
Source: BMC Bioinformatics. 2007 Oct 15;8:386. doi: 10.1186/1471-2105-8-386 (PMC2241646; doi:10.1186/1471-2105-8-386)
Supplement: Additional file 1 — top25solutionsBoutrosData. 25 highest scoring network structures for the data by Boutros et al. [file 1471-2105-8-386-S1.gz › nem/..Rcheck/nem/html/plot.nem.html]

R: plot nested effect model

|  |  |
| --- | --- |
| plot.nem {nem} | R Documentation |

## plot nested effect model

### Description

plot graph of nested effect model, the marginal likelihood distribution or the posterior position of the effected genes

### Usage

```
        plot.nem(x, what="graph", remove.singletons=FALSE, PDF=FALSE, filename="nemplot.pdf", thresh=0, transitiveReduction=FALSE, ...)
```

### Arguments

|  |  |
| --- | --- |
| `x` | nem object to plot |
| `what` | (i), "graph", (ii) "mLL" = likelihood distribution, (iii) "pos" = posterior position of effected genes |
| `remove.singletons` | remove unconnected nodes from the graph plot |
| `PDF` | output as PDF-file |
| `filename` | filename of PDF-file |
| `thresh` | if x has a real valued adjacency matrix (weight matrix), don't plot edges with weight <= thresh |
| `transitiveReduction` | plot a transitively reduced graph |
| `...` | other arguments to pass |

### Value

none

### Author(s)

Florian Markowetz <URL: http://genomics.princeton.edu/~florian>

### See Also

`nem`, `score`

---

[Package *nem* version 1.4.2 Index]
