# Supplementary material for: Large scale statistical inference of signaling pathways from RNAi and microarray data
Source: BMC Bioinformatics. 2007 Oct 15;8:386. doi: 10.1186/1471-2105-8-386 (PMC2241646; doi:10.1186/1471-2105-8-386)
Supplement: Additional file 1 — top25solutionsBoutrosData. 25 highest scoring network structures for the data by Boutros et al. [file 1471-2105-8-386-S1.gz › nem/..Rcheck/nem/html/subsets.html]

R: Subsets

|  |  |
| --- | --- |
| subsets {nem} | R Documentation |

## Subsets

### Description

subsets

### Usage

```
subsets(n, r, v = 1:n, set = TRUE)
```

### Arguments

|  |  |
| --- | --- |
| `n` | bli |
| `r` | bla |
| `v` | blo |
| `set` | blu |

### Details

taken from the programmers corner of some R-News issue by Dennis

### Value

|  |  |
| --- | --- |
| `n` | bli |
| `r` | bla |
| `v` | blo |

### Author(s)

Dennis Kostka <URL: http://www.molgen.mpg.de/~kostka>

### See Also

### Examples

```
 ## bla
```

---

[Package *nem* version 1.4.2 Index]
