# Supplementary material for: Large scale statistical inference of signaling pathways from RNAi and microarray data
Source: BMC Bioinformatics. 2007 Oct 15;8:386. doi: 10.1186/1471-2105-8-386 (PMC2241646; doi:10.1186/1471-2105-8-386)
Supplement: Additional file 1 — top25solutionsBoutrosData. 25 highest scoring network structures for the data by Boutros et al. [file 1471-2105-8-386-S1.gz › nem/..Rcheck/nem/html/nem.discretize.html]

R: Discretize perturbation data according to control experiments

|  |  |
| --- | --- |
| nem.discretize {nem} | R Documentation |

## Discretize perturbation data according to control experiments

### Description

discretizes raw data to define effects of interventions with respect to wildtype/control measurements

### Usage

```
nem.discretize(D,neg.control=NULL,pos.control=NULL,nfold=2,cutoff=0:10/10, pCounts=20, empPval=.05, verbose=TRUE)
```

### Arguments

|  |  |
| --- | --- |
| `D` | matrix with experiments as columns and effect reporters as rows |
| `neg.control` | either indices of columns in `D` or a matrix with the same number of rows as `D` |
| `pos.control` | either indices of columns in `D` or a matrix with the same number of rows as `D` |
| `nfold` | fold-change between neg. and pos. controls for selecting effect reporters. Default: 2 |
| `cutoff` | a (vector of) cutoff value(s) weighting the pos. controls versus the neg. controls. Default: 0:10/10 |
| `pCounts` | pseudo-counts to guard against unreasonable low error estimates |
| `empPval` | empirical p-value cutoff for effects if only one control is available |
| `verbose` | Default: TRUE |

### Details

Chooses cutoff such that separation between negative and positive controls becomes optimal.

### Value

|  |  |
| --- | --- |
| `dat` | discretized data matrix |
| `pos` | discretized positive controls [in the two-controls setting] |
| `neg` | discretized negative controls [in the two-controls setting] |
| `sel` | effect reporters selected [in the two-controls setting] |
| `cutoff` | error rates for different cutoff values [in the two-controls setting] |
| `para` | estimated error rates [in the two-controls setting] |

### Note

preliminary! will be developed to be more generally applicable

### Author(s)

Florian Markowetz <URL: http://genomics.princeton.edu/~florian>

### References

Markowetz F, Bloch J, Spang R, Non-transcriptional pathway features reconstructed from secondary effects of RNA interference, Bioinformatics, 2005

### See Also

`BoutrosRNAi2002`

### Examples

```
   # discretize Boutros data as in
   # Markowetz et al, 2005
   data("BoutrosRNAi2002")
   disc <- nem.discretize(BoutrosRNAiExpression,neg.control=1:4,pos.control=5:8,cutoff=.7)
   stopifnot(disc$dat==BoutrosRNAiDiscrete[,9:16])
```

---

[Package *nem* version 1.4.2 Index]
