# Supplementary material for: Large scale statistical inference of signaling pathways from RNAi and microarray data
Source: BMC Bioinformatics. 2007 Oct 15;8:386. doi: 10.1186/1471-2105-8-386 (PMC2241646; doi:10.1186/1471-2105-8-386)
Supplement: Additional file 1 — top25solutionsBoutrosData. 25 highest scoring network structures for the data by Boutros et al. [file 1471-2105-8-386-S1.gz › nem/..Rcheck/nem/html/selectEGenes.html]

R: Automatic selection of most relevant S-genes

|  |  |
| --- | --- |
| getRelevantEGenes {nem} | R Documentation |

## Automatic selection of most relevant S-genes

### Description

Selects those E-genes, which have the highest likelihood under the given network hypothesis.

### Usage

```
getRelevantEGenes(Phi, D, nEgenes=min(5*ncol(Phi), nrow(D1)), type="mLL", para=NULL, hyperpara=NULL, Pe=NULL, Pm=NULL, lambda=0)

selectEGenes(Phi,D1,D0=NULL,para=NULL,hyperpara=NULL,Pe=NULL,Pm=NULL,lambda=0,type="mLL", nEgenes=min(5*ncol(Phi), nrow(D1)))
```

### Arguments

|  |  |
| --- | --- |
| `Phi` | adjacency matrix with unit main diagonal |
| `D` | data matrix. Columns correspond to the nodes in the silencing scheme. Rows are effect reporters. |
| `nEgenes` | no. of E-genes to select |
| `type` | (1.) marginal likelihood "mLL" (only for cout matrix D), or (2.) full marginal likelihood "FULLmLL" integrated over a and b and depending on hyperparameters a0, a1, b0, b1 (only for count matrix D), or (3.) "CONTmLL" marginal likelihood for probability matrices, or (4.) "CONTmLLDens" marginal likelihood for probability density matrices |
| `para` | Vector with parameters `a` and `b` (for "mLL" with count data) |
| `hyperpara` | Vector with hyperparameters `a0`, `b0`, `a1`, `b1` for "FULLmLL" |
| `Pe` | prior position of effect reporters. Default: uniform over nodes in silencing scheme |
| `Pm` | prior on model graph (n x n matrix) with entries 0 <= priorPhi[i,j] <= 1 describing the probability of an edge between gene i and gene j. |
| `lambda` | regularization parameter to incorporate prior assumptions. |
| `D1` | (i) count matrix for discrete data: phenotypes x genes. How often did we see an effect after interventions? (ii) matrix describing the probabilities of an effect (iii) probability density matrix discribing the strength of an effect |
| `D0` | count matrix: phenotypes x genes. How often did we NOT see an effect after intervention? Not used for continious data |

### Details

uses `mLL` or `FULLmLL` to score each E-gene.

### Value

|  |  |
| --- | --- |
| `mLL` | marginal likelihood of a phenotypic hierarchy |
| `pos` | posterior distribution of effect positions in the hierarchy |
| `mappos` | Maximum aposteriori estimate of effect positions |

### Author(s)

Holger Froehlich

### See Also

`nem`, `score`, `mLL`, `FULLmLL`, `enumerate.models`

### Examples

```
   # Drosophila RNAi and Microarray Data from Boutros et al, 2002
   data("BoutrosRNAi2002")
   D <- BoutrosRNAiDiscrete[,9:16]

   # enumerate all possible models for 4 genes
   models <- enumerate.models(unique(colnames(D)))  
   
   getRelevantEGenes(models[[64]], D, para=c(.13,.05))
```

---

[Package *nem* version 1.4.2 Index]
