# Supplementary material for: Large scale statistical inference of signaling pathways from RNAi and microarray data
Source: BMC Bioinformatics. 2007 Oct 15;8:386. doi: 10.1186/1471-2105-8-386 (PMC2241646; doi:10.1186/1471-2105-8-386)
Supplement: Additional file 1 — top25solutionsBoutrosData. 25 highest scoring network structures for the data by Boutros et al. [file 1471-2105-8-386-S1.gz › nem/..Rcheck/nem/html/moduleNetwork.html]

R: Infers a phenotypic hierarchy using the module network

|  |  |
| --- | --- |
| moduleNetwork {nem} | R Documentation |

## Infers a phenotypic hierarchy using the module network

### Description

Function `moduleNetwork` estimates the hierarchy using a divide and conquer approach. In each step only a subset of nodes (called module)
is involved and no exhaustive enumeration of model space is needed as in function `score`.

### Usage

```
moduleNetwork(D,type="mLL",Pe=NULL,Pm=NULL,lambda=0,para=NULL,hyperpara=NULL,selEGenes=FALSE,verbose=TRUE)

#S3 methods for class 'moduleNetwork'
print.ModuleNetwork(x,...)
```

### Arguments

|  |  |
| --- | --- |
| `D` | data matrix. Columns correspond to the nodes in the silencing scheme. Rows are phenotypes. |
| `type` | (1.) marginal likelihood "mLL" (only for cout matrix D), or (2.) full marginal likelihood "FULLmLL" integrated over a and b and depending on hyperparameters a0, a1, b0, b1 (only for count matrix D), or (3.) "CONTmLL" marginal likelihood for probability matrices, or (4.) "CONTmLLDens" marginal likelihood for probability density matrices |
| `Pe` | prior position of effect reporters. Default: uniform over nodes in hierarchy |
| `Pm` | prior on model graph (n x n matrix) with entries 0 <= priorPhi[i,j] <= 1 describing the probability of an edge between gene i and gene j. |
| `lambda` | regularization parameter to incorporate prior assumptions. |
| `para` | vector with parameters a and b for "mLL", if count matrices are used |
| `hyperpara` | vector with hyperparameters a0, b0, a1, b1 for "FULLmLL" |
| `selEGenes` | optimize selection of E-genes for each model |
| `verbose` | do you want to see progress statements printed or not? Default: TRUE |
| `x` | nem object |
| `...` | other arguments to pass |

### Details

`moduleNetwork` is an alternative to exhaustive search
by the function `score` and more accurate than `pairwise.posterior`.
It uses clustering to sucessively split the network into smaller modules, which can then be estimated completely. Connections between modules are estimated pairwise between nodes of two different modules.

### Value

|  |  |
| --- | --- |
| `graph` | the inferred directed graph (graphNEL object) |
| `pos` | posterior over effect positions |
| `mappos` | MAP estimate of effect positions |
| `type` | as used in function call |
| `para` | as used in function call |
| `hyperpara` | as used in function call |
| `lambda` | as in function call |

### Author(s)

Holger Froehlich

### See Also

`score`, `nem`

### Examples

```
   data("BoutrosRNAi2002") 
   res <- moduleNetwork(BoutrosRNAiDiscrete[,9:16],para=c(.13,.05))
   
   # plot graph
   plot(res,what="graph")
   
   # plot posterior over effect positions
   plot(res,what="pos")
   
   # estimate of effect positions
   res$mappos
```

---

[Package *nem* version 1.4.2 Index]
