# Supplementary material for: Large scale statistical inference of signaling pathways from RNAi and microarray data
Source: BMC Bioinformatics. 2007 Oct 15;8:386. doi: 10.1186/1471-2105-8-386 (PMC2241646; doi:10.1186/1471-2105-8-386)
Supplement: Additional file 1 — top25solutionsBoutrosData. 25 highest scoring network structures for the data by Boutros et al. [file 1471-2105-8-386-S1.gz › nem/..Rcheck/nem/html/SCCgraph.html]

R: Combines Strongly Connected Components into single nodes

|  |  |
| --- | --- |
| SCCgraph {nem} | R Documentation |

## Combines Strongly Connected Components into single nodes

### Description

`SCCgraph` is used to identify all nodes which are not distinguishable given the data.

### Usage

```
SCCgraph(x,name=TRUE,nlength=20)
```

### Arguments

|  |  |
| --- | --- |
| `x` | graphNEL object or an adjacency matrix |
| `name` | Concatenate all names of summarized nodes, if TRUE, or number nodes, if FALSE. Default: TRUE |
| `nlength` | maximum length of names |

### Details

A graph inferred by either `score` or `pairwise.posterior` may have cycles if some phenotypic profiles are not distinguishable.
The function `SCCgraph` identifies cycles in the graph (the strongly conneced components) and summarizes them in a single node.
The resulting graph is then acyclic.

### Value

|  |  |
| --- | --- |
| `graph` | a graphNEL object with connected components of the input graph summarized into single nodes |
| `scc` | a list mapping SCCs to nodes |
| `which.scc` | a vector mapping nodes to SCCs |

### Author(s)

Florian Markowetz <URL: http://genomics.princeton.edu/~florian>

### See Also

`nem`, `transitive.reduction`

### Examples

```
   data("BoutrosRNAi2002")
   D   <- BoutrosRNAiDiscrete[,9:16]
   res <- nem(D,para=c(.13,.05))
   # 
   sccg <- SCCgraph(res$graph,name=TRUE)
   #
   par(mfrow=c(1,2))
   plot(res, main="inferred from data")
   plot(sccg$graph, main="condensed (rel,key)")
```

---

[Package *nem* version 1.4.2 Index]
