# Supplementary material for: Large scale statistical inference of signaling pathways from RNAi and microarray data
Source: BMC Bioinformatics. 2007 Oct 15;8:386. doi: 10.1186/1471-2105-8-386 (PMC2241646; doi:10.1186/1471-2105-8-386)
Supplement: Additional file 1 — top25solutionsBoutrosData. 25 highest scoring network structures for the data by Boutros et al. [file 1471-2105-8-386-S1.gz › nem/..Rcheck/nem/html/00Index.html]

R: Nested Effects Models to reconstruct phenotypic hierarchies

# Nested Effects Models to reconstruct phenotypic hierarchies

---

## Documentation for package `nem' version 1.4.2

## User Guides and Package Vignettes

Read overview or browse directory.

## Help Pages

|  |  |
| --- | --- |
| BFSlevel | Build (generalized) hierarchy by Breath-First Search |
| BoutrosRNAi2002 | RNAi data on Drosophila innate immune response |
| BoutrosRNAiDiscrete | RNAi data on Drosophila innate immune response |
| BoutrosRNAiExpression | RNAi data on Drosophila innate immune response |
| connectModules | internal functions |
| CONTmLL | Marginal likelihood of a phenotypic hierarchy with continuous data |
| enumerate.models | Exhaustive enumeration of models |
| FULLmLL | Full marginal likelihood of a phenotypic hierarchy |
| getRelevantEGenes | Automatic selection of most relevant S-genes |
| internal | internal functions |
| local.model.prior | Computes a prior to be used for edge-wise model inference |
| mLL | Marginal likelihood of a phenotypic hierarchy |
| moduleNetwork | Infers a phenotypic hierarchy using the module network |
| moduleNetwork.aux | internal functions |
| nem | Nested Effects Models - main function |
| nem.cont.preprocess | Calculate classification probabilities of perturbation data according to control experiments |
| nem.discretize | Discretize perturbation data according to control experiments |
| nemModelSelection | model selection for nested effect models |
| network.AIC | AIC criterion for network graph |
| pairwise.posterior | Infers a phenotypic hierarchy edge by edge |
| PhiDistr | Computes the marginal likelihood of phenotypic hierarchies |
| plot.effects | Plots data according to a phenotypic hierarchy |
| plot.ModuleNetwork | plot nested effect model |
| plot.nem | plot nested effect model |
| plot.pairwise | plot nested effect model |
| plot.score | plot nested effect model |
| plot.triples | plot nested effect model |
| print.ModuleNetwork | Infers a phenotypic hierarchy using the module network |
| print.pairwise | Infers a phenotypic hierarchy edge by edge |
| print.score | Computes the marginal likelihood of phenotypic hierarchies |
| print.triples | Infers a phenotypic hierarchy from triples |
| prune.graph | Prunes spurious edges in a phenotypic hierarchy |
| SCCgraph | Combines Strongly Connected Components into single nodes |
| score | Computes the marginal likelihood of phenotypic hierarchies |
| selectEGenes | Automatic selection of most relevant S-genes |
| subsets | Subsets |
| transitive.closure | Computes the transitive closure of a directed graph |
| transitive.reduction | Computes the transitive reduction of a graph |
| triples.posterior | Infers a phenotypic hierarchy from triples |
