# Supplementary material for: Large scale statistical inference of signaling pathways from RNAi and microarray data
Source: BMC Bioinformatics. 2007 Oct 15;8:386. doi: 10.1186/1471-2105-8-386 (PMC2241646; doi:10.1186/1471-2105-8-386)
Supplement: Additional file 1 — top25solutionsBoutrosData. 25 highest scoring network structures for the data by Boutros et al. [file 1471-2105-8-386-S1.gz › nem/..Rcheck/nem/html/nem.cont.preprocess.html]

R: Calculate classification probabilities of perturbation data according to control experiments

|  |  |
| --- | --- |
| nem.cont.preprocess {nem} | R Documentation |

## Calculate classification probabilities of perturbation data according to control experiments

### Description

Calculates probabilities of data to define effects of interventions with respect to wildtype/control measurements

### Usage

```
nem.cont.preprocess(D,neg.control=NULL,pos.control=NULL,nfold=2, influencefactor=NULL, empPval=.05, verbose=TRUE)
```

### Arguments

|  |  |
| --- | --- |
| `D` | matrix with experiments as columns and effect reporters as rows |
| `neg.control` | either indices of columns in `D` or a matrix with the same number of rows as `D` |
| `pos.control` | either indices of columns in `D` or a matrix with the same number of rows as `D` |
| `nfold` | fold-change between neg. and pos. controls for selecting effect reporters. Default: 2 |
| `influencefactor` | factor multiplied onto the probabilities, so that all negative control genes are treated as influenced, usually automatically determined |
| `empPval` | empirical p-value cutoff for effects if only one control is available |
| `verbose` | Default: TRUE |

### Details

Determines the empirical distributions of the controls and calculates the probabilities of pertubartion data to belong to the control distribution(s).

### Value

|  |  |
| --- | --- |
| `dat` | data matrix |
| `pos` | positive controls [in the two-controls setting] |
| `neg` | negative controls [in the two-controls setting] |
| `sel` | effect reporters selected [in the two-controls setting] |
| `prob.influenced` | probability of a reporter to be influenced |
| `influencefactor` | factor multiplied onto the probabilities, so that all negative control genes are treated as influenced |

### Note

preliminary! will be developed to be more generally applicable

### Author(s)

Florian Markowetz <URL: http://genomics.princeton.edu/~florian>

### References

Markowetz F, Bloch J, Spang R, Non-transcriptional pathway features reconstructed from secondary effects of RNA interference, Bioinformatics, 2005

### See Also

`BoutrosRNAi2002`

### Examples

```
   data("BoutrosRNAi2002")
   preprocessed <- nem.cont.preprocess(BoutrosRNAiExpression,neg.control=1:4,pos.control=5:8)
```

---

[Package *nem* version 1.4.2 Index]
