# Supplementary material for: Large scale statistical inference of signaling pathways from RNAi and microarray data
Source: BMC Bioinformatics. 2007 Oct 15;8:386. doi: 10.1186/1471-2105-8-386 (PMC2241646; doi:10.1186/1471-2105-8-386)
Supplement: Additional file 1 — top25solutionsBoutrosData. 25 highest scoring network structures for the data by Boutros et al. [file 1471-2105-8-386-S1.gz › nem/..Rcheck/nem/doc/nem.tmp]

— Nested Effects Models —
An example in *Drosophila* immune response

Florian Markowetz[*]

March 7, 2007

Abstract

Cellular signaling pathways, which are not modulated on a transcriptional level, cannot be directly deduced from expression profiling experiments. The situation changes, when external interventions like RNA interference or gene knock-outs come into play.

In Markowetz *et al.* (2005) and Markowetz (2006) we introduced an algorithm to infer non-transcriptional pathway features based on differential gene expression in silencing assays. The method is implemented in the Bioconductor package `nem`. Here we demonstrate its practical use in the context of an RNAi data set investigating the response to microbial challenge in Drosophila melanogaster.

We show in detail how the data is pre-processed and discretized, how the pathway can be reconstructed by different approaches, and how the final result can be post-processed to increase interpretability.

1 Drosophila RNAi data

We applied our method to data from a study on innate immune response in *Drosophila* (Boutros *et al.*, 2002). Selectively removing signaling components blocked induction of all, or only parts, of the transcriptional response to LPS.

Dataset summary The dataset consists of 16 Affymetrix-microarrays: 4 replicates of control experiments without LPS and without RNAi (negative controls), 4 replicates of expression profiling after stimulation with LPS but without RNAi (positive controls), and 2 replicates each of expression profiling after applying LPS and silencing one of the four candidate genes *tak*, *key*, *rel*, and *mkk4/hep*.

[*]Lewis-Sigler Institute for Integrative Genomics, Pinceton, NJ 08544, USA. eMail: florian@genomics.princeton.edu; URL: http://genomics.princeton.edu/~florian

Preprocessing and E-gene selection For preprocessing, we perform normalization on probe level using a variance stabilizing transformation (Huber *et al.*, 2002), and probe set summarization using a median polish fit of an additive model (Irizarry *et al.*, 2003). The result is included as a dataset in the package `nem`.

```
> library(nem)
> data("BoutrosRNAi2002")
```

The function `nem.discretize` implements the following two preprocessing steps: First, we select the genes as effect reporters (E-genes), which are more then two-fold upregulated by LPS treatment. Next, we transform the continuous expression data to binary values. We set an E-genes state in an RNAi experiment to `1` if its expression value is sufficiently far from the mean of the positive controls, *i.e.* if the intervention interrupted the information flow. If the E-genes expression is close to the mean of positive controls, we set its state to `0`.

Let C_{ik} be the continuous expression level of E_i in experiment k. Let μ_i^+ be the mean of positive controls for E_i, and μ_i^- the mean of negative controls. To derive binary data E_{ik}, we defined individual cutoffs for every gene E_i by:

$$E_{ik} = \begin{cases} 1 & \text{if } C_{ik} < \kappa \cdot \mu_i^+ + (1 - \kappa) \cdot \mu_i^-, \\ 0 & \text{else.} \end{cases} \qquad (1)$$

```
> res.disc <- nem.discretize(BoutrosRNAiExpression, neg.control = 1:4,
+     pos.control = 5:8, cutoff = 0.7)

discretizing with respect to POS and NEG controls
```

Estimating error probabilities From the positive and negative controls, we can estimate the error probabilities α and β. The type I error α is the number of positive controls discretized to state `1`, and the type II error β is the number of negative controls in state `0`. To guard against unrealistically low estimates we add pseudo counts. The error estimates are included into the discretization results:

```
> res.disc$para

   a    b
0.19 0.07
```

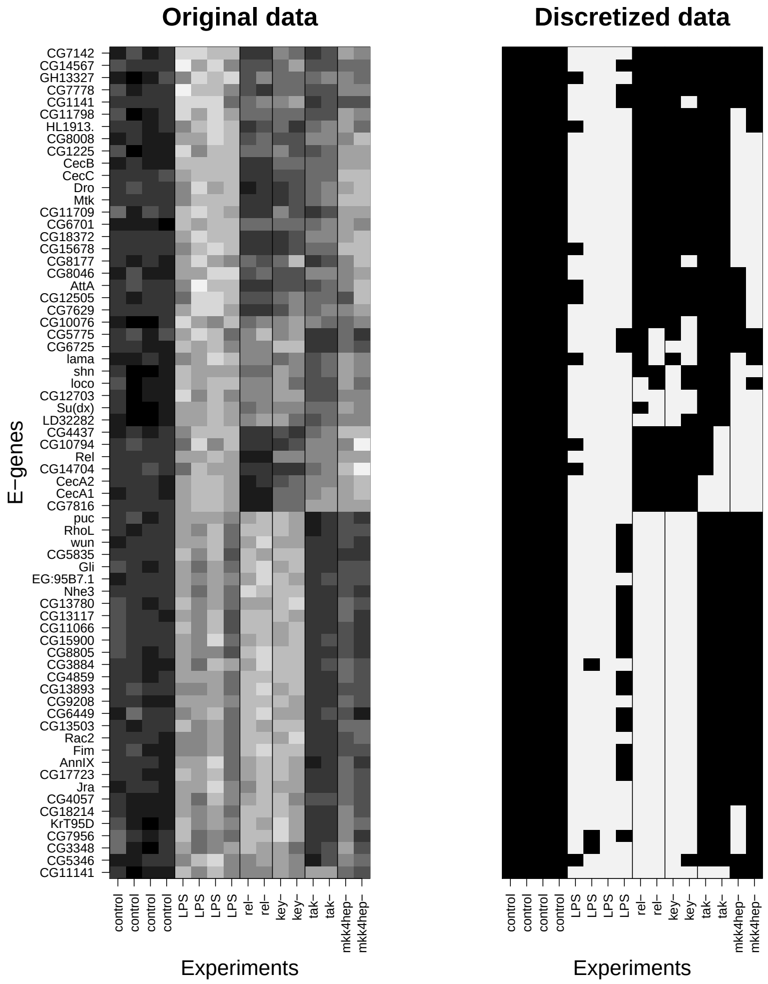

Figure 1: Continuous and discretized data

2 Applying Nested Effects Models

Which model explains the data best? With only four S-genes, we can exhaustively enumerate all pathway models and search the whole space for the best-fitting model. To score these models use either the marginal likelihood depending on α and β (details found in Markowetz et al (2005)) or the full marginal likelihood depending on hyperparameters (details in Markowetz, 2006). Additionally we show how to employ an edge-wise inference heuristic, which can also be applied to cases where exhaustive search over model space is infeasible (i.e. when we have more than 4 or 5 perturbed genes), an inference scheme from triples and so-called *module networks*. An interface to all inference techniques is provided by the function `nem()`.

2.1 Exhaustive search by marginal likelihood

Scoring models by marginal log-likelihood is implemented in function `score`. Input consists of models and data, the type of the score (`"mLL"` or `"FULLmLL"`), the corresponding paramters (`para`) or hyperparameters (`hyperpara`) and a prior for E-gene positions (`P`).

```
> result <- nem(res.disc$dat, type = "mLL", para = res.disc$para,
+     inference = "search")
```

```
Generated 355 unique models ( out of 4096 )
Computing marginal likelihood for 355 models
```

```
> result
```

```
Object of class 'score' generated by 'score()'

$graph:  phenotypic hierarchy on genes
$mLL:    a vector of length 355
$pos:    a list   of length 355
$mappos: a vector of length 355
$lambda: 0
```

The output is the highest scoring model (`result$graph`), a vector of scores (`result$mLL`) and a list of E-gene position posteriors (`result$pos`), and a MAP estimate of E-gene positions (`result$mappos`). We can plot the results using the commands:

```
> plot(result, what = "graph")
> plot(result, what = "mLL")
> plot(result, what = "pos")
```

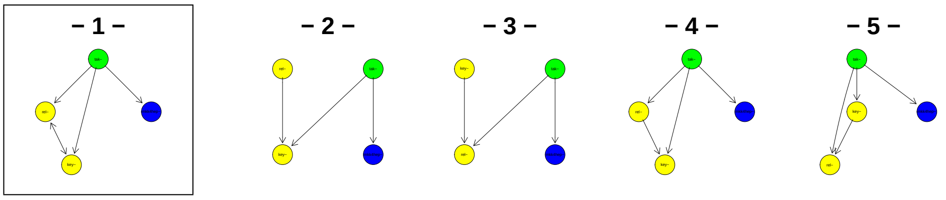

Figure 2: The five silencing schemes getting high scores in Fig. 3. It takes a second to see it, but Nr.2 to 5 are not that different from Nr.1. The main feature, ie. the branching downstream of *tak* is conserved in all of them.

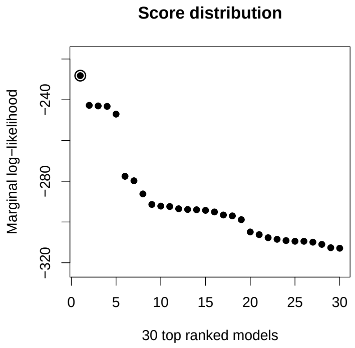

Figure 3: The best 30 scores

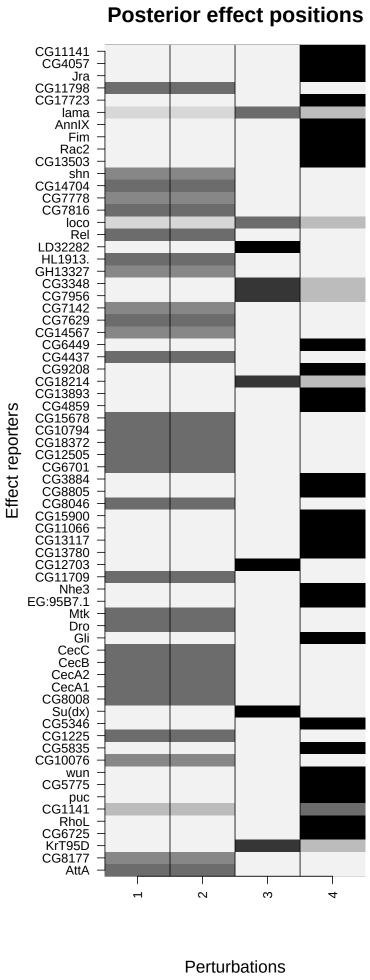

Figure 4: Posterior distributions of E-gene positions given the highest scoring silencing scheme (Nr. 1 in Fig. 2). The MAP estimate corresponds to the row-wise maximum.

2.2 Exhaustive search Full marginal likelihood

Additionally to what we did in the paper (Markowetz *et al.*, 2005) the PhD thesis (Markowetz, 2006) contains equations for a "full marginal likelihood" in which error probabilities α and β are integrated out. This section shows that using this score we learn the same pathways as before.

```
> result2 <- nem(res.disc$dat, type = "FULLmLL", hyperpara = c(1,
+     9, 9, 1), inference = "search")
```

```
Generated 355 unique models ( out of 4096 )
Computing FULL marginal likelihood for 355 models
```

```
> result2
```

```
Object of class 'score' generated by 'score()'

$graph:  phenotypic hierarchy on genes
$mLL:    a vector of length 355
$pos:    a list   of length 355
$mappos: a vector of length 355
$lambda: 0
```

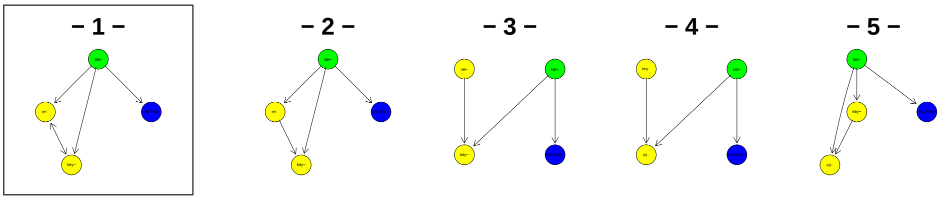

Figure 5: Same topologies as before.

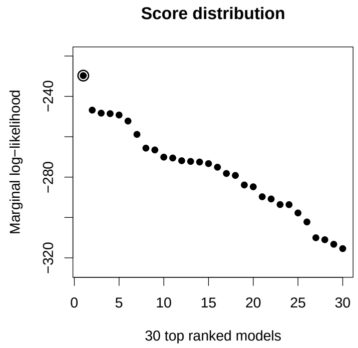

Figure 6: The best 30 scores by full marginal likelihood

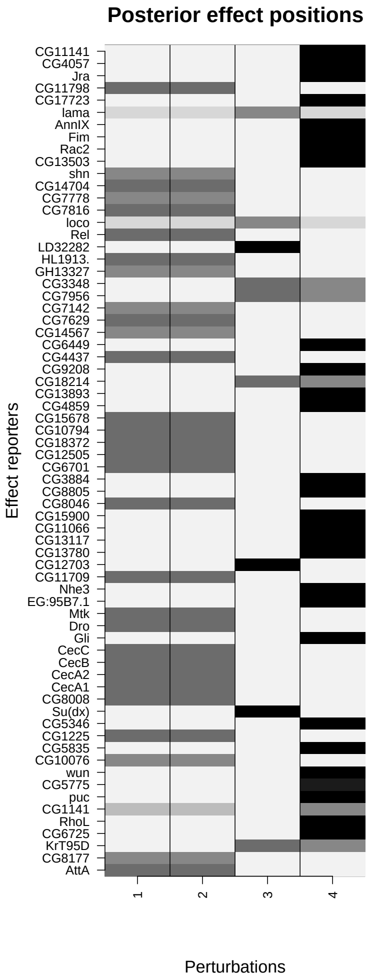

Figure 7: Posterior distributions of E-gene positions given the highest scoring silencing scheme (Nr. 1 in Fig. 5). The MAP estimate corresponds to the row-wise maximum.

2.3 Edge-wise learning

Instead of scoring whole pathways, we can learn the model edge by edge. For each pair of genes A and B we infer the best of four possible models: $A \cdot \cdot B$ (unconnected), $A \rightarrow B$ (effects of A are superset of effects of B), $A \leftarrow B$ (subset), and $A \leftrightarrow B$ (undistinguishable effects).

```
> result3 <- nem(res.disc$dat, para = res.disc$para, inference = "pairwise")
```

```
4 perturbed genes -> 6 pairwise tests (lambda =  0 )
......
estimating effect positions
```

```
> result3
```

```
Object of class 'pairwise' generated by 'pairwise.posterior()'

$graph:  phenotypic hierarchy on genes)
$scores: posterior distributions of local models

Summary of MAP estimates:
all  ..  -> <->
  6   2   3   1
```

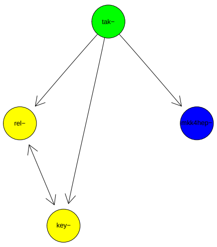

Figure 8: Result of edge-wise learning. Compare this to the result from global search. It looks exactely the same.

2.4 Inference from triples

Edge-wise learning assumes independence of edges. But this is not true in transitively closed graphs, where a direct edge must exist whenever there is a longer path between two nodes. Natural extension of edge-wise learning is inference from triples of nodes. In the package `nem` we do it by

```
> result4 <- nem(res.disc$dat, para = res.disc$para, inference = "triples")
```

```
4 perturbed genes -> 4 triples to check (lambda =  0 )
.....
Estimating effect positions in combined graph
```

```
> result4
```

```
Object of class 'triples' generated by 'triples.posterior()'

$graph:  phenotypic hierarchy on genes
$avg:    matrix of edge frequencies in triple models
```

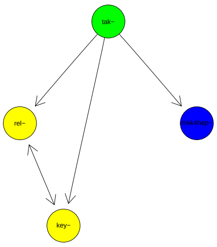

Figure 9: Result of triple learning. Compare this to the result from global search and pairwise learning

2.5 Inference with module networks

Rather than looking for a complete network hypothesis at once the idea of the module network is to build up a graph from smaller subgraphs, called *modules* in the following. The module network is thus a divide and conquer approach: We first split the complete node set into smaller subgroups. This can be done by PAM clustering [Kaufman and Roussew (1990)] on the preprocessed expression profiles of the S-genes. The idea is that S-genes with a similar E-gene response profile (here: with regard to the Manhattan distance) should be close in the signaling path. The number of clusters for the k-means clustering is chosen between 2 and half of the number of S-genes such that the average silhouette index becomes maximal. The silhouette value for each point in a cluster is a measure of how similar that point is to points in its own cluster vs. points in other clusters, and ranges from -1 to +1 [Rousseeuw (1987)]. It is defined as:

$$S(i) = \frac{\min_j(\bar{d}_B(i,j)) - \bar{d}_W(i)}{\max(\bar{d}_W(i), \min_j(\bar{d}_B(i,j))} \qquad (2)$$

where $\bar{d}_W(i)$ is the average distance from the i-th point to the other points in its own cluster, and $\bar{d}_B(i,j)$ is the average distance from the i-th point to points in another cluster j.

Each cluster of S-genes now forms one module. These modules are eventually further subdivided into smaller submodules until each submodule contains only 4 S-genes at most. This way we obtain a tree structure of modules, where each node (module) has children (submodules). We begin with estimating the leaves in the module tree. As each leaf module can contain 4 S-genes at maximum this can be done by enumerating all possible transitively closed network hypotheses and taking the highest scoring one. After the leaves in the module tree have been built, their connection is estimated. For this purpose we test all pairwise connections between any pair of S-genes from leaves L_1 and L_2. Denoting by $|L_1|$ and $|L_2|$ the number of S-genes in both leaves, these are $4 \cdot |L_1| \cdot |L_2|$ tests altogether, because between any pair of S-genes (n_1, n_2) we can either have no edge, an edge from n_1 to n_2, an edge from n_2 to n_1 or an edge in both directions. After the connection between L_1 and L_2 has been estimated, the corresponding subgraph is transitively closed. After all connections between leaves belonging to the same submodule in the module tree have been established, we recursively continue with connecting submodules in the same fashion as we did for leaf modules until the topology for the total network is completed.

In the package `nem` we call the module network by

```
> result5 <- nem(res.disc$dat, para = res.disc$para, inference = "ModuleNetwork")
```

```
Estimating module network of 4 S-genes (lambda = 0 )...

estimating network of genes:
rel- key- tak- mkk4hep-
--> estimating local model
Generated 355 unique models ( out of 4096 )
Computing marginal likelihood for 355 models
================ finished!!! ================
```

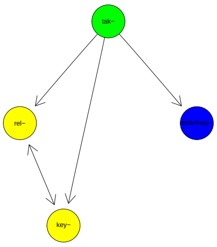

Figure 10: Result of module network learning. It is exactly the same as for the exhaustive search.

2.6 Incorporating prior Assumptions via Regularization

The `nem` package allows to specify a prior on the network structure itself. This can be thought of biasing the score of possible network hypotheses towards prior knowledge. It is crucial to understand that in principle in any inference scheme there exist two competing goals: Belief in prior assumptions / prior knowledge versus belief in data. Only trusting the data itself may lead to overfitting, whereas only trusting in prior assumptions does not give any new information and prevents learning. Therefore, we need a trade-off between both goals via a regularization constant $\lambda > 0$, which specifies the belief in our prior assumptions. In the simplest case our assumption could be that the true network structure is sparse, i.e. there are only very few edges.

```
> result6 <- nem(res.disc$dat, para = res.disc$para, Pm = matrix(0,
+     ncol = 4, nrow = 4), lambda = 10)
```

```
4 perturbed genes -> 6 pairwise tests (lambda =  10 )
......
estimating effect positions
```

```
> result6
```

```
Object of class 'pairwise' generated by 'pairwise.posterior()'

$graph:  phenotypic hierarchy on genes)
$scores: posterior distributions of local models

Summary of MAP estimates:
all  ..  -> <->
  6   2   4  NA
```

In practice we would like to choose a λ in an automated fashion. This leads to an instance of the classical *model selection* problem (e.g. [Hastie *et al.* (2001)]) in statistical learning. One way of dealing with it is to tune λ such that the *Akaike information criterion* (AIC)

$$AIC(\lambda, \Phi_{opt}) = -2 \log P(D|\Phi_{opt}) + 2d(\lambda, \Phi_{opt}) \tag{3}$$

becomes minimal [Hastie *et al.* (2001)]. Here $d(\lambda, \Phi_{opt})$ denotes the number of parameters (i.e. the number of edges) in the highest scoring network structure Φ_{opt}.

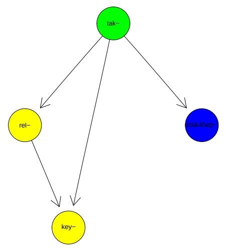

Figure 11: Result of module network learning with regularization towards sparse graph structures ($\lambda = 10$).

```
> result7 <- nemModelSelection(c(0.1, 1, 10), res.disc$dat, para = res.disc$para,
+     Pm = matrix(0, ncol = 4, nrow = 4))
```

```
4 perturbed genes -> 6 pairwise tests (lambda =  0.1 )
......
estimating effect positions
4 perturbed genes -> 6 pairwise tests (lambda =  1 )
......
estimating effect positions
4 perturbed genes -> 6 pairwise tests (lambda =  10 )
......
estimating effect positions
==> AIC ( lambda =  0.1 ) =  466.316716650454 ( #param = 5 )===============
==> AIC ( lambda =  1 ) =  466.316716650454 ( #param = 5 )===============
==> AIC ( lambda =  10 ) =  494.481213897227 ( #param = 4 )===============
====> chosen best model with lambda = 0.1
```

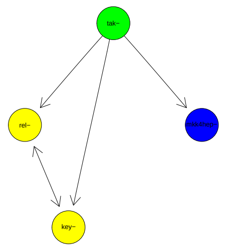

Figure 12: Result of module network learning with regularization towards sparse graph structures and automatic model selection.

2.7 Omitting the Data Discretization Step

In general performing a data discretization on the expression profiles as described in Sec. 1 can be critical. An alternative is given by taking the raw p-value profiles obtained from testing for differential gene expression. In this situation we assume the individual p-values in the data matrix to be drawn from a mixture of a uniform and an alternative distribution. The alternative distribution can e.g. be a Beta(1, β) distribution, maybe with an additional small uniform mixture component. The `nem` package supports such a data format using the option `type = "CONTmLLDens"` in the call of the function `nem`.

A second possibility to omit the data discretization step is to calculate the effect probability for each gene based on given the empirical distributions of the controls.

```
> preprocessed <- nem.cont.preprocess(BoutrosRNAiExpression, neg.control = 1:4,
+     pos.control = 5:8)
```

```
preprocessing with respect to POS and NEG controls
```

```
> result8 <- nem(preprocessed$prob.influenced, type = "CONTmLL",
+     inference = "search")
```

```
Generated 355 unique models ( out of 4096 )
Computing marginal likelihood for 355 models
```

```
> result8
```

```
Object of class 'score' generated by 'score()'

$graph:  phenotypic hierarchy on genes
$mLL:    a vector of length 355
$pos:    a list   of length 355
$mappos: a vector of length 355
$lambda: 0
```

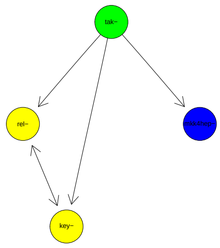

Figure 13: Learned structure from effect probabilities

3 Visualization

```
> plot.effects(res.disc$dat, result)
```

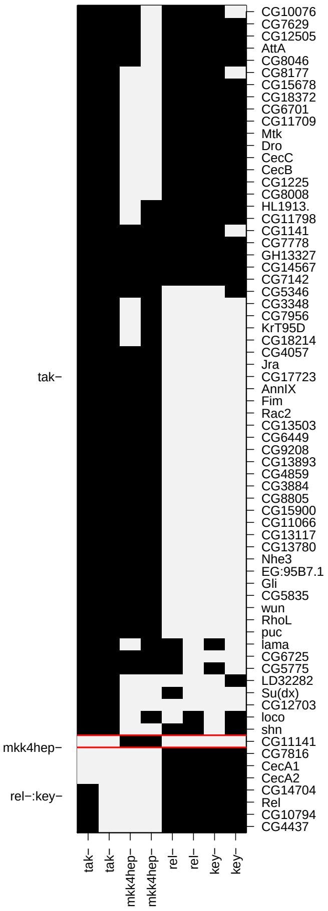

Figure 14: plotting data according to inferred hierarchy

4 Post-processing of results

Combining strongly connected components First, we identify all nodes/genes which are not distinguishable given the data. This amounts to finding the strongly connected components in the graph. Relish and Key are now combined into one node.

```
> result3.scc <- SCCgraph(result3$graph, name = TRUE)
> plot(result3.scc$graph)
```

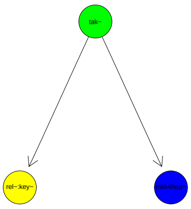

Figure 15: The undistinguishable profiles of *key* and *rel* are summarized into a single node.

Transitive reduction Additionally, in bigger graphs `transitive.reduction()` helps to see the structure of the network better. In this simple example there are no shortcuts to remove.

References

[Boutros *et al.* (2002)] Boutros M, Agaisse H, Perrimon N. Sequential activation of signaling pathways during innate immune responses in *Drosophila. Developmental Cell*, 3(5):711–722, 2002.

[Huber *et al.* (2002)] Huber W, Heydebreck A, Sültmann H, Poustka A, Vingron M. Variance Stabilization Applied to Microarray Data Calibration and to the Quantification of Differential Expression. *Bioinformatics*, 18:S96–S104, 2002.

[Irizarry *et al.* (2003)] Irizarry RA, Bolstad BM, Collin F, Cope LM, Hobbs B, Speed TP. Summaries of Affymetrix GeneChip probe level data. *Nucleic Acids Res.* 31(4):e15, 2003.

[Markowetz *et al.* (2005)] Markowetz F, Bloch J, Spang R. Non-transcriptional pathway features reconstructed from secondary effects of RNA interference *Bioinformatics*, 2005.

[Markowetz (2006)] Markowetz F. Probabilistic Models for Gene Silencing Data. *PhD thesis*, Free University Berlin, 2005.

[Kaufman and Roussew (1990)] L. Kaufman and P. Rousseeuw. Finding Groups in Data: An Introduction to Cluster Analysis. Wiley, New York, 1990.

[Rousseeuw (1987)] Rousseeuw, P.J. Silhouettes: a graphical aid to the interpretation and validation of cluster analysis. *J. Comp. and Applied Mathematics*, 20:53–65, 1987.

[Hastie *et al.* (2001)] T. Hastie and R. Tibshirani and J. Friedman. The Elements of Statistical Learning. Springer, 2001

Session Information

The version number of R and packages loaded for generating the vignette were:

- Version 2.3.1 (2006-06-01), `x86_64-pc-linux-gnu`
- Base packages: base, datasets, grDevices, graphics, methods, stats, tools, utils
- Other packages: RBGL 1.8.1, Rgraphviz 1.10.0, Ruuid 1.10.0, class 7.2-27.1, e1071 1.5-13, graph 1.10.6, nem 1.4.1
