# Supplementary figures and images for: Large scale statistical inference of signaling pathways from RNAi and microarray data
Source: BMC Bioinformatics. 2007 Oct 15;8:386. doi: 10.1186/1471-2105-8-386 (PMC2241646; doi:10.1186/1471-2105-8-386)

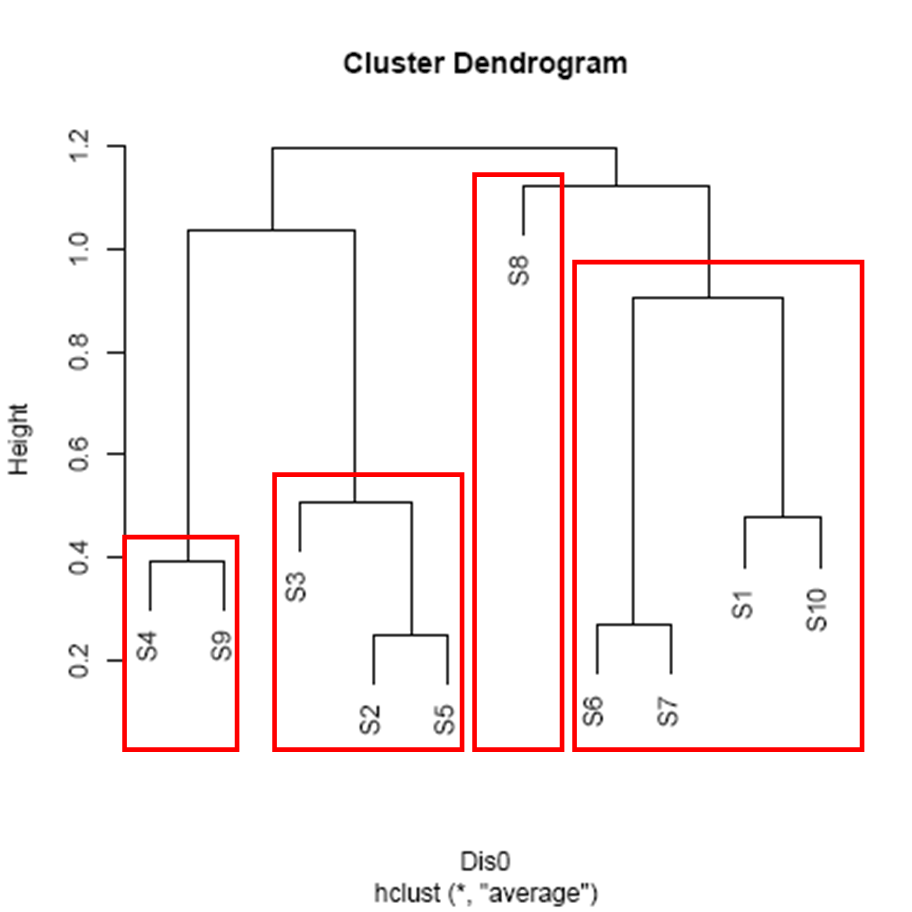

Supplement: Additional file 1 — top25solutionsBoutrosData. 25 highest scoring network structures for the data by Boutros et al. [file 1471-2105-8-386-S1.gz › nem/inst/doc/ModuleNetworks1.png]

# Original data

E-genes

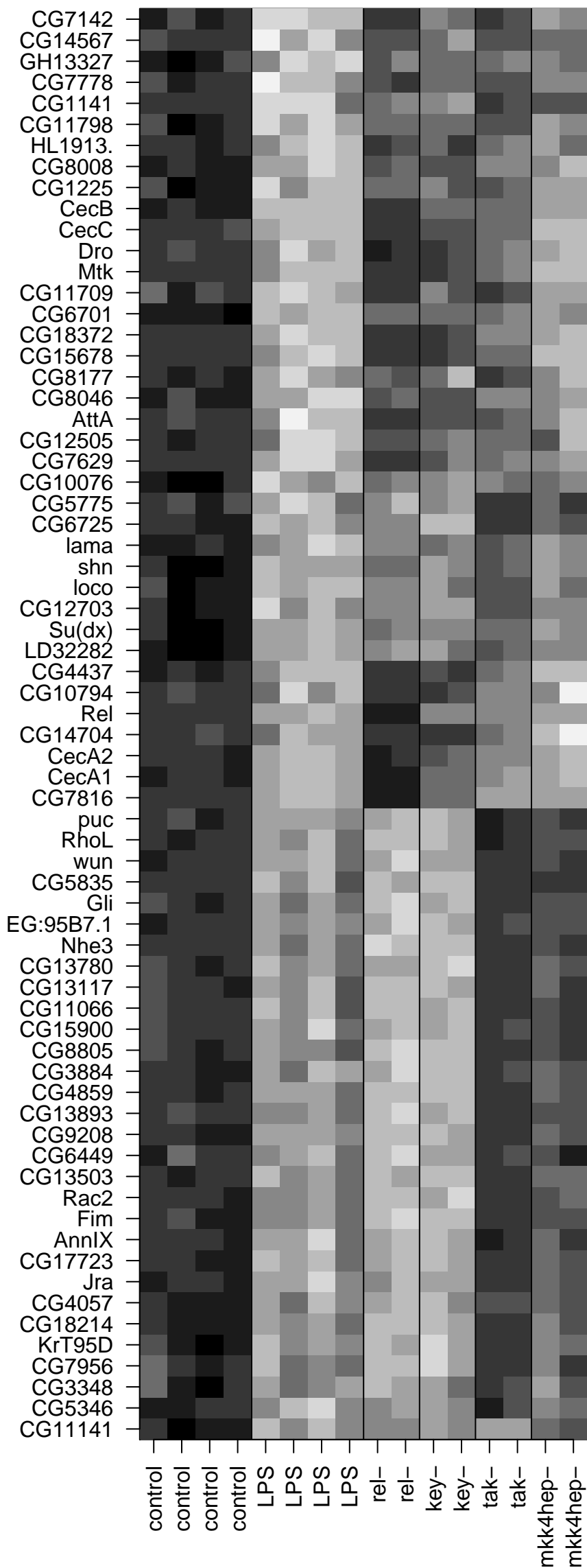

Experiments

Supplement: Additional file 1 — top25solutionsBoutrosData. 25 highest scoring network structures for the data by Boutros et al. [file 1471-2105-8-386-S1.gz › nem/..Rcheck/nem/doc/nem-data_cont.pdf]

# Discretized data

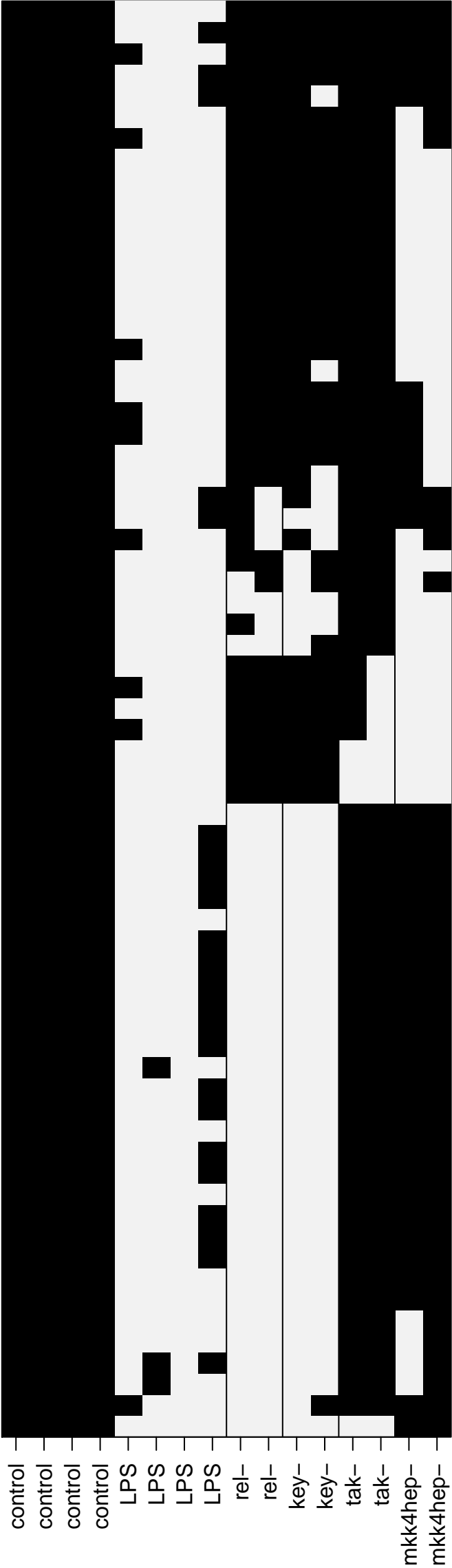

Supplement: Additional file 1 — top25solutionsBoutrosData. 25 highest scoring network structures for the data by Boutros et al. [file 1471-2105-8-386-S1.gz › nem/..Rcheck/nem/doc/nem-data_disc.pdf]

**top 25 models**

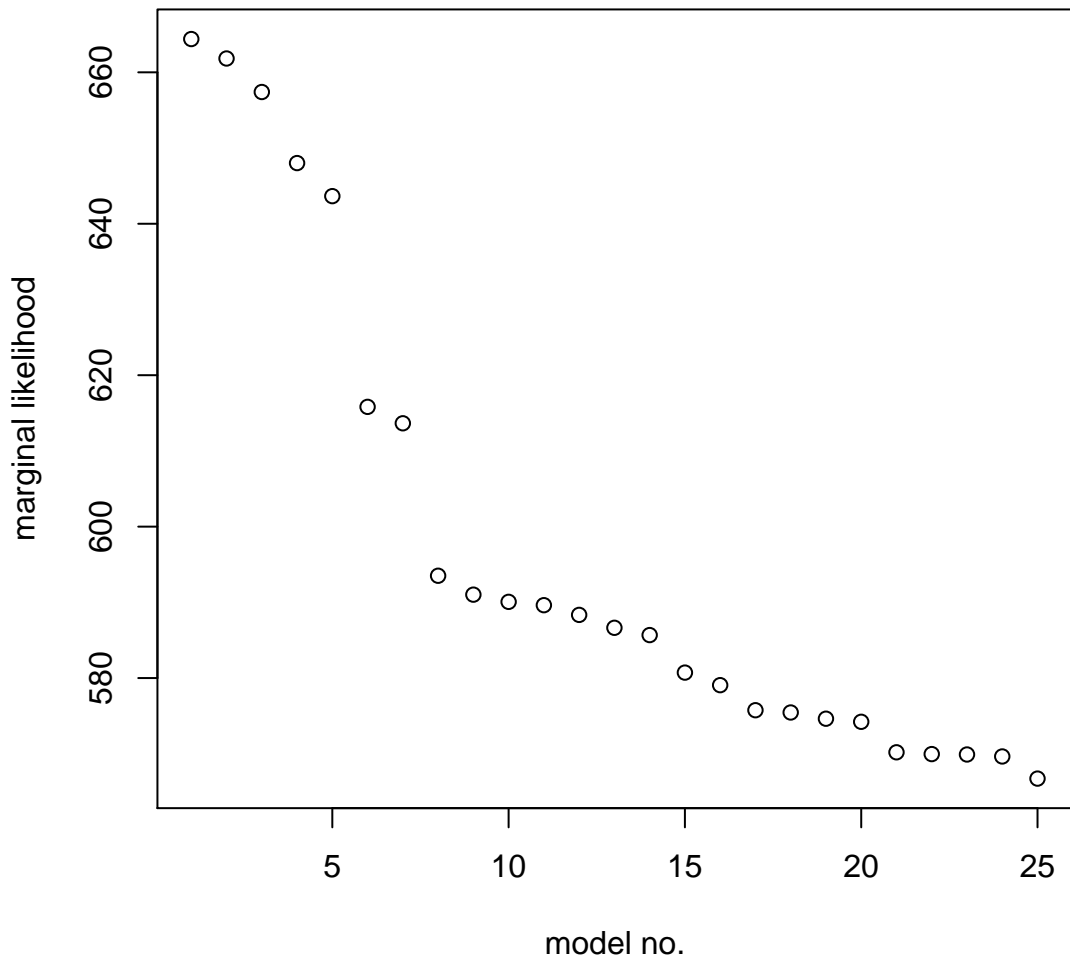

Supplement: Additional file 3 — nem_2.0.0. R package for nested effect models [file 1471-2105-8-386-S3.zip › mLLtop25.pdf]

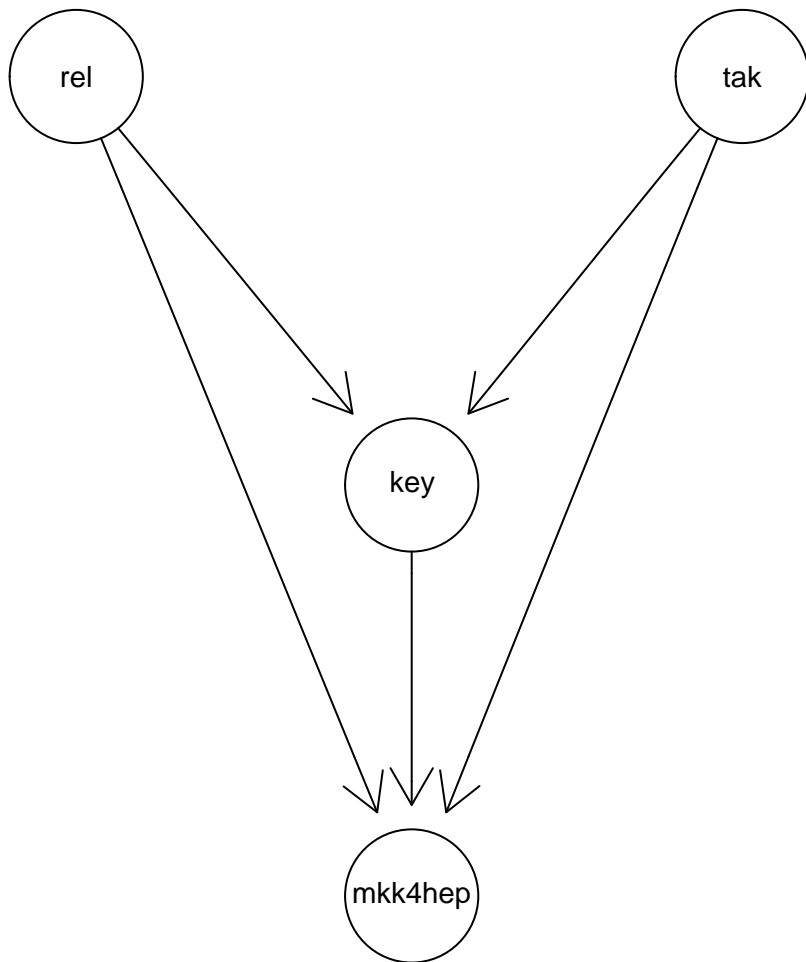

Supplement: Additional file 3 — nem_2.0.0. R package for nested effect models [file 1471-2105-8-386-S3.zip › solution10_orig.pdf]

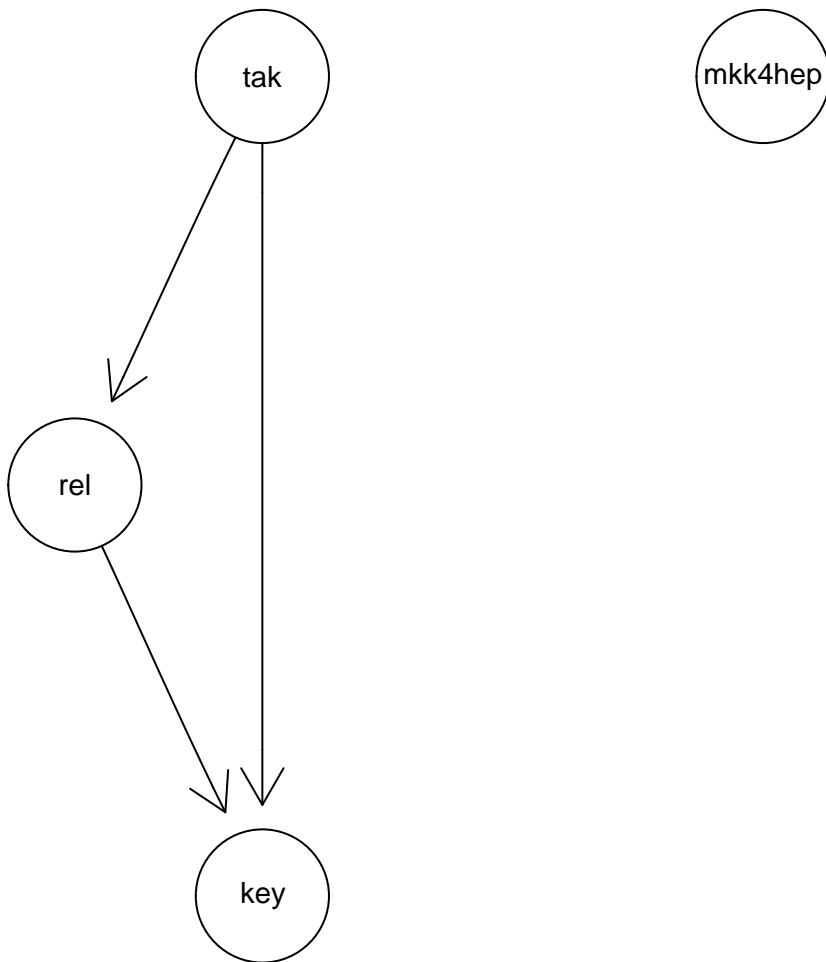

Supplement: Additional file 3 — nem_2.0.0. R package for nested effect models [file 1471-2105-8-386-S3.zip › solution11_orig.pdf]

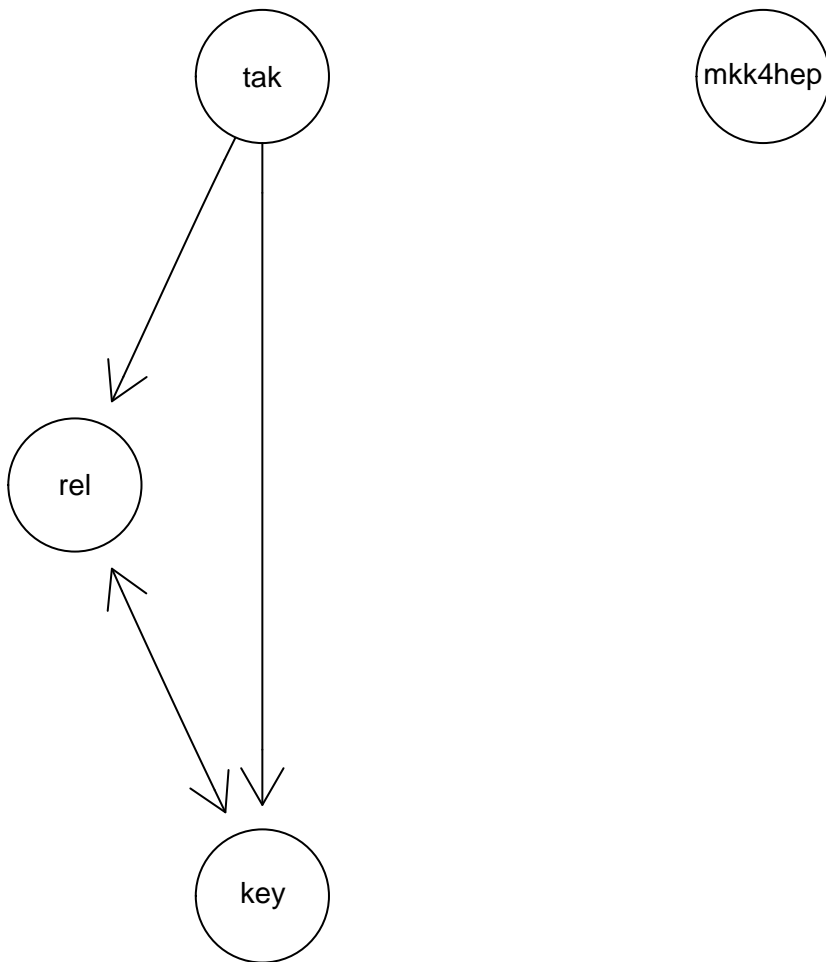

Supplement: Additional file 3 — nem_2.0.0. R package for nested effect models [file 1471-2105-8-386-S3.zip › solution12_orig.pdf]

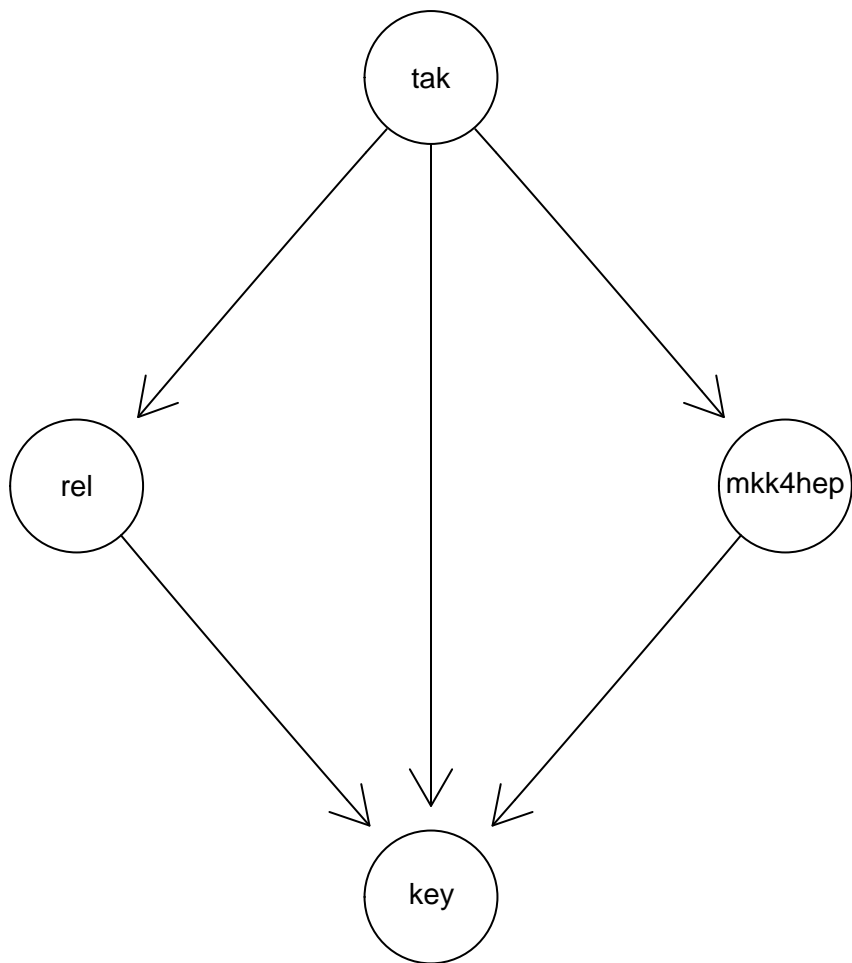

Supplement: Additional file 3 — nem_2.0.0. R package for nested effect models [file 1471-2105-8-386-S3.zip › solution13_orig.pdf]

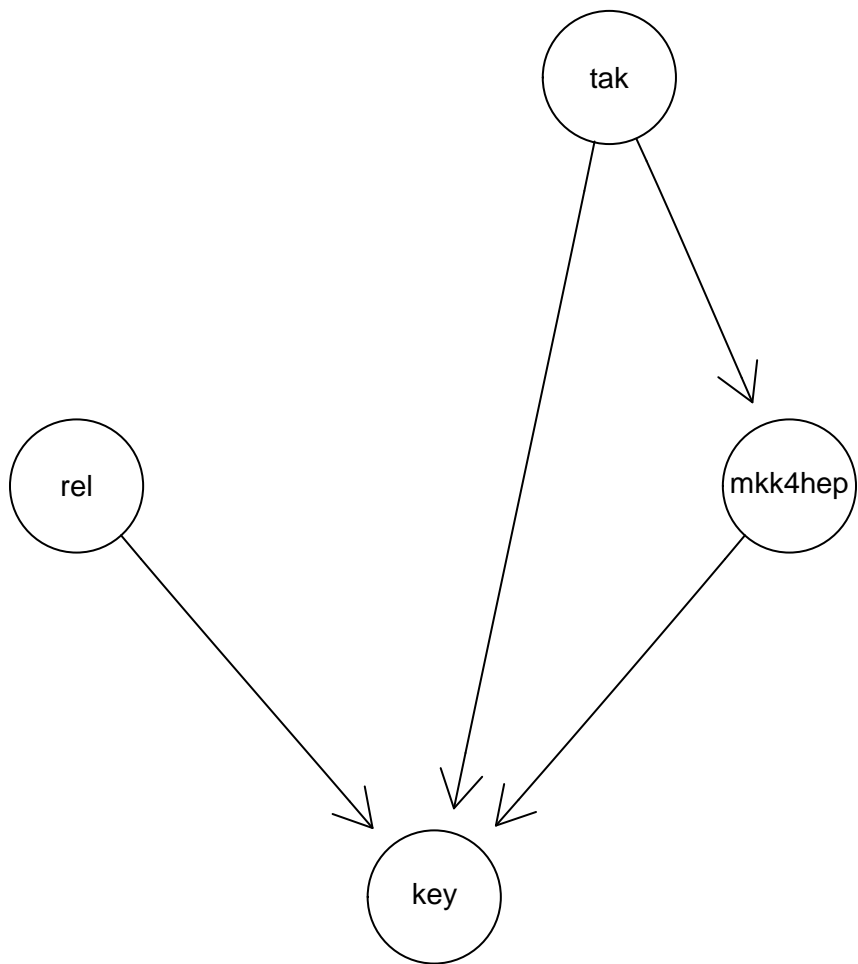

Supplement: Additional file 3 — nem_2.0.0. R package for nested effect models [file 1471-2105-8-386-S3.zip › solution14_orig.pdf]

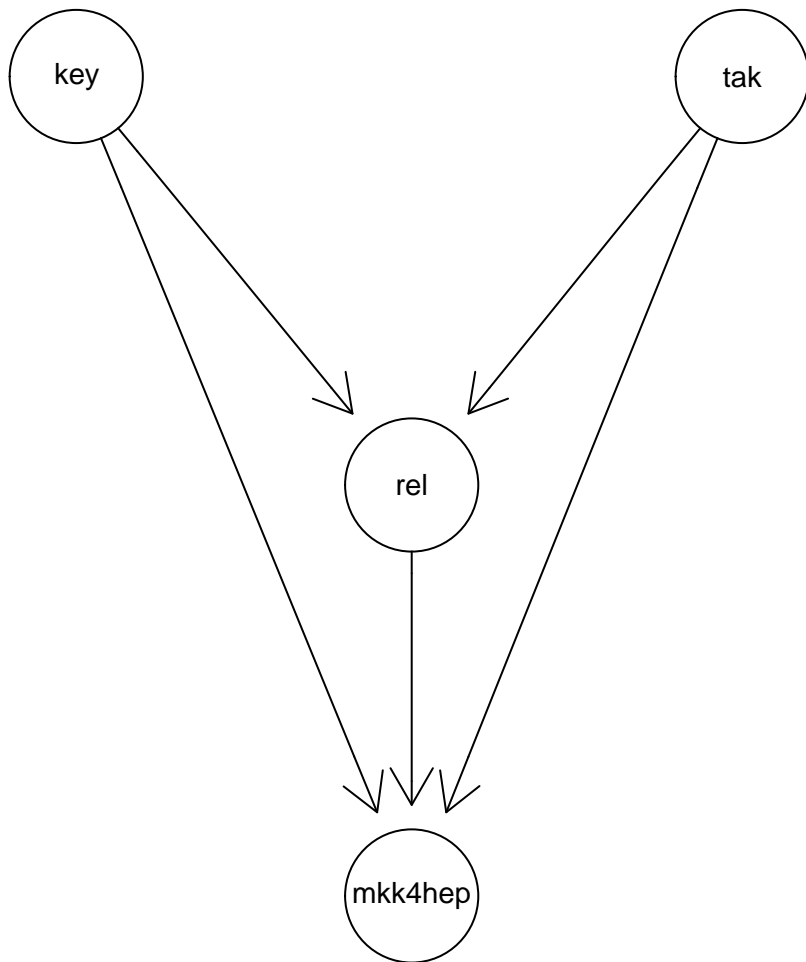

Supplement: Additional file 3 — nem_2.0.0. R package for nested effect models [file 1471-2105-8-386-S3.zip › solution15_orig.pdf]

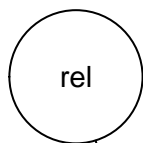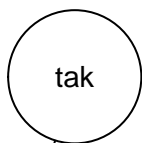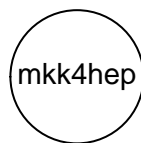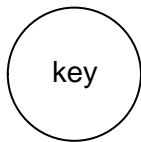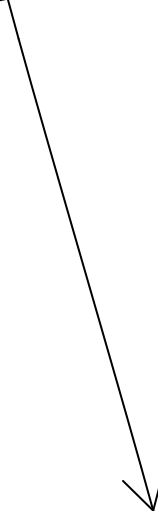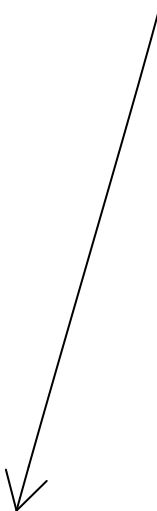

Supplement: Additional file 3 — nem_2.0.0. R package for nested effect models [file 1471-2105-8-386-S3.zip › solution16_orig.pdf]

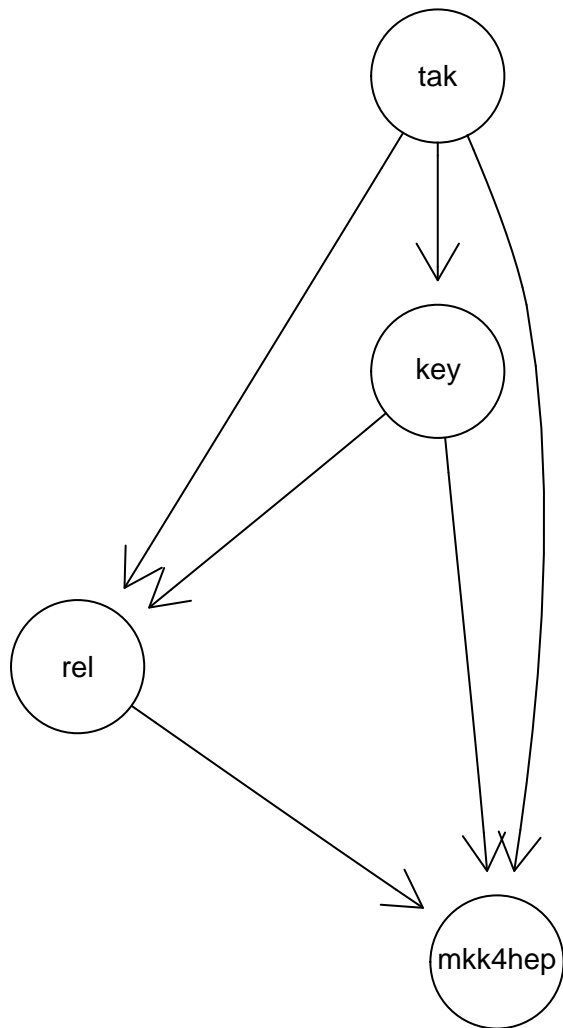

Supplement: Additional file 3 — nem_2.0.0. R package for nested effect models [file 1471-2105-8-386-S3.zip › solution17_orig.pdf]

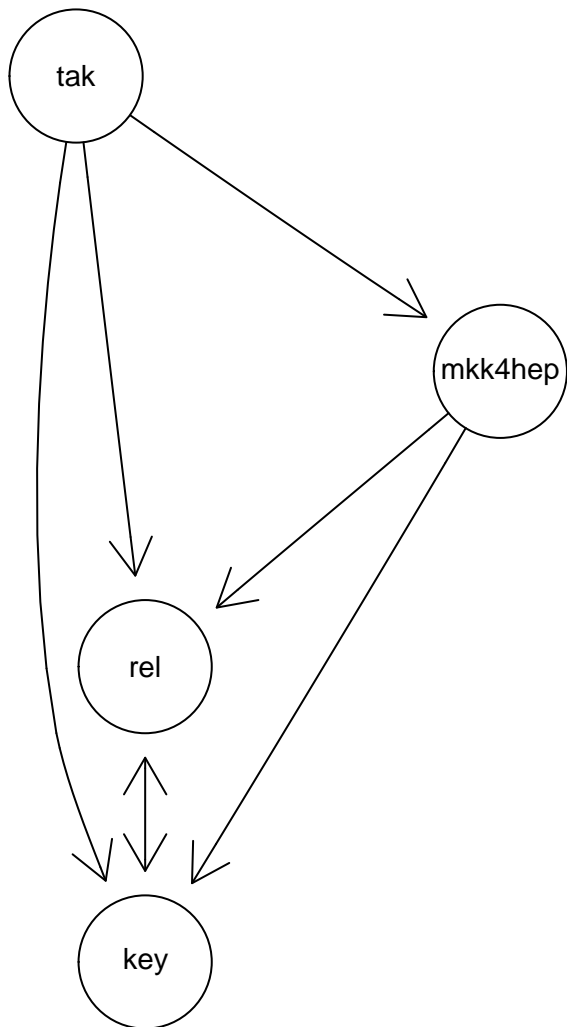

Supplement: Additional file 3 — nem_2.0.0. R package for nested effect models [file 1471-2105-8-386-S3.zip › solution18_orig.pdf]

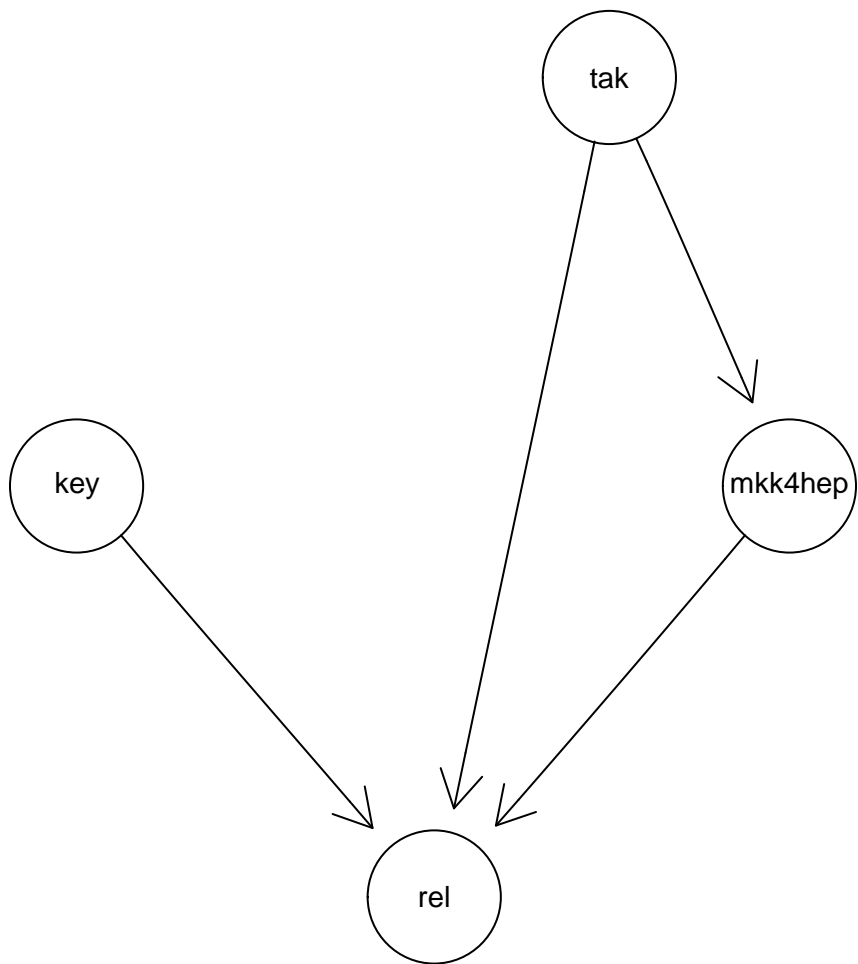

Supplement: Additional file 3 — nem_2.0.0. R package for nested effect models [file 1471-2105-8-386-S3.zip › solution19_orig.pdf]

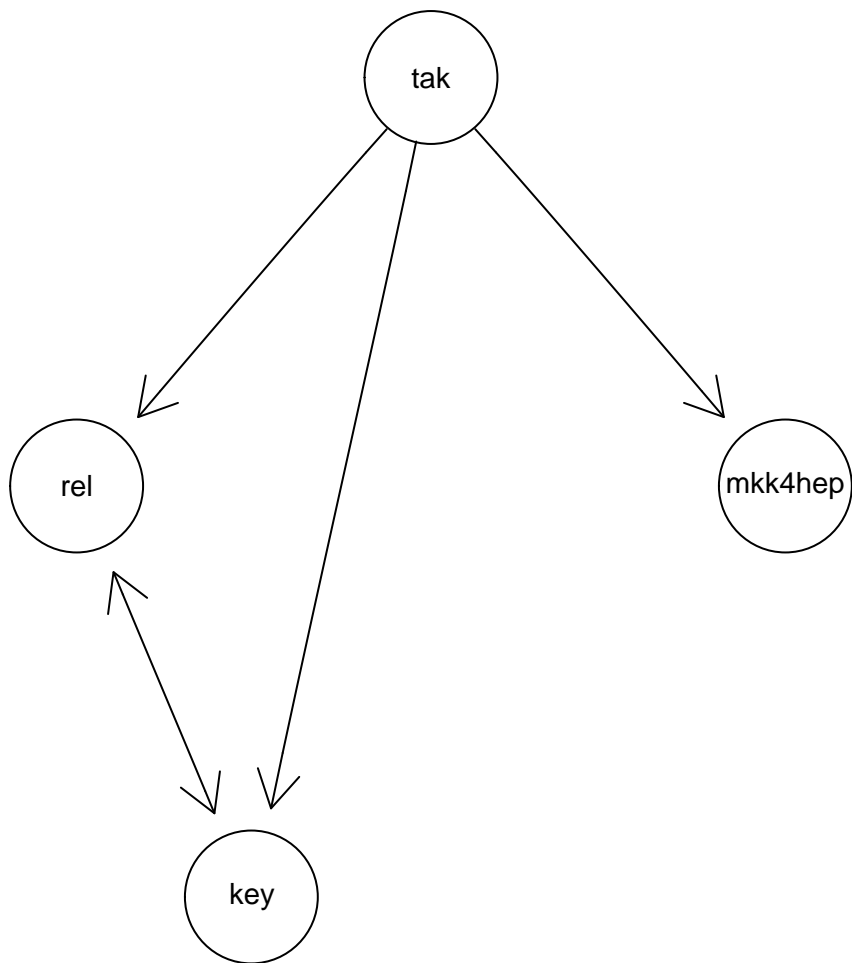

Supplement: Additional file 3 — nem_2.0.0. R package for nested effect models [file 1471-2105-8-386-S3.zip › solution1_orig.pdf]

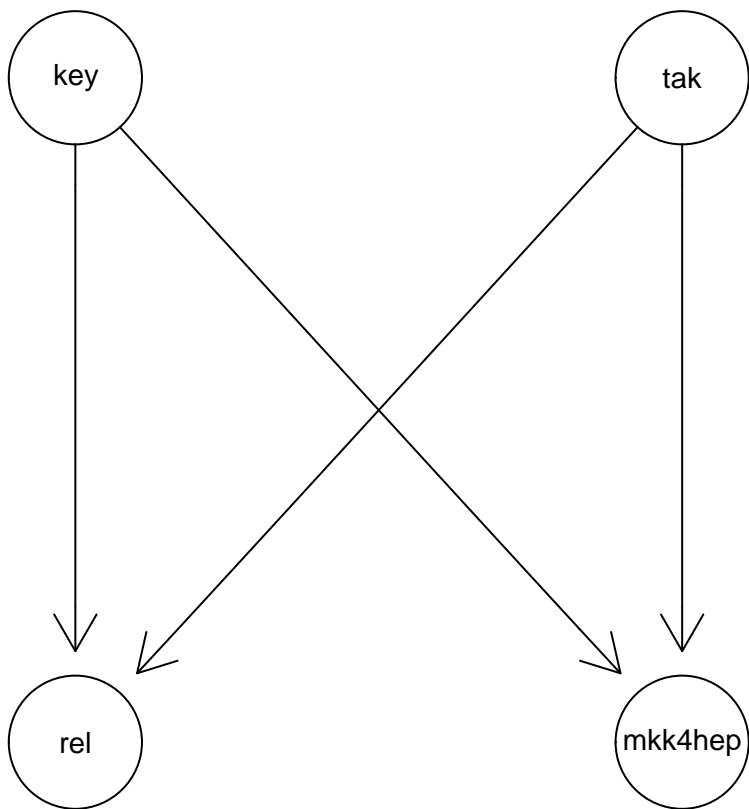

Supplement: Additional file 3 — nem_2.0.0. R package for nested effect models [file 1471-2105-8-386-S3.zip › solution20_orig.pdf]

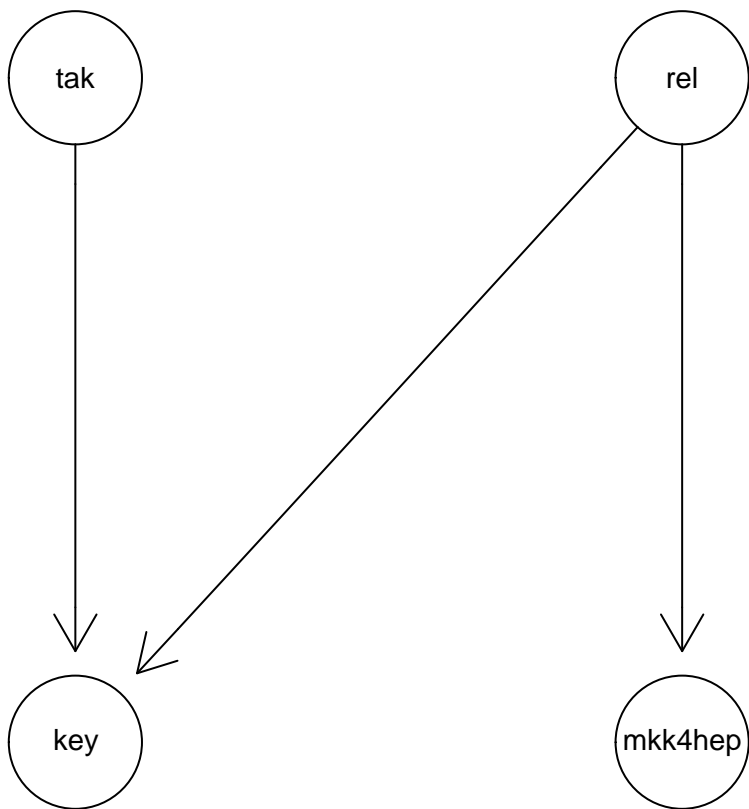

Supplement: Additional file 3 — nem_2.0.0. R package for nested effect models [file 1471-2105-8-386-S3.zip › solution21_orig.pdf]

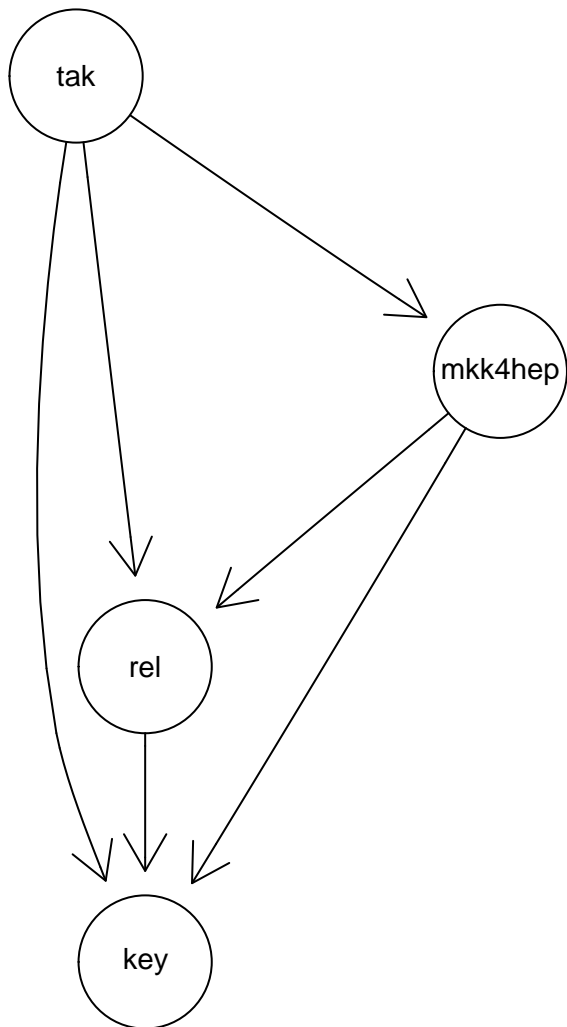

Supplement: Additional file 3 — nem_2.0.0. R package for nested effect models [file 1471-2105-8-386-S3.zip › solution22_orig.pdf]

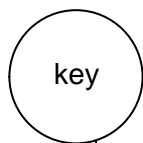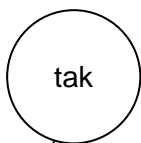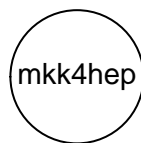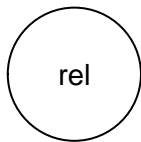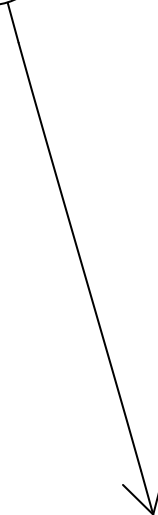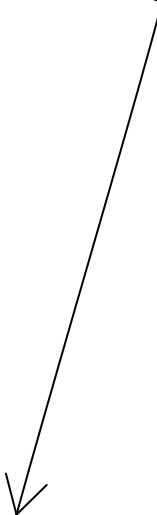

Supplement: Additional file 3 — nem_2.0.0. R package for nested effect models [file 1471-2105-8-386-S3.zip › solution23_orig.pdf]

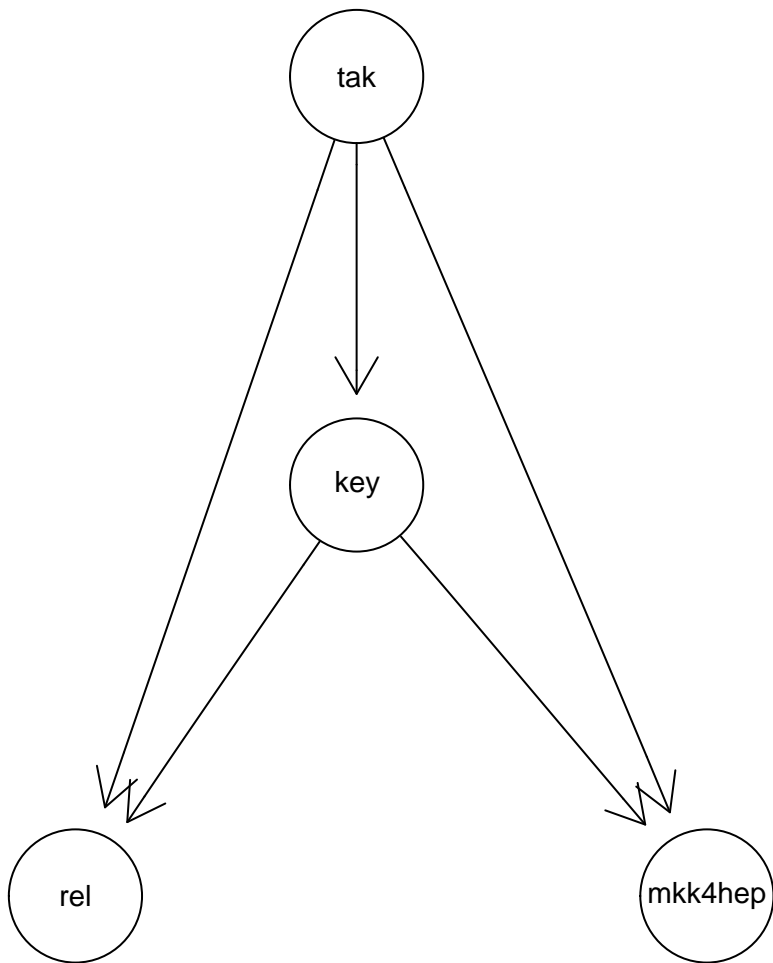

Supplement: Additional file 3 — nem_2.0.0. R package for nested effect models [file 1471-2105-8-386-S3.zip › solution24_orig.pdf]

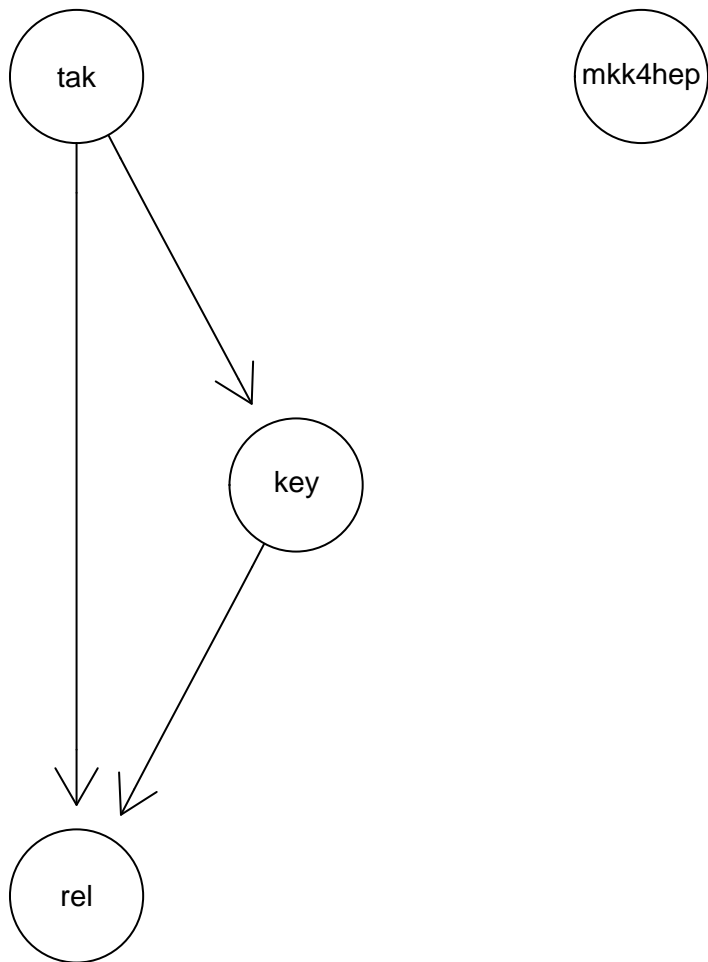

Supplement: Additional file 3 — nem_2.0.0. R package for nested effect models [file 1471-2105-8-386-S3.zip › solution25_orig.pdf]

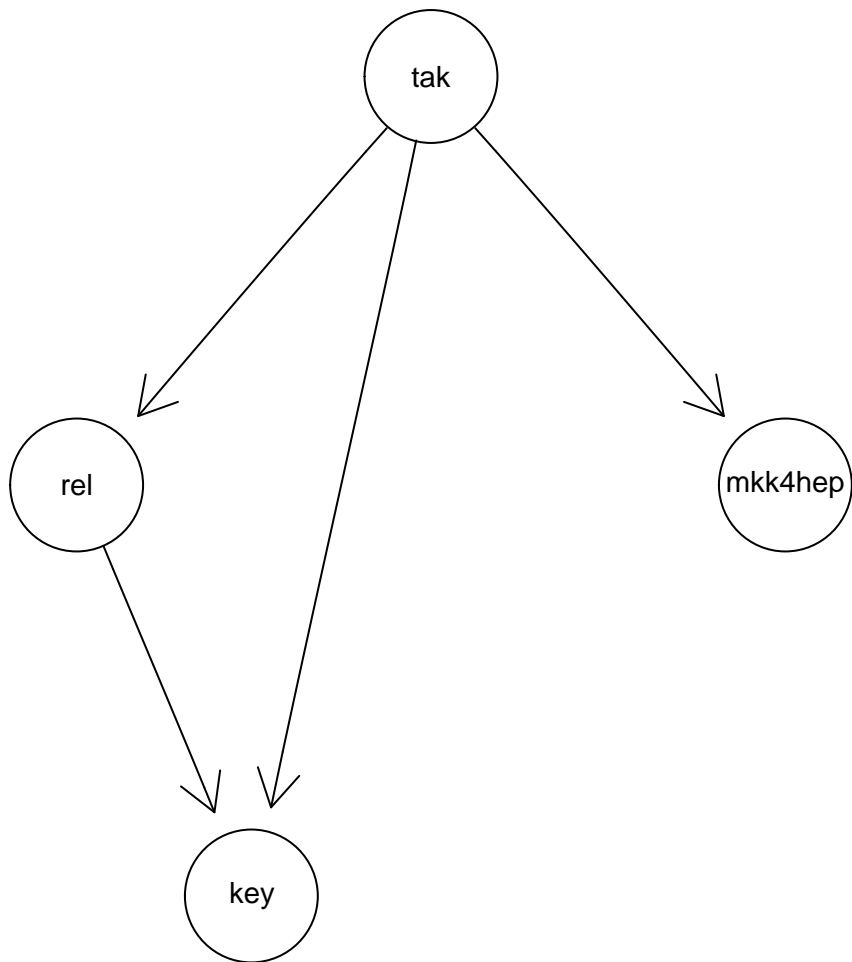

Supplement: Additional file 3 — nem_2.0.0. R package for nested effect models [file 1471-2105-8-386-S3.zip › solution2_orig.pdf]

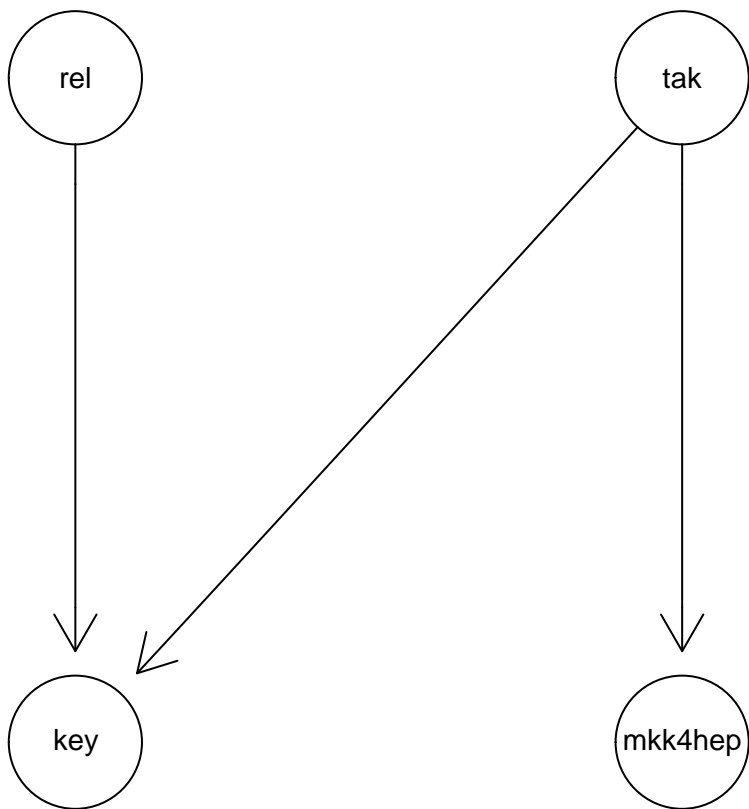

Supplement: Additional file 3 — nem_2.0.0. R package for nested effect models [file 1471-2105-8-386-S3.zip › solution3_orig.pdf]

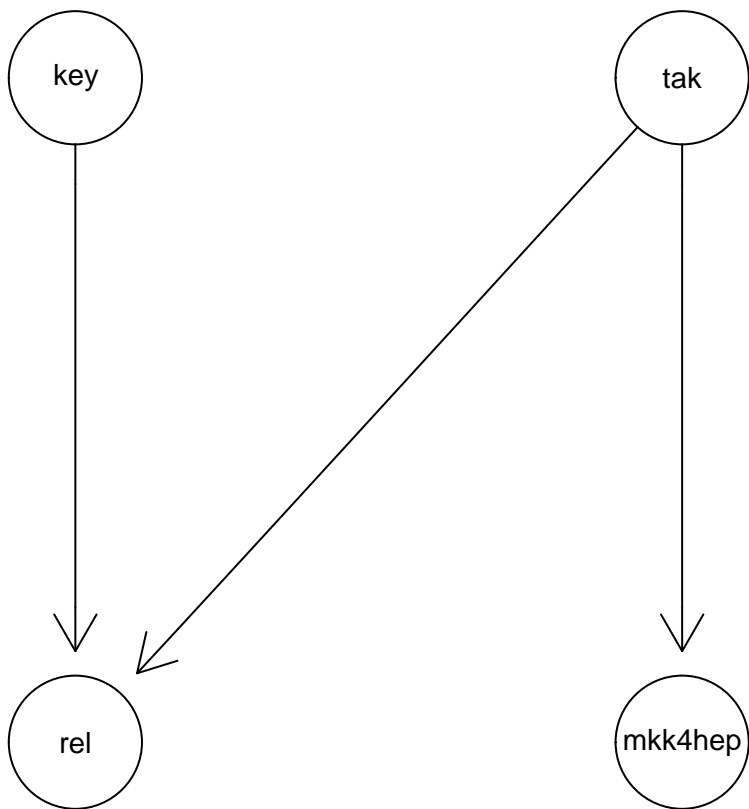

Supplement: Additional file 3 — nem_2.0.0. R package for nested effect models [file 1471-2105-8-386-S3.zip › solution4_orig.pdf]

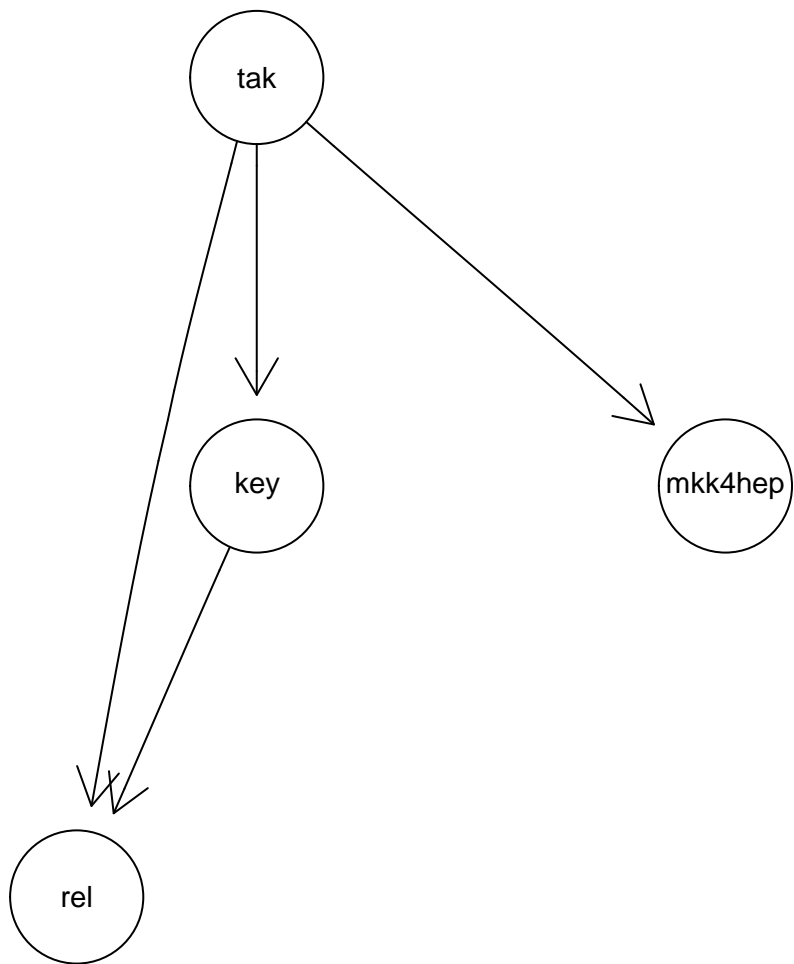

Supplement: Additional file 3 — nem_2.0.0. R package for nested effect models [file 1471-2105-8-386-S3.zip › solution5_orig.pdf]

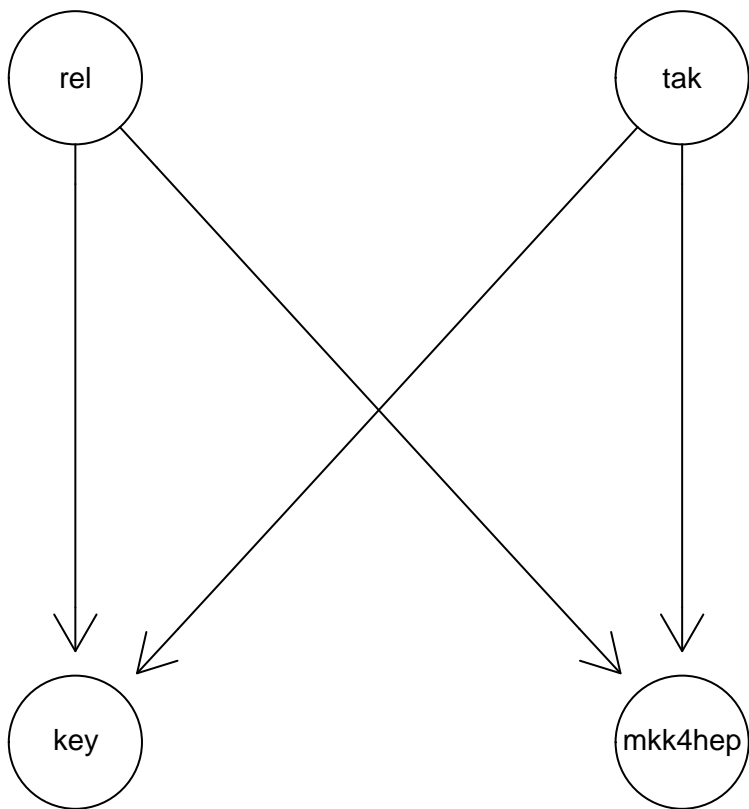

Supplement: Additional file 3 — nem_2.0.0. R package for nested effect models [file 1471-2105-8-386-S3.zip › solution6_orig.pdf]

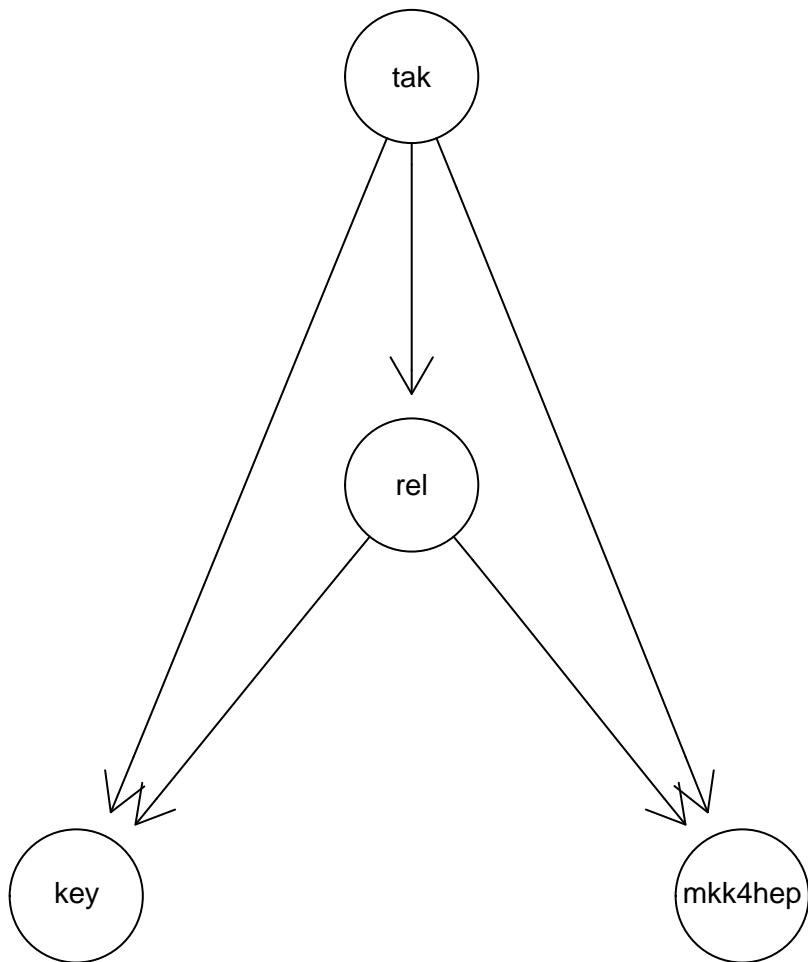

Supplement: Additional file 3 — nem_2.0.0. R package for nested effect models [file 1471-2105-8-386-S3.zip › solution7_orig.pdf]

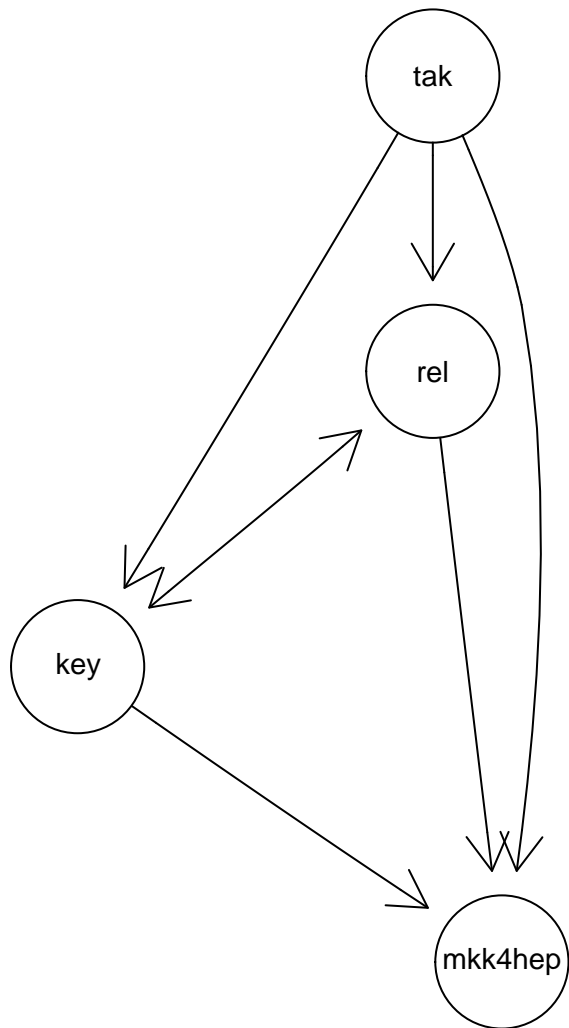

Supplement: Additional file 3 — nem_2.0.0. R package for nested effect models [file 1471-2105-8-386-S3.zip › solution8_orig.pdf]

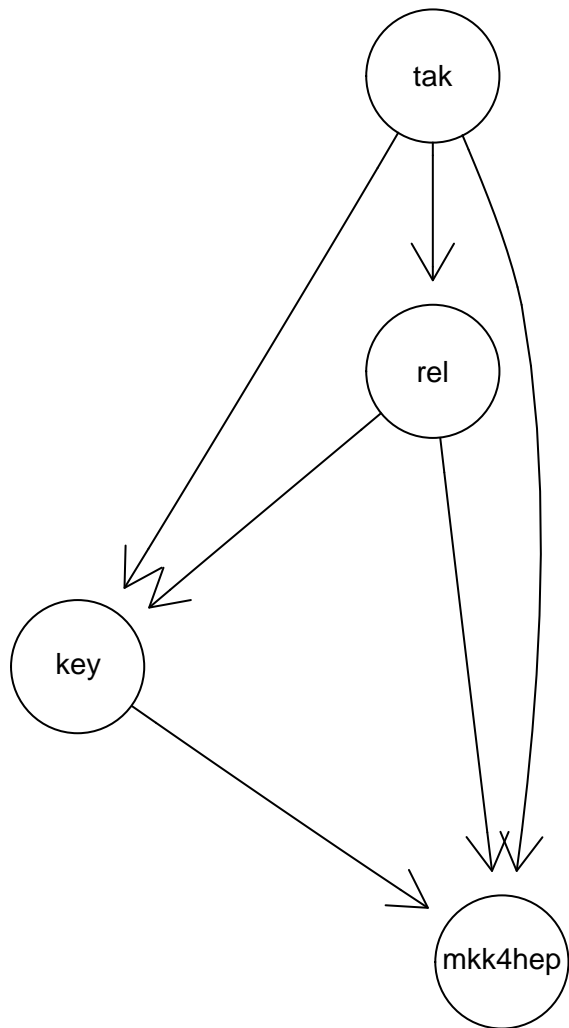

Supplement: Additional file 3 — nem_2.0.0. R package for nested effect models [file 1471-2105-8-386-S3.zip › solution9_orig.pdf]
